# Tungsten-Enabled Diels–Alder Cycloaddition and Cycloreversion of Arenes and Alkynes: Divergent Synthesis of Highly Functionalized Barrelenes and Arenes

**DOI:** 10.1021/jacs.5c08320

**Published:** 2025-08-08

**Authors:** Jeremy M. Bloch, Evan Savelson, Alvin Q. Meng, Megan N. Ericson, Ishaan U. Patel, Diane A. Dickie, Jetze J. Tepe, W. Dean Harman

**Affiliations:** Department of Chemistry, 2358 University of Virginia, Charlottesville, VA 22904, United States

## Abstract

The Diels–Alder reaction of benzenes remains a significant synthetic challenge, owing to their highly stabilized aromatic cores. In this work, the dearomatization agent {WTp­(NO)­(PMe_3_)} is used to promote Diels–Alder reactions of dihapto-coordinated (η^2^) benzenes with alkynes. The resulting η^2^-barrelene complexes can be oxidized to liberate intact barrelenes. Alternatively, mild pyrolysis leads to the extraction of the corresponding tungsten-acetylene complex and concomitant formation of new arenes possessing substituents originating from the acetylene dienophiles.

## Graphical abstract

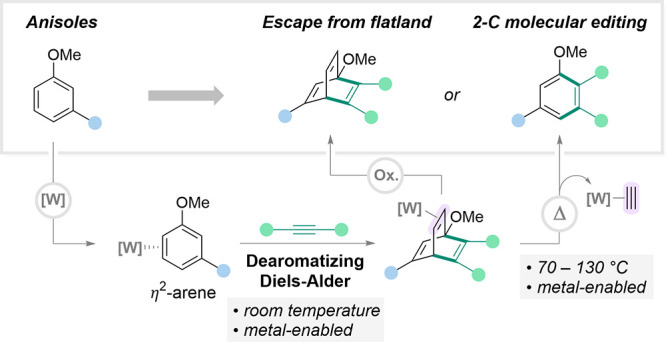

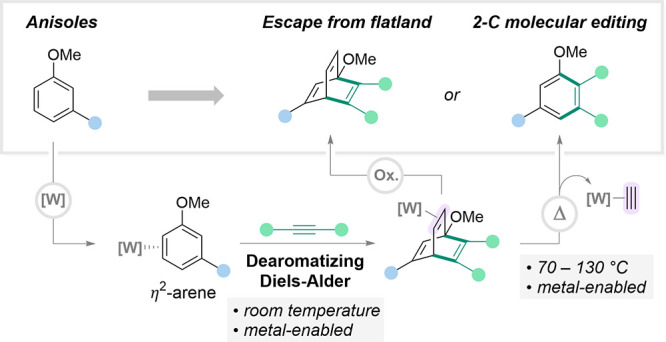

## Introduction

The Diels–Alder (DA) reaction is one of the most versatile reactions in organic chemistry, forming complex cyclic structures in a single step with predictable regio- and stereocontrol.^1^ While this reaction is relatively facile with activated dienes, DA reactivity is far more difficult to achieve with aromatic substrates.^2^ This lack of reactivity is attributed to the large energetic cost required to overcome the thermodynamic stabilization provided by aromaticity.^2^ As a result, DA reactions of benzenes are largely unrealized, with the few examples reported requiring Lewis acid additives, high reaction temperatures and pressures, or intramolecular pathways (Scheme 1A).^3–6^

**Scheme 1:**
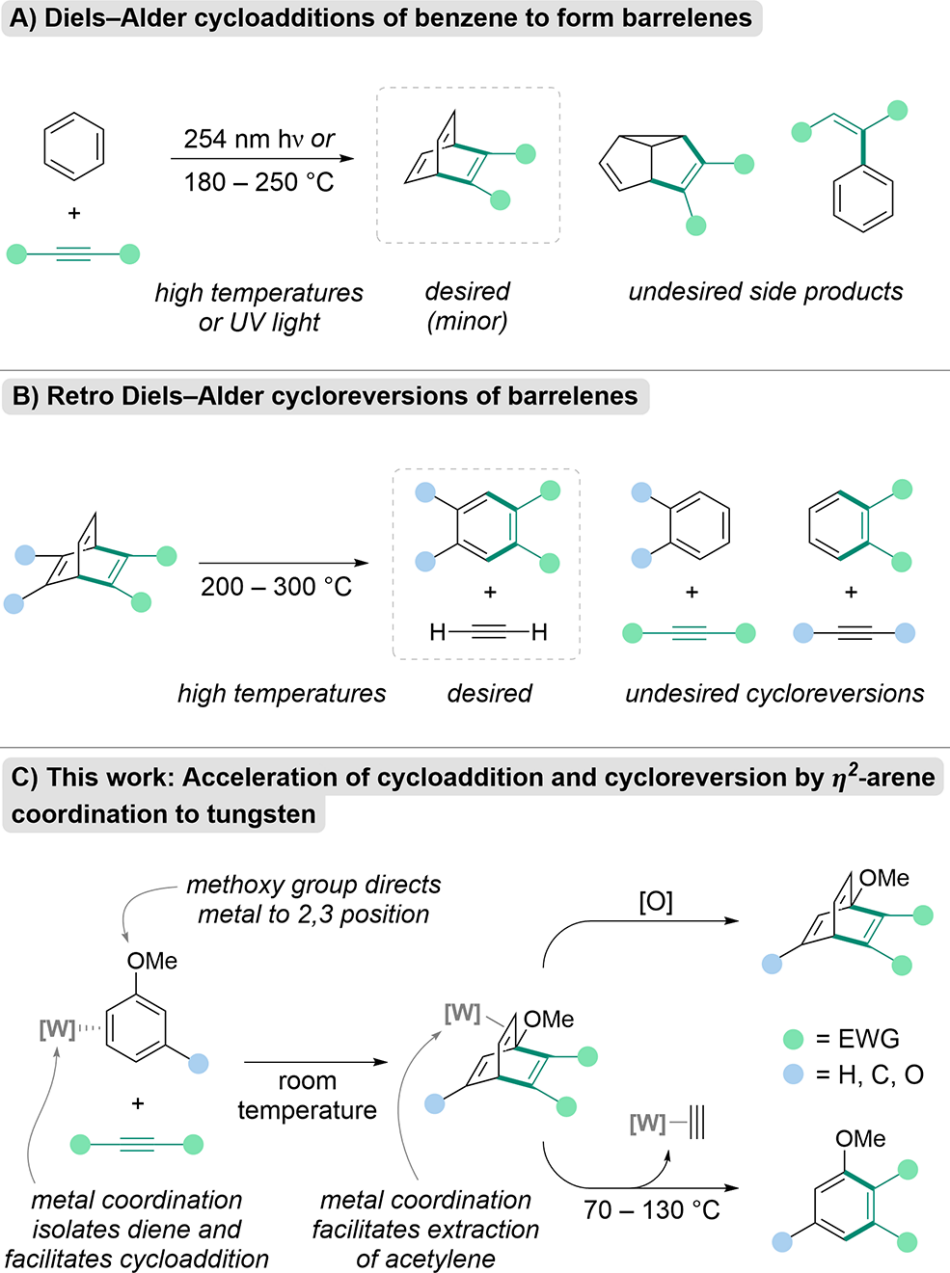
Overview of Proposed Work^a^

a [W] = {WTp­(NO)­(PMe_3_)}; [O] = oxidant.

Despite these challenges, a general method for effecting DA reactions with arenes would improve access to some otherwise synthetically challenging, topologically complex molecules. For example, barrelenes (bicyclo[2.2.2]­octa-2,5,7-trienes) can be formed, in principle, from the DA reaction of an arene (diene) with an alkyne (dienophile). The transformation of arenes into barrelenes would additionally represent a unique and direct approach to “escape from flatland,” introducing topological complexity into drug-like molecules.^7,8^ This approach would allow for the synthesis of potential arene isosteres with similar exit vectors to substituted arenes directly from the parent scaffold.^9,10^ Additionally, barrelenes are of theoretical and practical interest due to their unique electronic structures and their wide-ranging uses as ligands in transition metal complexes, as fluorescent materials, and as monomers for ring-opening metathesis polymerization reactions.^11–14^ Despite these promising applications, barrelenes remain a largely underexplored scaffold due to a dearth of straightforward syntheses.^15,16^

Barrelenes also hold promise as synthons to novel benzenes: The two-carbon molecular editing of a benzene can be envisioned through a DA cycloaddition/retro-DA (rDA) cycloreversion sequence (e.g., Scheme 1B). In recent years, the use of molecular editing in drug discovery has enabled chemists to “edit” existing drug skeletons in order to optimize safety and efficacy, while avoiding cost- and labor-intensive *de novo* syntheses.^17–20^ Given the prevalence of benzene rings in drugs, methods for editing this group would be particularly valuable.^21^ While methods are available for converting heteroarenes into other heteroarenes or carbocyclic arenes, far fewer molecular editing methodologies are known for interconversion between carbocyclic arenes.^17,22,23^

Over the past three decades, we have developed a family of highly π-basic transition metal (Os, Re, Mo, W) dearomatization agents that operate by binding arenes through only two carbons. This *η* ^2^-coordination mode is stabilized by a metal d_π_ → arene π* backbonding interaction.^24–26^ With two carbons of the arene π-system “locked” by their interaction with the metal, the four unbound carbons exhibit reactivity resembling a conjugated diene. We have previously reported instances of *η* ^2^-arene complexes participating in DA reactions with alkene dienophiles, including examples of *η* ^2^-benzene, *η* ^2^-naphthalene, and *η* ^2^-2-(dimethylamino)­pyridine.^27–29^ However, analogous reactivity of *η* ^2^-arene complexes using alkyne dienophiles to form *η* ^2^-barrelene complexes has not been well established.^30^ We hypothesized that the dearomatization agent {WTp­(NO)­(PMe_3_)} ([W]; Tp = hydridotris­(pyrazolyl)­borate) could enable DA reactions of *η* ^2^-arenes with alkyne dienophiles to form *η* ^2^-barrelene complexes. Further, we questioned whether the metal could accelerate not only the DA cycloaddition but a rDA reaction as well, thereby effecting the formation of newly substituted arenes (Scheme 1C).

## Results and Discussion

### Synthesis of *η* ^2^-Barrelene Complexes

We began our preliminary investigation using the parent complex [W]–(*η* ^2^-benzene). However, decomplexation of benzene was observed to proceed at a faster rate than the DA reaction for all dienophiles tested below. Seeking a more electron-rich system, we turned our attention to the analogous *η* ^2^-anisole complex (**1**). This complex features an activating π-donor group and can be prepared via a four-step pathway from W­(CO)_6_ on a decagram scale.^31^ This complex is fluxional with respect to the site of coordination.^26^ However, the 2,3-*η* ^2^ isomer dominates, which places the methoxy group at the terminal position of the “diene” fragment.^26^

A THF solution of **1** was treated with an excess of dimethyl acetylenedicarboxylate (DMAD) at room temperature and monitored by ^1^H NMR spectroscopy. Fortunately, over a period of 1 h, the complex was converted into the desired *η* ^2^-barrelene complex **5**.^30^ Further experiments showed that **1** would readily participate in DA reactions with other reactive alkynes as well, providing *η* ^2^-barrelene complexes (**4**–**7**) in good yield (Scheme 2). DA reactions with [W]–(*η* ^2^-1,3-dimethoxybenzene) (**2**) also proceeded to form *η* ^2^-barrelene complexes (**8**–**9**) in accordance with our initial observation.^30^ Additionally, this cycloaddition reaction can be carried out with 3-alkylated anisoles (vide infra), but 2- or 4-alkylated anisoles appear to be problematic (A full list of attempted alkyne/*η* ^2^-arene reaction combinations can be found in Scheme S1 of the SI).

**Scheme 2:**
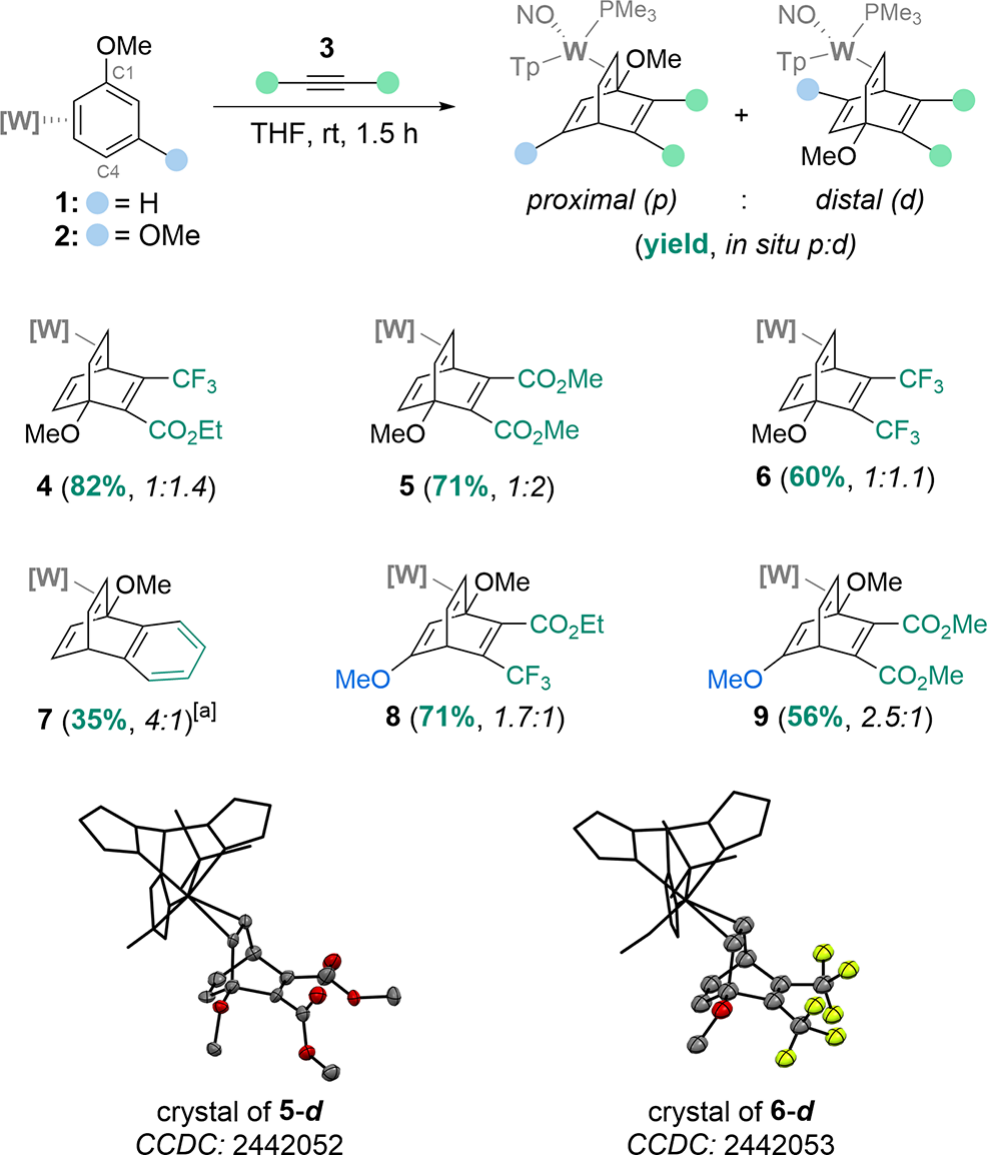
Preparation of *η* ^2^-Barrelene Complexes from *η* ^2^-Coordinated Arenes^a^

a For the crystal structures of **5** **-** ***d*** and **6** **-** ***d***, the tungsten fragment [W] is shown in capped sticks style while the *η* ^2^-barrelene ligands are shown in ORTEP style (50% probability for **5-** ***d*,** 30% probability for **6-** ***d***; see SI for full ORTEP diagrams). Hydrogen atoms and the minor orientation of disordered atoms are omitted for clarity. [a] **3d** (benzyne) prepared from *o*-trimethylsilylphenyl triflate.

Terminal alkynes (e.g., 3-butyn-2-one, methyl propiolate, diethyl ethynylphosphonate failed to react with **1** in the desired DA pathway. However, in one case (ethynyl *p*-tolyl sulfone), a product was isolated that appeared to be the result of a Michael addition to C4 of the *η* ^2^-bound anisole complex, followed by a secondary reaction in which the resulting oxocarbenium intermediate reacted with an additional equivalent of the (deprotonated) alkyne (see SI; compound **S1**).

As the tungsten atom is a stereogenic center, the *η* ^2^-coordination of anisole leads to the formation of coordination diastereomers that differ in the orientation of the methoxy group relative to the PMe_3_ ligand. In solution, the equilibrium ratio of the proximal (***p***) and distal (***d***) isomers is ∼3:1 (***p***: ***d***).^31^ In all anisole-derived barrelene complexes except **7**, the dominant isomer formed in solution and isolated was the ***d*** isomer, even though the ***p*** isomer of **1** is favored in solution. These observations suggest that the DA reaction occurs under Curtin-Hammett conditions, with the DA transition state (TS) of **1-** ***d*** being lower in energy than that of **1-** ***p***. Similar observations were reported for DA reactions of **1** with alkenes.^30^ Previous density functional theory (DFT) calculations have shown that the {WTp­(NO)­(PMe_3_)} system has a thermodynamic preference to orient allylic positive charges of bound ligands distal to the PMe_3_ ligand.^30^ Given that we identified a mechanism that invokes significant charge separation for both **1-** ***p*** and **1-** ***d*** (*vide infra*), the formation of an allylic oxocarbenium-like moiety is expected to be thermodynamically more favorable for **1-** ***d*** than for **1-** ***p***. This leads to the ***d*** isomer of the *η* ^2^-barrelene complex being formed in excess for cases where the rate of interconversion between **1-** ***p*** and **1-** ***d*** in solution is much faster than the rate of cycloaddition. Conversely, in the case of **7**–**9**, we posit that the rate of cycloaddition is greater than the rate of interconversion between **1-** ***p*** and **1-** ***d*** (or **2-** ***p*** and **2-** ***d***) such that the product distribution reflects the equilibrium preference of **1** and **2** to orient the allylic methoxy group proximal to the PMe_3_ ligand.

### Oxidative Decomplexation of Free Barrelenes

The oxidation of [W] weakens the extent of its backbonding, allowing for the removal of the organic ligand. We have utilized this oxidative decomplexation strategy extensively in the past to prepare a variety of carbocyclic alkenes.^31^ However, out of the limited examples of *η* ^2^-barrelene complexes previously reported, isolation of free barrelenes in synthetically useful quantities has remained elusive; previous attempts to liberate barrelenes from this tungsten system have been unsuccessful, and the oxidative decomplexation of a barrelene from a rhenium system was marred by significant rDA product [1:1 barrelene:arene (rDA)] and was not isolated.^27,30^

Several oxidants were screened for the decomplexation of barrelene complex **4**, identifying both 2,3-dichloro-5,6-dicyano-1,4-benzoquinone (DDQ) and ferrocenium hexafluorophosphate (FeCp_2_PF_6_) as suitable oxidants for forming free barrelene **10** at low temperatures (Scheme S1). After reaction optimization, complexes **4**–**8** were subjected to both sets of oxidative conditions, and yields of the reaction mixture were monitored by quantitative NMR (qNMR). Between the two oxidants, the higher yielding option was used for product isolation (Scheme S2). Preferable decomplexation conditions proved highly substrate-dependent, with DDQ being the preferred oxidant for forming **10** and **12**, while FeCp_2_PF_6_ performed better on **11**, **13**, and **14** (Scheme 3). In particular, barrelene **12** showed a sharp drop in yield upon isolation. This appears to result from the volatility of **12**, as the recoverable mass decreased *in vacuo*. The *η* ^2^-barrelene complex **8** was noted to undergo hydration of the enol ether functionality to a hemiacetal or ketone-containing bicyclic species when exposed to trace amounts of water (see SI), and this trend continued upon decomplexation, whereby a complex mixture of hydrated and hydrolyzed barrelenes was observed. Upon aqueous workup, this mixture could be converted into the ketone substrate **14** in 47% yield. While barrelene derivative **14** is chiral and the precursor *η* ^2^-arene **2** can be enantioenriched,^32^ the equilibrium between the ***p*** and ***d*** forms of **2** prevents the preparation of **14** in a highly enantioenriched form. Notably, **10**–**14** all share a 1-methoxy substituent on the barrelene scaffold, representing a structurally uncommon motif. To our knowledge, all previously synthesized 1-alkoxybarrelenes have contained fused aromatic rings (e.g., **13**).^33–35^

**Scheme 3:**
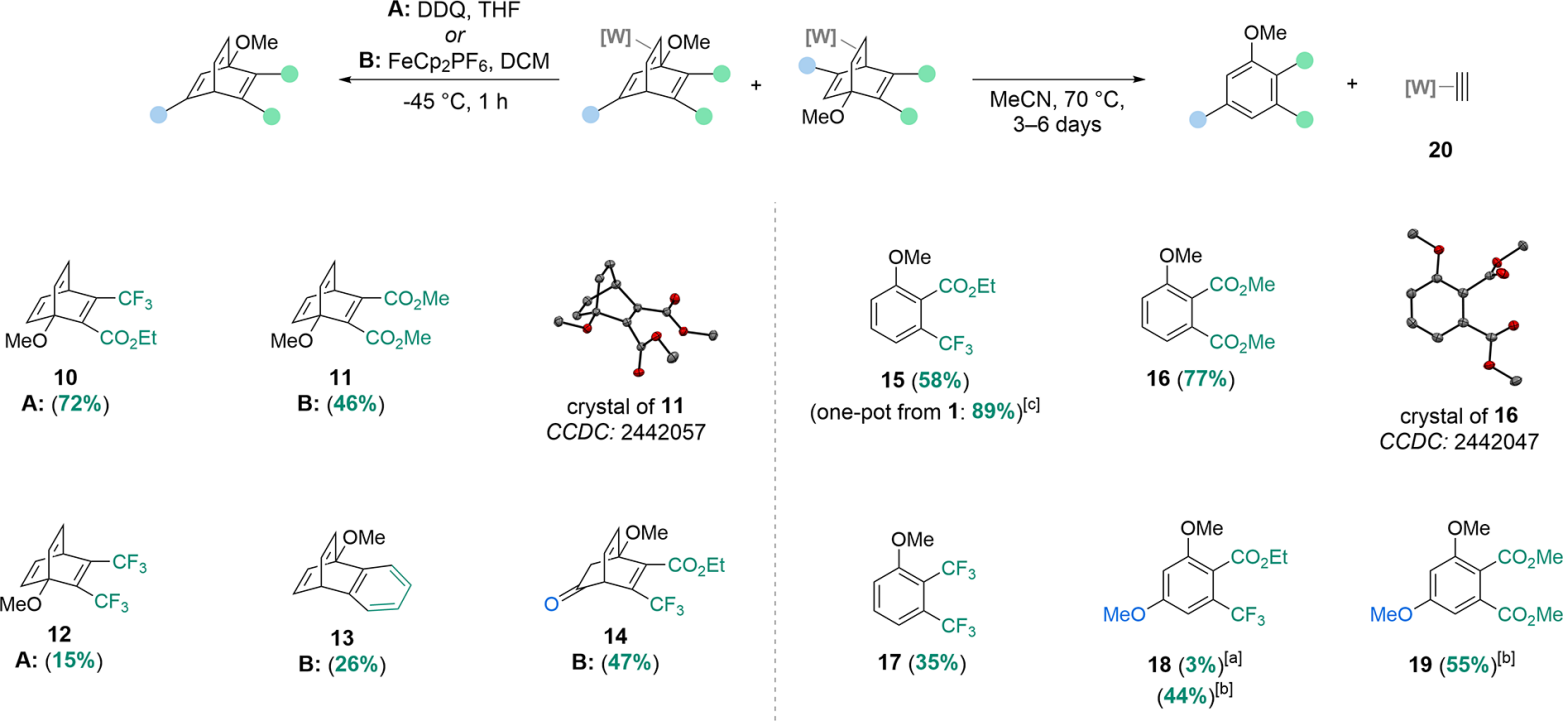
Preparation of Barrelenes and Substituted Arenes from η ^2^-Barrelene Complexes^a^

a [a] MeCN, 70 °C, 17 days. [b] Toluene, reflux, 2–5 days. [c] **1** and **3a** combined in 1,4-dioxane, rt to 90 °C, 41 h. Crystal structures of **11** and **16** are shown in ORTEP style (50% probability) with hydrogen atoms omitted for clarity.

### Synthesis of Molecularly Edited Arenes from *η* ^2^ *-*Barrelene Complexes

In each case of barrelene decomplexation (except for **13**), small amounts of corresponding rDA product (arenes) were isolated alongside the desired free barrelene products (see SI for ratios). This encouraged us to investigate rDA reactions for the free barrelenes. However, heating barrelenes **10**, **11**, and **13** at modest temperatures (70 °C) failed to induce rDA reactivity. Free barrelenes typically require high temperatures (∼200–300 °C) and/or catalysis to undergo rDA, suggesting that these reactions possess a large kinetic barrier.^36^ Therefore, we hypothesized that [W] was not only facilitating the forward DA reaction but was also able to accelerate the cycloreversion reaction. To test this, the thermal stabilities of the *η* ^2^-barrelene complexes were determined. Complex **5** was monitored by ^1^H NMR at 70 °C in MeCN-*d*_3_ for 3 days. The corresponding spectra indicated the formation of a new tungsten complex along with the presence of new aromatic signals. Two-dimensional NMR analysis and single-crystal X-ray diffraction revealed that the two new species were [W]–(*η* ^2^-acetylene) (**20**) and anisole derivative (**16**). These products were theorized to form via a thermally-promoted rDA of the parent *η* ^2^-barrelene complex, producing a two-carbon molecular edit of the original arene (Scheme 3). Therefore, all other **1**-derived *η* ^2^-barrelene complexes were tested for their ability to undergo rDA reactions. Gratifyingly, **4**–**6** underwent the rDA reaction under the same conditions, providing benzenes **15**–**17**. Further, compound **15** could be formed in higher yield (89%) directly from **1** and **3a** in a one-pot procedure. We note that structurally characterized *d* ^6^ *η* ^2^-acetylene complexes (“2e” donor) for Group 6 are uncommon, yet **20** is thermally stable at temperatures above 100 °C.^37,38^

Interestingly, rDA reactions of the 1,3-dimethoxybenzene-derived *η* ^2^-barrelene complex **8** resisted facile cycloreversion under these conditions. After heating **8** for 17 days, the major organic product obtained (**S2,** 9%; see SI) resulted from a ring-opening side reaction, whereas rDA produced the minor product (**18**, 3%). This side-reaction was ascribed to the ability of the methoxy group at the 3-position to stabilize the partial charge buildup in the TS of ring-opening through π-donation. Therefore, we hypothesized that using a nonpolar solvent would favor rDA and disfavor the polar ring-opening mechanism. To test this, **8** was refluxed in toluene for 5 days. As anticipated, this change in solvent provided **18** in significantly higher yield (44% vs 3%) and produced none of side product **S2**. These alternate toluene-based reaction conditions were also used to generate rDA product **19** from *η* ^2^-barrelene complex **9** with no ring-opened side product.

While other methods have been reported for the synthesis of arenes similar to (or the same as) arenes **15**–**19** via DA/rDA reactions using alkynes, the diene partners used for those methods are typically not derived from benzenes.^39–42^ An exception to this was reported by Birch and Wright, in which 1,3-dimethoxybenzene underwent Birch reduction to 1,3-dimethoxycyclohexa-1,3-diene, which was subsequently allowed to react with DMAD in an Alder-Rickert reaction to form the same arene as **19**.^43^

A recent report by Bouffard and coworkers outlined an elegant approach for two-carbon molecular editing of benzenes by utilizing 1,3-diazazoniaallene cations (DAAA^+^) to form DAAA^+^–barrelene adducts, which undergo cycloreversion to form new aromatic rings.^22^ While this approach constitutes a significant advance, the scope is limited to alkyl benzenes; anisoles were determined to be unsuitable as reaction partners.^22^ In the present study, the synthesis of barrelenes and molecularly edited benzenes occurs with complete regiocontrol by virtue of the π-donating substituents. In this regard, the method described herein provides a useful complement to the Bouffard process.

### Synthesis of Molecularly Edited Tramadol Analogs

To demonstrate the utility of this methodology, we sought a bioactive compound that could be combined with an alkyne to obtain both a barrelene and a new arene. We focused on the FDA-approved pain medication tramadol (Scheme 4).^44^ An unregulated analog of tramadol (**24**) was prepared (see SI) for synthetic testing due to accessibility and safety concerns. When compound **24** was combined with **1**, a ligand exchange reaction generated [W]–(*η* ^2^-**24**) *in situ*.^25,26^ The complexed tramadol analog could further undergo DA reactivity when exposed to **3a**, successfully forming *η* ^2^-barrelene complex **21** in 20% yield. **21** was found to undergo effective decomplexation when exposed to DDQ, providing barrelene **22** in 73% yield. Complex **21** proved more thermally stable than many other isolated *η* ^2^-barrelene complexes and resisted cycloreversion under conditions successfully employed for similar rDA reactions (MeCN, 70 °C; 1,4-dioxane, 90 °C; toluene, reflux). However, heating **21** in xylenes (130 °C) generated the desired, molecularly edited product **23** in 29% yield (unoptimized). This preliminary success highlights the potential to edit other arene-containing bioactive molecules, and such studies are currently underway.

**Scheme 4:**
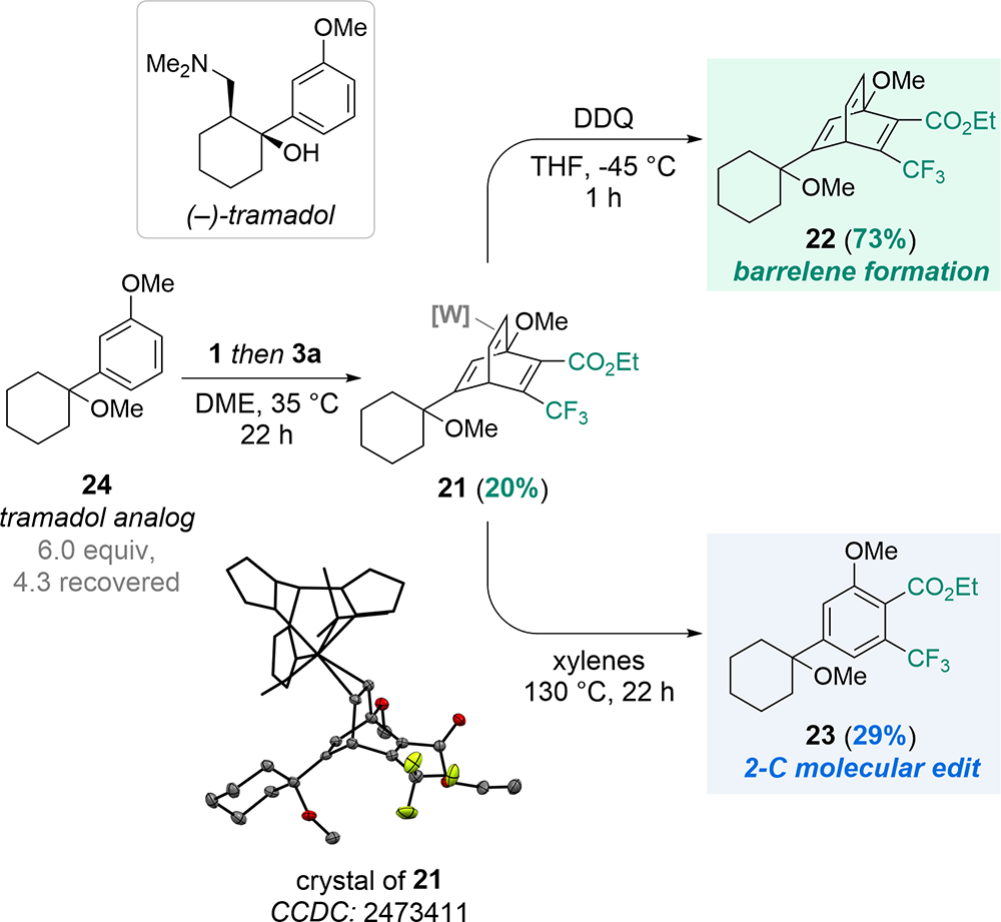
Molecular Edits of Tramadol Analog **24** to form Barrelene **22** and Arene **23.** For the crystal structure of **21**, the tungsten fragment [W] is shown in capped sticks style while the η^2^-barrelene ligand is shown in ORTEP style (50% probability) with hydrogen atoms omitted for clarity.

### Computational and Kinetic Studies

We next sought to computationally investigate both DA reactions of **1** and rDA reactions of *η* ^2^-barrelene complexes to better understand their reactivity (Scheme 5A; M06/6-31G­(d, p)/LANL2DZ on W, implicit THF solvation), using the compound methyl 4,4,4-trifluoro-2-butynoate as a computationally simplified model for the experimental dienophile **3a**.

**Scheme 5:**
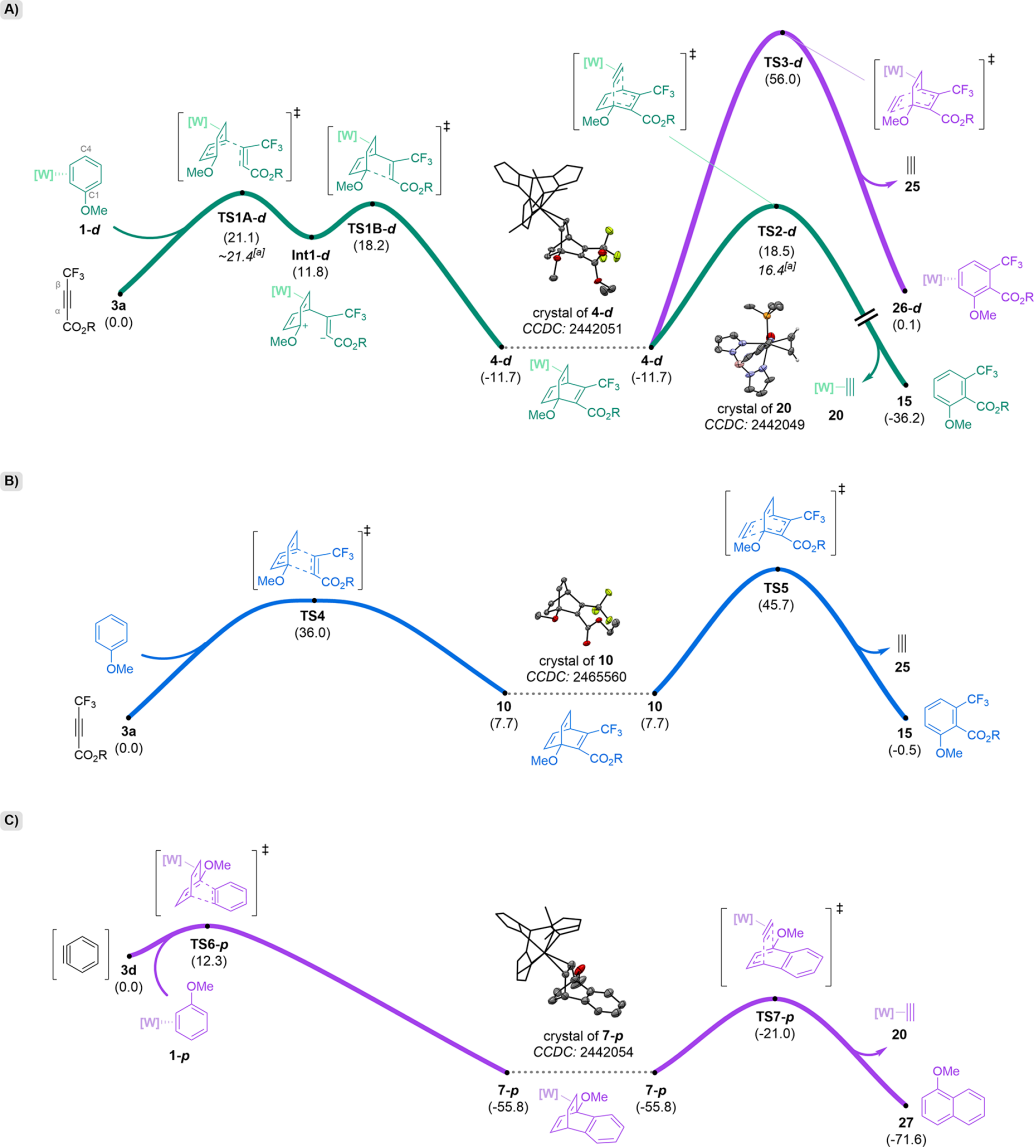
DFT (M06/6–31G­(d,p)/LANL2DZ on W/THF Solvation; kcal/mol) Studies Showing Tungsten-Facilitated Cycloaddition and Cycloreversion Reactions^a^

a For DFT studies, the R-group of **3a** was changed to methyl (from ethyl) to simplify the calculations. For the crystal structures, the tungsten fragment [W] of **4-** ***d*** and **7-** ***p*** are shown in capped sticks style while their remaining atoms and all atoms of **20** are drawn in ORTEP style (50% probability for **4-** ***d*** and **20**, 30% probability for **7-** ***p***). All hydrogen atoms except the *η* ^2^-acetylene ligand H-atoms of **20** were omitted for clarity, as were solvent molecules and the minor position of disordered atoms. [a] Experimental value from kinetic studies (*vide infra*).

### Cycloadditions (DA)

We first compared the reaction between the anisole complex **1-** ***d*** and **3a** (Scheme 5A) to the analogous reaction between free anisole and **3a** (Scheme 5B). The cycloaddition between anisole complex **1-** ***d*** and dienophile **3a** was calculated to be thermodynamically favorable (ΔG_**3a→4‑** ***d***_ = −11.7 kcal/mol) with a moderate kinetic barrier (ΔG**_3a→TS1A-*d* ^‡^_** = 21.1 kcal/mol). In contrast, the cycloaddition of anisole and dienophile **3a** was thermodynamically unfavorable (ΔG_**3a→10**_ = 7.7 kcal/mol) with a significantly higher energy barrier for the rate-determining step (ΔG**_3a→TS4^‡^_** = 36.0 kcal/mol; ΔΔG_(__**3a→TS1A-** ***d***)‑**(3a→TS4)^‡^**_ = −14.9 kcal/mol), in line with experimental observations.

Notably, we found that the mechanism of *η* ^2^-barrelene formation likely exists on a continuum between asynchronous-concerted and wholly stepwise, dependent on the electronic characteristics of the dienophile and reaction conditions. For example, the pathway identified under the aforementioned DFT parameters for the formation of **4-** ***d*** was stepwise, involving the Michael-type intermediate **Int1-*d*** (Scheme 5A). In contrast, the pathway identified for the formation of **7-** ***p*** under these same parameters was concerted, albeit highly asynchronous (Scheme 5C; *vide infra*). We note that **3d** lacks an ester group with which to form a stabilized enolate-like intermediate. As such, these calculations should be considered solely in terms of energetics, rather than a prescription of the reaction mechanism as occurs in solution.

The kinetics of the DA reaction between **1** and **3a** were also tested experimentally (Figure 1A). Holding the dienophile in large excess to force pseudo-first-order conditions, the reaction between **1** and **3a** was tracked by ^1^H NMR, and integrations of **1** were monitored over time relative to a TMS internal standard. The observed rate constant *k*’ (*k*’ = *k*[1.98 M]) was determined to be (1.16 ± 0.18) × 10^–3^ s^–1^. As this DA process occurs under Curtin-Hammett conditions (i.e., **1** isomerizes much faster than cycloaddition), these experiments do not provide an individual pseudo-first-order rate constant (*k*’) for each of the individual diastereomers of **1** going to their respective ***p*** and ***d*** products.

**Figure 1:**
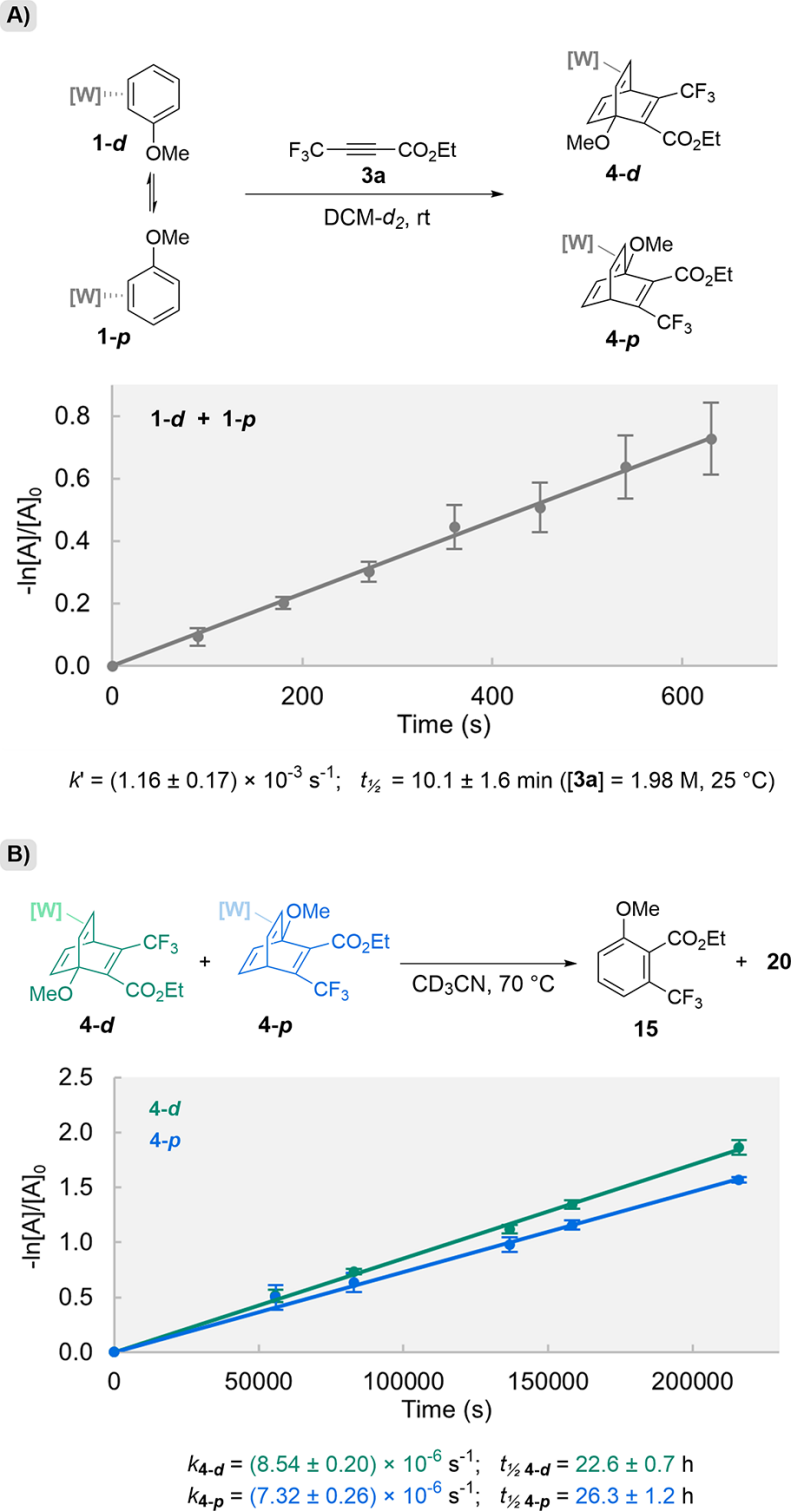
Kinetic plots for (A) Cycloaddition of **1** (mixture of coordination diastereomers) and **3a**; DCM-*d*_*2*_, rt. (B) Cycloreversion of **4-** *d* and **4-** *p*; CD_3_CN, 70 °C. Concentrations monitored by NMR relative to an internal standard (dimethyl sulfone or tetramethylsilane). Error bars indicate standard deviation (*n* = 3).

### Cycloreversion (rDA)

While the *η* ^2^-barrelene complex **4** readily undergoes rDA at elevated temperatures, the corresponding free barrelene **10** appears stable under the same conditions (Scheme 5B). To investigate these observations further, we studied the cycloreversion of **10** computationally and found that although this reaction is thermodynamically favorable (ΔG_**10→15+25**_ = −8.2 kcal/mol), it is accompanied by a high energy barrier (ΔG**_10→TS5_^‡^** = 38.0 kcal/mol) rendering this organic barrelene unreactive (Scheme 5B). This evidence indicates that the cycloreversion of **4** is made kinetically feasible by the coordination of [W] and subsequent release of **20**. All three possible modes of rDA reactivity for the barrelene complex **4-** ***d*** were investigated computationally, representing cycloreversion to **3a** and **1-** ***d*** (ΔG_**4‑** ***d*** **→3a+1‑** ***d***_ = 11.7 kcal/mol), cycloreversion to [W]–*η* ^2^-acetylene (**20**) and benzene **15** (ΔG_**4‑** ***d*** **→15+20**_ = −24.5 kcal/mol), and cycloreversion to free acetylene (**25**) and the tungsten complex of benzene **15** (**26-** ***d*;** ΔG_**4‑** ***d*** **→26‑** ***d*** **+25**_ = 11.8 kcal/mol) (Scheme 5A). Consistent with experimental results, the formation of **15** is the only thermodynamically favorable process, and unlike **TS1A-** ***d***, **TS2-** ***d*** appears to be synchronous in character in which acetylene is extracted from the barrelene by the tungsten (**20**).

Further kinetic studies were performed on **4-** ***d*** and **4-** ***p*** at 70 °C while normalized concentrations were monitored by ^1^H NMR (Figure 1B). First-order reactivity was observed for rDA of both **4-** ***d*** and **4** **-*p*** with rate constants of (8.54 ± 0.20) × 10^–6^ s^–1^ for **4-** ***d*** and (7.32 ± 0.26) × 10^–6^ s^–1^ for **4-** ***p***. Energy barriers for these transformations (calculated using the Eyring equation, *T* = 343 K) were found to be very similar for the two isomers (ΔG^‡^_**4‑** ***d*** **→TS2‑** ***d***_^*exp.*^ = 28.1 ± 0.0 kcal/mol; ΔG^‡^_**4‑** ***p*** **→TS2‑** ***p***_^*exp.*^ = 28.2 ± 0.0 kcal/mol).

Remarkably, the influence of [W] not only lowers the activation barrier of the cycloreversion (ΔΔG^‡^_(**4‑** ***d*** **→TS2‑** ***d***)‑(**10→TS5**)_ = −7.8 kcal/mol), but also dramatically stabilizes the combination of benzene and complexed acetylene (ΔΔG_(**4‑** ***d*** **→15+20)**‑(**10→22‑** ***d*** **+25)**_ = −16.3 kcal/mol). Whether this is a result of a destabilizing steric influence in the barrelene complex (**4-** ***d***), a stabilizing influence in the bound acetylene complex (e.g., M→L π_II_* or δ*i*nteractions), or both remains undetermined, but clearly the metal significantly facilitates both cycloaddition and cycloreversion reactions. We note that the alternative cycloreversion of **4-** ***d*** to *η* ^2^-benzene complex (**26d**) and free acetylene, which also would relieve the steric stress in the barrelene complex, has a barrier (ΔG^‡^_**4‑** ***d*** **→TS3‑** ***d***_ = 67.7 kcal/mol) and free energy difference (ΔG_**4‑** ***d*** **→26‑** ***d*** **+ 25**_ = 11.8 kcal/mol), even higher than the organic analog.

Lastly, the benzyne-derived *η* ^2^-barrelene complex **7-** ***p*** was investigated computationally to help explain its lack of rDA reactivity at elevated temperatures. Although the cycloreversion appears to be thermodynamically favorable (ΔG_**7‑** ***p*** **→27**_ = −15.8 kcal/mol), the transition state (**TS7-*p***; ΔG^‡^_**7‑** ***p*** **→TS7‑** ***p***_ = 34.8 kcal/mol) for the benzobarrelene complex **7** is significantly higher energy than for the simple barrelene analogue **4-** ***d*** (**TS2-*d***; ΔG^‡^_**4‑** ***d*** **→TS2‑** ***d***_ = 30.2 kcal/mol). In considering this difference, both reactions are driven in part by the formation of an aromatic ring. However, the additional stabilization gained by forming the second ring of naphthalene is less than for benzene. The corresponding reduced aromatic character in the transition state of **TS7-** ***p*** is expected to be less stabilizing than for **TS2-** ***d*,** accounting for the higher activation energy. Attempts to heat **7** at higher temperatures (∼170 °C in DMSO-*d*_*6*_) led to decomplexation of the barrelene instead of the desired rDA reaction.

## Conclusions

In summary, cycloadditions between tungsten-benzene complexes and electron deficient alkynes have been found to form *η* ^2^-barrelene complexes in good yield under ambient conditions. The thermodynamics and kinetics of these reactions were investigated computationally while the kinetics of these processes were also investigated experimentally. Under oxidative conditions, several novel free barrelenes were generated from their corresponding *η* ^2^-barrelene complexes and isolated. Alternatively, when these *η* ^2^-barrelene complexes were heated, the metal promoted a cycloreversion reaction through the extraction of acetylene, and the resulting substituted benzenes could be recovered in good yield. This latter reactivity pattern constitutes a rare example of a two-carbon molecular editing reaction of benzenes. This cycloreversion process has also been probed computationally and experimentally. The calculations reveal that the reaction is thermodynamically driven by the stabilization of acetylene as a result of its coordination to tungsten. These findings will allow for further exploration of barrelenes and expand the field of molecular editing.

## Supplementary Material





## Data Availability

.mnova files for all NMR spectra are located at: 10.5281/zenodo.16499365.

## Notes

^1^H and ^13^C NMR spectra of selected compounds (PDF)
Crystallographic information for compounds **4-*d***–**8-*d***, **7-*p***, **11**, **16**, **19**–**22** and **S1** (XYZ)
