# Supplementary material for: Tungsten-Enabled Diels–Alder Cycloaddition and Cycloreversion of Arenes and Alkynes: Divergent Synthesis of Highly Functionalized Barrelenes and Arenes
Source: J Am Chem Soc. 2025 Aug 8;147(33):30146–53. doi: 10.1021/jacs.5c08320 (PMC12371869; doi:10.1021/jacs.5c08320)
Supplement: Supplementary file 1 [file ja5c08320_si_001.pdf]

Supporting Information

Tungsten–enabled Diels–Alder Cycloaddition and Cycloreversion of Arenes and Alkynes: Divergent Synthesis of Highly Functionalized Barrelenes and Arenes

Jeremy M. Bloch[1‡], Evan Savelson[1‡], Alvin Q. Meng[1], Megan N. Ericson[1], Ishaan U. Patel[1], Diane A. Dickie[1], Jetze J. Tepe[1], and W. Dean Harman[1*]

[‡]J.B. and E.S. contributed equally to this paper.

[1]Department of Chemistry, University of Virginia, Charlottesville, VA 22904 U.S.A.

***Corresponding author:** wdh5z@virginia.edu

This PDF file includes:

Materials and Methods

Schemes S1 to S3

Figs. S1 to S43

Tables S1 to S7

Table of Contents

General methods — S3

Abbreviations — S4

Kinetic studies (Tables **S1–S4**, Figures **S1–S2**) — S5

Unsuccessful Diels-Alder reactions (Scheme **S1**) — S9

Experimental procedures for Diels-Alder reaction to form metal complexed barrelenes **4–8** — S10

Supplemental schemes and procedures for metal decomplexations to form barrelenes **10–14** (Schemes **S2–S3**) — S19

Experimental procedures for retro Diels–Alder reactions to form arenes **15–19** — S26

Experimental procedures for compounds **20**–**24** and **S1**–**S2** — S31

NMR spectra (Figures **S3–S28**) — S38

Crystallographic data (Tables **S5–S7**, Figures **S29–S42**) — S64

Computational details (Figure **S43**) — S82

References — S135

MATERIALS AND METHODS

Experimental Procedures & Characterizations

General Methods: IR spectra were recorded on a spectrometer as a glaze on a ZnSe anvil ATR assembly. All synthetic reactions were performed in a glovebox under a dry nitrogen atmosphere unless otherwise noted. All solvents used in DA and rDA reactions were sparged with nitrogen prior to use. Dry solvents were dried either over activated molecular sieves or by passing through an activated basic alumina column. Deuterated solvents were used as received from Cambridge Isotopes. Reagents were purchased from commercial vendors and used as received without purification. NMR spectra were recorded on 800, 600 or 400 MHz Bruker spectrometers. Proton signals were assigned via two-dimensional NMR techniques when possible. Chemical shifts are reported relative to the residue peaks of the solvent: $CDCl_3$: 7.26 ppm for ^1H and 77.23 ppm for ^{13}C, CD_3OD: 3.31 ppm for ^1H and 49.15 ppm for ^{13}C, CD_3CN: 1.94 ppm for ^1H and 1.39 ppm for ^{13}C, and DMSO-d_6: 2.50 ppm for ^1H and 39.51 ppm for ^{13}C. ^{19}F NMR spectra of selected compounds were obtained and chemical shifts are reported relative to hexafluorobenzene internal standard (δ = -164.9 ppm). Signal multiplicities are given as s (singlet), br. (broad), d (doublet), t (triplet), q (quartet) and m (multiplet). ^1H NMR chemical shift values are reported to two decimal places. ^{13}C NMR chemical shift values are reported to one decimal place unless two decimal places are needed to distinguish two similar—yet distinct—chemical shift values. Electrochemical experiments were performed under a dry nitrogen atmosphere using a potentiostat. Cyclic voltammetry data were taken at ambient temperature (20 °C) at 100 mV/s in a standard three-electrode cell with a glassy carbon working electrode using tetrabutylammonium hexafluorophosphate as an electrolyte (~0.5 M) and acetonitrile solvent unless otherwise noted. All potentials are reported versus normal hydrogen electrode (NHE) using cobaltocenium hexafluorophosphate ($E_{1/2}$ = −0.78 V) as an internal standard. The peak-to-peak separation was 100 mV or less for all reversible couples. Column chromatography was performed using a Teledyne ISCO Combi*Flash*® system or Büchi Pure C-815 Flash system with prepacked columns (RediSep Normal-phase silica or Büchi FlashPure EcoFlex silica, 40-63 microns). TLCs were performed on pre-coated 0.25 mm thick silica gel 60 F254 plates and pre-coated 150 um thick, visualized using UV light (**4–9, 15–19**), potassium permanganate staining (**10–14**, **22**), or phosphomolybdic acid staining (**23**). Compound **5** was prepared according to previous literature procedures with some modifications.[1] Compounds **1**, **2**, and **9** were prepared according to previous literature procedures. [1-3]

ABBREVIATIONS

[W]	—	{TpW(NO)(PMe_3)}
Tp	—	Hydridotris(pyrazolyl)borate
THF	—	Tetrahydrofuran
DCM	—	Dichloromethane
DME	—	1,2-dimethoxyethane
NMR	—	Nuclear magnetic resonance (spectroscopy)
TMS	—	Tetramethylsilane
IR	—	Infrared (spectroscopy)
ATR	—	Attenuated total reflectance
HRMS	—	High-resolution mass spectrometry
LRMS	—	Low-resolution mass spectrometry
ESI	—	Electrospray ionization
APCI	—	Atmospheric pressure chemical ionization
EI	—	Electron ionization
GC/MS	—	Gas chromatography/mass spectrometry
CV	—	Cyclic voltammetry
NHE	—	Normal hydrogen electrode
DA	—	Diels–Alder
rDA	—	Retro-Diels–Alder
TLC	—	Thin layer chromatography
p	—	Proximal
d	—	Distal

KINETIC STUDIES

Diels–Alder of 1 with 3a. Stock solutions of **1** in DCM-d_2 (38 mg/mL, 0.062 M), **3a** (ethyl 4,4,4-trifluorobut-2-ynoate) in DCM-d_2 (328 mg/mL, 1.98 M), and TMS in DCM-d_2 (55 mg/mL, 0.62 M) were prepared. To a test-tube charged with a stir pea was added DCM-d_2 (0.98 mL). Stirring was initiated, and TMS solution (2.0 µL) was then transferred to the test-tube, followed by the solution of **1** (10 µL) and then the solution of **3a** (10 µL). The reaction mixture was immediately transferred to an NMR tube and placed in an NMR spectrometer (800 MHz). The reaction was monitored via pseudo-two-dimensional NMR analysis (pulse program = zg2d; time between spectra = 90 seconds; relaxation delay = 3 seconds) until the reaction was observed to be complete. Two more replicates were then performed.

Normalized ^{1}H NMR integrals for **1-*p*** and **1-*d*** (δ = 5.79; signals for both isomers overlapping) were calculated relative to TMS (δ = 0.0) (Table S1). Each individual trial was then plotted as $-\ln[A]/[A]_0$ vs time and regression analysis used to calculate pseudo-first order rate constants (slope, k') (Figure S1) at which point statistical analysis was performed (n = 3) (Table S2).

Table S1. Relative concentrations of **1-*d*** + **1-*p*** ([A]/[A]$_0$) as monitored by NMR

time (s)	trial 1	trial 2	trial 3	avg	standard deviation
0	1.00	1.00	1.00	1.00	0.00
90	0.94	0.90	0.89	0.91	0.03
180	0.83	0.82	0.80	0.82	0.02
270	0.77	0.74	0.72	0.74	0.02
360	0.69	0.63	0.60	0.64	0.05
450	0.65	0.60	0.56	0.60	0.05
540	0.59	0.52	0.48	0.53	0.05
630	0.54	0.48	0.43	0.49	0.06

Figure S1. Pseudo-first order integrated rate law plots of **1**

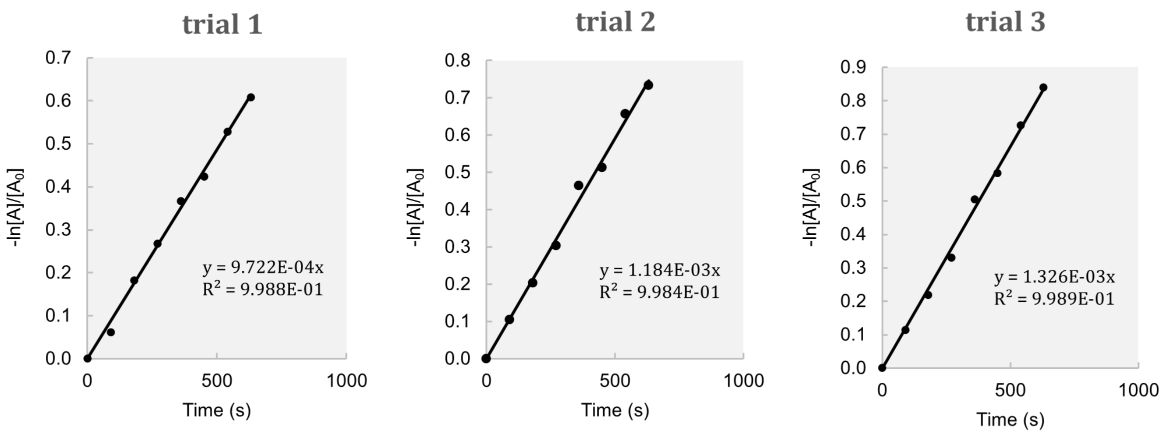

Table S2. Pseudo-first order rate constants of cycloadditions between **1** and **3a**

	trial 1	trial 2	trial 3	average	standard deviation
k' (s^{-1})	9.7E-04	1.18E-03	1.33E-03	1.16E-03	1.8E-04

Retro Diels–Alder of 4-*p* and 4-*d*. To a solution of **4** (117 mg, 0.150 mmol, mixture of stereoisomers) in 15 mL CD_3CN in a 20 mL scintillation vial was added an arbitrary amount of dimethylsulfone (~10 mg) as an internal standard. The solution was stirred until homogenous before being divided into three scintillation vials each with 5 mL of solution and a stir bar and sealed with electrical tape. The sealed vials were then heated with stirring in aluminum blocks at 70 °C. At specified time points, the vials were opened, and a 0.5 mL aliquot was removed for ^1H NMR analysis (400 MHz) before returning the aliquot to the original vial.

Normalized ^1H NMR integrals for **4-*p*** (δ = 3.59) and **4-*d*** (δ = 3.49) were calculated relative to dimethyl sulfone (δ = 2.91) (Table S3). Each individual trial was then plotted as $-\ln[A]/[A]_0$ vs time and regression analysis used to calculate first order rate constants (slope, k) (Figure S2). These rate constants were then used to find activation energies ($\Delta G^{\ddagger}$) via the Eyring equation at which point statistical analysis was performed (n = 3) (Table S4).

Table S3. Relative concentrations of **4-*d*** and **4-*p*** ($[A]/[A]_0$) at variable reaction temperatures as monitored by NMR

	**5.4-*d***					**5.4-*p***				
time (s)	trial 1	trial 2	trial 3	avg	standard deviation	trial 1	trial 2	trial 3	avg	standard deviation
0	1.00	1.00	1.00	1.00	0.00	1.00	1.00	1.00	1.00	0.00
55800	0.60	0.56	0.63	0.60	0.03	0.54	0.68	0.61	0.61	0.07
82800	0.49	0.48	0.47	0.48	0.01	0.52	0.58	0.49	0.53	0.05
136800	0.33	0.31	0.33	0.33	0.01	0.35	0.40	0.37	0.38	0.02
158400	0.27	0.25	0.26	0.26	0.01	0.30	0.33	0.32	0.31	0.01
216000	0.17	0.15	0.15	0.16	0.01	0.20	0.21	0.21	0.21	0.01

Figure S2. First order integrated rate law plots of **4-*d*** and **4-*p***

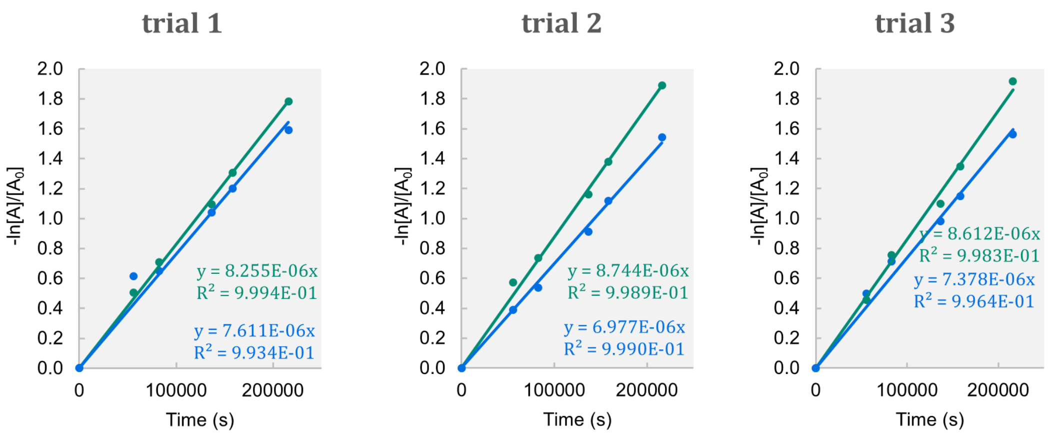

Table S4. First order rate constants and activation energies of cycloreversions of **4-*d*** and **4-*p***

	**4-*d***					**4-*p***				
	trial 1	trial 2	trial 3	avg	standard deviation	trial 1	trial 2	trial 3	avg	standard deviation
k (s^{-1})	8.26E-06	8.74E-06	8.61E-06	8.54E-06	2.0E-07	7.61E-06	6.98E-06	7.38E-06	7.32E-06	4.1E-07
$\Delta G^{\ddagger}$ (kcal/mol)	28.1	28.1	28.1	28.1	0.00	28.2	28.3	28.2	28.2	0.0

Scheme S1. Unsuccessful DA reactions

[W]-Arenes

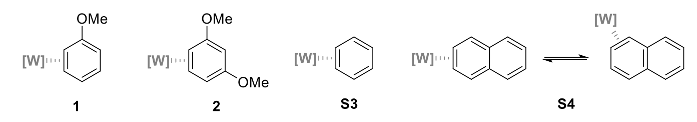

Dienophiles

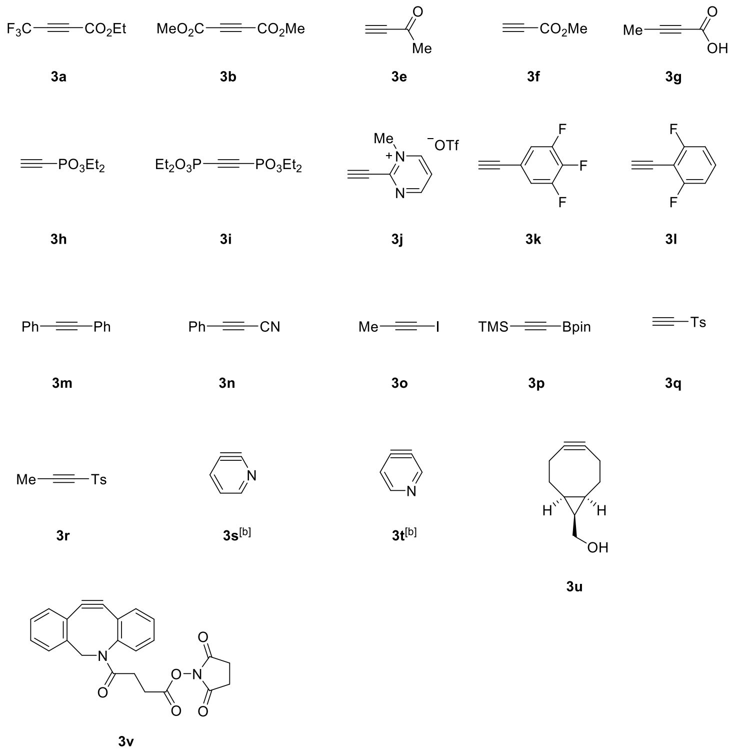

Unsuccessful DA Reactions

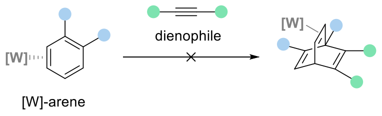

entry	[W]-arene	dienophile	solvent
1	S3	3a, 3b, 3e	THF-d_8
2	S4	3a, 3b	THF-d_8
3	1	3e	THF-d_8
4	1	3f	DCM-d_2, THF-d_8, neat
5	1	3g	THF-d_8
6	1	3h	THF-d_8
7	1	3i	THF-d_8
8	1	3j	THF-d_8
9	1	3k	THF-d_8
10	1	3l	THF-d_8
11	1	3m	DCM-d_2, CD_3OD
12	1	3n	THF-d_8
13	1	3o	THF-d_8
14	1	3p	DCM-d_2
15[a]	1	3q	THF-d_8, tol
16	1, 2	3r	THF-d_8
17	1	3s	THF-d_8
18	1	3t	THF-d_8
19	1	3u	THF-d_8
20	1	3v	THF-d_8

[a] **S1** isolated, 9% yield. [b] Prepared *in-situ* from respective *o*-trimethylsilyl triflate precursors. Lists of reagents represent separate experiments.

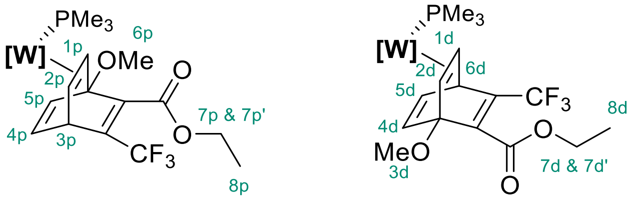

4-*p* : **4-*d***
1 : 1.3

Proximal and distal TpW(NO)(PMe₃)(5,6-η^2-ethyl 1-methoxy-3-(trifluoromethyl)bicyclo[2.2.2]octa-2,5,7-triene-2-carboxylate) (4). To a medium test-tube charged with a stir-pea was added **1** (500 mg, 0.818 mmol) and dry THF (~1 mL). Stirring was initiated followed by the addition of neat ethyl 4,4,4-trifluorobut-2-ynoate (300 mg, 0.26 mL, 1.8 mmol). The reaction was allowed to stir for 1.5 hours at which point the reaction mixture was added to stirring pentane (~100 mL), resulting in a brown precipitate. The test tube was washed with DCM (~1 mL) which was also added to the stirring pentane. The precipitate was then collected on a 15 mL fine porosity fritted disc and desiccated under static vacuum, providing brown powder **4** as a mixture of isomers (1:1.3 proximal:distal) (524 mg, 0.674 mmol, 82% yield). **^{1}H NMR (800 MHz, CD_3CN) δ:** 8.44 (d, J = 2.1 Hz, 1H, Tp), 8.07 (m, 2H, partially overlapping Tp)*, 7.86 (d, J = 2.1 Hz, 1H, Tp), 7.85 (m, 2H, partially overlapping Tp)*, 7.83 (d, J = 2.3 Hz, 1H, Tp), 7.81 (d, J = 2.3 Hz, 1H, Tp), 7.69 (d, J = 2.4 Hz, 1H, Tp), 7.65 (d, J = 2.4 Hz, 1H, Tp), 7.49 (d, J = 2.2 Hz, 1H, Tp), 7.36 (d, J = 2.2 Hz, 1H, Tp), 6.64 (d, J = 7.6 Hz, 1H, H4d), 6.51 (d, J = 7.7 Hz, 1H, H5p), 6.46 (dd, J = 7.8, 6.1 Hz, 1H, H4p), 6.37 (m, 2H, partially overlapping Tp)*, 6.28 (t, J = 2.2 Hz, 1H, Tp), 6.26 (t, J = 2.2 Hz, 1H, Tp), 6.25 (t, J = 2.2 Hz, 1H, Tp), 6.20 (m, 2H, Tp overlapping with H5d)*, 4.31 (m, 3H, H6d overlapping with H7p and H7p')*, 4.21 (dd, J = 7.1, 4.5 Hz, 2H, H7d overlapping with H7d')*, 4.18 (m, 1H, H3p), 3.59 (s, 3H, H6p), 3.49 (s, 3H, H3d), 3.07 (t, J = 11.0 Hz, 1H, H1p), 2.87 (ddd, J = 12.7, 9.9, 3.3 Hz, 1H, H1d), 1.90 (dd, J = 10.0, 3.0 Hz, 1H, H2d), 1.65 (dt, J = 9.9, 3.2 Hz, 1H, H2p), 1.32 (t, J = 7.2 Hz, 3H, H8p), 1.28 (d, J = 9.0 Hz, 9H, proximal PMe_3), 1.23 (t, J = 7.1 Hz, 3H, H8d), 1.20 (d, J = 8.7 Hz, 9H, distal PMe_3). **^{13}C NMR (201 MHz, CD_3CN) δ:** 167.72, 167.68, 154.5 (q, J_{CF} = 4.4 Hz), 153.2 (q, J_{CF} = 4.8 Hz), 148.8, 145.4, 144.9, 144.7, 143.0, 142.7, 140.2 (q, J_{CF} = 31.6 Hz), 139.2 (q, J_{CF} = 32.3 Hz), 138.2, 138.1, 137.5, 137.4, 136.7, 136.3, 136.2, 133.8, 133.2, 131.7, 125.0,** 125.0,** 107.6,*** 107.6,*** 107.2, 107.10, 107.08, 106.5, 99.0, 98.7, 67.6, 66.1, 65.6, 63.7, 62.22, 62.20, 56.0, 55.6, 47.2, 45.8, 14.5, 14.4***, 14.4*** (d, J_{CP} = 29.4 Hz), 13.9 (d, J_{CP} = 29.4 Hz). **^{19}F NMR (565 MHz, CD_2Cl_2) δ:** -66.4 (s, CF_3), -66.5 (s, CF_3) ppm. **CV** (MeCN; 100 mV/s): $E_{p,a}$ = +0.90 V (NHE). Composition confirmed by single-crystal X-ray diffraction.

*Overlapping proton signals as indicated by HSQC.
**Two partially overlapping (interlaced) carbon quartets.
***HSQC data indicate two overlapping carbons.

^{1}H NMR impurity peaks δ: 3.42 (Et_2O), 2.09 (acetone), 1.28 (pentane), 1.12 (Et_2O), and 0.89 (pentane) ppm.

^{13}C NMR impurity peaks δ: 207.5 (acetone), 130.0 (unidentified), 129.3 (unidentified), 126.3 (unidentified), 66.4 (Et_2O), 34.9 (pentane), 31.0 (acetone), 23.1 (pentane), 15.7 (Et_2O), and 14.4 (pentane) ppm.

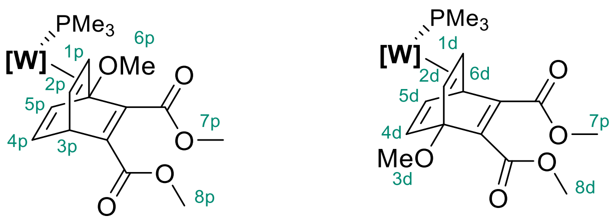

5-*p* : **5-*d***

1 : 2.8

Improved procedure for the synthesis of **proximal and distal TpW(NO)(PMe_3)(5,6-η^2-dimethyl 1-methoxybicyclo[2.2.2]octa-2,5,7-triene-2,3-dicarboxylate) (5).** To a medium test-tube charged with a stir-pea was added **1** (503 mg, 0.823 mmol) and dry THF (~4 mL). Stirring was initiated followed by the addition of neat dimethyl acetylenedicarboxylate (0.30 mL, 2.4 mmol). The reaction was allowed to stir for 1 h at which point the reaction mixture was added to stirring pentane (~100 mL), resulting in a tan-brown precipitate. The precipitate was then collected on a 15 mL fine porosity fritted disc, washed with pentane (~15 mL), and desiccated under static vacuum, providing tan-brown powder **5** as a mixture of isomers (1:2.8 proximal:distal) (442 mg, 0.587 mmol, 71% yield). Composition confirmed by single-crystal X-ray diffraction. **^{1}H NMR (800 MHz, CD_2Cl_2) δ:** 8.45 (d, J = 2.1 Hz, 1H, Tp), 8.07 (d, J = 2.0 Hz, 1H, Tp), 8.06 (d, J = 2.0 Hz, 1H, Tp), 7.85 (d, J = 2.0 Hz, 1H, Tp), 7.75 (d, J = 2.3 Hz, 2H, two overlapping Tp)*, 7.71 (d, J = 2.3 Hz, 1H, Tp), 7.70 (d, J = 2.3 Hz, 1H, Tp), 7.60 (d, J = 2.4 Hz, 1H, Tp), 7.56 (d, J = 2.4 Hz, 1H, Tp), 7.41 (d, J = 2.2 Hz, 1H, Tp), 7.33 (d, J = 2.2 Hz, 1H, Tp), 6.67 (d, J = 7.6 Hz, 1H, H4d), 6.51 (m, 2H, H4p and H5p)*, 6.33 (t, J = 2.1 Hz, 2H, two overlapping Tp)*, 6.26 (t, J = 2.2 Hz, 1H, Tp), 6.24 (dd, J = 7.7, 6.1 Hz, 1H, H5d), 6.20 (t, J = 2.2 Hz, 1H, Tp), 6.19 (t, J = 2.2 Hz, 1H, Tp), 6.18 (t, J = 2.2 Hz, 1H, Tp), 4.60 (m, 1H, H6d), 4.48 (m, 1H, H3p), 3.83 (s, 3H, H7p or H8p), 3.76 (s, 3H, H7d), 3.74 (s, 3H, H8d), 3.64 (s, 3H, H7p or H8p), 3.62 (s, 3H, H6p), 3.55 (s, 3H, H3d), 3.07 (dd, J = 12.2, 9.7 Hz, 1H, H1p), 2.88 (ddd, J = 12.9, 9.9, 3.4 Hz, 1H, H1d), 2.05 (dd, J = 9.9, 3.2 Hz, 1H, H2d), 1.82 (m, 1H, H2p), 1.31 (d, J = 8.9 Hz, 9H, minor PMe_3), 1.25 (d, J = 8.4 Hz, 9H, minor PMe_3). **^{13}C NMR (201 MHz, CD_2Cl_2) δ:** 169.22, 169.18, 165.31, 165.27, 159.8, 158.1, 152.7, 148.3, 144.7, 144.0, 143.9, 142.11, 142.06, 141.9, 137.3, 137.1, 136.53, 136.49, 135.30, 135.27, 135.25, 133.7, 132.5, 131.9, 106.8**, 106.4, 106.2, 106.1, 105.7, 98.7, 98.4, 67.9 (d, J_{CP} = 2.9 Hz), 66.5 (d, J_{CP} = 17.9 Hz), 65.4 (d, J_{CP} = 17.4 Hz), 63.8 (d, J_{CP} = 2.2 Hz), 55.7, 55.5, 52.43, 52.38, 52.3, 52.2, 47.7, 46.3, 14.6 (d, J_{CP} = 29.2 Hz), 14.2 (d, J_{CP} = 28.5 Hz).

*Overlapping proton signals as indicated by HSQC.

**HSQC data indicate two overlapping carbons.

Other characterization of **5** has previously been reported.[1]

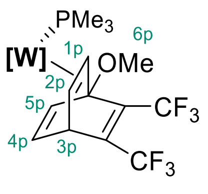 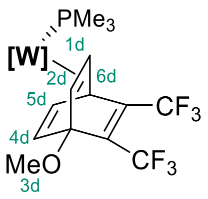

6-*p* 1 : **6-*d*** 4

Proximal and distal

TpW(NO)(PMe$_3$)(5,6-η^2-1-methoxy-2,3-bis(trifluoromethyl)bicyclo[2.2.2]octa-2,5,7-triene) (6). To a flame-dried 250 mL round-bottom flask with a stir egg was added **1** (595 mg, 0.974 mmol). Dry THF (~5 mL) was then added, and the round-bottom flask was sealed with a rubber septum. Stirring was initiated. A balloon was then attached to one end of a 1 mL plastic syringe with a needle placed at the other end. This needle was inserted through the septum. A separate needle was then inserted through the septum and into the stirring solution of **1**. Through this second needle was dispensed 1,1,1,4,4,4-hexafluorobut-2-yne gas (~550 cc, ~23 mmol). The second needle was then removed and the reaction allowed to proceed for an hour at room temperature. The reaction mixture was removed from the round-bottom flask, transferred to a 125 mL filter flask, and concentrated to a brown oil *in vacuo*. The oil was redissolved in DCM (minimal) which was then added to stirring pentane (~250 mL), resulting in a brown precipitate. The precipitate was then collected on a 60 mL fine porosity fritted disc, washed with pentane (~15 mL), and desiccated under static vacuum. The filtrate was concentrated *in vacuo* and desiccated under static vacuum. The precipitate and filtrate were redissolved in EtOAc, combined, and dry-loaded onto Celite®. The complex was then purified using a Combi*Flash*® (12-gram silica gel column; 100:0 → 50:50 hexanes/EtOAc; 18 minutes). Fractions containing the product were combined, concentrated to a solid *in vacuo*, and dried under high vacuum for ~1 hour, providing cream-colored **6** as a mixture of isomers (1:4 proximal:distal) (450 mg, 0.58 mmol, 60% yield). **^{1}H NMR (800 MHz, CD$_3$CN) δ:** 8.42 (d, J = 1.8 Hz, 1H, Tp), 8.08 (d, J = 1.9 Hz, 1H, Tp), 8.07 (d, J = 1.9 Hz, 1H, Tp), 7.86 (m, 2H, partially overlapping Tp)*, 7.84 (d, J = 1.8 Hz, 1H, Tp), 7.83 (d, J = 2.2 Hz, 1H, Tp), 7.81 (d, J = 2.3 Hz, 1H, Tp), 7.69 (d, J = 2.4 Hz, 1H, Tp), 7.65 (d, J = 2.4 Hz, 1H, Tp), 7.51 (d, J = 2.0 Hz, 1H, Tp), 7.38 (d, J = 2.0 Hz, 1H, Tp), 6.72 (dt, J = 7.6, 1.3 Hz, 1H, H4d), 6.57 (dt, J = 7.6, 1.3 Hz, 1H, H5p), 6.48 (t, J = 7.0 Hz, 1H, H4p), 6.38 (t, J = 2.2 Hz, 1H, Tp), 6.37 (t, J = 2.2 Hz, 1H, Tp), 6.28 (t, J = 2.2 Hz, 1H, Tp), 6.26 (t, J = 2.3 Hz, 1H, Tp), 6.24 (t, J = 2.3 Hz, 1H, Tp), 6.22 (m, 2H, Tp overlapping with H5d)*, 4.52 (m, 1H, H6d), 4.40 (t, J = 4.7 Hz, 1H, H3p), 3.65 (s, 3H, H6p), 3.58 (s, 3H, H3d), 3.05 (t, J = 11.0 Hz, 1H, H1p), 2.85 (ddd, J = 12.6, 10.0, 3.3 Hz, 1H, H1d), 1.88 (dd, J = 9.6, 2.9 Hz, 1H, H2d), 1.66 (dt, J = 9.9, 3.3 Hz, 1H, H2p), 1.27 (d, J = 9.1 Hz, 9H, proximal PMe$_3$), 1.19 (d, J = 8.7 Hz, 9H, distal PMe$_3$). **^{13}C NMR (201 MHz, CD$_3$CN) δ:** 148.7, 148.5 (q, J_{CF} = 28.3 Hz), 146.4 (q, J_{CF} = 30.4 Hz), 145.5, 144.7, 144.6, 143.1, 142.8, 138.3, 138.1, 137.54, 137.49, 136.40, 136.36, 136.3, 134.0, 132.3, 131.1, 124.2 (q, J_{CF} = ~271 Hz)**, 107.7, 107.6, 107.3, 107.1***, 106.5, 98.8, 98.6, 67.0 (d, J_{CP} = 3.3 Hz), 66.0 (d, J_{CP} = 17.5 Hz), 65.3 (d, J_{CP} = 18.5 Hz), 64.5 (d, J_{CP} = 2.2 Hz), 56.5 (q, J_{CF} = 3.3 Hz), 56.2 (q, J_{CF} = 3.3 Hz), 48.8 (d, J_{CP} = 2.8 Hz), 47.5, 14.3 (d, J_{CP} = 29.4 Hz),

13.8 (d, J_{CP} = 29.4 Hz). **CV** (MeCN; 100 mV/s): $E_{p,a}$ = +0.56 V (NHE). Composition confirmed by single-crystal X-ray diffraction.

^{1}H NMR impurity peaks δ: 2.14 (s, H_2O) ppm.

*Overlapping proton signals as indicated by HSQC.
**Two partially overlapping (interlaced) carbon quartets
***HSQC data indicate two overlapping carbons.

Note: due to weak signals in the 1D ^{13}C NMR spectrum as well as in the HSQC and HMBC, the two CF_3 group carbons and the two alkene quaternary carbons of the minor isomer could not be located.

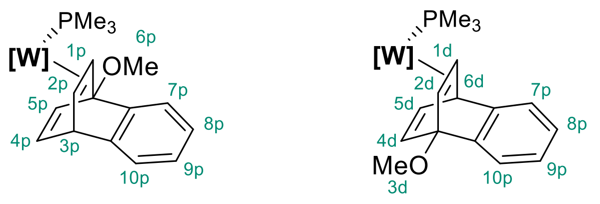

7-*p* 3 : **7-*d*** 1

Proximal and distal

TpW(NO)(PMe$_3$)(2,3-η^2-1-methoxy-1,4-dihydro-1,4-ethenonaphthalene) (7). To a flame-dried medium test-tube charged with a stir-pea was added **1** (665 mg, 1.09 mmol), dry THF (~2 mL), and 2-(trimethylsilyl)phenyl trifluoromethanesulfonate (0.40 mL, 1.6 mmol). Stirring was initiated and tetrabutylammonium fluoride (2.72 mL, 2.72 mmol, 1M solution in THF) was added dropwise, after which the reaction was allowed to stir for 1 h. The reaction mixture was added to pentane (~100 mL) and this solution was transferred to a 250 mL round-bottom flask. Solvent was then removed *in vacuo*. The resulting crude mixture was redissolved in acetone, dry-loaded onto silica gel, and purified using a Combi*Flash*® (12-gram silica gel column; 100:0 → 0:100 hexanes/EtOAc; 20 minutes). Fractions containing the desired isomeric mixture of complexes were combined, concentrated to a solid via rotary evaporation, and desiccated under static vacuum, providing tan solid **7** as a mixture of isomers (3:1 proximal:distal) (259 mg, 0.377 mmol, 35% yield). **^{1}H NMR (800 MHz, CD$_3$CN) δ:** 8.71 (d, J = 1.8 Hz, 1H, Tp), 8.10 (d, J = 1.8 Hz, 1H, Tp), 8.09 (d, J = 1.9 Hz, 1H, Tp), 8.01 (d, J = 1.7 Hz, 1H, Tp), 7.85 (m, 2H, two overlapping Tps)*, 7.78 (d, J = 2.2 Hz, 1H, Tp), 7.76 (d, J = 2.2 Hz, 1H, Tp), 7.69 (d, J = 2.4 Hz, 1H, Tp), 7.65 (d, J = 2.4 Hz, 1H, Tp), 7.32 (m, 2H, Tp overlapping with H7p)*, 7.20 (m, 3H, Tp overlapping with H7d and H10d)*, 7.02 (d, J = 7.1 Hz, 1H, H10p), 6.97 (td, J = 7.4, 1.1 Hz, 1H, H8p), 6.93 (m, 2H, H8d and H9d overlapping)*, 6.86 (td, J = 7.4, 1.1 Hz, 1H, H9p), 6.59 (dt, J = 8.0, 1.2 Hz, 1H, H4d), 6.51 (dt, J = 8.1, 1.2 Hz, 1H, H5p), 6.48 (dd, J = 7.9, 6.1 Hz, 1H, H4p), 6.37 (m, 2H, two overlapping Tps)*, 6.31 (t, J = 2.1 Hz, 1H, Tp), 6.24 (t, J = 2.1 Hz, 1H, Tp), 6.22 (dd, J = 7.9, 6.2 Hz, 1H, 5d), 6.16 (t, J = 2.2 Hz, 1H, Tp), 6.13 (t, J = 2.2 Hz, 1H, Tp), 4.41 (ddd, J = 6.0, 3.2, 1.3 Hz, 1H, H6d), 4.29 (t, J = 4.6 Hz, 1H, H3p), 3.75 (s, 3H, H6p), 3.63 (s, 3H, H3d), 2.70 (m, 2H, H1p and H1d overlapping)*, 1.61 (ddd, J = 9.9, 3.1, 1.0 Hz, 1H, H2d), 1.40 (dt, J = 9.9, 3.1 Hz, H2p), 1.34 (d, J = 9.0 Hz, proximal PMe$_3$), 1.26 (d, J = 8.5 Hz, distal PMe$_3$). **^{13}C NMR (201 MHz, CD$_3$CN) δ:** 152.3, 151.5**, 151.3, 149.0, 145.4, 144.8, 144.7, 142.8, 142.6, 138.0, 137.8, 137.3, 137.2**, 136.2, 136.1, 134.02, 133.98, 132.6, 124.5, 124.4, 124.2, 124.1, 122.7, 122.6, 119.6, 119.4, 107.4**, 107.1, 106.9, 106.8, 106.4, 95.4, 94.9, 66.7, 65.6 (d, J_{CP} = 16.4 Hz), 65.5 (d, J_{CP} = 17.4 Hz), 63.0, 55.1, 54.6, 50.6, 49.0, 14.7 (d, J_{CP} = 29.4 Hz), 14.1 (d, J_{CP} = 28.5 Hz). **CV** (MeCN; 100 mV/s): $E_{p,a}$ = +0.76 V (NHE). Composition of both proximal and distal isomers confirmed by single-crystal X-ray diffraction.

*Overlapping proton signals as indicated by HSQC.

**Two overlapping carbon signals as indicated by HSQC.

NOTE: The proximal and distal isomers of **7** were found to have different enough retention times that separation was achieved during the purification step described above in the synthetic procedure for **7**. NMR data for the separate isomers are reported below:

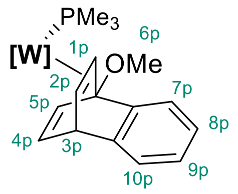

7-*p*

Proximal TpW(NO)(PMe$_3$)(2,3-η^2-1-methoxy-1,4-dihydro-1,4-ethenonaphthalene) (7-*p*). ^{1}H NMR (800 MHz, CD$_3$CN) δ: 8.08 (d, *J* = 2.0 Hz, 1H, Tp), 8.01 (d, *J* = 2.0 Hz, 1H, Tp), 7.85 (d, *J* = 2.4 Hz, 1H, Tp), 7.76 (d, *J* = 2.2 Hz, 1H, Tp), 7.70 (d, *J* = 2.4 Hz, 1H, Tp), 7.32 (d, *J* = 7.4 Hz, 1H, H7p), 7.21 (d, *J* = 2.2 Hz, 1H, Tp), 7.02 (d, *J* = 1.2 Hz, 1H, H10p), 6.97 (td, *J* = 7.4, 1.2 Hz, 1H, H8p), 6.87 (td, *J* = 7.3, 1.2 Hz, 1H, H9p), 6.51 (dt, *J* = 8.0, 1.4 Hz, 1H, H5p), 6.47 (dd, *J* = 8.1, 6.0 Hz, 1H, H4p), 6.37 (t, *J* = 2.2 Hz, 1H, Tp), 6.31 (t, *J* = 2.2 Hz, 1H, Tp), 6.13 (t, *J* = 2.2 Hz, 1H, Tp), 4.29 (t, *J* = 4.9 Hz, 1H, H3p), 3.75 (s, 3H, H6p), 2.70 (dd, *J* = 11.9, 10.5 Hz, 1H, H1p), 1.40 (dt, *J* = 9.9, 3.3 Hz, 1H, H2p), 1.34 (d, *J* = 9.0 Hz, 9H, proximal PMe$_3$). **^{13}C NMR (201 MHz, CD$_3$CN) δ:** 151.5, 145.4, 144.82, 144.81, 142.6, 137.8, 137.3, 136.2, 134.02, 133.98, 124.5, 124.1, 122.6, 119.4, 107.4, 107.1, 106.8, 95.4, 66.7, 65.6 (d, J_{CP} = 16.4 Hz), 55.1, 49.0, 14.7 (d, J_{CP} = 29.4 Hz).

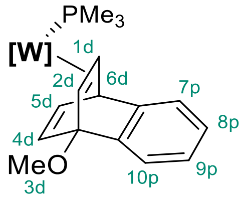

7-*d*

Distal TpW(NO)(PMe$_3$)(2,3-η^2-1-methoxy-1,4-dihydro-1,4-ethenonaphthalene) (7-*d*). ^{1}H NMR (800 MHz, CD$_3$CN) δ: 8.71 (d, *J* = 2.0 Hz, 1H, Tp), 8.10 (d, *J* = 2.0 Hz, 1H, Tp), 7.85 (d, *J* = 2.4 Hz, 1H, Tp), 7.78 (d, *J* = 2.3 Hz, 1H, Tp), 7.65 (d, *J* = 2.4 Hz, 1H, Tp), 7.33 (d, *J* = 2.2 Hz, 1H, Tp), 7.20 (m, 2H, H7d and H10d), 6.93 (m, 2H, H8d and H9d), 6.59 (dt, *J* = 8.0, 1.4 Hz, 1H, H4d), 6.37 (t, *J* = 2.2 Hz, 1H, Tp), 6.24 (t, *J* = 2.2 Hz, 1H, Tp), 6.22 (dd, *J* = 8.0, 6.1 Hz, 1H, H5d), 6.16 (t, *J* = 2.3 Hz, 1H, Tp), 4.41 (ddd, *J* = 6.2, 3.4, 1.4 Hz, 1H, H6d), 3.63 (s, 3H, H3d), 2.71 (ddd, *J* = 12.9, 9.9, 3.3 Hz, 1H, H1d), 1.61 (ddd, *J* = 9.9, 3.1, 1.2 Hz, 1H, H2d), 1.26 (d, *J* = 8.6 Hz, 9H, distal PMe$_3$). **^{13}C NMR (201 MHz, CD$_3$CN) δ:** 152.8, 151.8, 149.6, 145.2, 143.3, 138.4, 137.8, 137.7, 136.6, 133.2, 125.0, 124.7, 123.2, 120.1, 107.9, 107.4, 107.0, 95.4, 66.0 (d, J_{CP} = 17.4 Hz), 63.6, 55.1, 51.1, 14.7 (d, J_{CP} = 28.4 Hz).

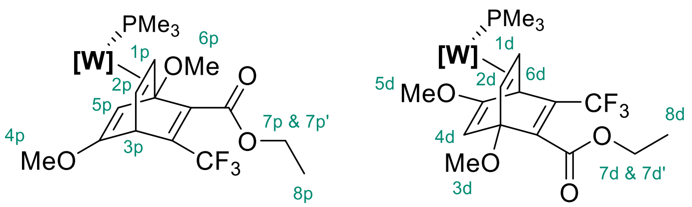

8-*p* : **8-*d***
2 : 1

Proximal and distal TpW(NO)(PMe$_3$)(5,6-η^2-ethyl 1,5-dimethoxy-3-(trifluoromethyl)bicyclo[2.2.2]octa-2,5,7-triene-2-carboxylate) (8). To a medium test-tube charged with a stir-pea was added **2** (438 mg, 0.683 mmol) and dry THF (~1.5 mL). Stirring was initiated followed by the addition of neat ethyl 4,4,4-trifluorobut-2-ynoate (0.22 mL, 1.5 mmol). The reaction was allowed to stir for 1.5 hours at which point the reaction mixture was added to stirring pentane (~125 mL), resulting in a tan precipitate. The precipitate was then collected on a 15 mL fine porosity fritted disc, washed with pentane (~15 mL), and desiccated under static vacuum, providing tan powder **8** as a mixture of isomers (2:1 proximal:distal) (390 mg, 0.483 mmol, 71% yield). **^{1}H NMR (800 MHz, CD$_3$CN) δ:** 8.56 (d, J = 1.8 Hz, 1H, Tp), 8.10 (d, J = 1.8 Hz, 1H, Tp), 8.09 (d, J = 1.8 Hz, 1H, Tp), 8.03 (d, J = 1.7 Hz, 1H, Tp), 7.86 (m, 2H, two overlapping Tps)*, 7.83 (d, J = 2.3 Hz, 1H, Tp), 7.82 (d, J = 2.2 Hz, 1H, Tp), 7.70 (d, J = 2.4 Hz, 1H, Tp), 7.65 (d, J = 2.3 Hz, 1H, Tp), 7.51 (d, J = 2.1 Hz, 1H, Tp), 7.31 (d, J = 2.0 Hz, 1H, Tp), 6.37 (t, J = 2.2 Hz, 2H, two overlapping Tps)*, 6.29 (t, J = 2.2 Hz, 1H, Tp), 6.26 (m, 2H, two overlapping Tps)*, 6.21 (t, J = 2.2 Hz, 1H, Tp), 5.39 (d, J = 2.5 Hz, 1H, H4d), 5.23 (d, J = 2.2 Hz, 1H, H5p), 4.33 (m, 2H, H7d and H7d' overlapping)*, 4.23 (m, 2H, H7p and H7p' overlapping)*, 3.88 (t, J = 3.0 Hz, 1H, H6d), 3.74 (s, 3H, H4p), 3.71 (m, 1H, H3p), 3.68 (s, 3H, H5d), 3.56 (s, 3H, H6p), 3.52 (s, 3H, H3d), 3.18 (dd, J = 13.0, 10.2 Hz, 1H, H1p), 2.78 (td, J = 11.0, 3.5 Hz, 1H, H1d), 1.97 (dd, J = 10.3, 3.3 Hz, 1H, H2d), 1.56 (ddd, J = 10.3, 3.8, 2.6 Hz, 1H, H2p), 1.32 (t, J = 7.1 Hz, 3H, H8p), 1.29 (d, ^2J = 9.1 Hz, 9H, proximal PMe$_3$), 1.24 (t, J = 7.1 Hz, 3H, H8d), 1.22 (d, ^2J = 8.7 Hz, 9H, distal PMe$_3$). **^{13}C NMR (201 MHz, CD$_3$CN) δ:** 167.72, 167.69, 163.3, 161.9, 156.0 (q, J_{CF} = 4.0 Hz), 149.0, 145.5, 144.42, 144.41, 144.35, 144.34, 142.9, 142.5, 138.7 (q, J_{CF} = 32.2 Hz), 138.3, 138.0, 137.5, 137.4, 136.3, 136.2, 125.0 (q, $^1J_{CF}$ = 269.4 Hz), 107.6, 107.5, 107.3, 107.2, 107.1, 106.5, 100.0, 99.1, 99.0, 98.0, 68.8, 67.5 (d, $^2J_{CP}$ = 18.5 Hz), 67.3 (d, $^2J_{CP}$ = 17.5 Hz), 66.3, 62.20, 62.17, 57.2, 57.1, 55.6, 55.5, 48.8, 47.4, 14.5, 14.4, 14.1 (d, $^1J_{CP}$ = 29.4 Hz), 13.7 (d, $^1J_{CP}$ = 29.4 Hz). **APCI-HRMS** (m/z): [M+H]+ calculated for [$C_{25}H_{33}BF_3N_7O_5PW$]+: 794.1830; found: 794.1830. **CV** (MeCN; 100 mV/s): $E_{p,a}$ = +0.87 V (NHE). Composition confirmed by single-crystal X-ray diffraction.**

Note: due to poor signal to noise ratio in the ^{13}C NMR, the carbon of the CF_3 group of the minor isomer could not be located.

*Two overlapping proton signals as indicated by HSQC.

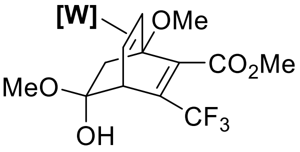

8a

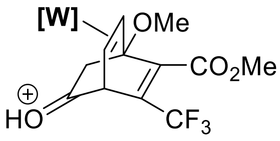

8b

The product (8**) co-crystallized out with **TpW(NO)(PMe3)(5,6-η^2-ethyl (1R,4R,8S)-8-hydroxy-1,8-dimethoxy-3-(trifluoromethyl)bicyclo[2.2.2]octa-2,5-diene-2-carboxylate) (8a),** formed from the hydration of the enol ether (-OH added to the same carbon as -OMe). This is supported by HRMS data which showed the presence of a cationic fragment [**8a**-OMe]+ (**8b**).

Scheme S2. Screening of metal decomplexation condition on **4**

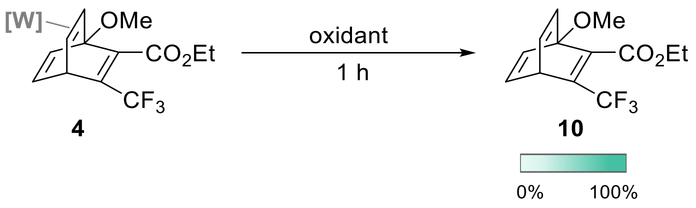

entry[a]	oxidant	solvent	temperature	Yield[b]
1	CAN	THF	0 °C	36%
2	$AgNO_3$	MeCN	0 °C	0%[c]
3	$FeCp_2PF_6$	DCM	0 °C	68%
4	DDQ	MeCN	0 °C	49%
5	DDQ	THF	0 °C	68%
6	DDQ	THF	-12 °C	79%
7	DDQ	THF	-45 °C	88%
8[d]	DDQ	THF	-45 °C	95%
9[d][e]	DDQ	THF	-45 °C	72%[f]

[a] 0.05 mmol scale, 0.05 M. 2.2 equiv. oxidant. [b] Yields determined by crude NMR. Internal standard = dimethyl sulfone. [c] No reaction. [d] 3.3 equiv. oxidant. [e] 0.3 mmol scale. [f] Isolated yield.

Scheme S3. NMR yields of free barrelenes

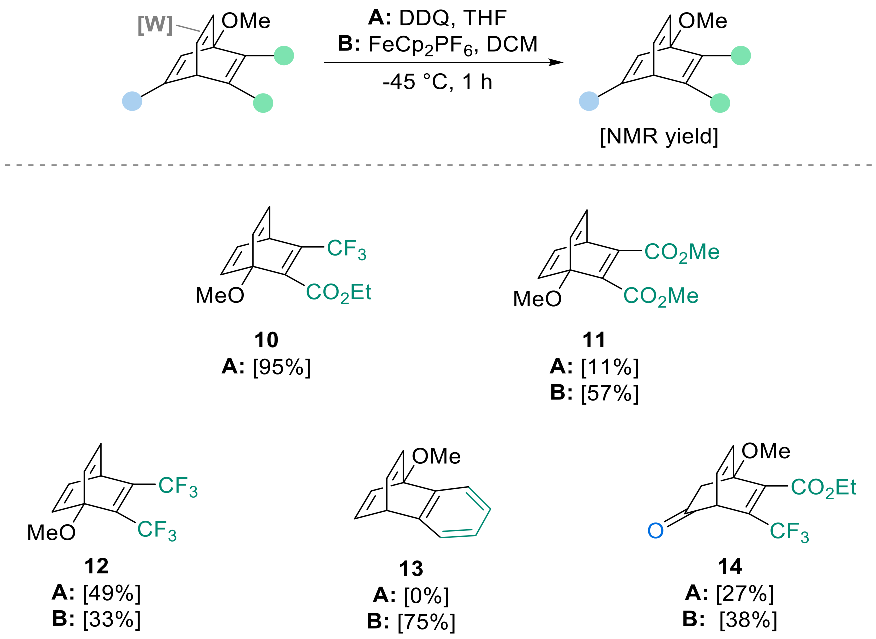

Barrelene complex oxidant screening procedure: To a 1-dram vial was added barrelene complex (0.050 mmol, 1.0 equiv) and solvent (2 mL). The solution was cooled to a specified temperature, then oxidant (2.2-3.3 equiv) was added, and the reaction was stirred for 1 hour while returning to room temperature. The solution was concentrated and re dissolved in $CDCl_3$ (3 mL) then decanted and syringe filtered to remove insoluble residue. Dimethylsulfone (~5 mg) was then added to the solution for quantitative ^1H NMR analysis.

Barrelene complex oxidation general procedure A: To a 25 mL round-bottom flask was added barrelene complex (0.150 mmol, 1 equiv) and THF (6 mL) and cooled to -45 °C in a MeCN/CO_2 bath. To this solution was added DDQ (112 mg, 0.495 mmol, 3.3 equiv) and stirred for 1 hour while returning to room temperature. The solution was quenched with 5 mL sat. Na_2CO_3 solution then diluted with water (10 mL) and extracted with DCM (3 x 10 mL). The combined organic layers were dried with Na_2SO_4 and concentrated *in vacuo* before being filtered through a Celite® plug and washed with DCM (20 mL), then subjected to purification using a Pure C-815 system (12-gram silica gel column, 100:0 → 20:80 hexanes/EtOAc; 20 minutes).

Barrelene complex oxidation general procedure B: To a 25 mL round-bottom flask was added barrelene complex (0.150 mmol, 1.0 equiv) and DCM (6 mL) and cooled to -45 °C in a MeCN/CO_2 bath. To this solution was added $FeCp_2PF_6$ (167 mg, 0.495 mmol, 3.3 equiv) and stirred for 1 hour while returning to room temperature. The solution was diluted with water (10 mL) and extracted with DCM (3 x 10 mL). The combined organic layers were dried with Na_2SO_4 and concentrated *in vacuo* before being filtered through a Celite® plug and washed with DCM (20 mL), then subjected to purification using a Pure C-815 system (12-gram silica gel column, 100:0 hexanes/EtOAc → 20:80 hexanes/EtOAc; 20 minutes).

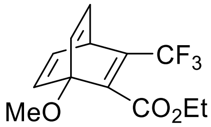

10

Ethyl 1-methoxy-3-(trifluoromethyl)bicyclo[2.2.2]octa-2,5,7-triene-2-carboxylate (10). General procedure A. Isolated as a clear oil that crystallized into a clear solid upon prolonged standing (29.6 mg, 0.108 mmol, 72%). **^{1}H NMR (800 MHz, CD_3CN) δ:** 7.10 (dd, J = 6.5, 1.6 Hz, 2H), 6.92 (t, J = 6.2 Hz, 2H), 4.87 (tt, J = 5.9, 1.6 Hz, 1H), 4.23 (q, J = 7.1 Hz, 2H), 3.66 (s, 3H), 1.24 (t, J = 7.1 Hz, 3H). **^{13}C NMR (201 MHz, CD_3CN) δ:** 165.5, 153.5 (q, J_{CF} = 4.8 Hz), 141.9, 139.9, 138.3 (q, J_{CF} = 34.7 Hz), 124.1 (q, J_{CF} = 269.1 Hz), 94.0, 62.8, 55.5, 46.5, 14.4. **^{19}F NMR (565 MHz, CD_2Cl_2) δ:** -67.9 (s, CF_3) ppm. **APCI-HRMS** (m/z): [M+H]+ calculated for $[C_{13}H_{14}F_3O_3]^+$: 275.0890; found: 275.0886. Composition confirmed by single-crystal X-ray diffraction.

^{1}H NMR impurity peaks δ: 2.14 (H_2O).

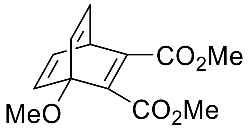

11

Dimethyl 1-methoxybicyclo[2.2.2]octa-2,5,7-triene-2,3-dicarboxylate (11). General procedure B. Clear microcrystalline solid (19.1 mg, isolated as a 10:1 mixture of **11**:**16**, 0.0690 mmol, 46%). Recrystallization from acetone/pentanes led to a 46:1 ratio of **11**:**16** for which NMR data were obtained: **^{1}H NMR (800 MHz, CD_3CN) δ:** 7.04 (dd, *J* = 6.5, 1.6 Hz, 2H), 6.90 (t, *J* = 6.2 Hz, 2H), 5.14 (tt, *J* = 5.9, 1.7 Hz, 1H), 3.74 (s, 3H), 3.67 (s, 3H), 3.65 (s, 3H). **^{13}C NMR (201 MHz, CD_3CN) δ:** 167.3, 164.3, 158.1, 141.9, 141.1, 140.6, 94.2, 55.3, 52.93, 52.86, 48.0. **ESI-HRMS** (m/z): [M+H]+ calculated for $[C_{13}H_{15}O_5]^+$: 251.0194; found: 251.0193. Composition confirmed by single-crystal X-ray diffraction.

^{1}H NMR impurity peaks δ: 2.13 (s; water).

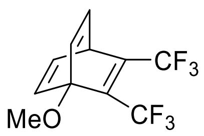

12

1-methoxy-2,3-bis(trifluoromethyl)bicyclo[2.2.2]octa-2,5,7-triene (12). General procedure A. Slightly yellow oil (10.1 mg, 57% purity with DCM, 0.0230 mmol, 15%). **NOTE:** Due to the volatility of the product under high-vacuum, residual DCM could not be fully removed and yield was calculated based on NMR integrals. The spectra also contained a small amount of unknown Tp salt from the oxidation step and an aromatic impurity identified as being from contaminated glassware, so assignments were made via two-dimensional NMR techniques (**NMR1**). Further purification was performed by vacuum distillation. A solution of **12** in $CDCl_3$ (2 mL) was added to a 10 mL round-bottom flask with a stir bar. A short path vacuum distillation head was attached to the distillation flask and an additional 10 mL receiving flask was attached to the other end and submerged in liquid nitrogen. The system was briefly put under vacuum until the solvent visibly boiled before the vacuum pump was removed and the solution stirred in this low pressure for 10 minutes. The solution in the receiving flask was then taken directly for ^{1}H NMR analysis, which showed the removal of the Tp salt and unknown aromatic impurity (**NMR2**). **NMR (800 MHz, $CDCl_3$) δ:** 7.07 (dd, J = 6.5, 1.7 Hz, 2H), 6.85 (t, J = 6.3 Hz, 2H), 5.04 (tt, J = 6.0, 1.7 Hz, 1H), 3.73 (s, 3H). **^{13}C NMR (201 MHz, $CDCl_3$) δ:** 147.2 (q, J_{CF} = 33.0 Hz), 145.2 (q, J_{CF} = 36.0 Hz), 141.8, 138.1, 122.1 (q, J_{CF} = 271.4 Hz)*, 121.4 (q, J_{CF} = 272.5 Hz)*, 92.9, 55.3, 47.1. **NOTE: 12** failed to ionize in APCI(+/-) and ESI(+/-) HRMS; therefore, EI-LRMS data was obtained: **EI-LRMS (GC/MS)** (m/z) (% relative intensity; ion): 201.0 (100; [M-CF_3]+), 270.0 (33; [M]+).

*The two CF_3 group carbons are interlaced carbon quartets.

^{1}H NMR impurity peaks (NMR1) δ: 8.24 (dd, unidentified), 7.72 (d, free Tp salt), 7.55 (dd, unidentified), 7.17 (d, free Tp salt), 6.08 (t, free Tp salt), 5.30 (s, DCM), 1.27 (m, n-hexane), 1.26 (br. s, grease), 0.88 (t, n-hexane), 0.86 (m, grease), and 0.07 (s, poly(dimethylsiloxane)) ppm.

^{13}C NMR impurity peaks (NMR1) δ: 140.1 (free Tp salt), 134.7 (free Tp salt), 129.5 (unidentified), 123.7 (unidentified), 103.8 (free Tp salt), 53.6 (DCM), 31.8 (n-hexane), 29.9 (grease), 22.9 (n-hexane), 14.3 (n-hexane), and 1.2 (poly(dimethylsiloxane)) ppm.

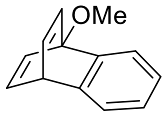

13

1-methoxy-1,4-dihydro-1,4-ethenonaphthalene (13). General procedure B. Slightly yellow oil (7.4 mg, 0.039 mmol, 26%). **^{1}H NMR (400 MHz, $CDCl_3$) δ:** 7.38 (dd, *J* = 7.3, 1.3 Hz, 1H), 7.15 (dd, *J* = 7.0, 1.4 Hz, 1H), 6.97 (m, 3H), 6.89 (m, 3H), 4.84 (tt, *J* = 5.8, 1.7 Hz, 1H), 3.85 (s, 3H). **^{13}C NMR (101 MHz, $CDCl_3$) δ:** 147.8, 145.9, 140.4, 138.5, 123.61, 123.55, 122.0, 119.3, 89.9, 54.6, 48.4. **ESI-HRMS** (m/z): [M+H]+ calculated for $[C_{13}H_{13}O]^+$: 185.0961; found: 185.0954.

^{1}H NMR impurity peaks δ: 0.09 (s, poly(dimethylsiloxane)) ppm.
^{13}C NMR impurity peaks δ: 1.2 (poly(dimethylsiloxane)) ppm.

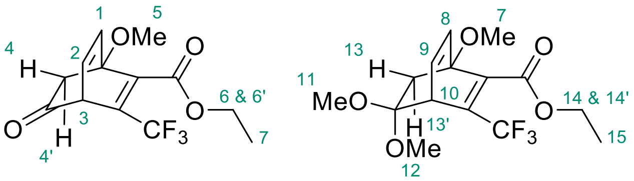

14 : **14a**

6.25 : 1

Ethyl 1-methoxy-8-oxo-3-(trifluoromethyl)bicyclo[2.2.2]octa-2,5-diene-2-carboxylate (14) and **ethyl (1*R*,4*R*)-1,8,8-trimethoxy-3-(trifluoromethyl)bicyclo[2.2.2]octa-2,5-diene-2-carboxylate (14a)**. General procedure B. Slightly yellow oil (25.9 mg, isolated as a 6.25:1 mixture of **14**:**14a**, 0.071 mmol, 47%). **^{1}H NMR (800 MHz, $CDCl_3$) δ:** 6.83 (dd, J = 7.6, 1.5 Hz, 1H, H1), 6.66 (dd, J = 7.7, 1.4 Hz, 1H, H8), 6.59 (ddd, J = 7.4, 6.3, 0.8 Hz, 1H, H2), 6.41 (dd, J = 7.8, 6.3 Hz, 1H, H9), 4.33 (qd, J = 7.1, 1.0 Hz, 2H, H6 and H6')*, 4.30 (m, 2H, H14 and H14')*, 4.23 (dd, J = 6.3, 1.4 Hz, 1H, H3), 3.94 (dd, J = 6.3, 1.4 Hz, 1H, H10), 3.56 (s, 3H, H5), 3.50 (s, 3H, H7), 3.20 (s, 3H, H11 or H12), 3.20 (s, 3H, H11 or H12), 2.35 (d, J = 16.4 Hz, 1H, H4 or H4'), 2.29 (d, J = 16.4 Hz, 1H, H4 or H4'), 1.92 (m, 2H, H13 and H13')*, 1.32 (m, 6H, H7 and H15).* **^{13}C NMR (201 MHz, $CDCl_3$) δ:** 197.3, 164.9, 163.9, 147.1 (q, J_{CF} = 3.8 Hz), 146.5 (q, J_{CF} = 4.4 Hz), 137.3, 135.0, 130.8, 130.4 (q, J_{CF} = 34.8 Hz), 127.6, 126.9 (q, J_{CF} = 35.6 Hz), ~122 (q, J_{CF} = 271.4 Hz), 121.8 (q, J_{CF} = 271.3 Hz), 108.2, 84.1, 83.4, 62.4, 61.9, 54.8, 53.9, 53.7, 49.8, 49.2, 43.4, 41.1, 37.1, 14.19, 14.16. **^{19}F NMR (565 MHz, CD_2Cl_2) δ:** -65.8 (s, CF_3), -66.4 (s, CF_3) ppm. **ESI-HRMS** (m/z) for **14**: [M+H]+ calculated for $[C_{13}H_{14}F_3O_4]^+$: 291.0839; found: 291.0831.

*Overlapping proton signals as indicated by HSQC.

^{1}H NMR impurity peaks δ: 0.06 (s, poly(dimethylsiloxane)) ppm.

^{13}C NMR impurity peaks δ: 1.2 (poly(dimethylsiloxane)) ppm.

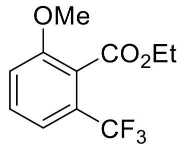

15

Ethyl 2-methoxy-6-(trifluoromethyl)benzoate (15). To a large test-tube charged with a stir-pea was added **4** (500 mg, 0.643 mmol) and MeCN (~5 mL) to form a brown slurry. After capping the test-tube and sealing the cap with electrical tape, this slurry was removed from the glovebox, heated to 70 °C, and allowed to stir for three days, over which period the slurry became a homogeneous dark brown solution. This crude reaction mixture was dry loaded onto silica gel and purified using a Combi*Flash*® (12-gram silica gel column; 100:0 → 0:100 hexanes/EtOAc; 20 minutes). Fractions containing the desired organic were combined, concentrated via rotary evaporation, and desiccated under active vacuum (bell jar) to provide yellow oil **15** (92 mg, 0.37 mmol, 58% yield). **^{1}H NMR (800 MHz, $CDCl_3$) δ:** 7.47 (t, *J* = 8.1 Hz, 1H), 7.25 (d, *J* = 8.0 Hz, 1H), 7.12 (d, *J* = 8.1 Hz, 1H), 4.41 (q, *J* = 7.2 Hz, 2H), 3.88 (s, 3H), 1.37 (t, *J* = 7.2 Hz, 3H). **^{13}C NMR (201 MHz, $CDCl_3$) δ:** 166.0, 156.9, 130.9, 128.7 (q, J_{CF} = 32.1 Hz), 123.5 (q, J_{CF} = 273.8 Hz), 122.6 (q, J_{CF} = 2.1 Hz), 118.1 (q, J_{CF} = 4.6 Hz), 114.8, 62.2, 56.6, 14.2. **^{19}F NMR (565 MHz, CD_2Cl_2) δ:** -62.4 (s, CF_3) ppm. **APCI-HRMS** (m/z): [M]+ calculated for $[C_{11}H_{11}F_3O_3]^+$: 248.0660; found: 248.0660. **IR**: v(CO) 1734.78 cm^{-1}.

Alternative one-pot procedure for the synthesis of **15**. To a medium test-tube charged with a stir-pea was added **1** (348 mg, 0.569 mmol) and dry 1,4-dioxane (~2 mL). Stirring was initiated followed by the addition of neat ethyl 4,4,4-trifluorobut-2-ynoate (218 mg, 1.31 mmol). After capping the test-tube and sealing the cap with electrical tape, the reaction was stirred at room temperature for 5 min. The test-tube was the removed from the glovebox, heated to 90 °C, and allowed to stir for 41 h. This crude reaction mixture was dry loaded onto silica gel and purified using a Combi*Flash*® (12-gram silica gel column; 100:0 → 0:100 hexanes/EtOAc; 20 minutes). Fractions containing the desired organic were combined, concentrated via rotary evaporation, and desiccated under active vacuum (bell jar) to provide yellow oil **15** (126 mg, 0.508 mmol, 89% yield).

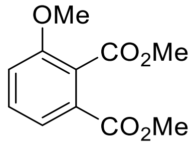

16

Dimethyl 3-methoxyphthalate (16). To a large test-tube charged with a stir-pea was added **5** (719 mg, 0.955 mmol) and MeCN (~5 mL) to form a brown slurry. After capping the test-tube and sealing the cap with electrical tape, this slurry was removed from the glovebox, heated to 70 °C, and allowed to stir for six days, over which period the slurry became a homogeneous dark brown solution. This crude reaction mixture was dry loaded onto silica gel and purified using a Combi*Flash*® (12-gram silica gel column; 100:0 → 0:100 hexanes/EtOAc; 20 minutes). Fractions containing the desired organic were combined and concentrated to an oil via rotary evaporation, and subsequent desiccation of this oil under active vacuum (bell jar) provided off-white solid **16** (164 mg, 0.73 mmol, 77% yield). **^{1}H NMR (800 MHz, $CDCl_3$) δ:** 7.57 (dd, *J* = 7.8, 1.0 Hz, 1H), 7.39 (t, *J* = 8.1 Hz, 1H), 7.11 (d, *J* = 8.4 Hz, 1H), 3.92 (s, 3H), 3.85 (s, 3H), 3.83 (s, 3H). **^{13}C NMR (201 MHz, $CDCl_3$) δ**: 168.0, 165.8, 156.5, 130.4, 128.7, 125.4, 122.0, 115.6, 56.4, 52.8, 52.6. **IR**: ν(CO) 1721.45 cm^{-1}. Composition confirmed by single-crystal X-ray diffraction.

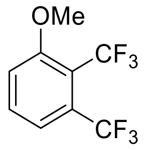

17

1-methoxy-2,3-bis(trifluoromethyl)benzene (17). To a large test-tube charged with a stir-pea was added **6** (472 mg, 0.611 mmol) and MeCN (~3 mL) to form a brown slurry. After capping the test-tube and sealing the cap with electrical tape, this slurry was removed from the glovebox, heated to 70 °C, and allowed to stir for 92 h, over which period the slurry became a homogeneous dark brown solution. This crude reaction mixture was dry loaded onto silica gel and purified using a Combi*Flash*® (12-gram silica gel column; 100:0 → 0:100 hexanes/EtOAc; 20 minutes). Fractions containing the desired organic were combined and concentrated to an oil via rotary evaporation. The oil was redissolved in Et_2O (~1 mL) which was subsequently removed via rotary evaporation; this process was then repeated twice more to provide clear oil **17** (47 mg, 0.19 mmol, 59% purity with Et_2O, 32% yield). **NOTE:** Due to the volatility of the product under high-vacuum, residual Et_2O could not be fully removed, and yield was calculated based on NMR integrals. **^{1}H NMR (800 MHz, CD_3CN) δ:** 7.73 (t, J = 8.3 Hz, 1H), 7.49 (m, 2H), 3.95 (s, 3H). **^{13}C NMR (201 MHz, CD_3CN) δ:** 160.7, 134.9, 129.4 (q, J_{CF} = 32.7 Hz), ~124.3 (q, J_{CF} = 273.0 Hz)*, ~124.3 (q, J_{CF} = 273.1 Hz)*, 120.2 (q, J_{CF} = 7.6 Hz), 118.9, 116.6 (q, J_{CF} = 31.7 Hz), 57.8. **^{19}F NMR (565 MHz, CD_2Cl_2) δ:** -59.6 (q, J_{FF} = 16.1 Hz, CF_3), -60.6 (q, J_{FF} = 16.0 Hz, CF_3) ppm. **APCI-HRMS** (m/z): [M-CH_3]- calculated for [$C_8H_3F_6O$]-: 229.0094; found: 229.0092.

*****The two CF_3 group carbons are interlaced carbon quartets.

^{1}H NMR impurity peaks δ: 3.42 (q, Et_2O), 1.12 (t, Et_2O).
^{13}C NMR impurity peaks δ: 66.4 (Et_2O), 15.7 (Et_2O).

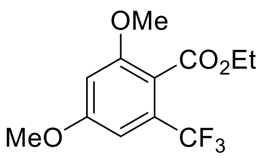

18

Ethyl 2,4-dimethoxy-6-(trifluoromethyl)benzoate (18). To a 25 mL round-bottom flask was added **8** (161 mg, 0.200 mmol) and toluene (10 mL). The solution was refluxed under nitrogen for five days. The reaction mixture was then concentrated *in vacuo*. The crude reaction mixture was purified using a Pure C-815 system (4-gram silica gel column; 100:0 → 30:70 hexanes/EtOAc; 20 minutes), providing **18** as a clear oil (24.6 mg, 0.0880 mmol, 44% yield). **^{1}H NMR (400 MHz, $CDCl_3$) δ:** 6.72 (d, J = 2.2 Hz, 1H), 6.62 (d, J = 2.2 Hz, 1H), 4.37 (q, J = 7.1 Hz, 2H), 3.85 (s, 3H), 3.84 (s, 3H), 1.35 (t, J = 7.2 Hz, 3H). **^{13}C NMR (101 MHz, $CDCl_3$) δ:** 166.0, 161.6, 158.4, 129.6 (q, J_{CF} = 32.2 Hz), 123.4 (q, J_{CF} = 273.9 Hz), 115.6 (q, J_{CF} = 2.0 Hz), 102.5, 101.9, 62.0, 56.5, 55.9, 14.1. **^{19}F NMR (565 MHz, CD_2Cl_2) δ:** -62.5 (s, CF_3) ppm. **ESI-HRMS** (m/z): [M+H]+ calculated for $[C_{12}H_{14}F_3O_4]^+$: 279.0839; found: 279.0838.

^{1}H NMR impurity peaks δ: 0.07 (s, poly(dimethylsiloxane)) ppm.

^{13}C NMR impurity peaks δ: 1.2 (poly(dimethylsiloxane)) ppm.

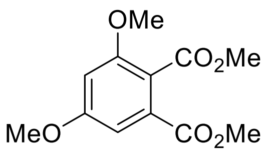

19

Dimethyl 3,5-dimethoxyphthalate (19). To a flame-dried 25 mL round-bottom flask charged with a stir-egg was added **9** (317 mg, 0.405 mmol) and toluene (10 mL) to form a brown solution. This solution was refluxed under nitrogen for two days. The crude reaction mixture was dry loaded onto silica gel and purified using a Combi*Flash*® (12-gram silica gel column; 100:0 → 0:100 hexanes/EtOAc; 20 minutes). Fractions containing the desired organic were combined and concentrated via rotary evaporation and then dried under active vacuum (bell jar) overnight to provide **19** as a clear crystalline solid (57 mg, 0.22 mmol, 55% yield). **^{1}H NMR (400 MHz, CDCl$_3$) δ:** 7.06 (d, *J* = 2.3 Hz, 1H), 6.65 (d, *J* = 2.3 Hz, 1H), 3.91 (s, 3H), 3.88 (s, 3H), 3.85 (s, 3H), 3.83 (s, 3H). **^{13}C NMR (101 MHz, CDCl$_3$) δ:** 168.1, 166.1, 161.4, 158.0, 130.2, 118.5, 105.6, 103.2, 56.5, 55.9, 52.83, 52.82. **IR**: ν(CO) 1720.82 cm^{-1}. **APCI-HRMS** (m/z): [M+H]+ calculated for $[C_{12}H_{15}O_6]^+$: 255.0863; found: 255.0865. Composition confirmed by single-crystal X-ray diffraction.

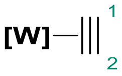

20

TpW(NO)(PMe_3)(η^2-acetylene) (20). To a large test-tube charged with a stir-pea was added **4** (986 mg, 1.27 mmol) and MeCN (~5 mL) to form a brown slurry. After capping the test-tube and sealing the cap with electrical tape, this slurry was removed from the glovebox, heated to 70 °C, and allowed to stir for 27 h, over which period the slurry became a homogeneous dark brown solution. This crude reaction mixture was dry loaded onto silica gel and purified using a Combi*Flash*® (12-gram silica gel column; 100:0→ 0:100 hexanes/EtOAc over 20 minutes followed by a 100% EtOAc hold for 7 minutes). Fractions containing the desired complex were combined and concentrated to a brown oily solid via rotary evaporation. This oil was redissolved in DCM (minimal) and precipitated into pentane (~100 mL). The resulting brown precipitate was collected on a 15 mL fine porosity fritted disc and washed with pentane (~15 mL). The filtrate was subsequently concentrated to a solid via rotary evaporation. Both the precipitate and material were dried under active vacuum (bell jar), providing brown powder **20** (249 mg, 0.471 mmol, 37% yield). **^{1}H NMR (800 MHz, CD_3CN) δ:** 9.26 (m, 1H, H1), 8.86 (m, 1H, H2), 8.21 (d, *J* = 1.5 Hz, 1H, Tp), 8.16 (d, *J* = 1.7 Hz, 1H, Tp), 7.89 (d, *J* = 2.2 Hz, 1H, Tp), 7.74 (d, *J* = 2.2 Hz, 1H, Tp), 7.70 (d, *J* = 2.1 Hz, 1H, Tp), 7.10 (d, *J* = 1.7 Hz, 1H, Tp), 6.40 (t, *J* = 2.2 Hz, 1H, Tp), 6.30 (t, *J* = 2.1 Hz, 1H, Tp), 6.09 (t, *J* = 2.1 Hz, 1H, Tp), 1.44 (d, *J* = 8.8 Hz, 9H, PMe_3)**. ^{13}C NMR (201 MHz, CD_3CN) δ:** 146.1, 145.5, 144.5, 143.0 (d, J_{CP} = 5.5 Hz), 137.4, 136.9, 136.5, 133.6 (d, J_{CP} = 24.0 Hz), 107.24, 107.19, 106.2, 15.2 (d, J_{CP} = 30.5 Hz). Proton-coupled ^{13}C NMR data were obtained for **20**, and the one-bond ^{13}C-^{1}H coupling constants obtained ($J_{C1\text{-}H1}$ = 200.6 Hz; $J_{C2\text{-}H2}$ = 200.2 Hz) are consistent with other reported tungsten-η^2-acetylene complexes.[4] **APCI-HRMS** (m/z): [M]+ calculated for [$C_{14}H_{21}BN_7OPW$]+: 529.1148; found: 529.1126. **CV** (MeCN; 100 mV/s): $E_{p,a}$ = +0.58 V (NHE). Composition confirmed by single-crystal X-ray diffraction.

^{1}H NMR impurity peaks δ: 2.09 (s, acetone) ppm.
^{13}C NMR impurity peaks δ: 31.0 (acetone) ppm.

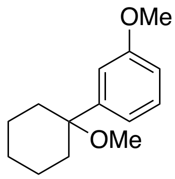

24

1-methoxy-3-(1-methoxycyclohexyl)benzene (24). To a 250 mL pressure flask was added 1-(3-methoxyphenyl)cyclohexan-1-ol[5] (9.48 g, 46.0 mmol) and dry THF (150 mL). The solution was cooled to 0 °C and sodium hydride (2.76 g, 67 mmol, 60% w/w in mineral oil) was added portion-wise, then stirred for 30 minutes. Methyl iodide (14.3 mL, 230 mmol) was then added, and the flask was sealed then allowed to stir overnight while returning to room temperature. The reaction was quenched with saturated ammonium chloride solution (40 mL) and extracted with EtOAc (3 x 30 mL) then the combined organic layers were washed with brine (30 mL) and dried with Na_2SO_4. Purified by column chromatography (100-gram silica gel column; 100:0 → 80:20 hexanes/EtOAc; 20 minutes) to give a clear liquid that was dried on high-vacuum for 2 hours (6.34 g, 59% yield). **^{1}H NMR (400 MHz, CDCl$_3$) δ:** 7.26 (m, 1H), 6.99 (m, 2H), 6.79 (ddd, *J* = 8.2, 2.5, 1.1 Hz, 1H), 3.81 (s, 3H), 2.98 (s, 3H), 2.01 (m, 2H), 1.64 (m, 7H), 1.26 (m, 1H). **^{13}C NMR (101 MHz, CDCl$_3$) δ:** 159.9, 148.2, 129.3, 118.7, 112.3, 112.2, 77.6, 55.4, 49.9, 35.6, 25.9, 22.2.

NOTE: 24 failed to ionize in APCI(+/-) HRMS; therefore, APCI-LRMS data was obtained: **APCI-LRMS (direct injection)** (m/z) (% relative intensity; ion): 189.0 (100; [M-OMe]+).

^{1}H NMR impurity peaks δ: 0.07 (s, poly(dimethylsiloxane)) ppm.

^{13}C NMR impurity peaks δ: 1.2 (poly(dimethylsiloxane)) ppm.

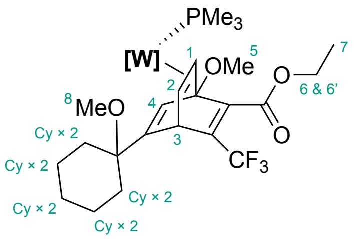

21

Proximal TpW(NO)(PMe_3)(5,6-η^2-ethyl (1*S*,4*S*)-1-methoxy-5-(1-methoxycyclohexyl)-3-(trifluoromethyl)bicyclo[2.2.2]octa-2,5,7-triene-2-carboxylate) (21). To an oven-dried 4-dram vial charged with a stir-pea was added **1** (538 mg, 0.880 mmol) and 1-methoxy-3-(1-methoxycyclohexyl)benzene (1120 mg, 5.08 mmol). Dry DME (~4 mL) was added and stirring was initiated. The reaction was allowed to stir for 21.5 hours, at which point neat ethyl 4,4,4-trifluorobut-2-ynoate (327 mg, 1.97 mmol) was added. The solution was allowed to stir for 20 more minutes. This crude reaction mixture was dry loaded onto silica gel and purified using a Combi*Flash*® (12-gram silica gel column; 100:0 → 70:30 hexanes/EtOAc; 20 minutes). Fractions containing the desired complex were combined and concentrated to a yellow amorphous solid via rotary evaporation, and subsequent desiccation of this solid under active vacuum (bell jar) provided yellow solid **21** (154 mg, 0.173 mmol, 20% yield). **^{1}H NMR (600 MHz, CD_2Cl_2) δ:** 8.30 (d, *J* = 2.1 Hz, 1H, Tp), 8.22 (d, *J* = 1.4 Hz, 1H, Tp), 7.79 (d, *J* = 2.3 Hz, 1H, Tp), 7.69 (d, *J* = 2.3 Hz, 1H, Tp), 7.55 (d, *J* = 2.3 Hz, 1H, Tp), 7.41 (d, *J* = 2.2 Hz, 1H, Tp), 6.38 (t, *J* = 2.2 Hz, 1H, Tp), 6.32 (m, 1H, H4), 6.20 (t, *J* = 2.2 Hz, 1H, Tp), 6.18 (t, *J* = 2.2 Hz, 1H, Tp), 4.35 (m, 1H, H6 or H6'), 4.30 (m, 1H, H3), 4.24 (m, 1H, H6 or H6'), 3.60 (s, 3H, H5), 3.27 (t, *J* = 11.0 Hz, 1H, H1), 2.85 (s, 3H, H8), 2.17 (m, 1H, Cy), 2.05 (m, 1H, Cy), 1.97 (m, 1H, Cy), 1.90 (m, 2H, Cy overlapping with H2), 1.72 (qt, *J* = 13.0, 4.1 Hz, 1H, Cy), 1.54 (m, 3H, three overlapping Cy)*‡, 1.36 (m, 4H, Cy overlapping with H7)*, 1.21 (d, *J* = 9.1 Hz, 9H, PMe_3), 1.12 (qt, *J* = 13.0, 3.7 Hz, 1H, Cy). **^{13}C NMR (201 MHz, CD_2Cl_2) δ:** 167.6, 152.6 (q, J_{CF} = 5.3 Hz), 148.4, 146.2, 143.4, 142.22 (q, J_{CF} = 31.6 Hz)**, 142.20**, 137.4, 136.5, 135.6, 128.4, 124.6 (q, J_{CF} = 271.0 Hz), 107.2, 106.2, 106.0, 98.3, 76.2, 68.8 (d, J_{CP} = 17.6 Hz), 66.9, 61.6, 55.7, 49.1, 44.1, 34.8, 28.6, 26.0, 22.3, 22.1, 14.4, 13.7 (d, J_{CP} = 29.0 Hz). Composition confirmed by single-crystal X-ray diffraction.

*Overlapping proton signals as indicated by HSQC.

‡Overlap with H_2O impurity

**HMBC data indicate a ^{13}C singlet at 142.20 ppm interlaced with a ^{13}C quartet centered at 142.22 ppm.

Impurity ^{1}H NMR signals at: 4.08 (q, EtOAc), 2.00 (s, EtOAc), 1.54 (H_2O), and 1.23 (EtOAc) ppm.

Impurity ^{13}C NMR signals at: 60.8 (EtOAc), 21.4 (EtOAc), and 14.5 (EtOAc) ppm.

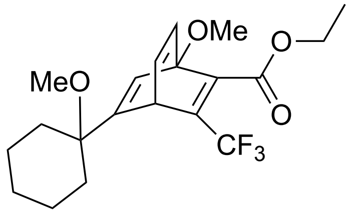

22

Ethyl (1*S*,4*S*)-1-methoxy-5-(1-methoxycyclohexyl)-3-(trifluoromethyl)bicyclo[2.2.2]octa-2,5,7-triene-2-carboxylate (22). To a solution of **21** (65 mg, 0.073 mmol) in THF (1.5 mL) was added DDQ (38 mg, 0.17 mmol) and the reaction was allowed to stir at -45 °C for 1 hour. The reaction mixture was quenched with saturated aqueous K_2CO_3 (5 mL), extracted with DCM (3 x 10 mL), washed with saturated aqueous K_2CO_3 (5 mL), and dried with Na_2SO_4. The organics were then concentrated *in vacuo* to an oil, redissolved in DCM (minimal), then subjected to purification using a Pure C-815 system (4-gram silica gel column, 100:0 → 80:20 hexanes/EtOAc; 20 min) to provide **22** as a colorless crystalline solid (20.6 mg, 0.0533 mmol, 73%). **^{1}H NMR (600 MHz, $CDCl_3$) δ:** 7.03 (dd, *J* = 6.8, 1.6 Hz, 1H), 6.87 (t, *J* = 6.4 Hz, 1H), 6.67 (d, *J* = 2.0 Hz, 1H), 4.88 (dt, *J* = 6.0, 1.9 Hz, 1H), 4.29 (q, *J* = 7.1 Hz, 2H), 3.70 (s, 3H), 2.88 (s, 3H), 1.85 (m, 2H), 1.56 (m, 3H), 1.45 (m, 2H), 1.35 (m, 2H), 1.29 (t, *J* = 7.1 Hz, 3H), 1.20 (m, 1H). **^{13}C NMR (151 MHz, $CDCl_3$) δ:** 165.0, 156.9, 152.1 (q, J_{CF} = 4.5 Hz), 141.3, 139.0, 138.1 (q, J_{CF} = 34.5 Hz), 135.6, 123.0 (q, J_{CF} = 270.2 Hz), 92.7, 75.5, 62.0, 55.2, 50.2, 45.6 (q, J_{CF} = 1.6 Hz), 33.1, 32.0, 25.9, 21.7*, 14.2. Composition confirmed by single-crystal X-ray diffraction.

*HSQC data indicate two overlapping ^{13}C signals.

Impurity ^{1}H NMR signals at: 0.07 (poly(dimethylsiloxane)) ppm.
Impurity ^{13}C NMR signals at: 1.23 (poly(dimethylsiloxane)) ppm.

<u>**NOTE:**</u> Product was visualized on TLC with $KMnO_4$ stain.

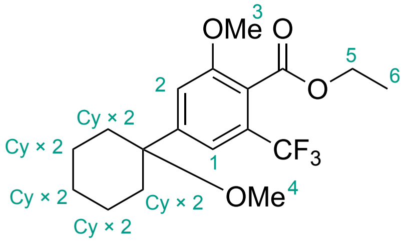

23

Ethyl 2-methoxy-4-(1-methoxycyclohexyl)-6-(trifluoromethyl)benzoate (23). To a 50 mL round-bottom flask charged with a stir-egg was added a solution of **21** (146 mg, 0.164 mmol) in xylenes (10 mL). A water condenser was placed over the round-bottom flask, and the solution was placed under nitrogen atmosphere. The solution was then heated to 130 °C and the reaction allowed to proceed under nitrogen atmosphere for 22 hours. The reaction was then allowed to cool to room temperature. After adding methanol to form an azeotrope, the organics were concentrated *in vacuo*, redissolved in DCM (minimal), passed through a silica plug, and washed with DCM (~50 mL). The filtrate was then concentrated *in vacuo* to a clear oil, redissolved in DCM (minimal), then subjected to purification using a Pure C-815 system (4-gram silica gel column, 100:0 → 85:15 hexanes/EtOAc; 20 min) to provide **23** as a clear oil (16.6 mg, 0.0461 mmol, 29% yield). **^{1}H NMR (400 MHz, $CDCl_3$) δ:** 7.23 (br. s, 1H, H1), 7.22 (br. s, 1H, H2), 4.41 (q, J = 7.2 Hz, 2H, H5), 3.88 (s, 3H, H3), 2.99 (s, 3H, H4), 2.00 (m, 2H, two overlapping Cy)*, 1.68 (m, 7H, 7 overlapping Cy)*, 1.37 (t, J = 7.2 Hz, 3H, H6), 1.25 (m, 1H, Cy)*. **^{13}C NMR (151 MHz, $CDCl_3$) δ:** 166.1, 157.2, 150.6, 128.5 (q, J_{CF} = 31.9 Hz), 123.6 (q, J_{CF} = 274.1 Hz), 115.7 (q, J_{CF} = 4.9 Hz), 112.6, 77.7, 62.2, 56.6, 50.1, 35.6, 25.6, 21.9, 14.2. **NOTE: 23** failed to ionize in APCI(+/-) HRMS and EI-LRMS (GC/MS); therefore, APCI-LRMS data was obtained: **APCI-LRMS (TLC-MS)** (m/z) (% relative intensity; ion): 329.2 (30; [M-OMe]+), 361.2 (100; [M+H]+).

*Overlapping proton signals as indicated by HSQC.

Impurity ^{1}H NMR signals at: 0.07 (poly(dimethylsiloxane)) ppm.
Impurity ^{13}C NMR signals at: 1.23 (poly(dimethylsiloxane)) ppm.

<u>**NOTE:**</u> Compound **23** fluoresces on TLC only very weakly under UV light but stains with phosphomolybdic acid.

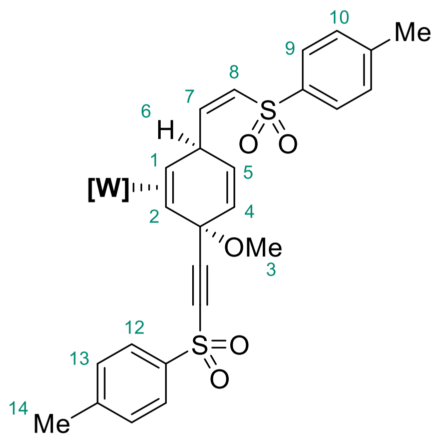

S1

TpW(NO)(PMe$_3$)(2,3-*η*^{2}-1-((((1*r*,4*r*)-1-methoxy-4-((*Z*)-2-tosylvinyl)cyclohexa-2,5-dien-1-yl)ethynyl)sulfonyl)-4-methylbenzene) (S1). To a test-tube charged with a stir-pea was added **1** (242 mg, 0.396 mmol), 1-(ethynylsulfonyl)-4-methylbenzene (160 mg, 0.888 mmol), and THF (~1.5 mL). Stirring was initiated and the reaction was allowed to proceed for five days. After five days, the reaction mixture was dry-loaded onto silica gel and purified using a Combi*Flash*® (12-gram silica gel column; 100:0 → 0:100 hexanes/EtOAc; 30 minutes). Fractions containing the crude product were combined, concentrated to a yellow oily solid via rotary evaporation, and dried under active vacuum (bell jar) for 12 hours. The solid was then triturated in MeCN for one day, collected on a 15 mL fine-porosity fritted disc, washed with MeCN (~15 mL), and desiccated under static vacuum, providing **S1** as a white powder (34 mg, 0.035 mmol, 9% yield). **^{1}H NMR (800 MHz, CD$_2$Cl$_2$) δ:** 8.45 (d, *J* = 1.7 Hz, 1H, Tp), 8.10 (d, *J* = 1.7 Hz, 1H, Tp), 7.91 (m, 5H, H9, H12, and Tp overlapping)*, 7.83 (d, *J* = 2.3 Hz, 1H, Tp), 7.71 (d, *J* = 2.2 Hz, 1H, Tp), 7.52 (d, *J* = 7.9 Hz, 2H, H13), 7.45 (d, *J* = 1.7 Hz, 1H, Tp), 7.43 (d, *J* = 7.9 Hz, 2H, H10), 6.43 (t, *J* = 2.2 Hz, 1H, Tp), 6.41 (t, *J* = 2.2 Hz, 1H, Tp), 6.33 (t, *J* = 10.9 Hz, 1H, H7), 6.22 (t, *J* = 2.2 Hz, 1H, Tp), 6.12 (d, *J* = 10.9 Hz, 1H, H8), 5.94 (dt, *J* = 9.8, 1.2 Hz, 1H, H4), 5.68 (ddd, *J* = 9.8, 6.4, 1.0 Hz, 1H, H5), 5.18 (dd, *J* = 10.8, 6.5 Hz, 1H, H6), 3.01 (s, 3H, H3), 2.69 (dd, *J* = 14.0, 11.7 Hz, 1H, H1), 2.58 (s, 3H, H14), 2.51 (s, 3H, H11), 2.02 (dt, *J* = 11.2, 1.5 Hz, 1H, H2), 1.24 (d, *J* = 8.5 Hz, 9H, PMe$_3$). **^{13}C NMR (201 MHz, CD$_2$Cl$_2$) δ:** 149.7, 148.4, 145.9, 145.3, 142.9, 140.7, 139.7, 139.6, 137.3, 136.7, 136.6, 130.6, 130.5, 129.1, 129.0, 127.8, 127.6, 127.5, 107.0, 106.8, 105.7, 102.2, 82.0, 77.7, 57.3, 52.4 (d, J_{CP} = 13.1 Hz), 51.9, 40.0 (d, J_{CP} = 3.3 Hz), 22.0, 21.9, 13.2 (d, J_{CP} = 28.3 Hz). CV (MeCN; 100 mV/s): $E_{p,a}$ = +0.78 V (NHE). Composition confirmed by single-crystal X-ray diffraction.

*Three overlapping proton signals as indicated by HSQC.

^{1}H NMR impurity peaks δ: 3.54 (m, unidentified) and 2.25 (s, MeCN).

^{13}C NMR impurity peaks δ: 117.1 (MeCN) and 2.25 (MeCN).

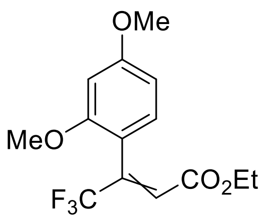

S2

Ethyl 3-(2,4-dimethoxyphenyl)-4,4,4-trifluorobut-2-enoate (S2). To a large test-tube charged with a stir-pea was added **8** (374 mg, 0.463 mmol) and MeCN (~4 mL) to form a brown slurry. After capping the test-tube and sealing the cap with electrical tape, this slurry was removed from the glovebox, heated to 70 °C, and allowed to stir for 17 days. This crude reaction mixture was dry-loaded onto silica gel and an attempt was made to purify the organic using a Combi*Flash*® (12-gram silica gel column; 100:0 → 30:70 hexanes/EtOAc; 20 minutes). After concentrating the fractions containing product to an oil and subsequent desiccation under active vacuum (bell jar), ^{1}H NMR and TLC-MS indicated the presence of both **S2** and **18**. Therefore, further purification was performed by Combi*Flash*® (4 gram silica gel column; 100:0 → 40:60 hexanes/EtOAc; 20 minutes), providing **S2** as a clear oil (13 mg, 0.043 mmol, 9% yield). **^{1}H NMR (600 MHz, $CDCl_3$) δ:** 7.11 (d, J = 8.1 Hz, 1H), 6.48 (m, 2H), 6.22 (s, 1H), 4.29 (q, J = 7.2 Hz, 2H), 3.83 (s, 3H), 3.81 (s, 3H), 1.34 (t, J = 7.1 Hz, 3H). **^{13}C NMR (151 MHz, $CDCl_3$) δ:** 164.4, 162.3, 158.7, 131.3, 128.7 (q, J_{CF} = 2.7 Hz), 125.8 (q, J_{CF} = 5.4 Hz), 122.1 (q, J_{CF} = 276.1 Hz), 116.6, 104.6, 98.9, 61.6, 55.9, 55.7, 14.2. **ESI-HRMS** (m/z): [M+H]+ calculated for $[C_{14}H_{16}F_3O_4]^+$: 305.0995; found: 305.0989.

^{1}H NMR impurity peaks δ: 0.07 (s, poly(dimethylsiloxane)) ppm.
^{13}C NMR impurity peaks δ: 1.2 (poly(dimethylsiloxane)) ppm.

NMR SPECTRA

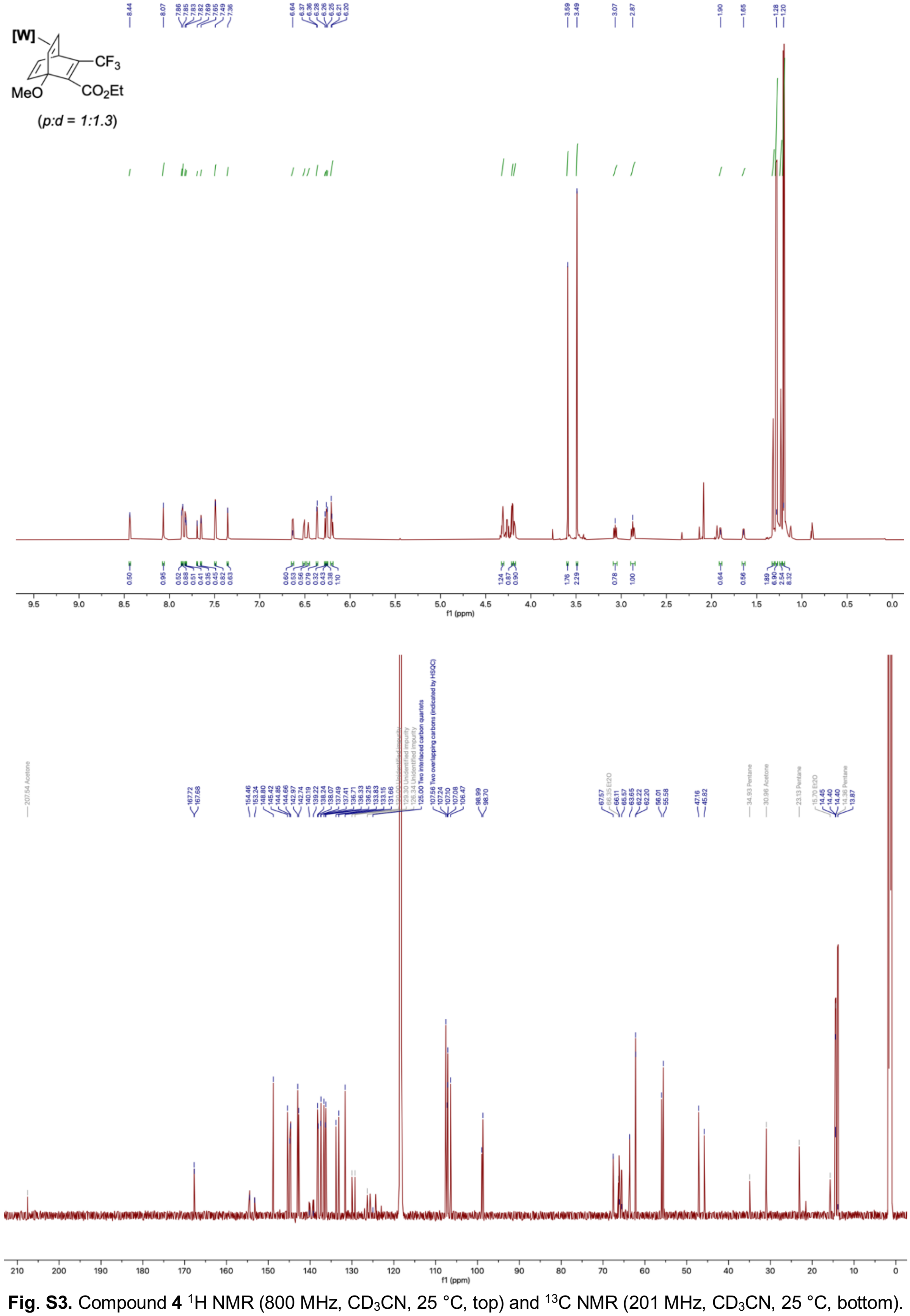

Fig. **S3.** Compound **4** ^{1}H NMR (800 MHz, CD_3CN, 25 °C, top) and ^{13}C NMR (201 MHz, CD_3CN, 25 °C, bottom).

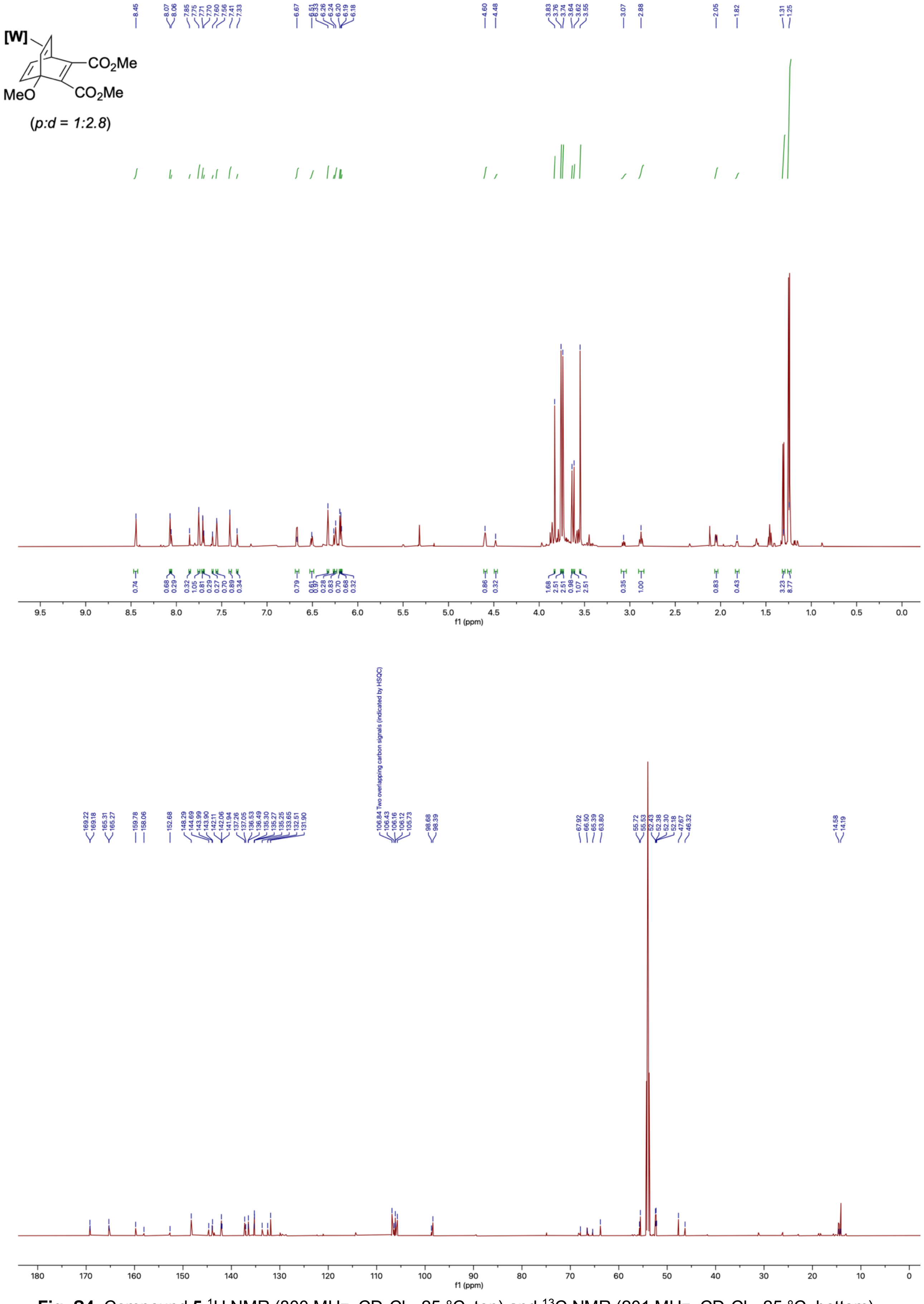

Fig. **S4.** Compound **5** ^{1}H NMR (800 MHz, CD_2Cl_2, 25 °C, top) and ^{13}C NMR (201 MHz, CD_2Cl_2, 25 °C, bottom).

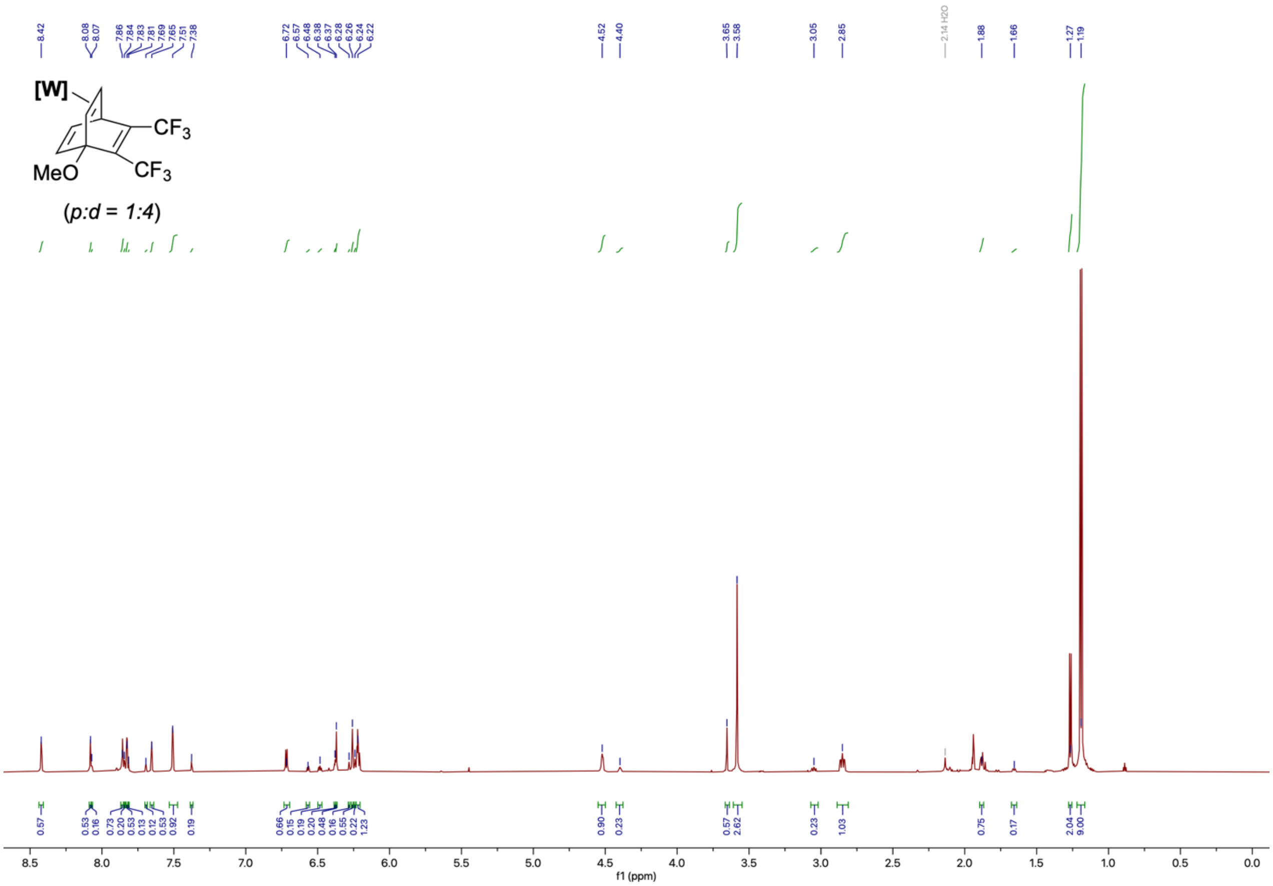

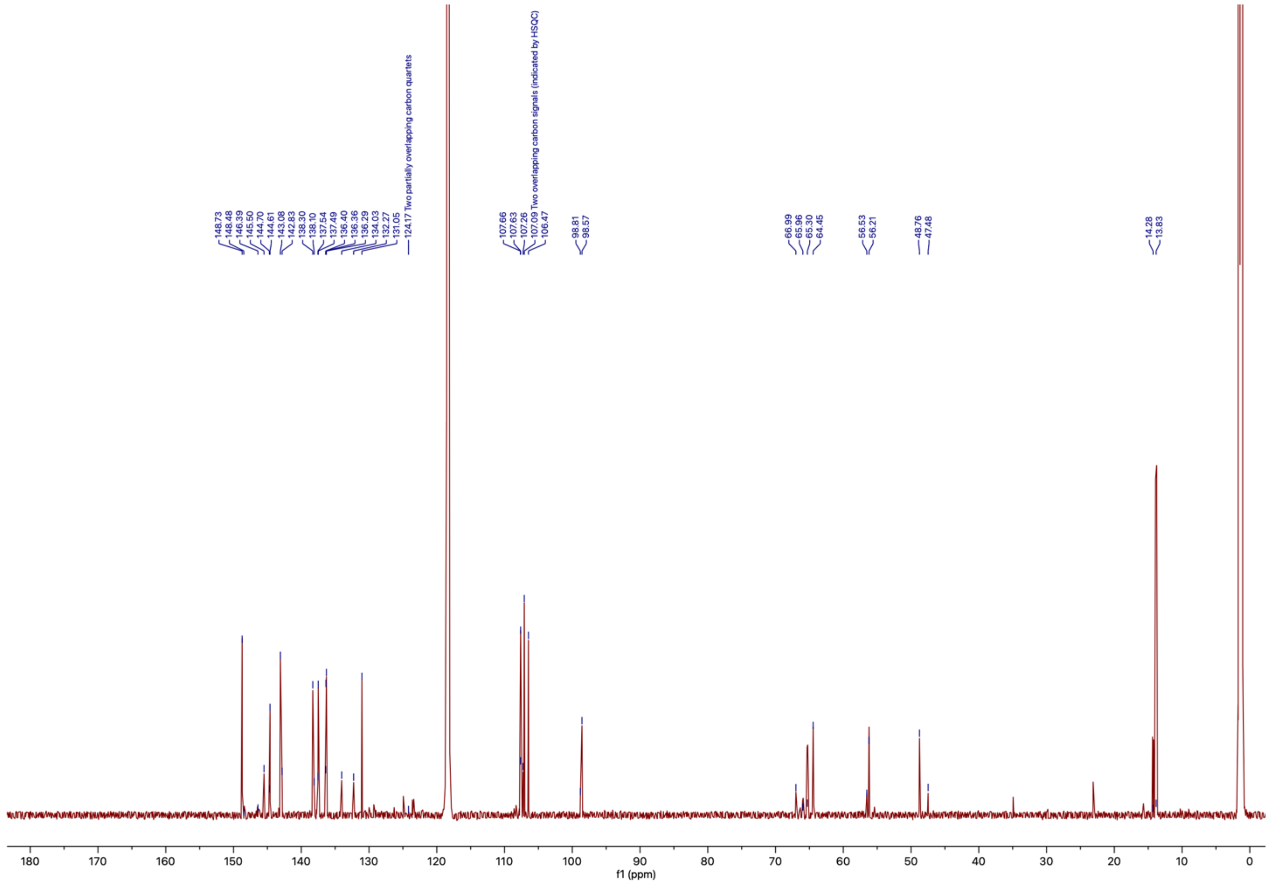

Fig. S5. Compound **6** ^{1}H NMR (800 MHz, CD_3CN, 25 °C, top) and ^{13}C NMR (201 MHz, CD_3CN, 25 °C, bottom).

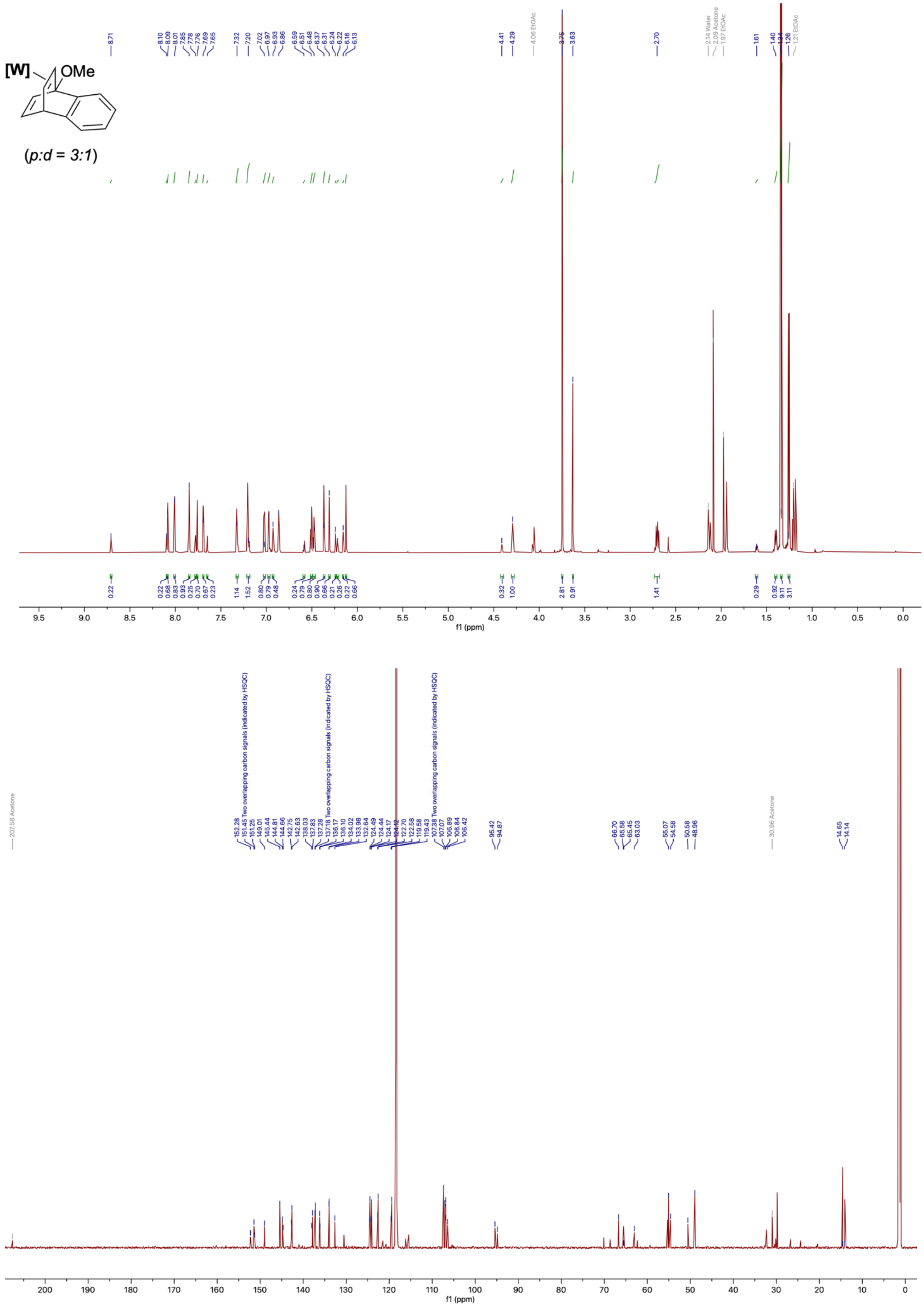

Fig. S6. Compound **7** ^{1}H NMR (800 MHz, CD_3CN, 25 °C, top) and ^{13}C NMR (201 MHz, CD_3CN, 25 °C, bottom).

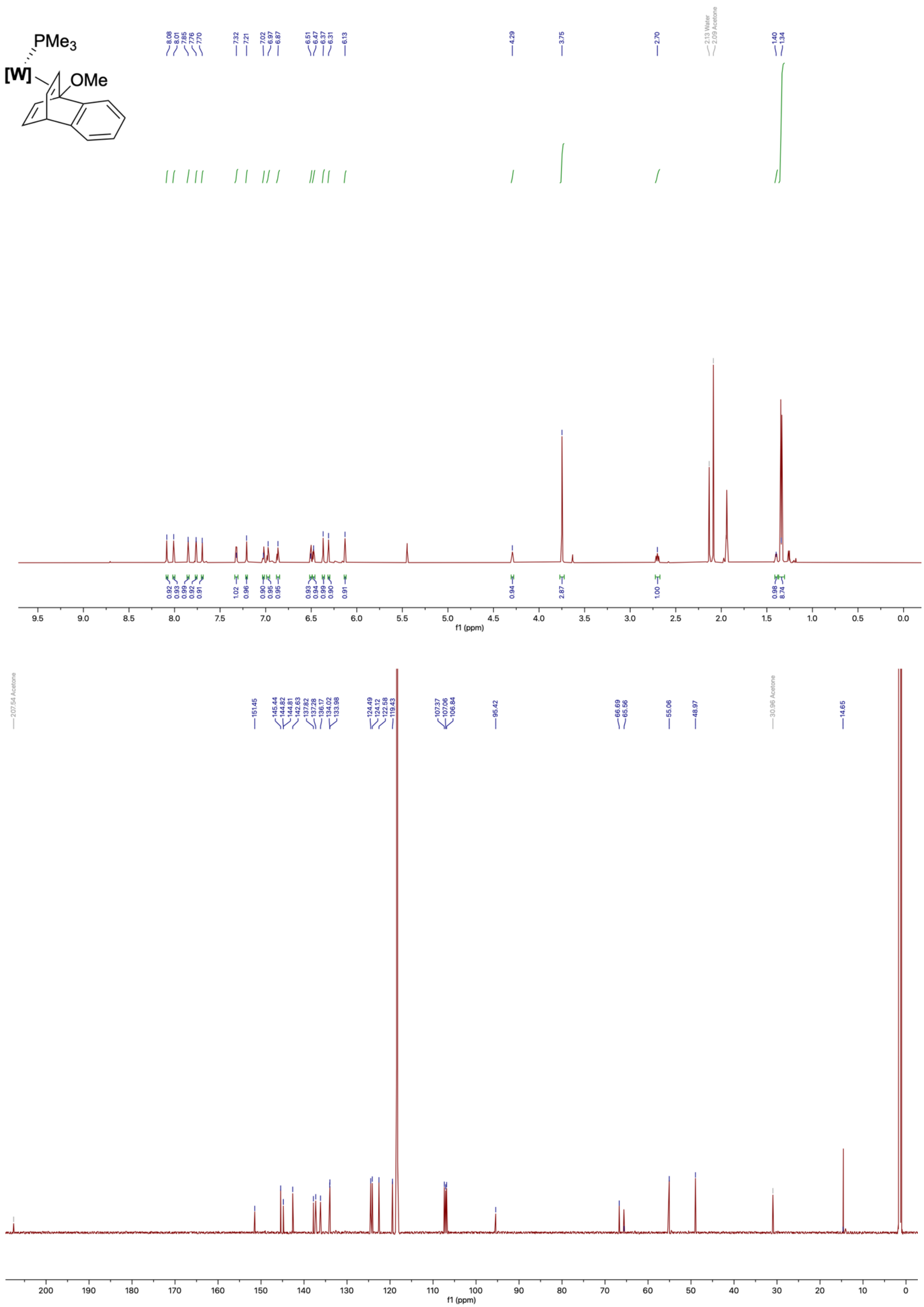

Fig. **S7.** Compound **7p** ^{1}H NMR (800 MHz, CD_3CN, 25 °C, top) and ^{13}C NMR (201 MHz, CD_3CN, 25 °C, bottom).

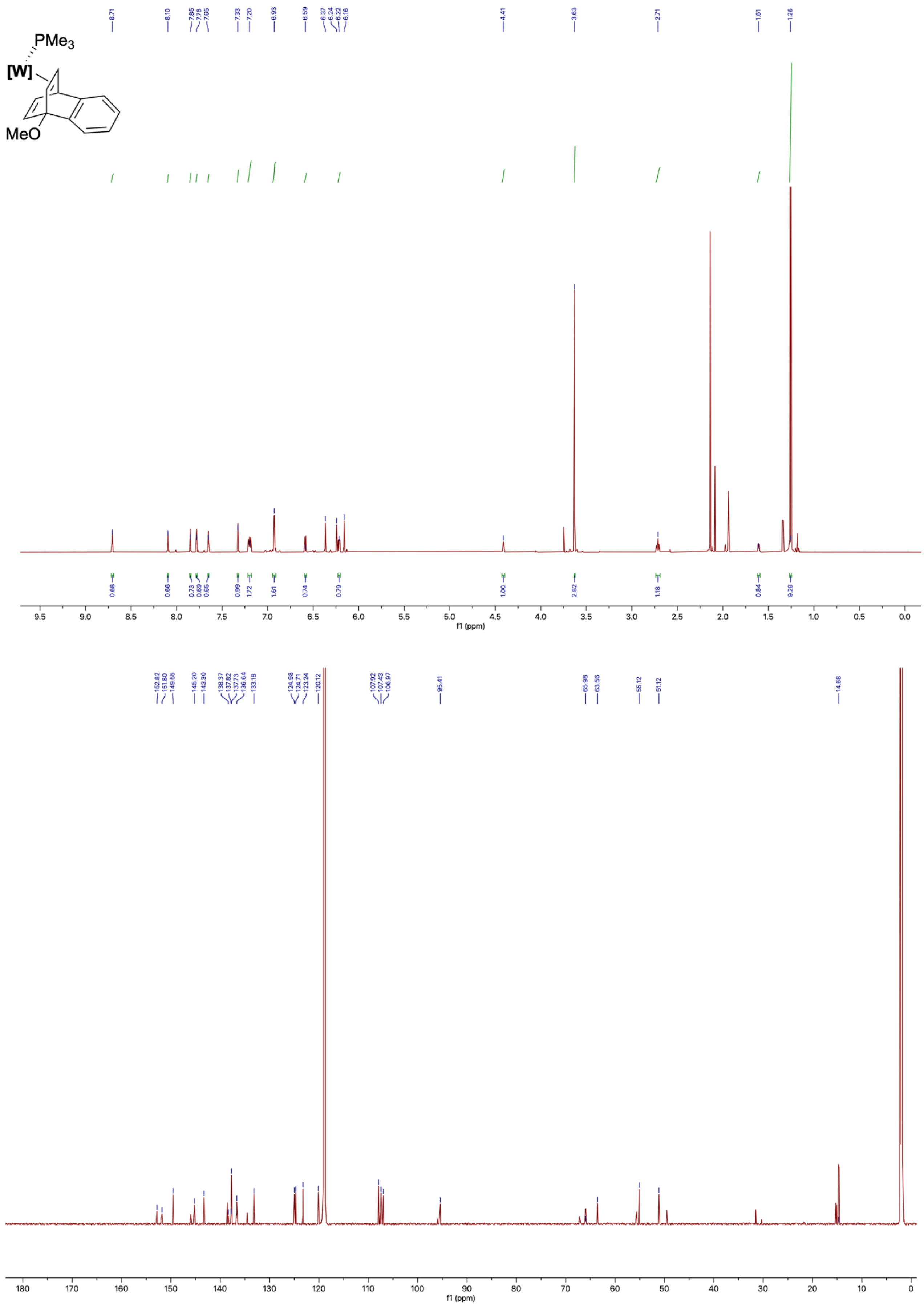

Fig. S8. Compound **7d** ^{1}H NMR (800 MHz, CD_3CN, 25 °C, top) and ^{13}C NMR (201 MHz, CD_3CN, 25 °C, bottom).

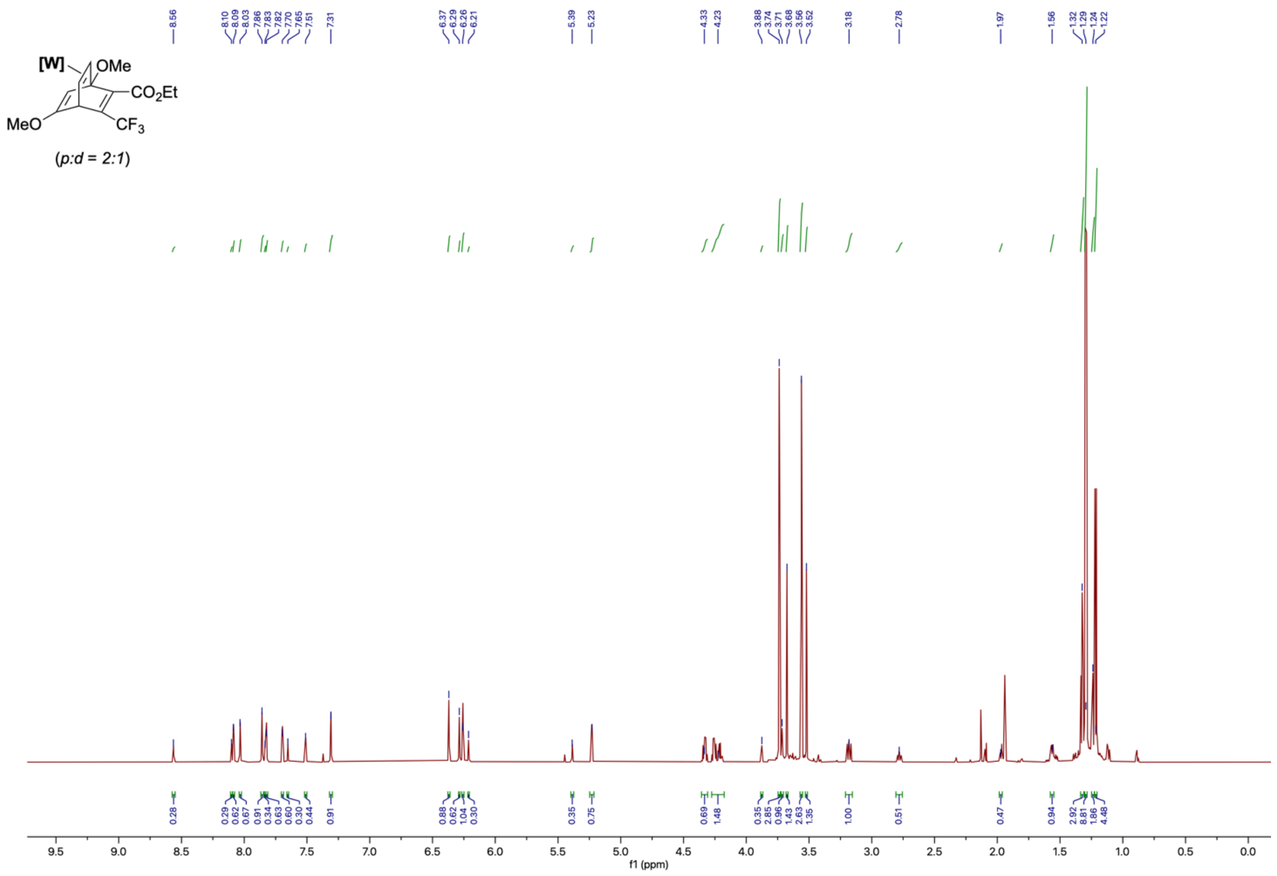

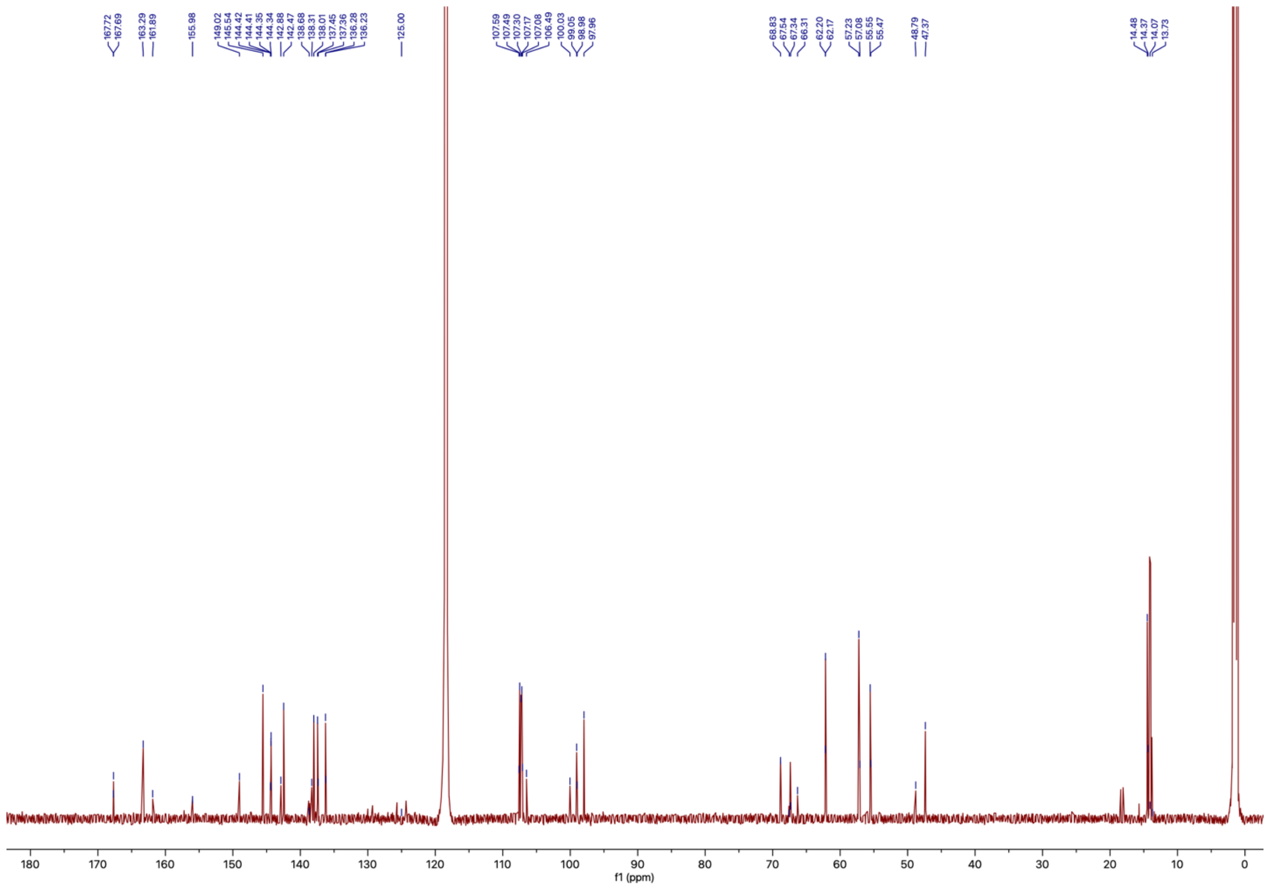

Fig. **S9.** Compound **8** ^{1}H NMR (800 MHz, CD_3CN, 25 °C, top) and ^{13}C NMR (201 MHz, CD_3CN, 25 °C, bottom).

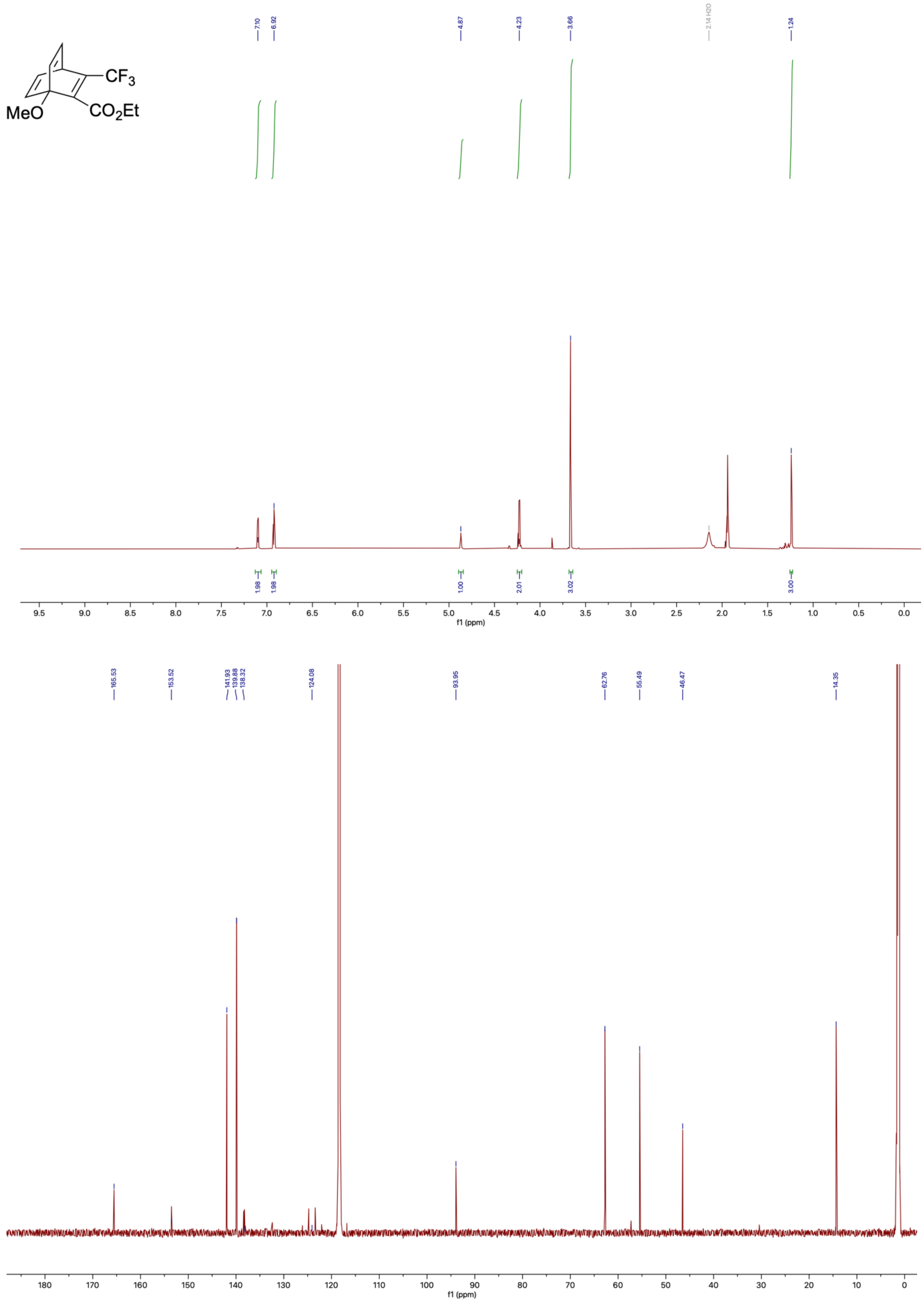

Fig. **S10.** Compound **10** ^{1}H NMR (800 MHz, CD_3CN, 25 °C, top) and ^{13}C NMR (201 MHz, CD_3CN, 25 °C, bottom).

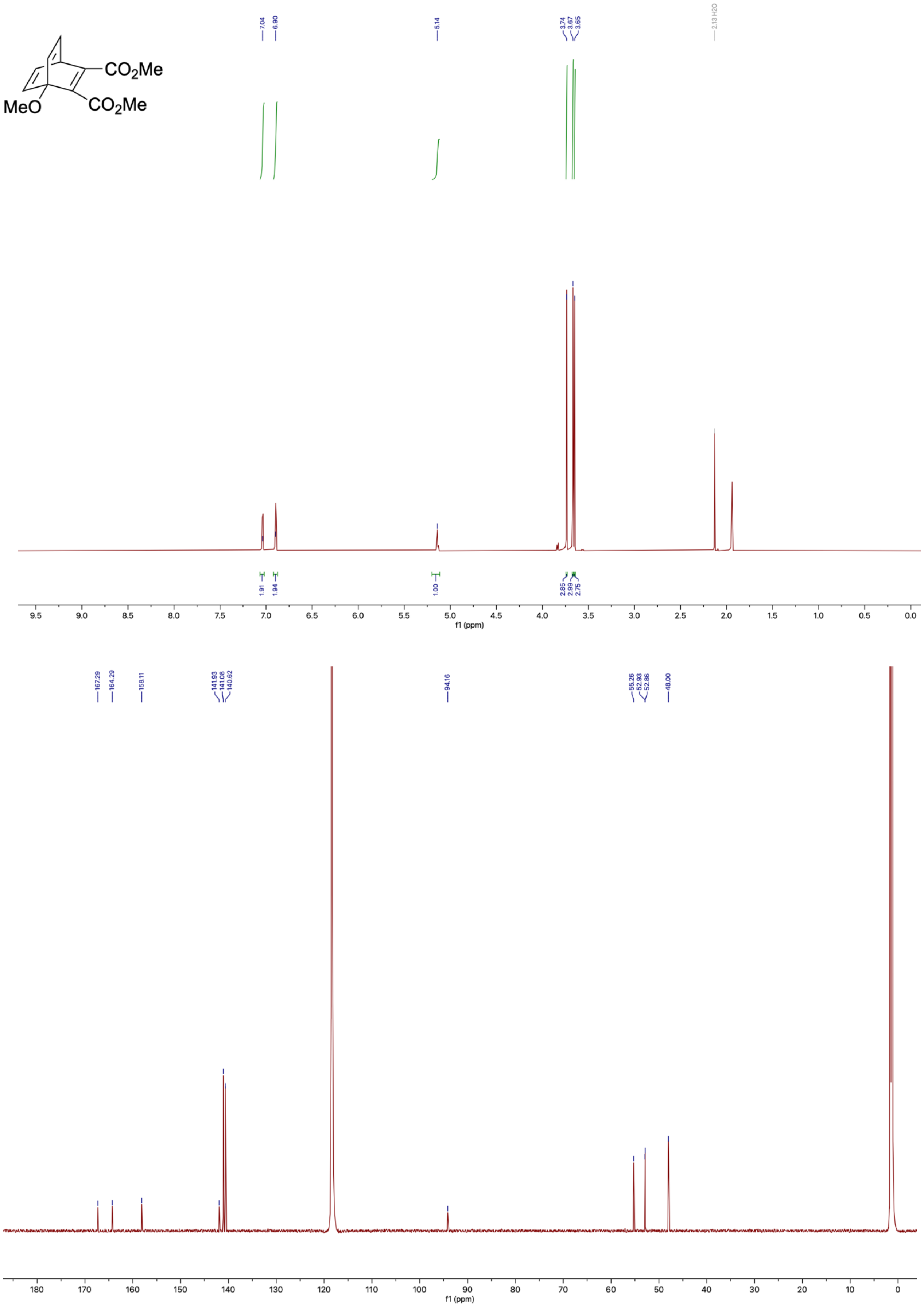

Fig. **S11.** Compound **11** ^{1}H NMR (800 MHz, CD_3CN, 25 °C, top) and ^{13}C NMR (201 MHz, CD_3CN, 25 °C, bottom).

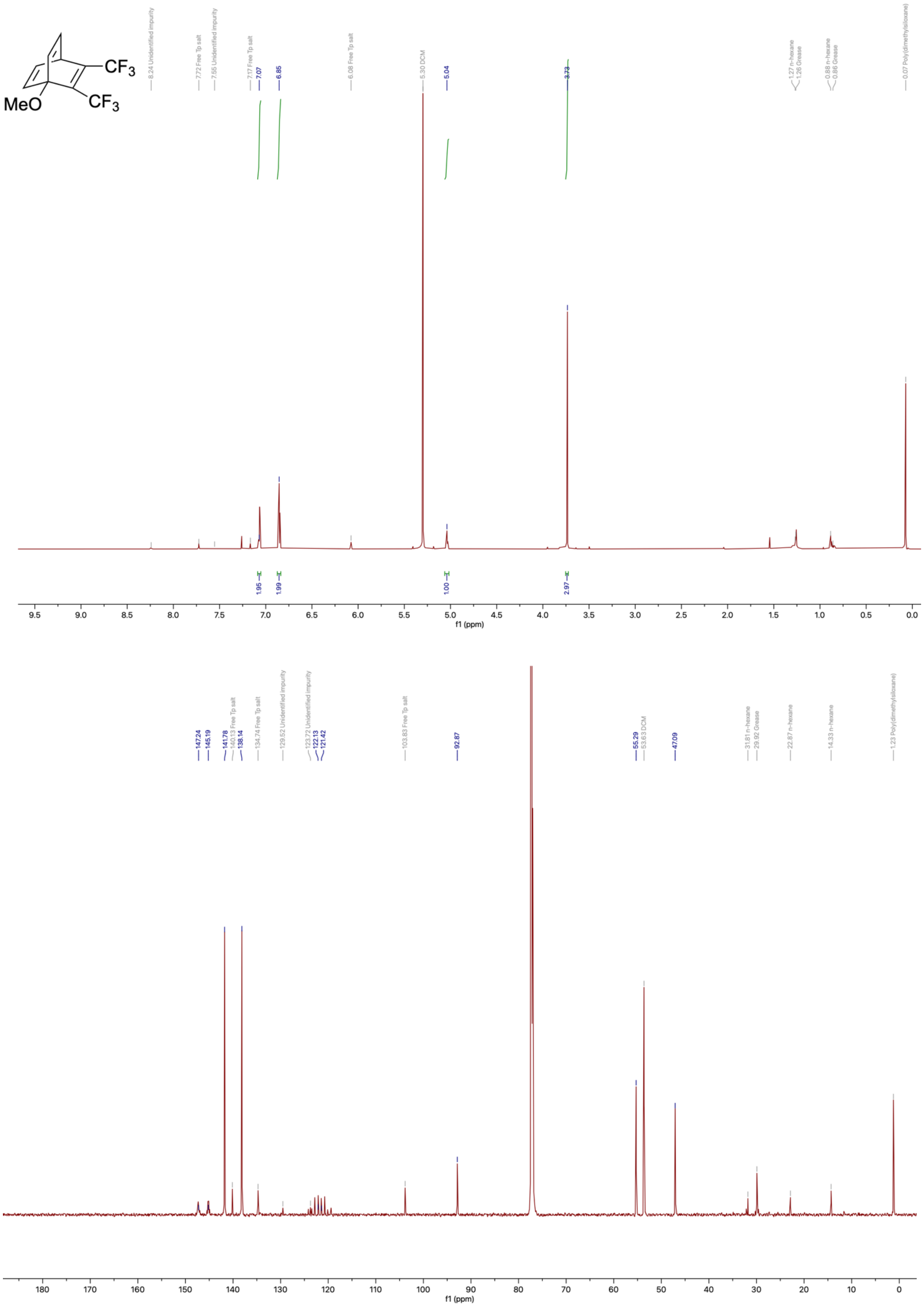

Fig. S12. Compound **12** ^{1}H NMR (**NMR1**) (800 MHz, $CDCl_3$, 25 °C, top) and ^{13}C NMR (201 MHz, $CDCl_3$, 25 °C, bottom).

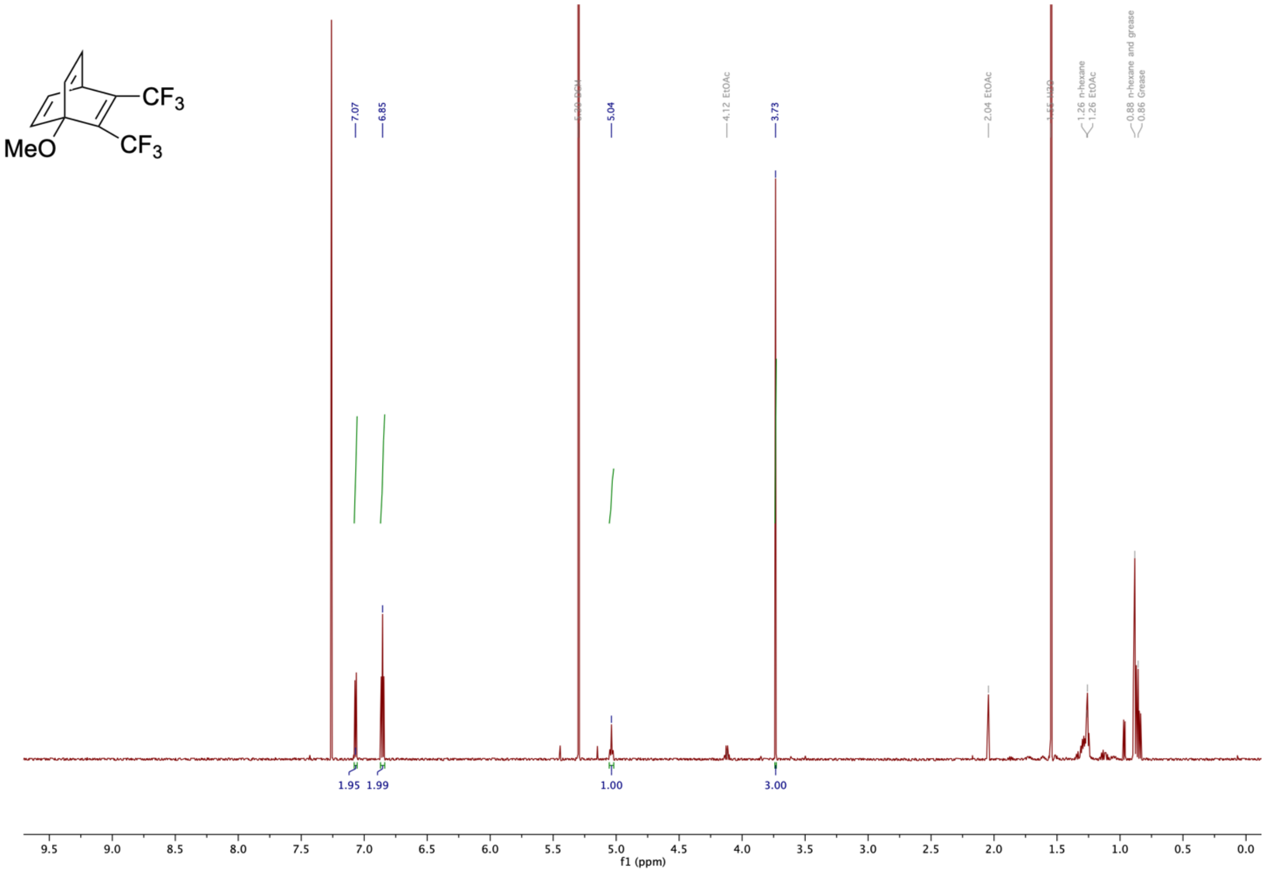

Fig. **S13.** Compound **12** ^{1}H NMR after crude vacuum distillation (**NMR2**) (600 MHz, $CDCl_3$, 25 °C, top).

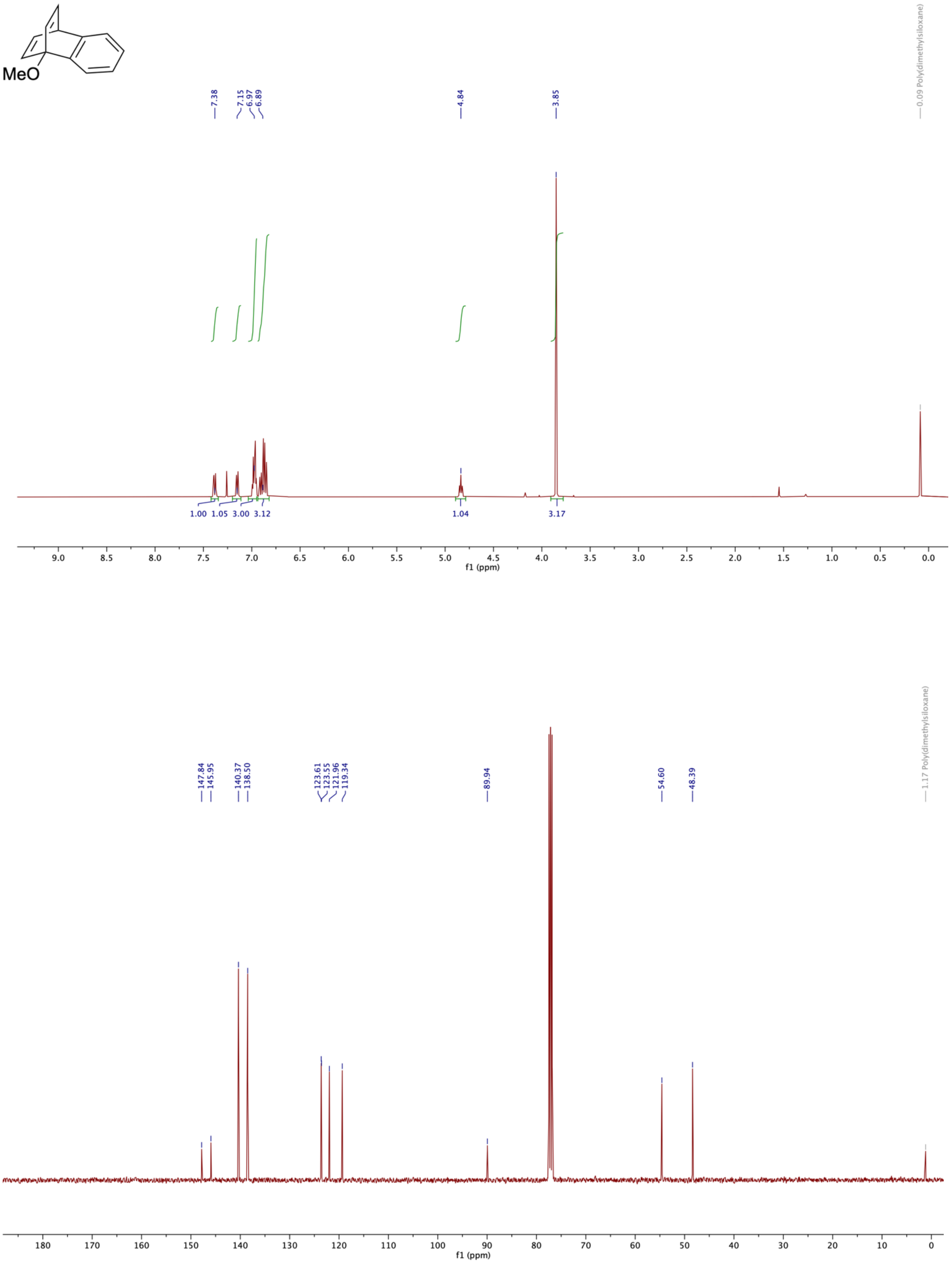

Fig. S14. Compound **13** ^{1}H NMR (400 MHz, $CDCl_3$, 25 °C, top) and ^{13}C NMR (101 MHz, $CDCl_3$, 25 °C, bottom).

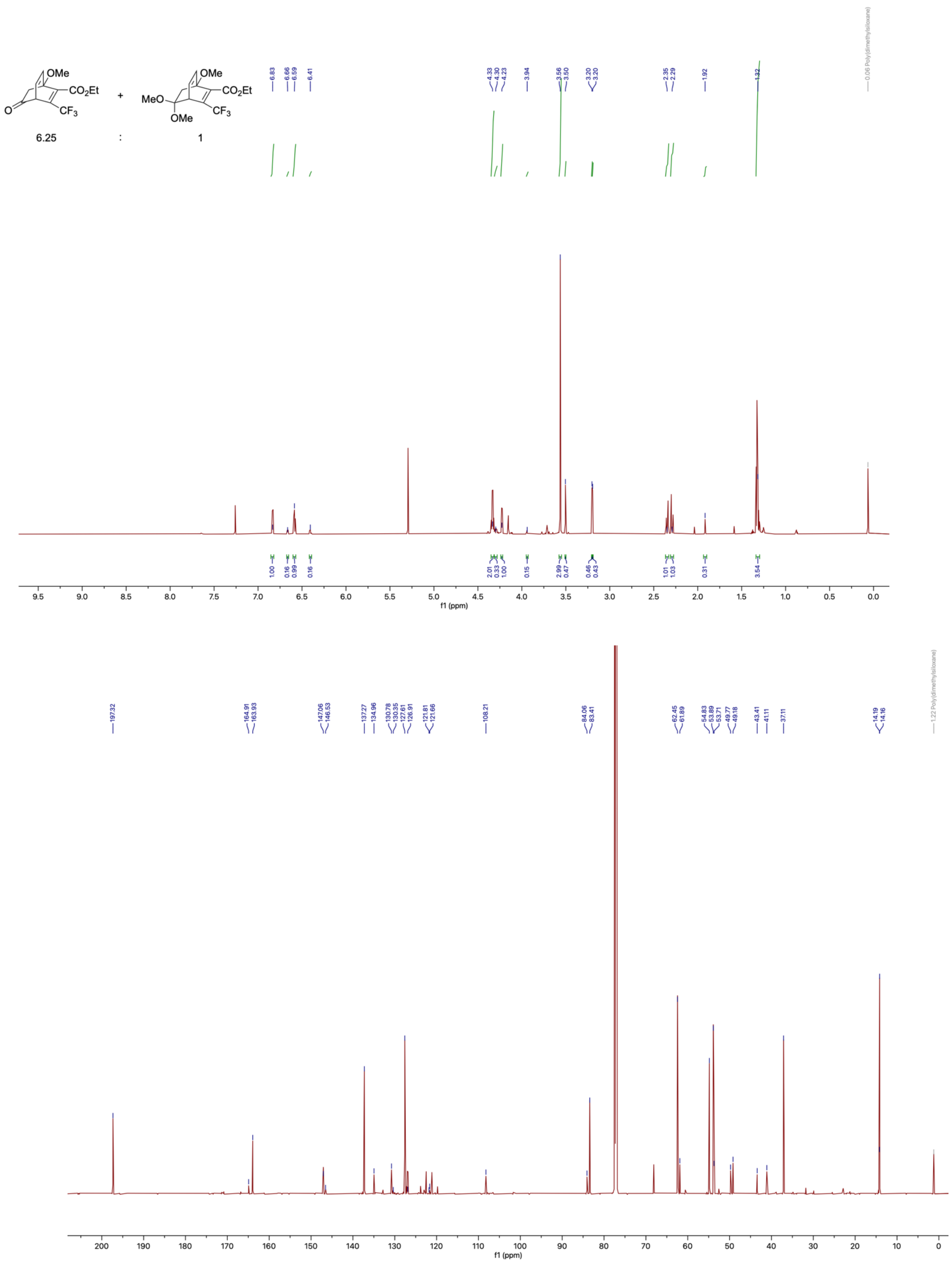

Fig. S15. Compound **14** ^{1}H NMR (800 MHz, $CDCl_3$, 25 °C, top) and ^{13}C NMR (201 MHz, $CDCl_3$, 25 °C, bottom).

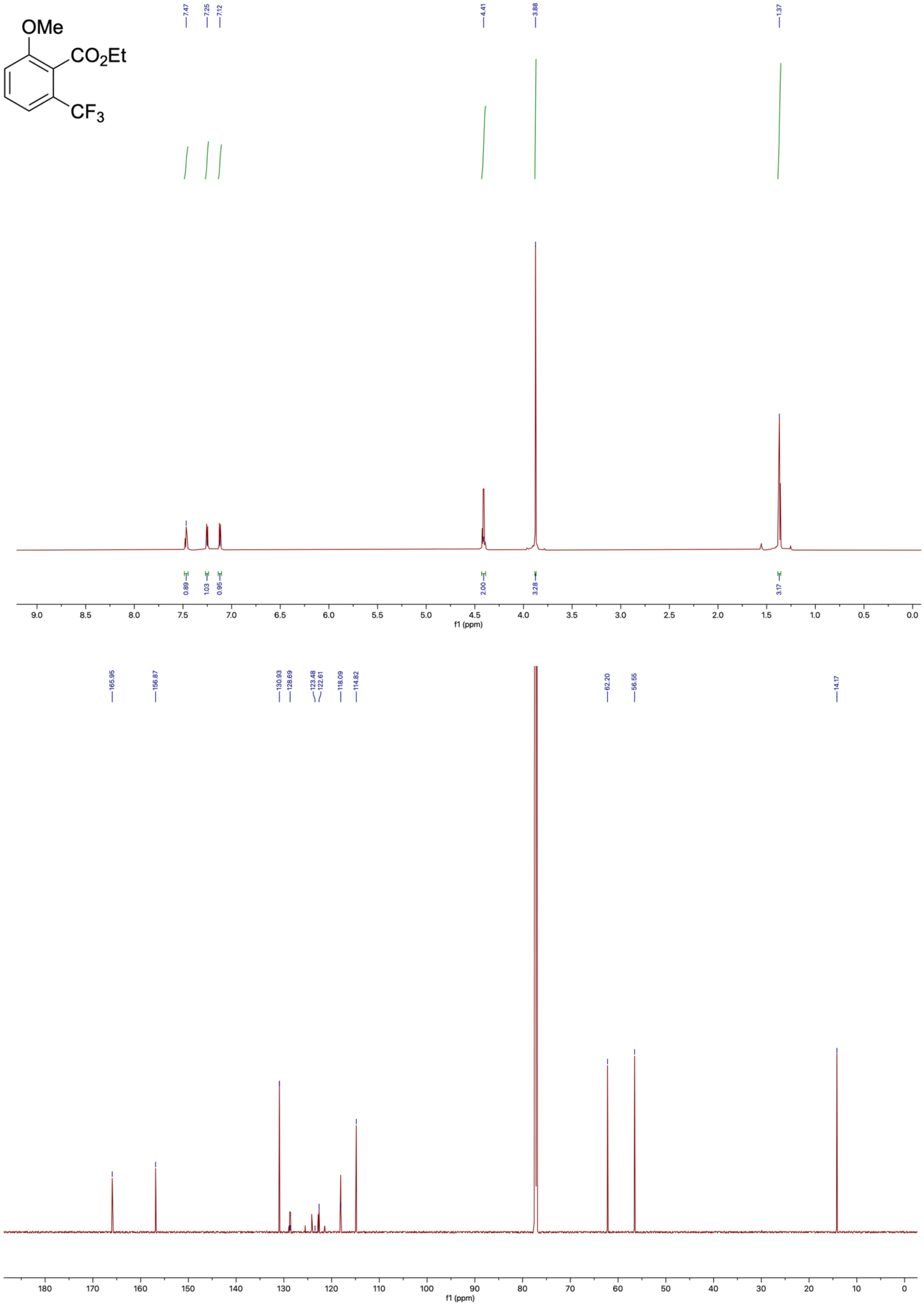

Fig. **S16.** Compound **15** ^{1}H NMR (800 MHz, $CDCl_3$, 25 °C, top) and ^{13}C NMR (201 MHz, $CDCl_3$, 25 °C, bottom).

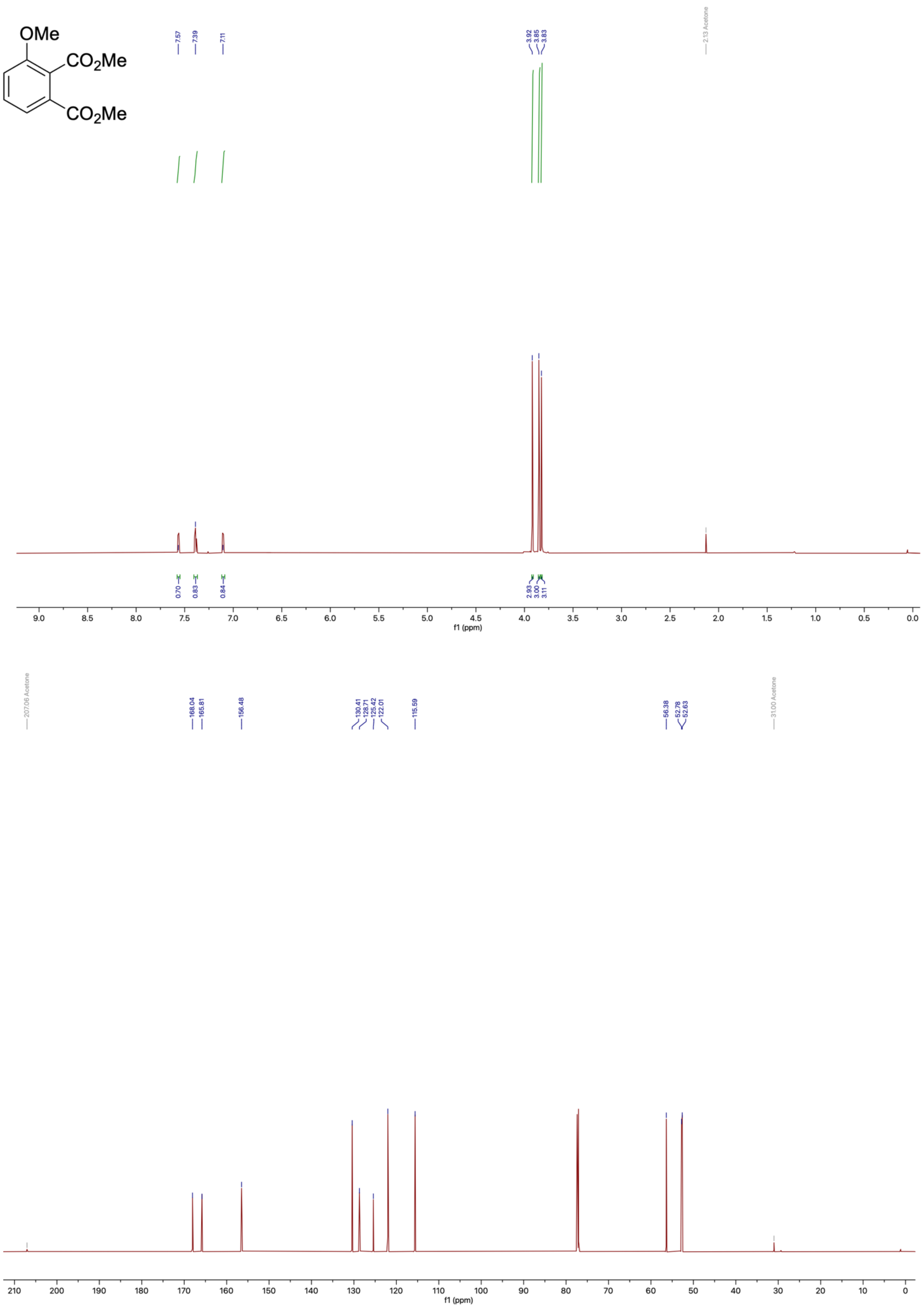

Fig. **S17.** Compound **16** ^{1}H NMR (800 MHz, $CDCl_3$, 25 °C, top) and ^{13}C NMR (201 MHz, $CDCl_3$, 25 °C, bottom).

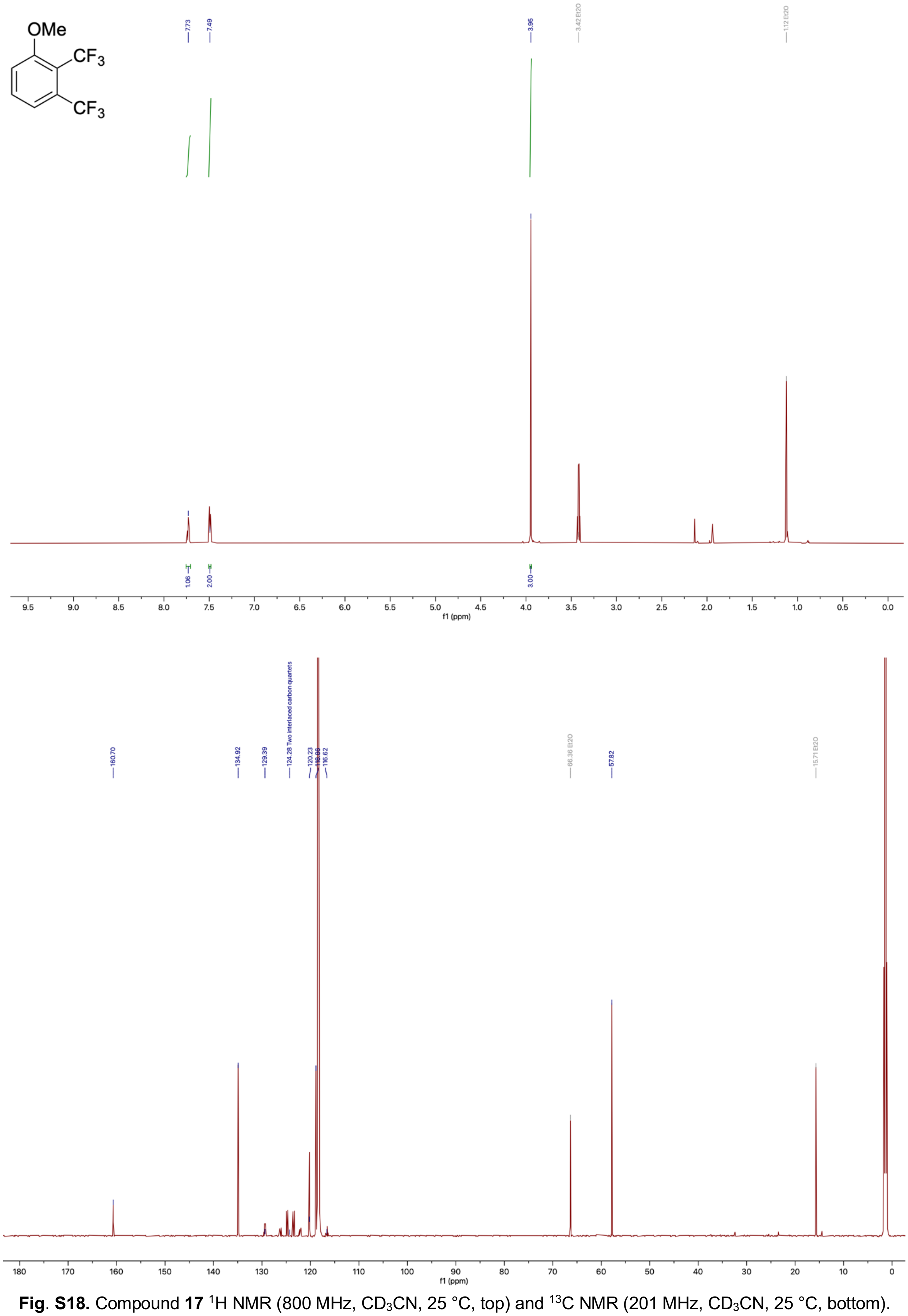

Fig. S18. Compound **17** ^{1}H NMR (800 MHz, CD_3CN, 25 °C, top) and ^{13}C NMR (201 MHz, CD_3CN, 25 °C, bottom).

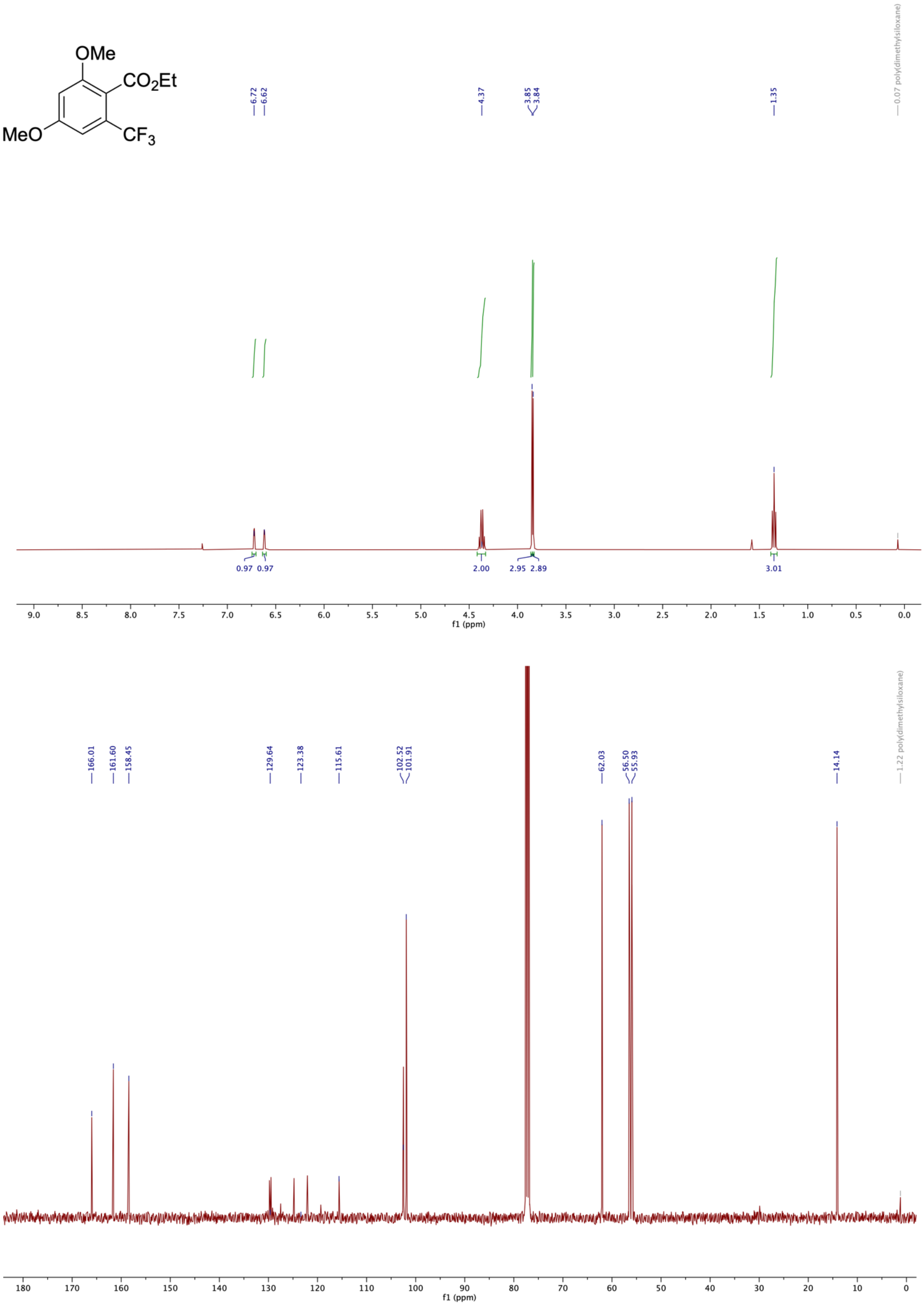

Fig. **S19.** Compound **18** ^{1}H NMR (400 MHz, $CDCl_3$, 25 °C, top) and ^{13}C NMR (101 MHz, $CDCl_3$, 25 °C, bottom).

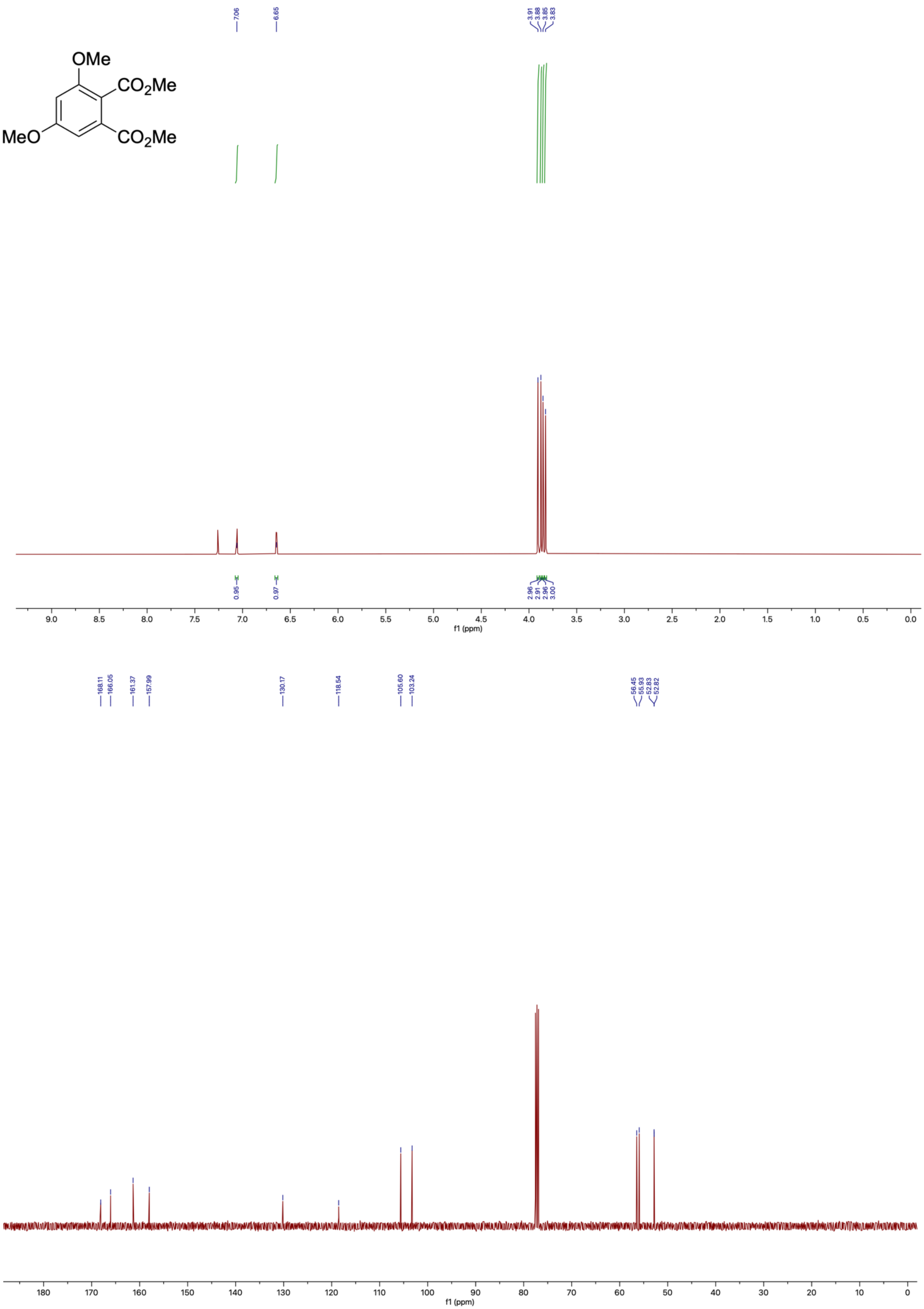

Fig. **S20.** Compound **19** ^{1}H NMR (400 MHz, $CDCl_3$, 25 °C, top) and ^{13}C NMR (101 MHz, $CDCl_3$, 25 °C, bottom).

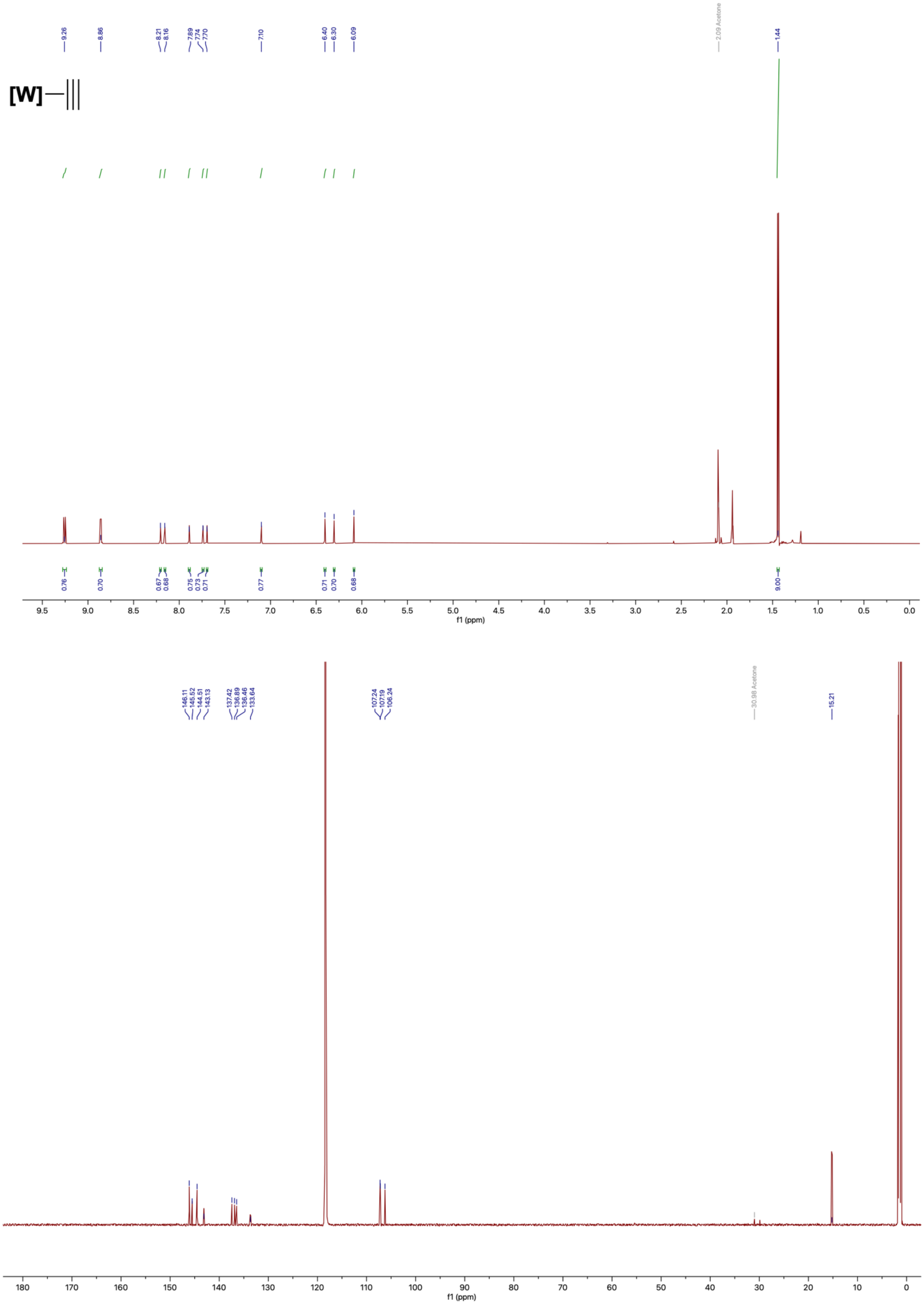

Fig. **S21.** Compound **20** ^{1}H NMR (800 MHz, CD_3CN, 25 °C, top) and ^{13}C NMR (201 MHz, CD_3CN, 25 °C, bottom).

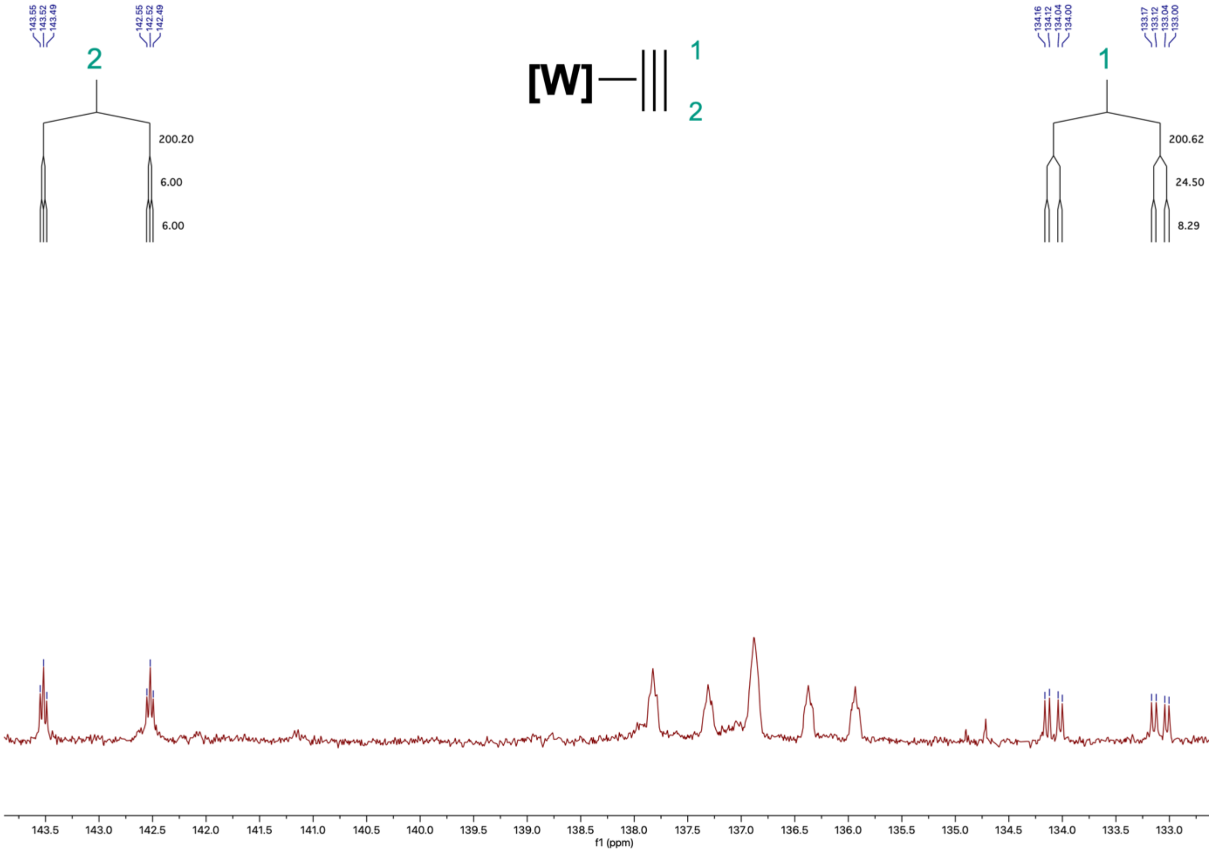

Fig. **S22.** Proton-coupled ^{13}C NMR (201 MHz, CD_3CN, 25 °C) of compound **20** showing coupling constants for the η^2-acetylene ligand in hertz.

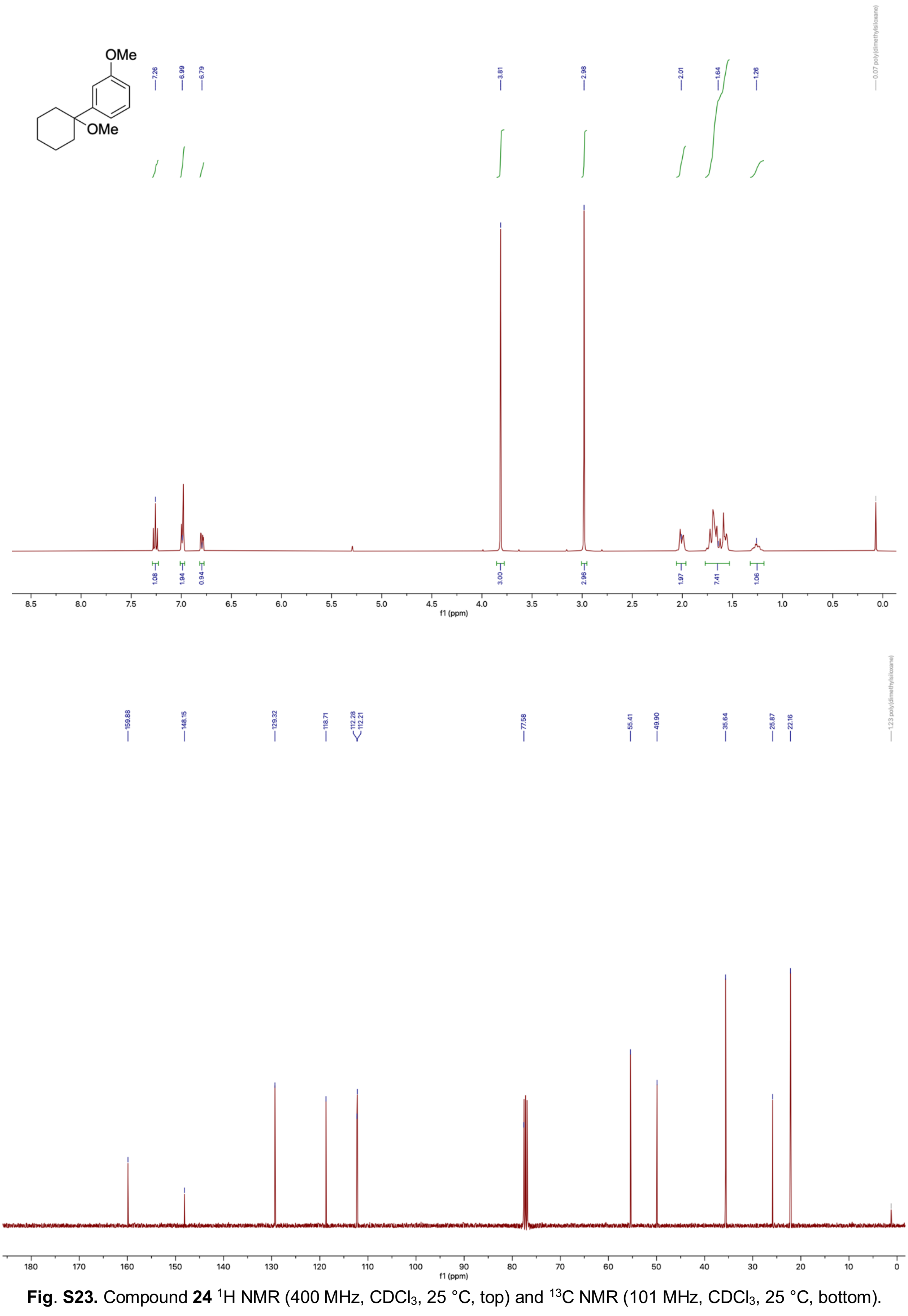

Fig. **S23.** Compound **24** ^{1}H NMR (400 MHz, $CDCl_3$, 25 °C, top) and ^{13}C NMR (101 MHz, $CDCl_3$, 25 °C, bottom).

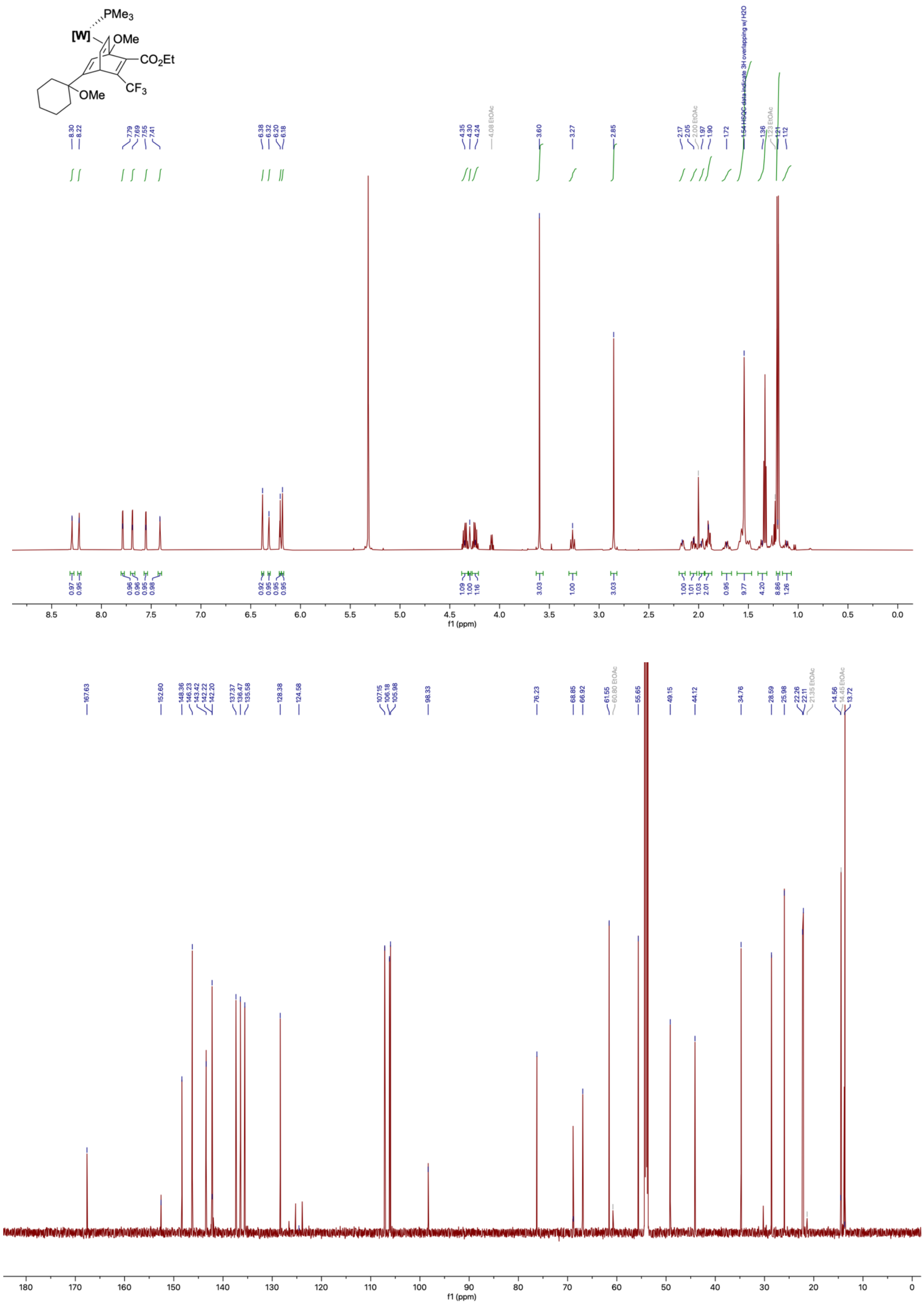

Fig. S24. Compound **21** ^{1}H NMR (600 MHz, CD_2C_2, 25 °C, top) and ^{13}C NMR (201 MHz, CD_2Cl_2, 25 °C, bottom).

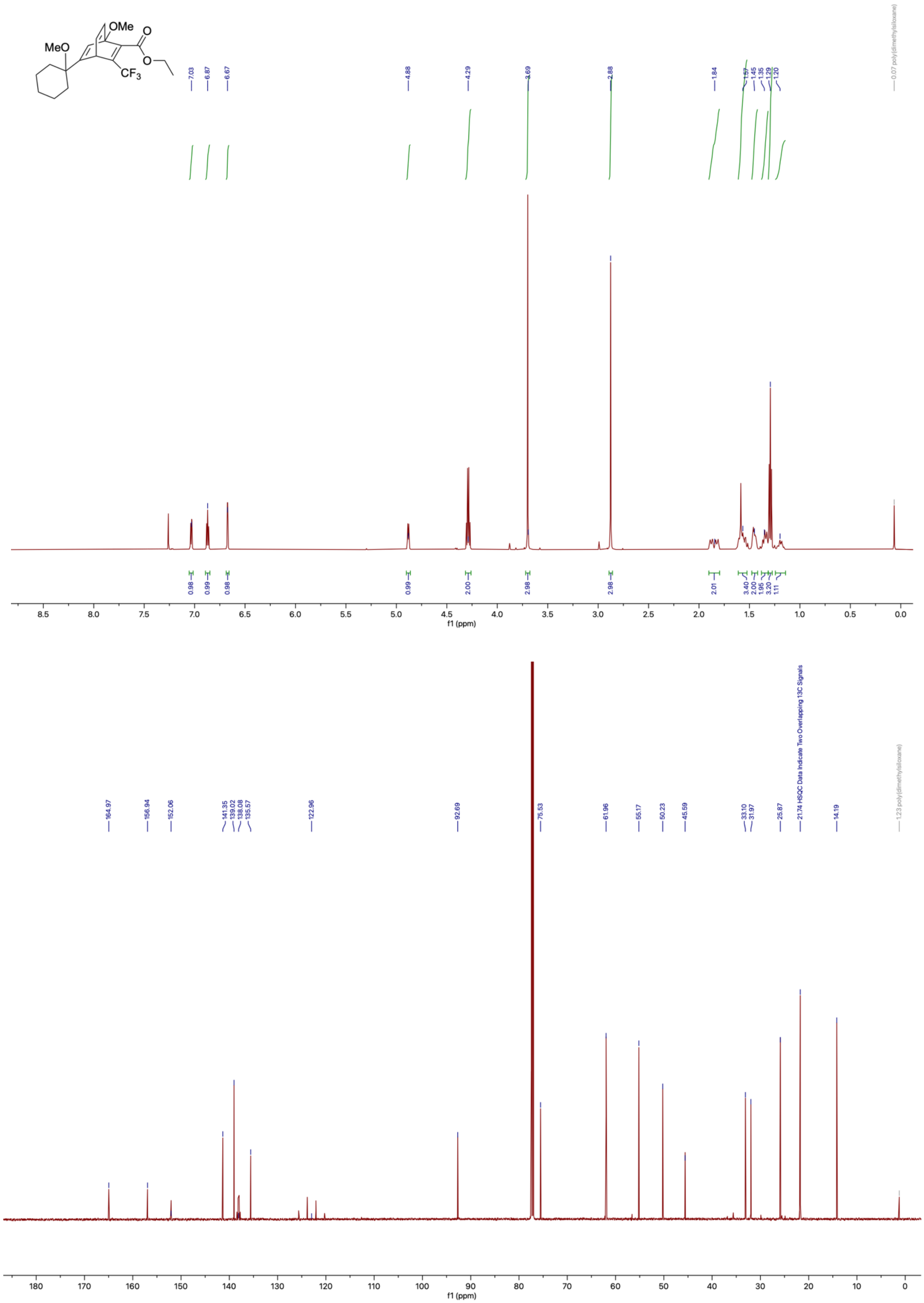

Fig. S25. Compound **22** ^{1}H NMR (600 MHz, $CDCl_3$, 25 °C, top) and ^{13}C NMR (151 MHz, $CDCl_3$, 25 °C, bottom).

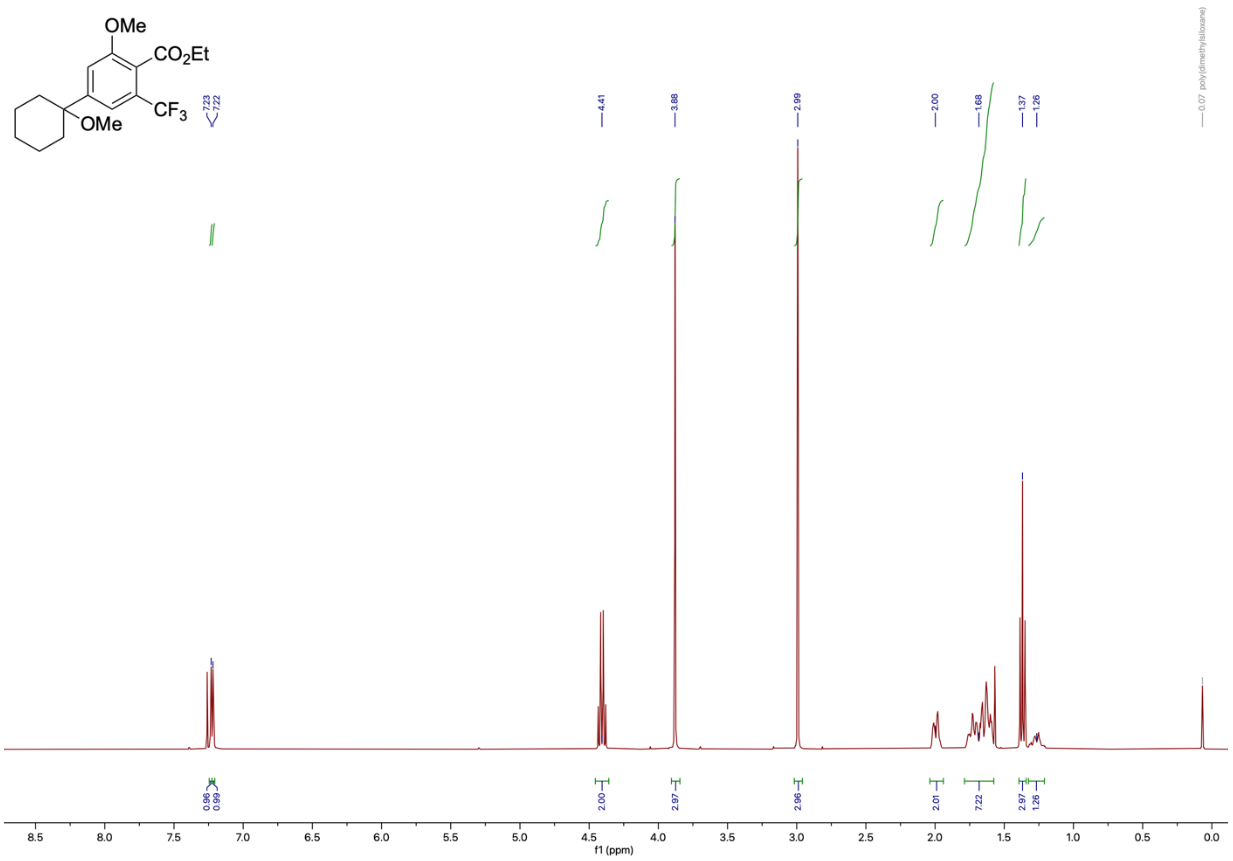

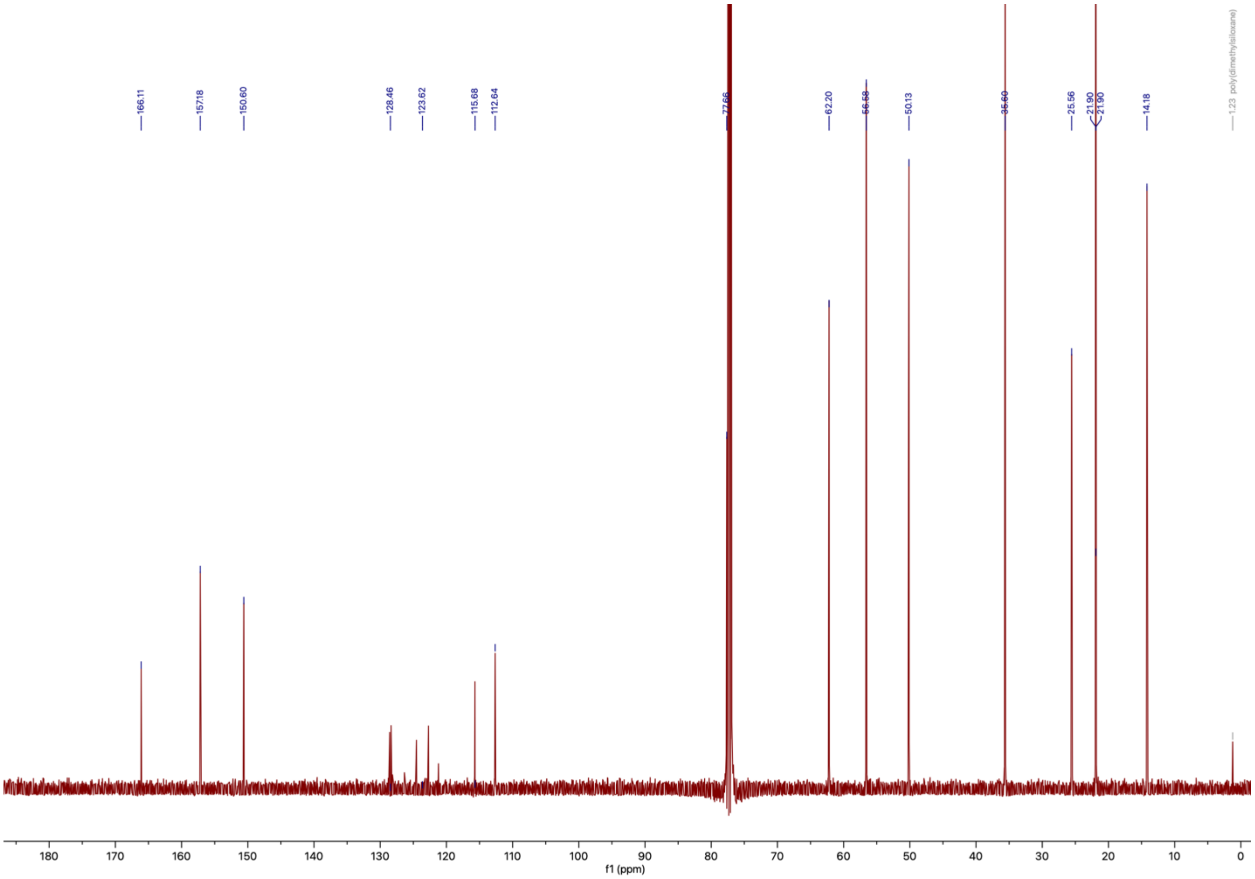

Fig. **S26.** Compound **23** ^{1}H NMR (400 MHz, $CDCl_3$, 25 °C, top) and ^{13}C NMR (151 MHz, $CDCl_3$, 25 °C, bottom).

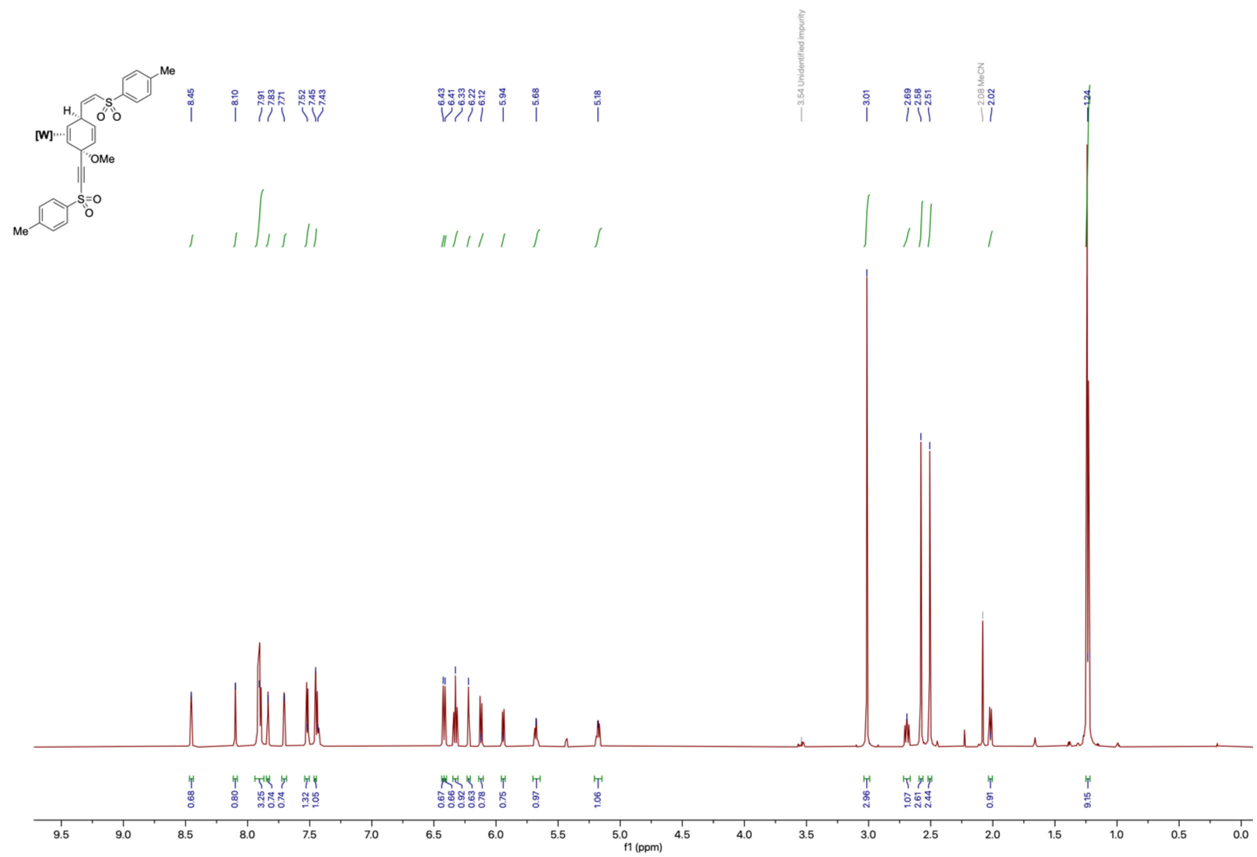

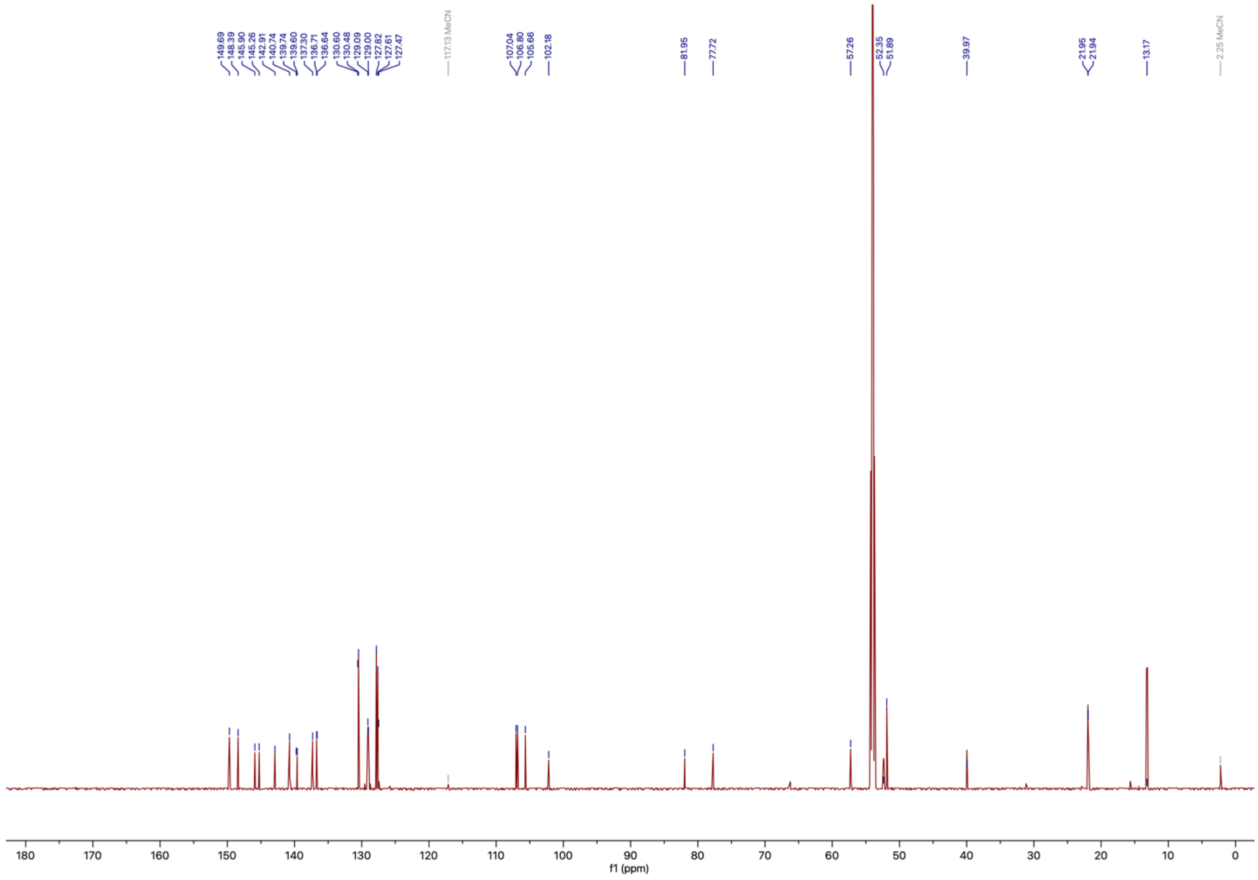

Fig. **S27.** Compound **S1** ^{1}H NMR (800 MHz, CD_2Cl_2, 25 °C, top) and ^{13}C NMR (201 MHz, CD_2Cl_2, 25 °C, bottom).

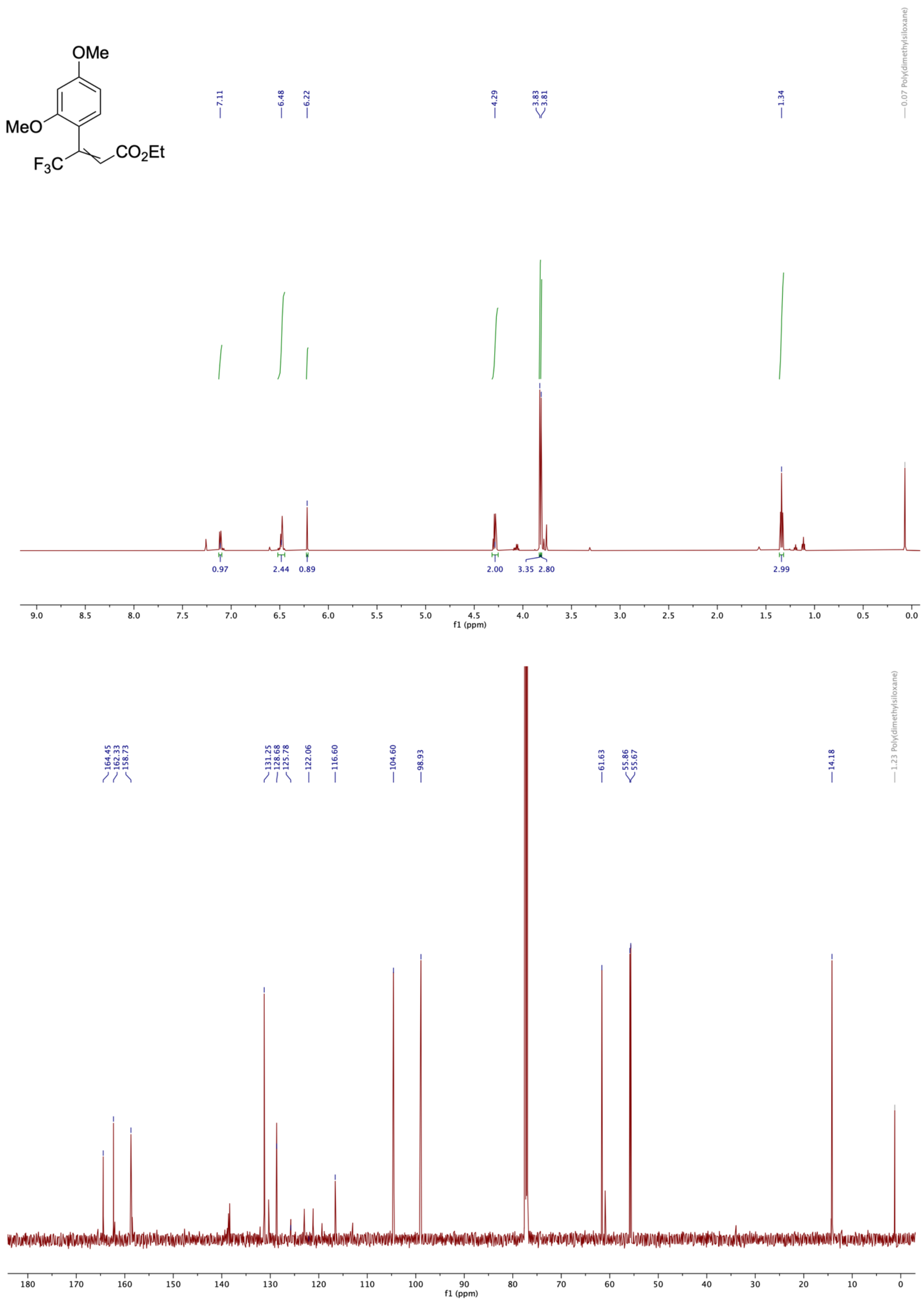

Fig. **S28.** Compound **S2** ^{1}H NMR (600 MHz, $CDCl_3$, 25 °C, top) and ^{13}C NMR (151 MHz, $CDCl_3$, 25 °C, bottom).

CRYSTALLOGRAPHIC DATA

Single crystals of **4-*d*, 5-*d*, 6-*d*, 7-*p*, 7-*d*, 8-*d*, 10, 11, 16, 19** – **22,** and **S1** were coated with Paratone oil and mounted on a MiTeGen micromount. Data were collected on a Bruker D8 VENTURE dual wavelength Mo/Cu Kappa four-circle diffractometer with a PHOTON III_C14 detector. The diffractometer was equipped with either an Oxford Cryostream 800 or 800Plus low temperature device. Data for **7-*p*** and **19** were collected using Cu K_α radiation (λ = 1.54178Å) from an Incoatec IµS 3.0 microfocus sealed tube with a HELIOS EF double bounce multilayer mirror as monochromator. Data for all other crystals were collected using Mo K_α radiation (λ = 0.71073Å) from an Incoatec IµS 3.0 microfocus sealed tube with a HELIOS double bounce multilayer mirror as monochromator. Data collection and processing for all structures were done within the Bruker APEX5 or APEX6 software suite.[1] All data were integrated with SAINT using a narrow-frame algorithm and a Multi-Scan absorption correction using SADABS was applied.[2] Using Olex2 as a graphical interface, [3] each structure was solved by dual methods with SHELXT[4] and refined by full-matrix least-squares methods against F^2 using SHELXL.[5] All non-hydrogen atoms were refined with anisotropic displacement parameters. For **4-*d***, **5-*d***, **7-*d*, 8-*d*, 20, 21** and **S1** the B-H hydrogen atoms were located in the electron density map and refined isotropically. For **4-*d***, **5-*d***, **7-*d*, 8-*d*** and **21** this was also done for the hydrogens on the carbons bound directly to W. All other hydrogen atoms in all structures except for those of **11** (see below) were placed in calculated positions using a riding model with their U_{iso} values constrained to 1.5 times the U_{eq} of their pivot atoms for terminal sp^3 carbon atoms and 1.2 times for all other carbon atoms. Most CIF files were generated using FinalCif.[6]

For **5-*d*, 6-*d***, **8-*d*, 19, 20** disordered moieties were refined using a combination of constraints and restraints on the bond lengths and anisotropic displacement parameters of the disordered atoms. For **6-*d***, the structure was refined as an inversion twin, with the BASF parameter converging at 0.22699. For **7-*p*** and **S1**, severely disordered solvent located in the crystal lattice could not be adequately modeled with or without restraints. Therefore, the solvent was accounted for using the Platon SQUEEZE method.[7] A void space of 2170 A^3 containing 604 electrons was found for **7-*p***, and a void space of 293 A^3 containing 51 electrons was found for **S1**. For **11**, the structure was refined with non-spherical atomic form factors using the NoSpherA2 routine of Olex2.[8] The ORCA 5.0 quantum mechanical package[9,10] was used for the non-spherical refinement. The basis set for the final refinement cycles was cc-pVTZ and the method was PBE.

---

[1] APEX5, APEX6, *SAINT*. Bruker AXS Inc., Madison, Wisconsin, USA.

[2] Krause, L.; Herbst-Irmer, R.; Sheldrick, G. M.; Stalke, D. Comparison of silver and molybdenum microfocus X-ray sources for single-crystal structure determination., *J. Appl. Cryst.* **2015**, *48*, 3–10, doi:10.1107/S1600576714022985.

[3] Dolomanov, O. V.; Bourhis, L. J.; Gildea, R. J.; Howard, J. A. K.; Puschmann, H. OLEX2: a complete structure solution, refinement and analysis program., *J. Appl. Cryst.* **2009**, *42*, 339-341, doi:10.1107/S0021889808042726.

[4] Sheldrick, G. M. SHELXT - integrated space-group and crystal-structure determination, *Acta Cryst.* **2015**, *A71*, 3–8, doi:10.1107/S2053273314026370.

[5] Sheldrick, G. M., Crystal structure refinement with SHELXL. *Acta Crystallogr. Sect C: Struct. Chem.* **2015**, *C71*, 3-8. doi: 10.1107/S2053229614024218

[6] Kratzert, D. FinalCif, https://dkratzert.de/finalcif.html.

[7] Spek, A. L., PLATON SQUEEZE: a tool for the calculation of the disordered solvent contribution to the calculated structure factors. *Acta Crystallogr. Sect C: Struct. Chem.* **2015**, *C71*, 9-18. doi: 10.1107/S2053229614024929

[8] Kleemiss, F.; Dolomanov, O. V.; Bodensteiner, M.; Peyerimhoff, N.; Midgley, L.; Bourhis, L. J.; Genoni, A.; Malaspina, L. A.; Jaytilaka, D.; Spencer, J. L.; White, F.; Grundkötter-Stock, B.; Steinhauer, S.; Lentz, D.; Puschmann, H.; Grabowsky, S., Accurate crystal structures and chemical properties from NoSpherA2. *Chem. Sci.* **2021**, *12*, 1675-1692. doi: 10.1039/D0SC05526C

[9] Neese,F.; Wennmohs,F.; Becker,U.; Riplinger,C., The ORCA quantum chemistry program package. *J. Chem. Phys.* **2020,** *152*, 224108. doi: 10.1063/5.0004608

[10] Neese, F., Software update: The ORCA program system—Version 5.0. *WIREs Comp. Mol. Sci.* **2022**, *12*, e1606. doi: 10.1002/wcms.1606

Table **S5.** Crystallographic data for **4-*d*** – **7-*d***.

	**4-*d***	**5-*d***	**6-*d***	**7-*p***	**7-*d***
CCDC no.	2442051	2442052	2442053	2442054	2442055
Empirical formula	$C_{53}H_{70}B_2F_6N_{14}O_9P_2W_2$	$C_{25}H_{33}BN_7O_6PW$	$C_{23}H_{27}BF_6N_7O_2PW$	$C_{25}H_{31}BN_7O_2PW$	$C_{28}H_{37}BN_7O_3PW$
FW [g/mol]	1612.49	753.00	773.14	687.20	745.27
Temp. [K]	100.00	100.00	100.00	100.00	100.00
Cryst. Sys.	monoclinic	monoclinic	orthorhombic	orthorhombic	monoclinic
Space group	I2/a	$P2_1/n$	$Pna2_1$	Pccn	$P2_1/n$
a [Å]	15.5792(7)	10.3249(4)	15.6286(8)	13.6711(3)	11.2695(4)
b [Å]	12.1942(6)	14.4588(5)	12.4656(7)	18.0124(4)	20.3392(7)
c [Å]	33.387(2)	19.2070(8)	28.4311(18)	28.2777(8)	14.0263(4)
α [°]	90	90	90	90	90
β [°]	93.471(2)	90.2330(10)	90	90	111.9320(10)
γ [°]	90	90	90	90	90
Volume [Å^3]	6331.1(6)	2867.31(19)	5538.9(5)	6963.4(3)	2982.33(17)
Z	4	4	8	8	4
ρ_{calc} [g/cm^3]	1.692	1.744	1.854	1.311	1.660
μ [mm^{-1}]	3.763	4.137	4.305	6.814	3.970
F(000)	3200	1496	3024	2720	1488
Crystal size [mm^3]	0.019 x 0.031 x 0.040	0.095×0.208×0.227	0.07×0.072×0.107	0.059×0.101×0.128	0.064×0.105×0.169
Crystal habit	Yellow plate	Colourless plate	Yellow plate	Colourless plate	Yellow plate
λ [Å]	0.71073	0.71073	0.71073	1.54178	0.71073
2θ range [°]	3.56 to 54.94	4.47 to 56.54	4.42 to 50.74	7.95 to 137.13	3.98 to 66.32
Index ranges	$-17 \le h \le 20$<br>$-15 \le k \le 15$<br>$-43 \le l \le 35$	$-13 \le h \le 13$<br>$-19 \le k \le 18$<br>$-25 \le l \le 25$	$-18 \le h \le 18$<br>$-14 \le k \le 15$<br>$-34 \le l \le 34$	$-16 \le h \le 16$<br>$-18 \le k \le 21$<br>$-19 \le l \le 34$	$-16 \le h \le 17$<br>$-31 \le k \le 31$<br>$-21 \le l \le 16$
Reflections collected	53665	46935	77014	30320	93863
Independent reflections	7252 [R_{int} = 0.1037]	7094 [R_{int} = 0.0674]	10136 [R_{int} = 0.12493]	6401 [R_{int} = 0.0726]	11367 [R_{int} = 0.0371]
Data / Restraints / Parameters	7252 / 0 / 414	7094 / 9 / 402	10136 / 443 / 707	6401 / 0 / 347	11367 / 0 / 387
Goodness-of-fit on F^2	1.011	1.048	1.038	1.164	1.038
Final *R* indexes [$I \geq 2\sigma(I)$]	R1 = 0.0420, wR2 = 0.0839	R_1 = 0.0382 wR_2 = 0.0773	R_1 = 0.0560 wR_2 = 0.1210	R_1 = 0.0858 wR_2 = 0.1951	R_1 = 0.0186 wR_2 = 0.0364
Final *R* indexes [all data]	R1 = 0.0736, wR2 = 0.0943	R_1 = 0.0580 wR_2 = 0.0842	R_1 = 0.0907 wR_2 = 0.1380	R_1 = 0.1003 wR_2 = 0.2014	R_1 = 0.0229 wR_2 = 0.0376

Table **S6.** Crystallographic data for **8-*d***, **10, 11** and **16**.

	**8-*d***	**10**	**11**	**16**
CCDC no.	2442056	2465560	2442057	2442047
Empirical formula	$C_{26}H_{34.67}BF_3N_7O_{5.34}PW$	$C_{13}H_{13}F_3O_3$	$C_{13}H_{14}O_5$	$C_{11}H_{12}O_5$
FW [g/mol]	813.27	274.23	250.253	224.21
Temp. [K]	100.00	100.00	100.00	100.00
Crystal system	monoclinic	monoclinic	monoclinic	monoclinic
Space group	$P2_1/n$	$P2_1/n$	$P2_1/c$	$P2_1/c$
a [Å]	7.9086(2)	12.6047(5)	13.5236(10)	7.5367(4)
b [Å]	20.9439(5)	7.9513(3)	6.3185(4)	20.3927(13)
c [Å]	19.3270(6)	12.9673(6)	13.8437(12)	7.2440(3)
α [°]	90	90	90	90
β [°]	98.4460(10)	101.3540(10)	94.796(3)	101.821(2)

γ [°]	90	90	90	90
Volume [Å^3]	3166.55(15)	1274.20(9)	1178.79(15)	1089.74(10)
Z	4	4	4	4
ρ_{calc} [g/cm^3]	1.706	1.430	1.410	1.367
μ [mm^{-1}]	3.765	0.129	0.109	0.109
F(000)	1613	568	528.410	472
Crystal size [mm^3]	0.06×0.116×0.141	0.214×0.388×0.58	0.045×0.05×0.197	0.066×0.158×0.418
Crystal habit	Brown plate	Colourless plate	Colourless plate	Colourless block
λ [Å]	0.71073	0.71073	0.71073	0.71073
2θ range [°]	3.89 to 61.03	5.03 to 62.97	5.90 to 51.36	3.99 to 56.56
Index ranges	−11 ≤ h ≤ 11<br>−29 ≤ k ≤ 28<br>−27 ≤ l ≤ 27	−18 ≤ h ≤ 17<br>−11 ≤ k ≤ 10<br>−17 ≤ l ≤ 19	−16 ≤ h ≤ 16<br>−7 ≤ k ≤ 7<br>−16 ≤ l ≤ 14	−10 ≤ h ≤ 9<br>−27 ≤ k ≤ 27<br>−8 ≤ l ≤ 9
Reflections collected	69849	18591	18761	22297
Independent reflections	9673<br>[R_{int} = 0.0589]	4074<br>[R_{int} = 0.0362]	2243<br>[R_{int} = 0.0684]	2691<br>[R_{int} = 0.0625]
Data / Restraints / Parameters	9673 / 2 / 458	4074 / 0 / 174	2243 / 0 / 289	2691 / 0 / 148
Goodness-of-fit on F^2	1.033	1.052	1.0658	1.074
Final *R* indexes [I≥2σ(I)]	R_1 = 0.0272<br>wR_2 = 0.0560	R_1 = 0.0400<br>wR_2 = 0.1088	R_1 = 0.0266<br>wR_2 = 0.0526	R_1 = 0.0405<br>wR_2 = 0.1048
Final *R* indexes [all data]	R_1 = 0.0401<br>wR_2 = 0.0602	R_1 = 0.0472<br>wR_2 = 0.1143	R_1 = 0.0463<br>wR_2 = 0.0606	R_1 = 0.0571<br>wR_2 = 0.1163

Table **S7.** Crystallographic data for **19** – **22** and **S1**.

	**19**	**20**	**21**	**22**	**S1**
CCDC no.	2442048	2442049	2473411	2475758	2442050
Empirical formula	$C_{12}H_{14}O_6$	$C_{14}H_{21}BN_7OPW$	$C_{32}H_{44}BF_3N_7O_5PW$	$C_{20}H_{25}F_3O_4$	$C_{37}H_{43}BN_7O_6PS_2W$
FW [g/mol]	254.23	529.01	889.37	386.40	971.53
Temp. [K]	100.00	100.00	100.00	100.00	100.00
Crystal system	monoclinic	monoclinic	monoclinic	triclinic	monoclinic
Space group	P2$_1$/n	P2$_1$/n	P2$_1$/n	P -1	P2$_1$/c
a [Å]	10.5219(6)	10.3863(7)	11.9541(6)	7.4673(4)	17.3263(11)
b [Å]	6.7785(5)	12.6484(6)	24.6319(13)	10.1420(5)	16.4249(9)
c [Å]	34.0881(19)	14.6203(9)	12.6663(8)	12.6913(7)	15.9827(9)
α [°]	90	90	90	92.592(2)	90
β [°]	97.065(4)	104.936(2)	100.141(2)	102.685(2)	113.062(2)
γ [°]	90	90	90	94.035(2)	90
Volume [Å^3]	2412.8(3)	1855.78(19)	3671.4(4)	933.55(9)	4184.9(4)
Z	8	4	4	2	4
ρ_{calc} [g/cm^3]	1.400	1.893	1.609	1.375	1.542
μ [mm^{-1}]	0.966	6.328	3.254	0.114	2.950
F(000)	1072	1024	1784	408	1952
Crystal size [mm^3]	0.035×0.043×0.146	0.034×0.041×0.076	0.088×0.139×0.2	0.102×0.145×0.458	0.037×0.058×0.127
Crystal habit	Colourless needle	Brown plate	Colourless plate	Colourless plate	Colourless needle
λ [Å]	1.54178	0.71073	0.71073	0.71073	0.71073
2θ range [°]	5.22 to 136.85	4.32 to 51.37	3.84 to 56.61	5.05 to 61.05	4.96 to 50.80
Index ranges	−12 ≤ h ≤ 12<br>−8 ≤ k ≤ 6<br>−40 ≤ l ≤ 40	−12 ≤ h ≤ 12<br>−15 ≤ k ≤ 13<br>−17 ≤ l ≤ 17	−15 ≤ h ≤ 14<br>−32 ≤ k ≤ 32<br>−16 ≤ l ≤ 16	−10 ≤ h ≤ 10<br>−14 ≤ k ≤ 14<br>−18 ≤ l ≤ 18	−20 ≤ h ≤ 20<br>−19 ≤ k ≤ 17<br>−19 ≤ l ≤ 19

Reflections collected	18135	29511	71196	39702	52265
Independent reflections	4382 [R_{int} = 0.1518]	3522 [R_{int} = 0.0935]	9095 [R_{int} = 0.0720]	5649 [R_{int} = 0.0271]	7694 [R_{int} = 0.1286]
Data / Restraints / Parameters	4382 / 14 / 348	3522 / 32 / 282	9095 / 0 / 468	5649 / 0 / 247	7694 / 0 / 510
Goodness-of-fit on F^2	0.960	1.017	1.020	1.025	0.999
Final *R* indexes [$I \geq 2\sigma(I)$]	R_1 = 0.0718<br>wR_2 = 0.1655	R_1 = 0.0344<br>wR_2 = 0.0682	R_1 = 0.0291<br>wR_2 = 0.0579	R_1 = 0.0355<br>wR_2 = 0.0965	R_1 = 0.0428<br>wR_2 = 0.0920
Final *R* indexes [all data]	R_1 = 0.1778<br>wR_2 = 0.2259	R_1 = 0.0627<br>wR_2 = 0.0779	R_1 = 0.0478<br>wR_2 = 0.0644	R_1 = 0.0407<br>wR_2 = 0.1004	R_1 = 0.0844<br>wR_2 = 0.1083

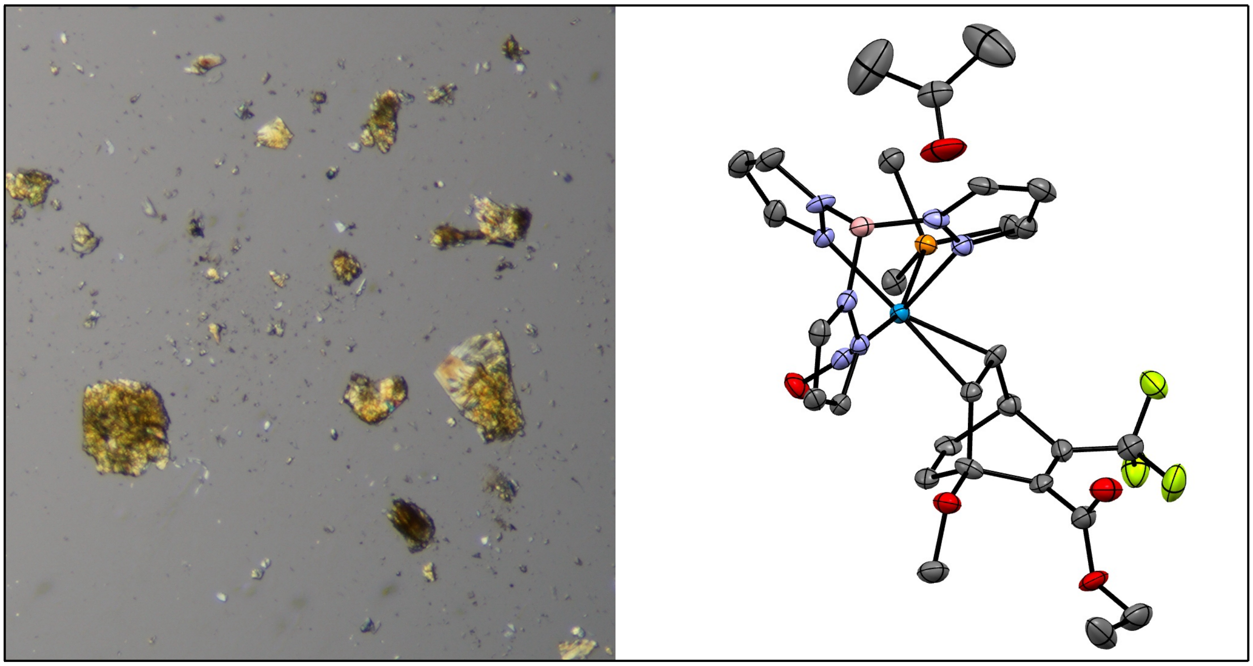

Fig. S29. Crystals of **4-*d*** (left) and ORTEP diagram (50% probability) showing the asymmetric unit of **4-*d*** (right) with acetone cosolvent displayed and hydrogen atoms omitted for clarity.

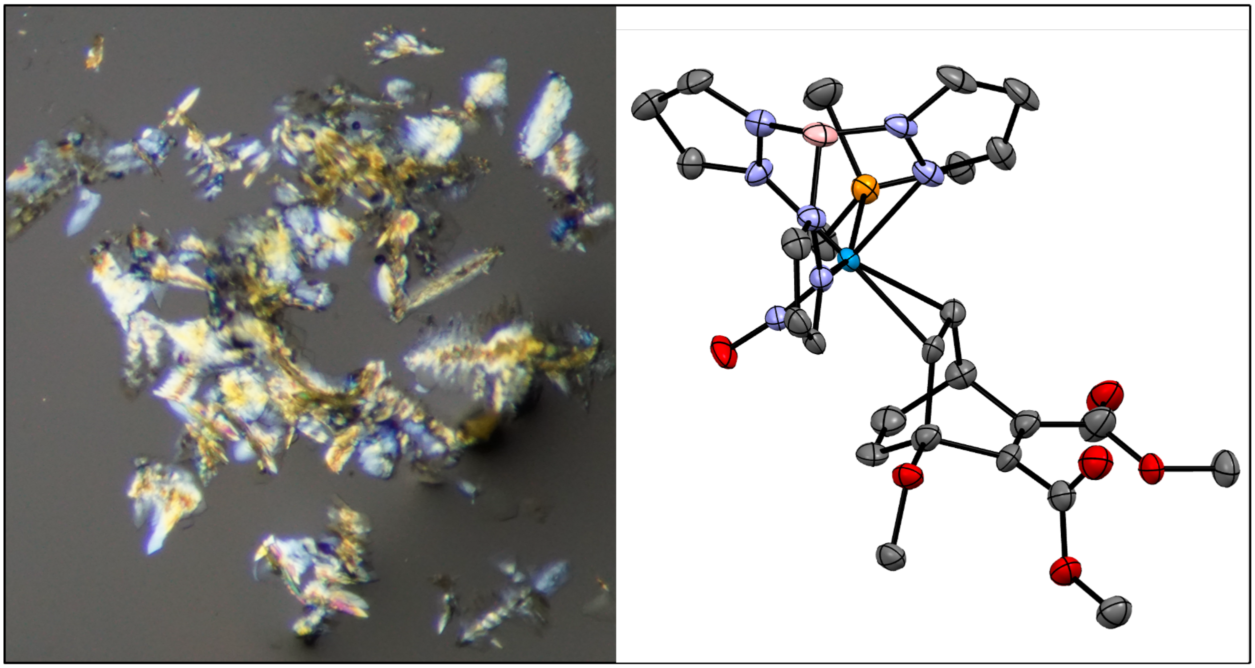

Fig. S30. Crystals of **5-*d*** (left) and ORTEP diagram (50% probability) showing the asymmetric unit of **5-*d*** (right). Hydrogen atoms and the minor orientation of disordered atoms are omitted for clarity.

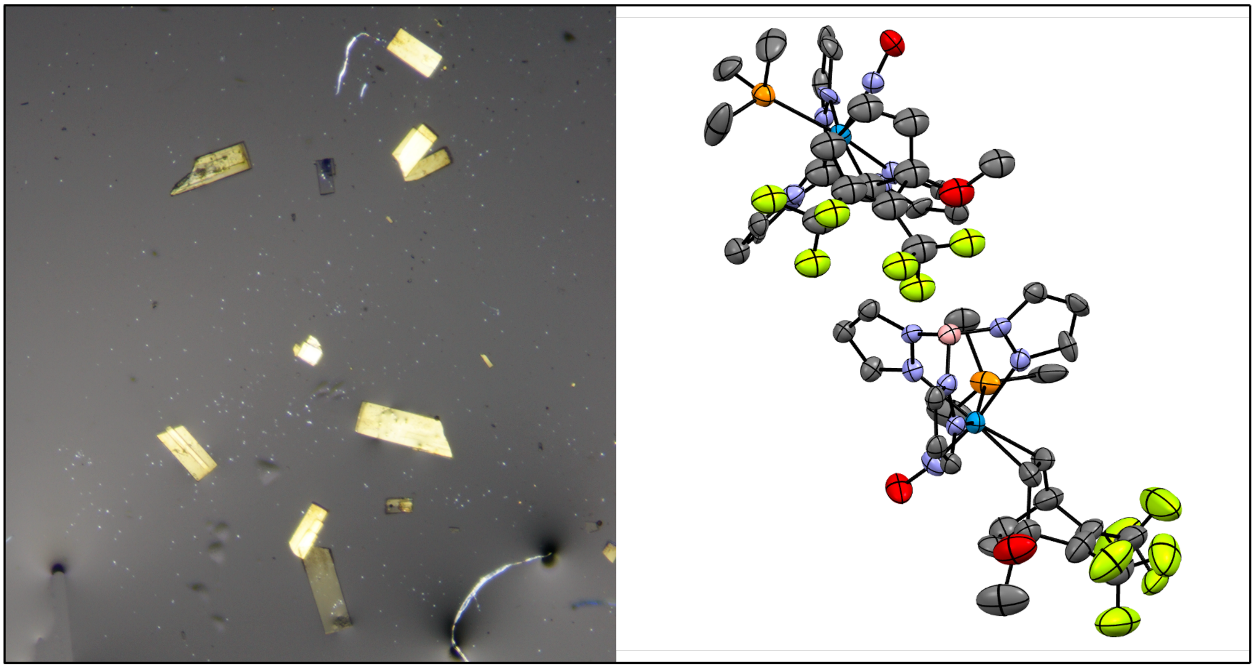

Fig. S31. Crystals of **6-*d*** (left) and ORTEP diagram (50% probability) showing the asymmetric unit of **6-*d*** (right). Hydrogen atoms and the minor orientation of disordered atoms are omitted for clarity.

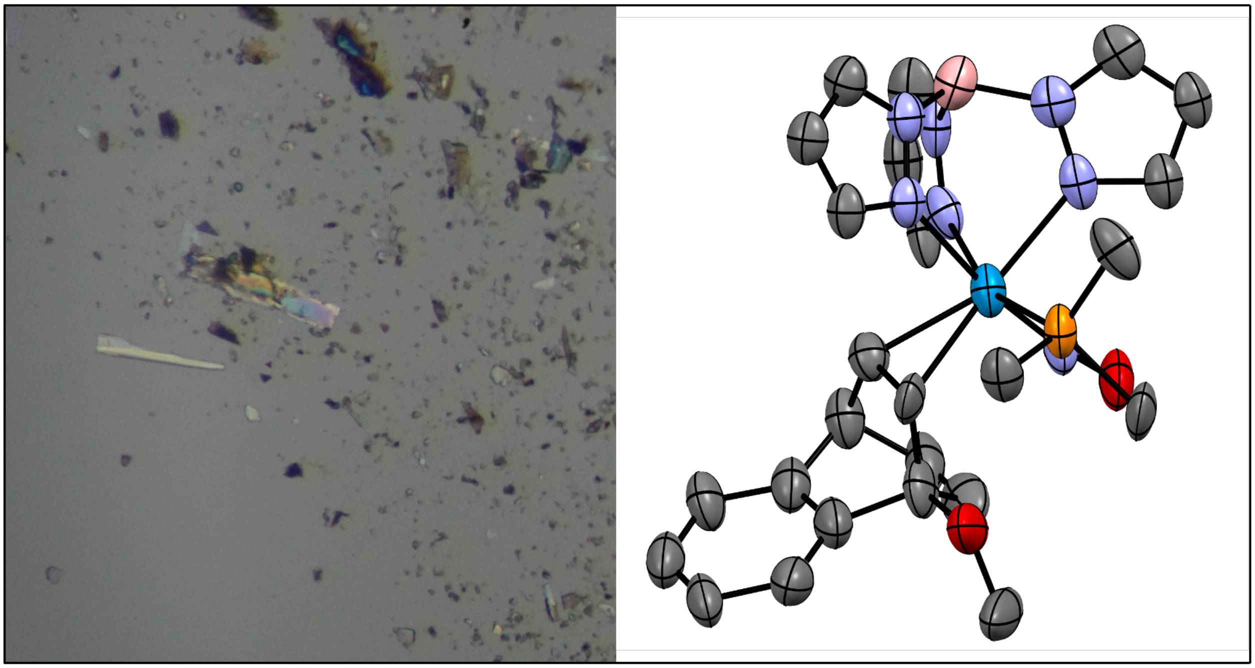

Fig. S32. Crystals of **7-*p*** (left) and ORTEP diagram (50% probability) showing the asymmetric unit of **7-*p*** (right) with hydrogen atoms omitted for clarity.

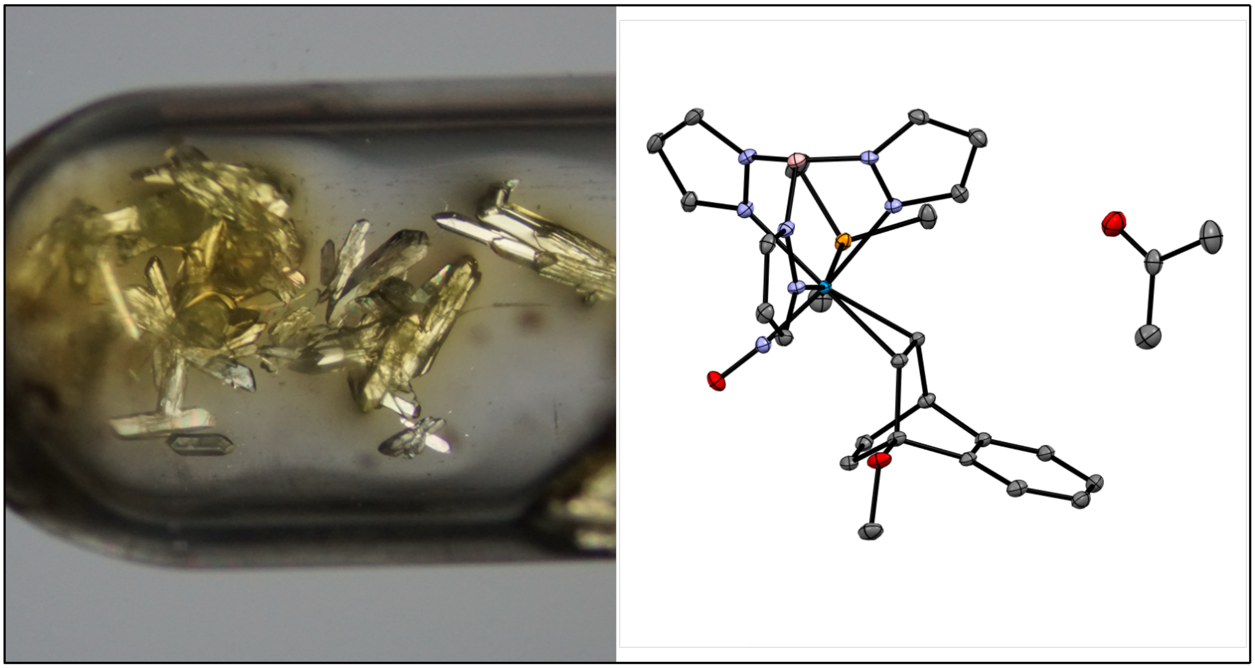

Fig. S33. Crystals of **7-*d*** (left) and ORTEP diagram (50% probability) showing the asymmetric unit of **7-*d*** (right) with acetone cosolvent displayed and hydrogen atoms omitted for clarity.

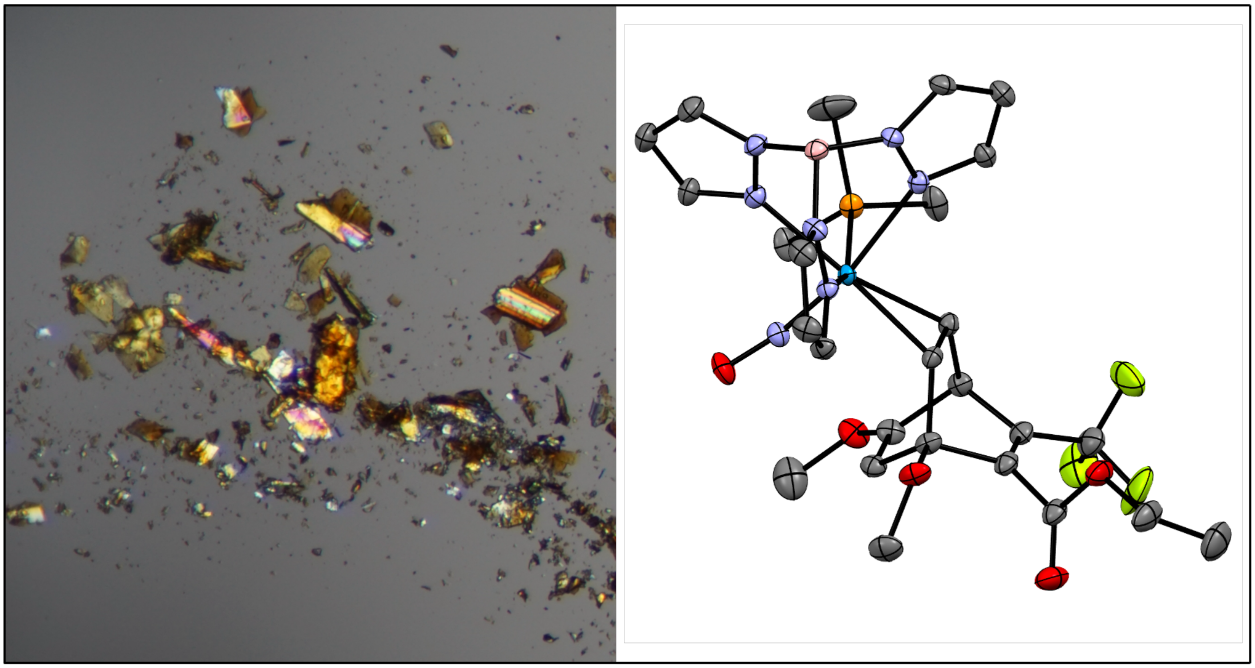

Fig. S34. Crystals of **8-*d*** (left) and ORTEP diagram (50% probability) showing the asymmetric unit of **8-*d*** (right). Hydrogen atoms and the minor orientation of disordered atoms are omitted for clarity.

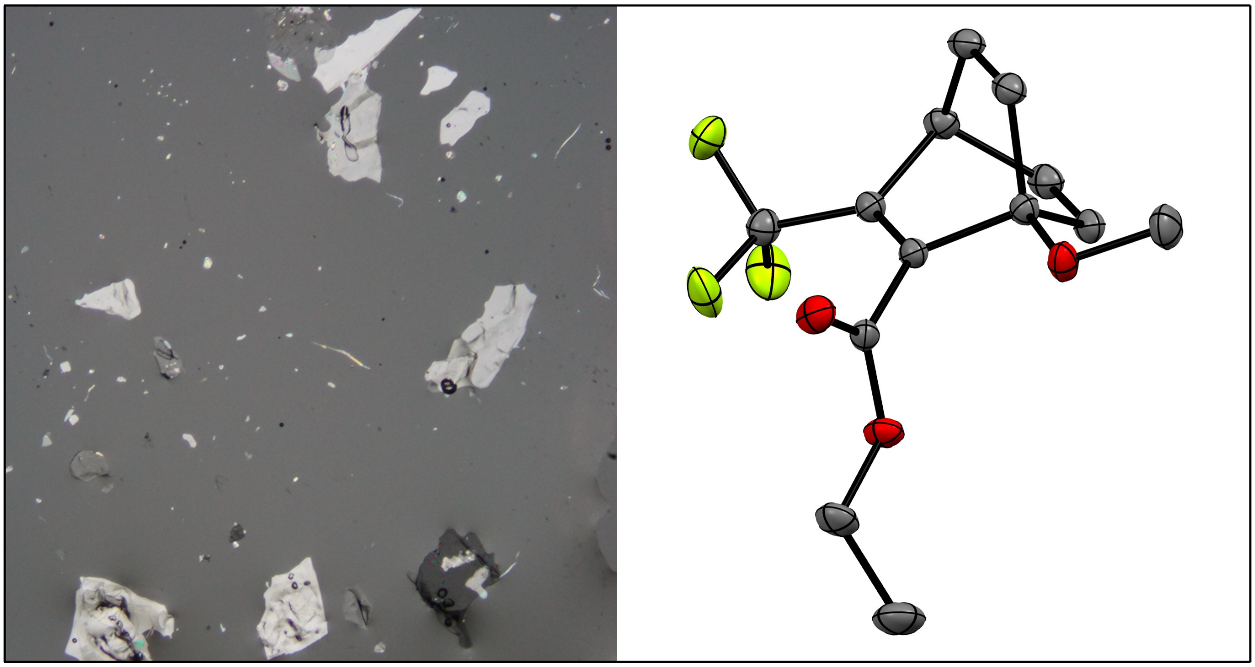

Fig. S35. Crystals of **10** (left) and ORTEP diagram (50% probability) showing the asymmetric unit of **10** (right) with hydrogen atoms omitted for clarity.

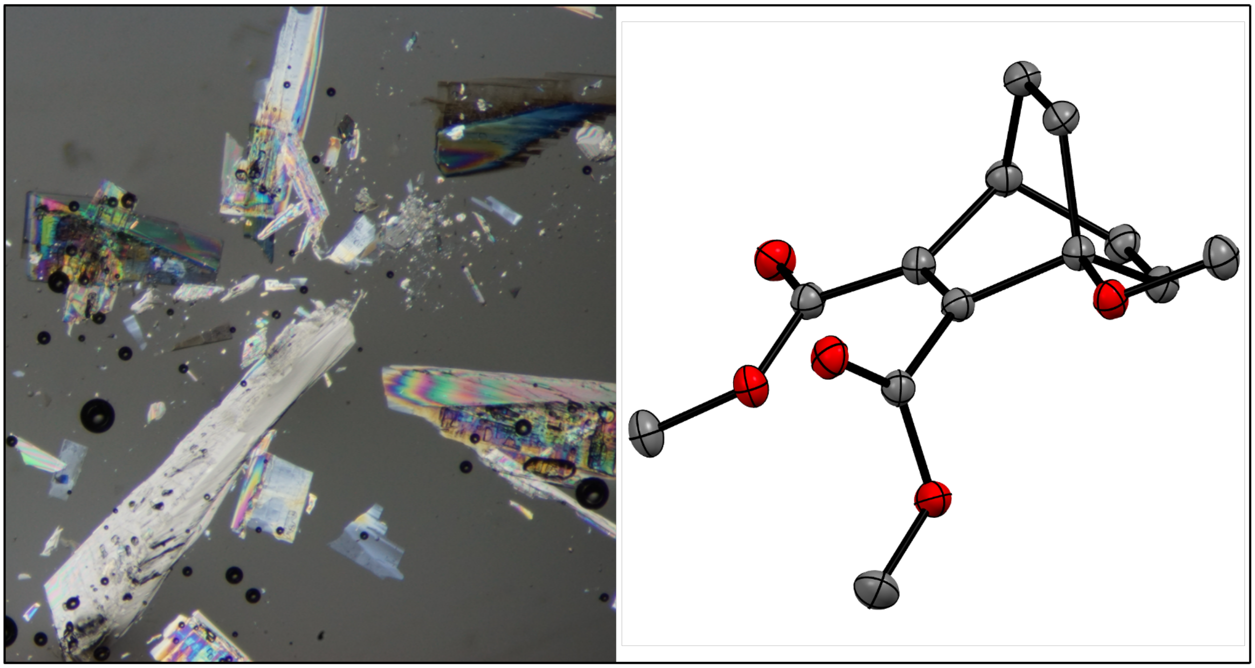

Fig. S36. Crystals of **11** (left) and ORTEP diagram (50% probability) showing the asymmetric unit of **11** (right) with hydrogen atoms omitted for clarity.

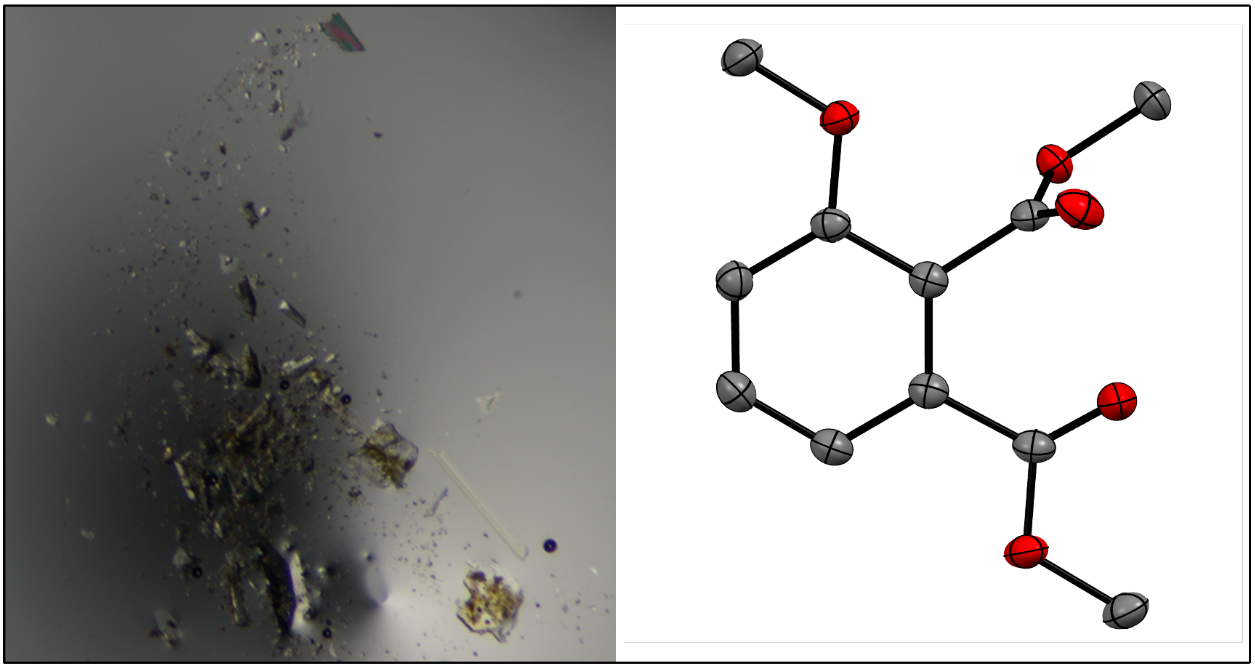

Fig. S37. Crystals of **16** (left) and ORTEP diagram (50% probability) showing the asymmetric unit of **16** (right) with hydrogen atoms omitted for clarity.

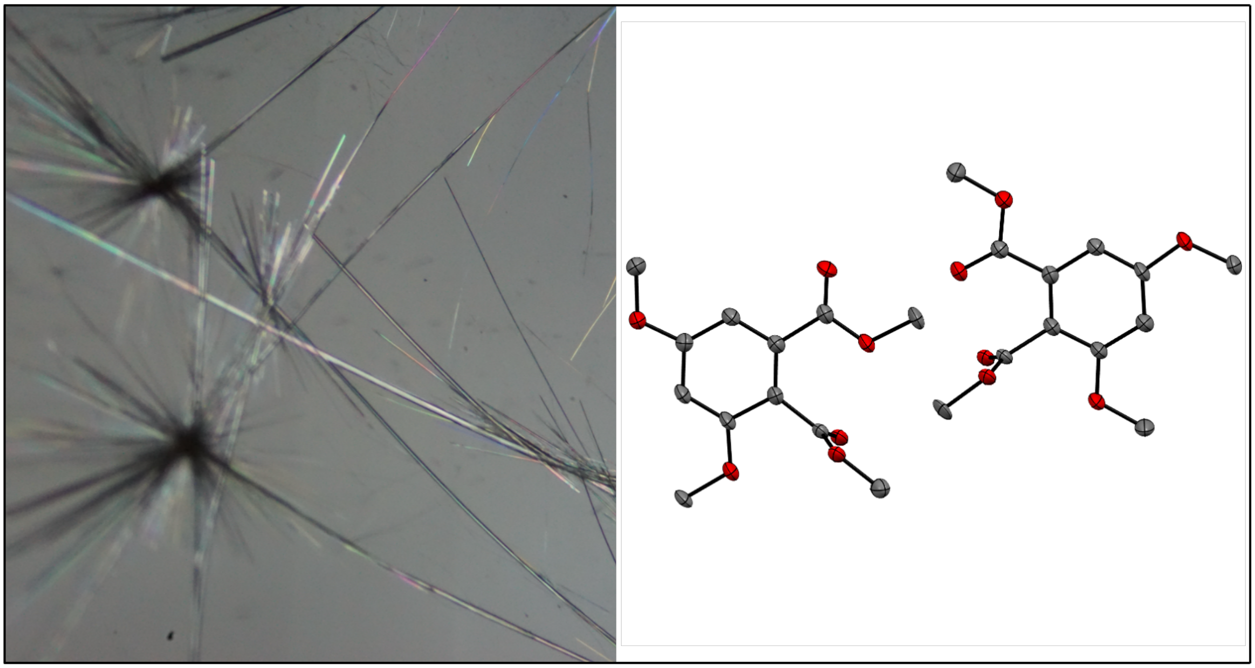

Fig. S38. Crystals of **19** (left) and ORTEP diagram (50% probability) showing the asymmetric unit of **19** (right). Hydrogen atoms and the minor orientation of disordered atoms are omitted for clarity.

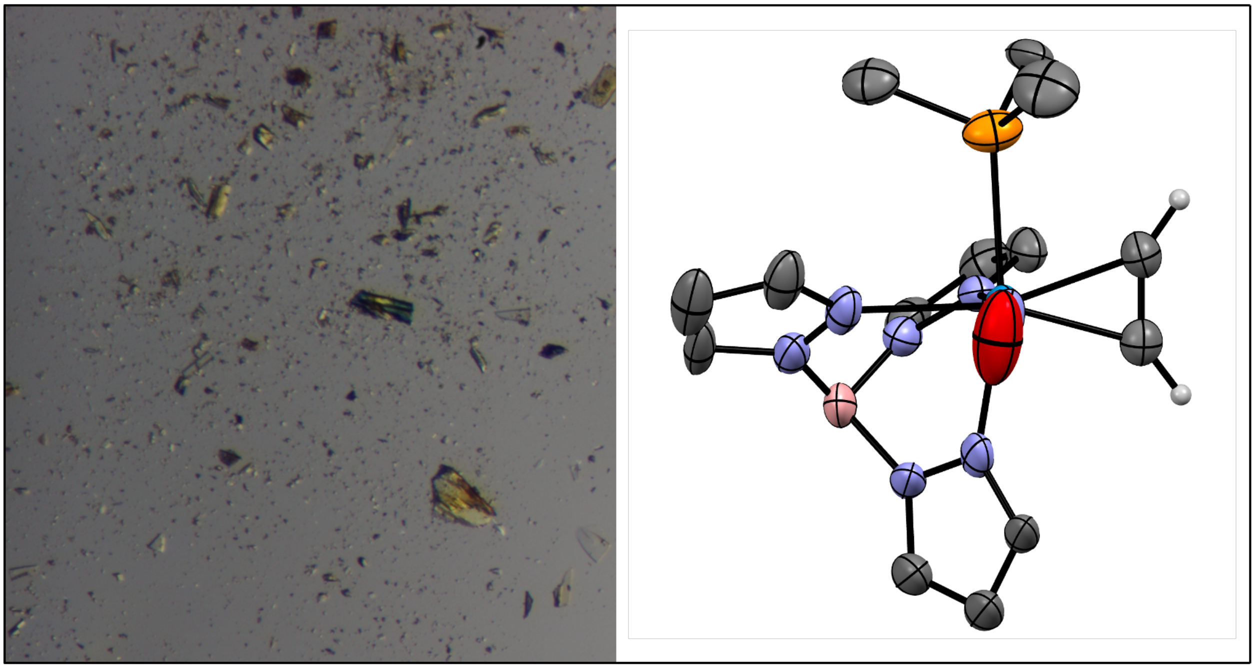

Fig. S39. Crystals of **20** (left) and ORTEP diagram (50% probability) showing the asymmetric unit of **20** (right). All hydrogen atoms except the η^2-acetylene ligand hydrogen atoms are omitted for clarity as are the minor position of disordered atoms.

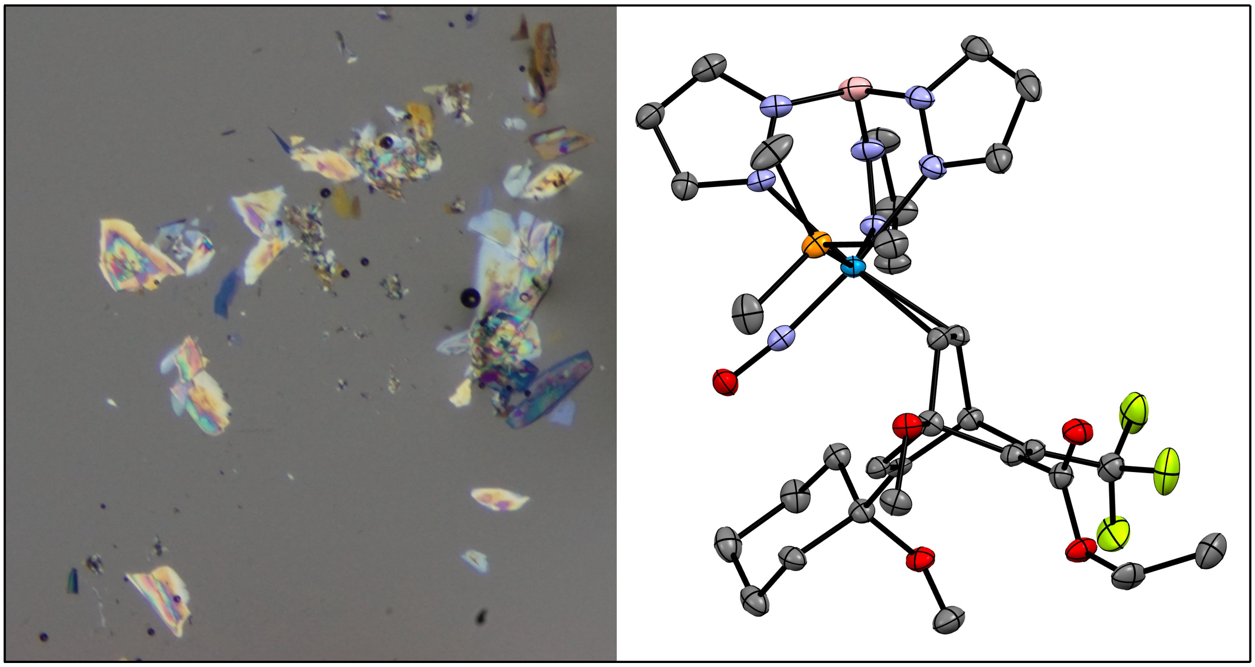

Fig. S40. Crystals of **21** (left) and ORTEP diagram (50% probability) showing the asymmetric unit of **21** (right) with hydrogen atoms omitted for clarity.

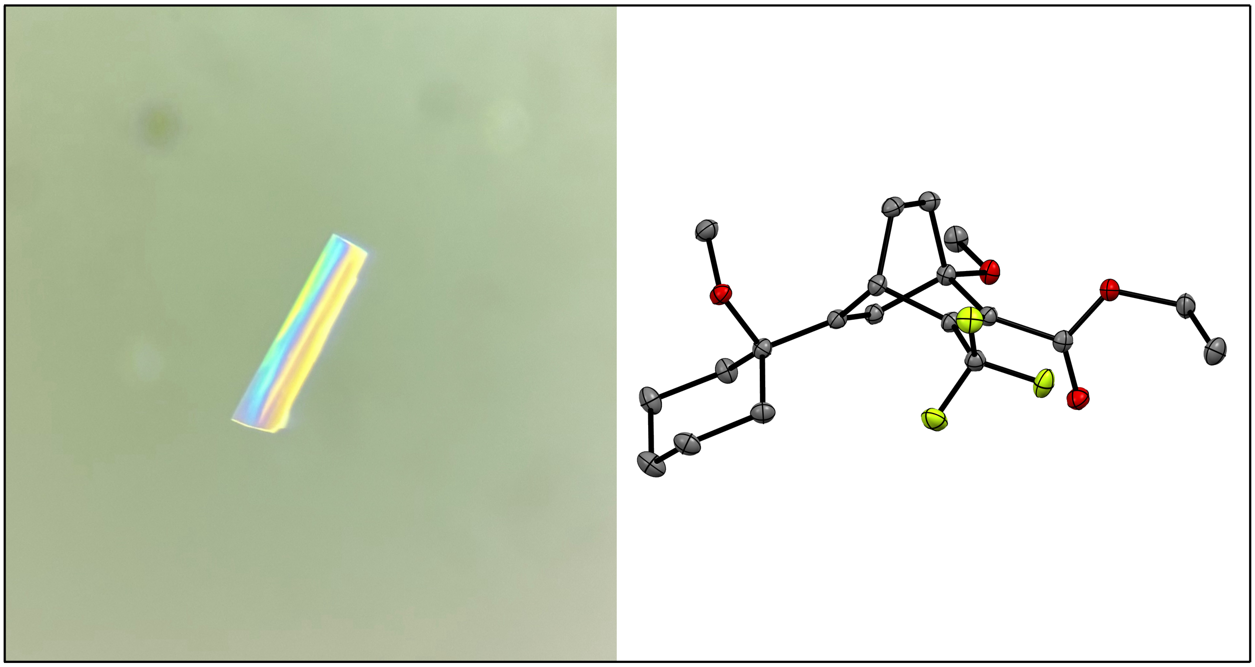

Fig. S41. Crystal of **22** (left) and ORTEP diagram (50% probability) showing the asymmetric unit of **22** (right) with hydrogen atoms omitted for clarity.

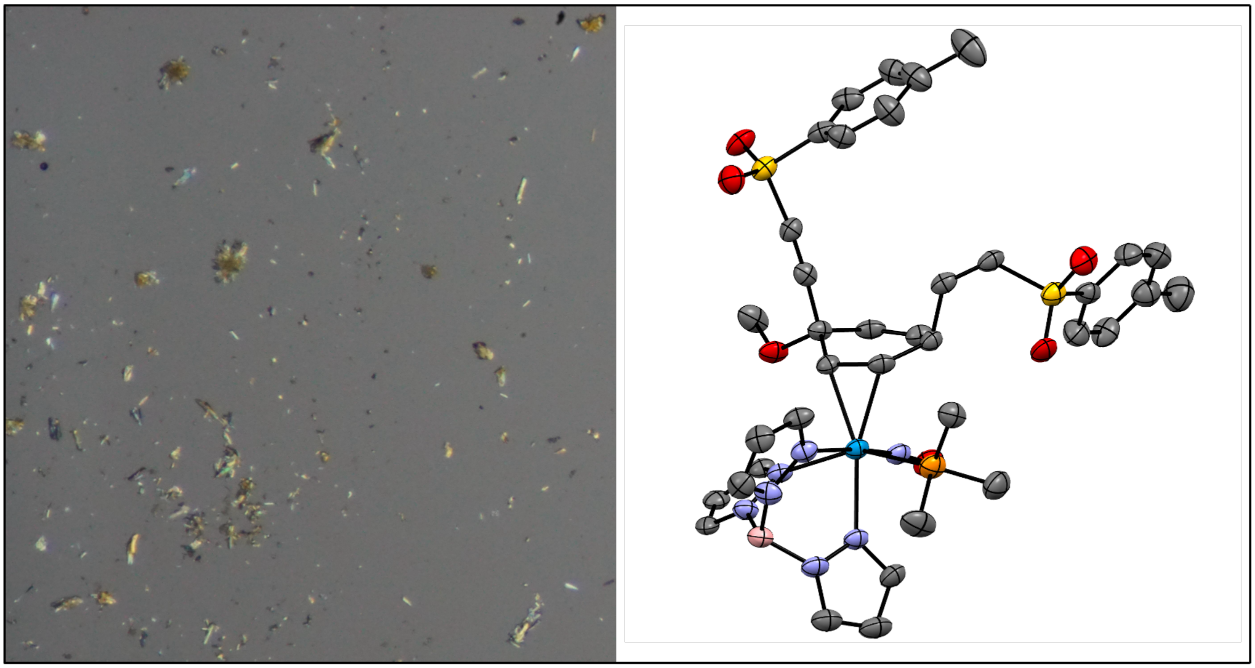

Fig. S42. Crystals of **S1** (left) and ORTEP diagram (50% probability) showing the asymmetric unit of **S1** (right) with hydrogen atoms omitted for clarity.

COMPUTATIONAL DETAILS

General procedure. All computations were performed using the Gaussian 16 suite, Revision B.01. Optimizations and frequency calculations were performed using the M06 functional, with the 6-31G(d, p) basis set on light atoms and the LANL2DZ basis set and effective core potential on tungsten. Implicit solvation was applied using the SMD model, with parameters for THF. Thermochemical corrections were applied at 298 K and 1 atm using the default implementation. All ground states were verified to have no imaginary frequencies and all transition states (TSes) exactly one imaginary frequency. TS searches were performed using the QST2 and QST3 algorithms, with force constants re-calculated analytically every 10 steps; in some cases, direct saddle point optimization (Opt=TS) was performed from structures known to be close to the TS. Tight convergence criteria were used as necessary when oscillatory convergence behavior was observed. IRC calculations were performed for all TSes to verify they connected the expected minima, using the default HPC algorithm with a step size of 0.1 Bohr where possible, and the LQA algorithm with step sizes of 0.1, 0.15, or 0.2 Bohr in cases where HPC failed to proceed to the minimum; force constants were re-calculated every 10 steps. Conformer search was carried out manually in GaussView 6.

DFT of minor pathways. The energetics and mechanisms of the minor pathways of DA reactivity were also probed using DFT (*i.e.*, the proximal pathway for dienophile **3a** and the distal pathway for dienophile **3d**; Figure S36). No significant differences in mechanism compared to the major pathways were observed. Further, consistent with the proposal that the significant degree of charge separation in **Int1** contributes to the Curtin-Hammett selectivity for the formation of **4-*d***, the proximal form of **Int1** is markedly less stable than **Int1-*d*** ($\Delta\Delta G^{\ddagger}_{(\mathbf{3a+1}\text{-}\boldsymbol{p}\rightarrow\mathbf{Int1}\text{-}\boldsymbol{p})\,-\,(\mathbf{3a+1}\text{-}\boldsymbol{d}\rightarrow\mathbf{Int1}\text{-}\boldsymbol{d})}$ = +5.7 kcal/mol). The energetics of the major and minor pathways were otherwise comparable.

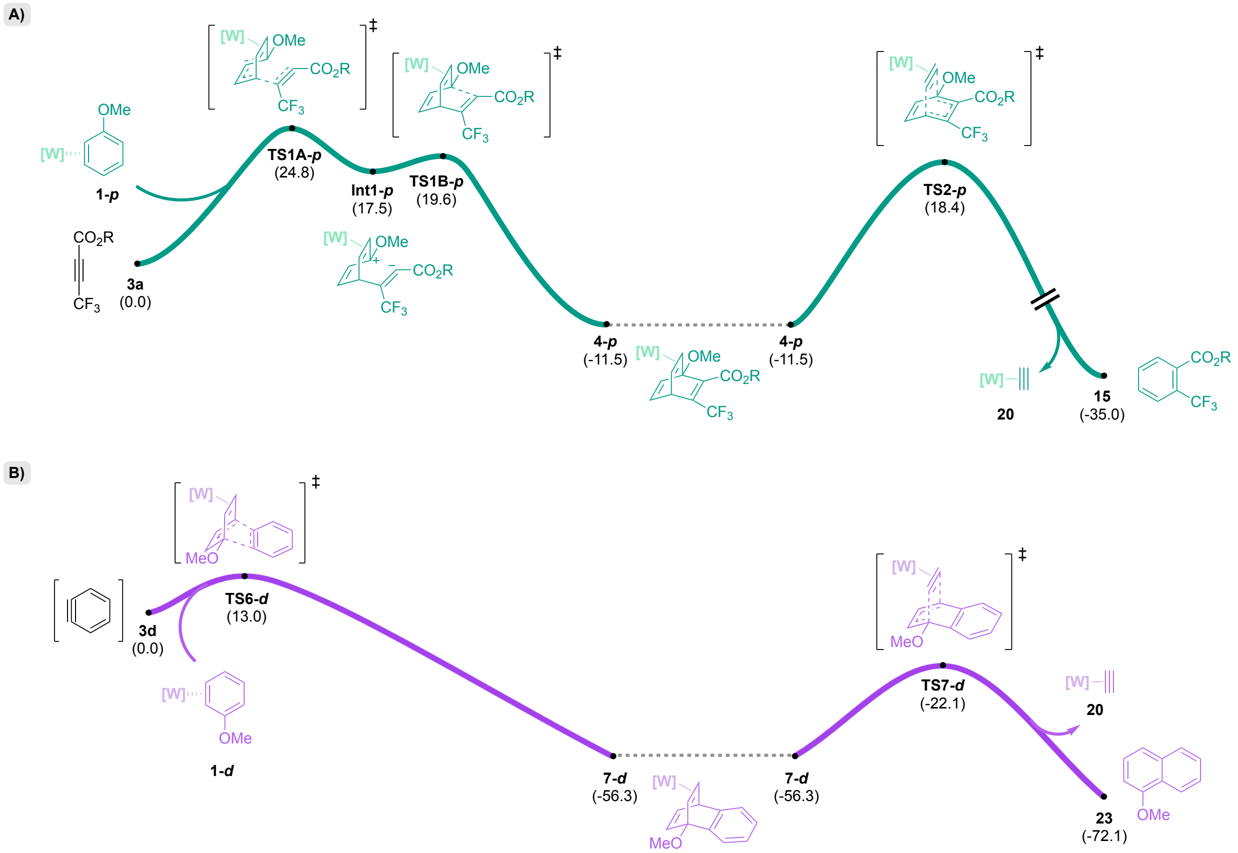

Figure S43. DFT (M06/6-31G(d,p)/LANL2DZ on W/THF solvation; kcal/mol) studies of the minor pathways of tungsten-facilitated DA and rDA reactions. For DFT studies, the R-group of **3a** was changed to methyl (from ethyl) to simplify the calculations.

Structure 1-*d*

Electronic energy: -1707.20417675 a.u.

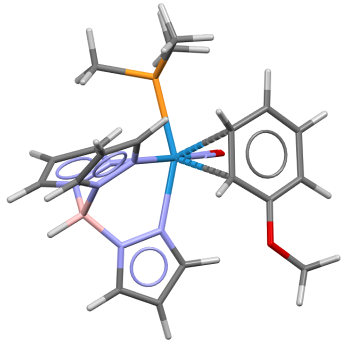

O	-1.42461100	-1.00386600	-2.73761200
N	-0.39687900	1.95152400	-0.29304300
N	0.61978400	2.78864000	0.04205200
N	1.02429800	0.31188400	1.73150800
N	1.88084000	1.36193100	1.69501000
N	1.69009600	0.40568100	-1.23306200
N	2.49809100	1.37822300	-0.75049400
N	-0.92599800	-0.70610500	-1.67469500
C	0.27313000	4.05894000	-0.24778400
C	-1.00020300	4.05725000	-0.78541700
C	-1.37243600	2.70917900	-0.79635300
C	2.49946800	1.49385200	2.88214000
C	2.03865200	0.50264800	3.73050800
C	1.11408800	-0.20739400	2.96188500
C	3.50726800	1.60056500	-1.61497700
C	3.36130300	0.74385800	-2.69217600
C	2.19730400	0.02314000	-2.40909200
B	2.01121600	2.24298400	0.43785600
H	2.77835400	3.14526500	0.64144500
H	0.96365000	4.86965900	-0.05071200
H	-1.57783600	4.90753800	-1.12007500
H	-2.29273200	2.24641700	-1.13140500
H	3.21873300	2.28839200	3.03739800
H	2.32406500	0.32489800	4.75772000
H	0.50815100	-1.06174900	3.24123800
H	4.24695600	2.36335400	-1.40585500
H	3.99786500	0.66161600	-3.56191800
H	1.70029300	-0.74988400	-2.98519100
C	2.98914800	-2.18469600	0.07526500
H	3.49694500	-3.15142400	0.17771800
H	3.36573300	-1.67685400	-0.81851400
H	3.22466300	-1.56386400	0.94943500
C	0.96005000	-3.51122000	-1.47747000
H	1.24772200	-2.97279000	-2.38726700
H	1.56979100	-4.41846200	-1.38761500
H	-0.09684600	-3.78863400	-1.56619200
W	-0.13359800	-0.27442700	-0.15282900
P	1.16963600	-2.42545800	-0.02083900
C	0.94716600	-3.60872600	1.36354600
H	1.63956300	-4.44954100	1.23546100
H	1.16774500	-3.11834700	2.31834900
H	-0.07530200	-3.99735900	1.39326400
C	-1.50155000	-1.51954000	1.14088400
C	-1.96709900	-0.13360500	1.10464900
C	-3.26138100	0.13695800	0.50614000
H	-0.98167800	-1.85668800	2.04188200

H	-1.72529400	0.52230300	1.94649200
C	-2.34361400	-2.52002400	0.49759300
H	-2.04840700	-3.56908200	0.55313800
O	-3.68170300	1.41232200	0.71389600
C	-4.90610600	1.79390900	0.13049600
H	-5.06263200	2.84580000	0.38303400
H	-4.88204400	1.68679000	-0.96448900
H	-5.74541100	1.20175100	0.52237900
C	-3.48392900	-2.17572600	-0.14863000
C	-3.97497500	-0.82278900	-0.14871400
H	-4.93405300	-0.60374600	-0.60921200
H	-4.08118600	-2.94034800	-0.64403400

Structure 1-*p*

Electronic energy: -1707.20604467 a.u.

Free energy: -1706.808403 a.u., -1071038.4434 kcal/mol

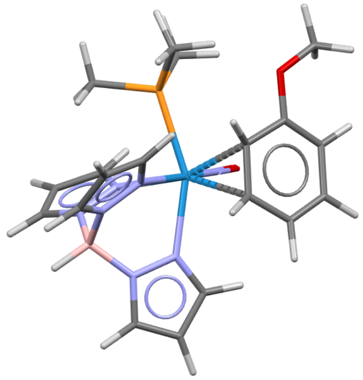

O	-1.45839100	0.98357400	-2.67576500
N	1.52775300	1.74583300	-0.14226600
N	2.80652200	1.38888800	0.14201400
N	1.05743200	-0.51541600	1.69179200
N	2.40930300	-0.59782800	1.63581700
N	1.49474700	-0.76338100	-1.30459100
N	2.76244500	-0.88499500	-0.84668900
N	-0.93711900	0.70026600	-1.61895200
C	3.62066000	2.45076200	-0.02476700
C	2.85903000	3.53092500	-0.42948800
C	1.55317300	3.03346300	-0.49187800
C	2.88769500	-1.09905900	2.78886700
C	1.82161100	-1.35538000	3.63311400
C	0.69855200	-0.96771900	2.89899500
C	3.52476000	-1.49321200	-1.77640300
C	2.73220400	-1.78593400	-2.87253000
C	1.46827400	-1.29616800	-2.53014000
B	3.18827700	-0.08631600	0.40784100
H	4.37092400	-0.19185300	0.59247000
H	4.68407000	2.35481800	0.15667500
H	3.19751800	4.53397300	-0.64854400
H	0.63066200	3.53209000	-0.76617500
H	3.95378600	-1.23087400	2.92630100
H	1.85358400	-1.75628800	4.63633100
H	-0.34693300	-0.98548100	3.18598900
H	4.57918500	-1.65979200	-1.59387900
H	3.02832100	-2.27359200	-3.79068900
H	0.54326100	-1.30348900	-3.09673200
C	0.20151000	-3.43239700	-0.23928900
H	-0.26465900	-4.42284800	-0.17051000
H	0.76006000	-3.36607100	-1.17811100
H	0.90923800	-3.31396700	0.59164200
C	-2.16855900	-2.47489200	-1.55568600
H	-1.62758400	-2.28446600	-2.48932100
H	-2.51327400	-3.51613900	-1.54366800
H	-3.03351200	-1.80247900	-1.51556900
W	-0.11859900	0.21702900	-0.12639200
P	-1.08069800	-2.11869000	-0.12943100
C	-2.05081500	-2.77617800	1.28152700
H	-2.34174700	-3.81141000	1.06446600
H	-1.43946900	-2.77322000	2.19129800
H	-2.94834600	-2.17271200	1.44218400
C	-1.91761300	0.56473900	1.18538300

C	-1.02607800	1.72029500	1.25493900
C	-3.19508600	0.76840300	0.51700300
H	-1.96664200	-0.12773300	2.03077600
H	-0.33919300	1.76528200	2.10569100
C	-1.50850200	3.00272400	0.77135100
H	-0.90288900	3.89032300	0.95284800
O	-3.97250500	-0.34912000	0.50806800
C	-5.21813000	-0.27545000	-0.14979100
H	-5.67298100	-1.26664700	-0.07438900
H	-5.88258800	0.46133900	0.32299500
H	-5.09678500	-0.01393400	-1.21085400
C	-2.70428900	3.10818300	0.14128700
C	-3.56522200	1.96674000	-0.01465200
H	-4.51880400	2.08668200	-0.52078900
H	-3.04923800	4.07199100	-0.23055600

Structure 3a

Electronic energy: -641.96155714 a.u.

Free energy: -641.922342 a.u., -402812.3512 kcal/mol

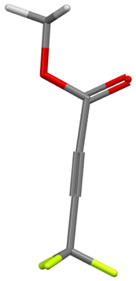

C	-1.88751600	0.39236900	-0.00232600
O	-2.52753300	-0.76993400	-0.00260700
O	-2.39616100	1.48721200	0.00060300
C	-0.44858300	0.18529000	-0.00497800
C	0.75062900	0.07877500	-0.00552300
C	2.20566400	-0.06826900	-0.00018700
F	2.61282900	-0.80735200	-1.03189900
F	2.81277100	1.11521300	-0.08072600
F	2.61612800	-0.66256500	1.11988700
C	-3.96084700	-0.67779400	0.00274700
H	-4.30604900	-0.15362700	0.89820100
H	-4.31264200	-0.14913600	-0.88746300
H	-4.32340000	-1.70535300	0.00153400

Structure 3d

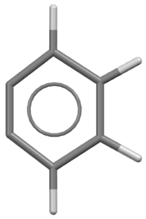

Electronic energy: -230.73757359 a.u.

Free energy: -230.690823 a.u., -144760.6770 kcal/mol

C	-0.62445500	-1.23094100	0.00012400
C	-1.46247600	-0.13277800	-0.00017000
C	-0.70104200	1.05103100	0.00001900
C	0.70105300	1.05102400	0.00002100
C	1.46247700	-0.13279200	-0.00016600
C	0.62444000	-1.23094100	0.00012700
H	-2.54886400	-0.12941900	-0.00005700
H	-1.22556500	2.00562800	0.00018600
H	1.22558600	2.00561600	0.00019000
H	2.54886500	-0.12944600	-0.00005000

Structure 4-*d*

Electronic energy: -2349.21368463 a.u.

Free energy: -2348.747520 a.u., -1473861.3211 kcal/mol

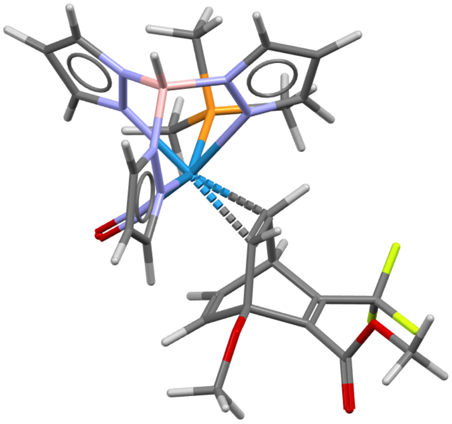

B	3.34984400	1.62249200	1.28783500
H	4.18084900	2.26022100	1.87608900
C	0.82459100	-0.37160900	2.97778300
H	-0.06831100	-0.98171600	2.90566600
C	1.52257500	0.03166000	4.11754500
H	1.29866700	-0.19898800	5.14946700
C	2.55869100	0.80671200	3.62774200
H	3.35180400	1.34103800	4.13588400
C	4.04314100	-0.61488500	-1.45622100
H	3.62733600	-1.21501600	-2.25801300
C	5.37411600	-0.28899800	-1.18161500
H	6.25689800	-0.58421100	-1.73118200
C	5.30738400	0.51307600	-0.05512500
H	6.08229300	1.02281200	0.50398500
C	0.88597700	3.08402200	-0.95925500
H	0.02609300	2.91710300	-1.59847600
C	1.54591800	4.27037500	-0.62601500
H	1.33290000	5.26942400	-0.97964700
C	2.52834100	3.88151300	0.26643900
H	3.28054900	4.45457800	0.79439400
C	-1.00684300	-0.89818300	0.14981000
H	-1.09107500	-1.31345100	1.15919500
C	-1.04612800	0.54359600	0.01679500
H	-1.09657100	1.14432000	0.93305000
C	-2.16348300	0.98134900	-0.96835700
C	-1.92691500	0.28314500	-2.28123900
H	-1.70070200	0.84604200	-3.18359700
C	-1.87800700	-1.04314900	-2.18346100
H	-1.61435400	-1.70512700	-3.00409500
C	-2.07314200	-1.55960500	-0.78562300
H	-2.06895500	-2.65445000	-0.72839600
C	-3.37021700	-0.96979600	-0.25290100
C	-3.42907900	0.36084100	-0.33585000
C	-4.31578100	-1.85890600	0.45142600
C	0.38717700	-3.57308500	1.15051400
H	0.40117200	-3.16046700	2.16539700
H	0.75681900	-4.60490500	1.18957000
H	-0.64573300	-3.57961200	0.78838300
C	3.10056500	-2.92426600	0.84054700
H	3.20327400	-3.99381000	1.06014100
H	3.16895100	-2.35786200	1.77852800
H	3.92370900	-2.61221300	0.18985100

C	1.50643800	-3.59662200	-1.46655800
H	0.51291000	-3.57105700	-1.92966100
H	1.77781200	-4.63627000	-1.24696800
H	2.22245600	-3.17630800	-2.18092600
C	-2.97876700	2.92558300	-2.08901100
H	-2.38110400	2.98651600	-3.01088200
H	-3.26998300	3.94149700	-1.80307500
H	-3.89182300	2.35043300	-2.29765800
F	-5.35314300	-1.21042500	0.99611400
F	-4.82126400	-2.80026100	-0.36377600
F	-3.69673400	-2.52551600	1.44699400
N	1.40044600	0.11704100	1.87250100
N	2.46786800	0.84489700	2.28634800
N	3.23438200	-0.06534900	-0.54509700
N	4.01739900	0.62858900	0.31164900
N	1.43567500	2.05188900	-0.31587700
N	2.44791600	2.54839100	0.44045100
N	0.93611200	-0.37474100	-2.12109900
O	1.03858800	-0.55921500	-3.31380800
O	-2.23812900	2.38496100	-1.01325600
P	1.47518000	-2.58719900	0.05584000
W	0.99458100	-0.14249800	-0.36819600
O	-5.43107300	1.67661200	-0.36262000
C	-4.46213400	1.26402700	0.23341200
O	-4.15178400	1.59952200	1.48812700
C	-5.05363300	2.51548500	2.11527900
H	-6.05652700	2.08290600	2.18592300
H	-4.65291500	2.69788700	3.11333600
H	-5.10615700	3.45317000	1.55344800

Structure 4-*p*

Electronic energy: -2349.21589005 a.u.

Free energy: -2348.749069 a.u., -1473862.2931 kcal/mol

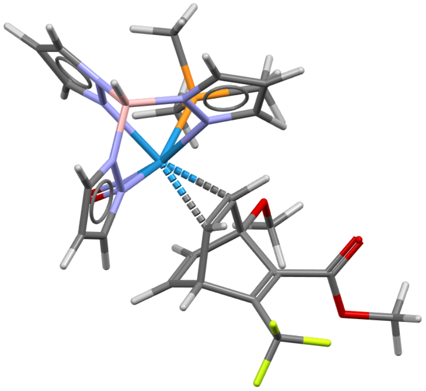

B	3.66931000	1.46335800	1.06462900
H	4.59842800	2.04001000	1.56186700
C	0.89862400	0.13029700	2.99836800
H	-0.07282400	-0.34997500	3.00722700
C	1.65261000	0.60370200	4.07365600
H	1.40308800	0.56995800	5.12472900
C	2.78763600	1.13500600	3.48813600
H	3.65276200	1.62148100	3.92132700
C	3.95836200	-1.18987000	-1.36220400
H	3.43908900	-1.82348500	-2.07356600
C	5.32919800	-1.02007400	-1.15038200
H	6.14592800	-1.50405800	-1.66719000
C	5.40741100	-0.08023100	-0.13722900
H	6.26133300	0.38321700	0.34127700
C	1.44157000	2.95277600	-1.39640100
H	0.59301000	2.81653000	-2.05726400
C	2.26257400	4.06850400	-1.21088300
H	2.19883000	5.02856800	-1.70343400
C	3.17593500	3.66834500	-0.25223200
H	4.00247400	4.19398800	0.20962000
C	-1.06330100	-0.42135000	0.27537400
H	-1.21613500	-0.66347400	1.33208500
C	-0.82301700	0.96188000	-0.07837200
H	-0.72788700	1.67924300	0.74549000
C	-1.86504900	1.45317600	-1.13545700
C	-1.80048100	0.52903500	-2.31782900
H	-1.50690100	0.88304200	-3.30305700
C	-2.00209700	-0.74929300	-2.00931400
H	-1.88549200	-1.58110300	-2.69988400
C	-2.24957800	-0.99611600	-0.54422100
C	-3.41483300	-0.07234100	-0.11625800
C	-3.19285500	1.20770700	-0.43384600
C	0.03504500	-3.12264900	1.76764700
H	0.35448700	-2.62119500	2.68812300
H	0.16977500	-4.20374100	1.89628000
H	-1.02065500	-2.91058100	1.57740000
C	2.69443000	-3.21203200	0.89303600
H	2.58091600	-4.22199400	1.30506700
H	3.10852900	-2.55249200	1.66748100
H	3.39952000	-3.24760600	0.05748600
C	0.56187100	-3.69862400	-0.97893000
H	-0.49665300	-3.51803600	-1.20137200

H	0.69947400	-4.74474100	-0.67926600
H	1.14867900	-3.49398700	-1.88112900
N	1.53306000	0.35247200	1.84069900
N	2.69740600	0.97446100	2.15572200
N	3.26052400	-0.42335500	-0.51960900
N	4.15723800	0.25961700	0.22930900
N	1.83291400	1.95042100	-0.60629700
N	2.90307400	2.39636700	0.09664800
N	0.90745700	-0.58374300	-2.05084800
O	0.95251500	-0.94526400	-3.20652900
P	1.06100100	-2.56118200	0.36040400
W	1.04149200	-0.13222700	-0.34618700
O	-2.45879900	-2.34238000	-0.19751400
C	-3.43070700	-3.02478000	-0.96058600
H	-3.03031900	-3.38155100	-1.92122000
H	-3.75314400	-3.89326100	-0.37752400
H	-4.31154500	-2.39981900	-1.17074900
C	-4.48074200	-0.59653300	0.77090900
O	-4.27770800	-0.99235100	1.89620800
O	-5.67672400	-0.61135800	0.17175600
C	-6.75604700	-1.07371900	0.98636100
H	-6.88432600	-0.42541800	1.85925800
H	-7.64705000	-1.03626900	0.35810300
H	-6.57630800	-2.09917500	1.32476100
C	-3.98550600	2.38129600	-0.00359800
F	-3.21399900	3.25515200	0.67002100
F	-4.48522800	3.05599000	-1.05268500
F	-5.01535900	2.07515600	0.79608500
H	-1.73007900	2.50749900	-1.39928300

Structure 7-*d*

Electronic energy: -1938.06153818 a.u.

Free energy: -1937.587077 a.u., -1215854.2477 kcal/mol

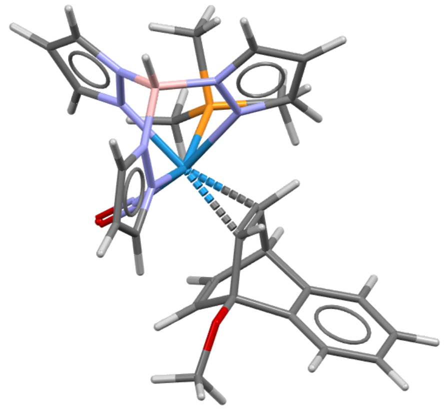

B	3.01936700	1.48129800	1.24721800
H	3.91150100	2.05955500	1.80729400
C	0.69742400	-0.71488300	2.97792900
H	-0.19552400	-1.32757300	2.93964800
C	1.52114400	-0.43342700	4.06934600
H	1.41684700	-0.78193900	5.08712500
C	2.49449300	0.40480300	3.55557300
H	3.34055700	0.88894600	4.02731700
C	3.39725600	-0.43001500	-1.78740000
H	2.89027800	-0.93654700	-2.60132800
C	4.75127700	-0.12011200	-1.63040800
H	5.56544400	-0.34013600	-2.30658500
C	4.81397200	0.54872000	-0.41988200
H	5.64743600	1.00284100	0.10168900
C	0.33279000	3.15229500	-0.55253200
H	-0.59458400	3.04512400	-1.10422700
C	1.03203500	4.30168200	-0.17191100
H	0.78765900	5.33079600	-0.39513500
C	2.10238000	3.82818100	0.56467700
H	2.91000100	4.34744800	1.06575500
C	-1.45303500	-0.93697200	0.33829700
H	-1.41685600	-1.42441900	1.31804300
C	-1.50083500	0.51385400	0.31888600
H	-1.44853300	1.04049000	1.28010400
C	-2.70723000	1.02331700	-0.50966700
C	-2.61871900	0.40932200	-1.88455400
H	-2.49844000	1.02882200	-2.77064700
C	-2.56556500	-0.92197100	-1.88784500
H	-2.40967100	-1.51706800	-2.78472000
C	-2.60469000	-1.54617000	-0.51855100
H	-2.58103900	-2.64328000	-0.54473000
C	-3.84831600	-1.02347200	0.17428000
C	-3.90309600	0.38010800	0.19208300
C	0.05941700	-3.70623300	0.87471000
H	0.14393700	-3.38904900	1.91992400
H	0.45666400	-4.72459100	0.78517400
H	-0.99769200	-3.71206600	0.59225900
C	2.72162100	-2.97343600	0.36591300
H	2.85494600	-4.05797100	0.45982400
H	2.88285800	-2.50657600	1.34630900
H	3.47026200	-2.58040900	-0.32935100
C	0.90899600	-3.43630100	-1.82372700

H	-0.12499300	-3.38192800	-2.18405700
H	1.20936600	-4.48770700	-1.73851700
H	1.54875600	-2.93346200	-2.55676800
C	-3.62184700	3.03870300	-1.40278900
H	-3.86485300	4.03715800	-1.02274600
H	-4.56039600	2.47857900	-1.52732400
H	-3.15733400	3.15612300	-2.39412900
N	1.14183900	-0.09711800	1.87667100
N	2.25027600	0.59243400	2.24625900
N	2.69829000	0.00316900	-0.73406100
N	3.57401900	0.60553300	0.10163900
N	0.94206600	2.06213800	-0.08212700
N	2.03314200	2.48367600	0.60769400
N	0.25214500	-0.18547800	-2.07603300
O	0.23958600	-0.26004100	-3.28612800
O	-2.75042900	2.43334600	-0.47530400
P	1.02073300	-2.58649700	-0.20960100
W	0.49102300	-0.12225800	-0.32445100
C	-4.91744700	1.03220200	0.87217200
C	-5.90881700	0.27942100	1.51388500
C	-5.86499600	-1.10877100	1.48457400
C	-4.82244100	-1.76637000	0.82044500
H	-4.93624000	2.12026500	0.91447100
H	-6.71422000	0.78657700	2.04198100
H	-6.63747600	-1.68828800	1.98668200
H	-4.77251200	-2.85544500	0.81406300

Structure 7-*p*

Electronic energy: -1938.06404090 a.u.

Free energy: -1937.588226 a.u., -1215854.9687 kcal/mol

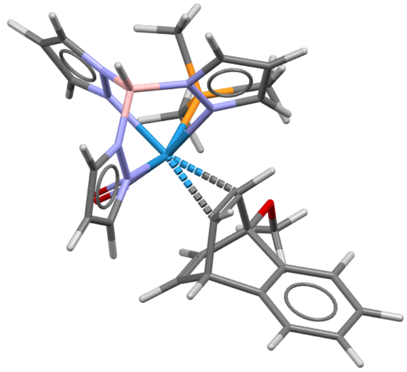

B	3.33509700	1.37443000	0.84653600
H	4.33190900	1.90713100	1.25449100
C	0.59962200	0.36645200	3.01315200
H	-0.40111700	-0.03919500	3.10535800
C	1.44925300	0.84432200	4.01272300
H	1.26256700	0.89000200	5.07632800
C	2.58109700	1.25502700	3.33180400
H	3.50323600	1.70032100	3.68409700
C	3.28585800	-1.43359200	-1.41738600
H	2.67988700	-2.06730100	-2.05621400
C	4.67594500	-1.35533400	-1.29388000
H	5.42325100	-1.92989900	-1.82291500
C	4.88255600	-0.36429100	-0.35007600
H	5.79487600	0.06119300	0.04938600
C	1.08494100	2.87994700	-1.58341400
H	0.19211000	2.76903900	-2.18845500
C	1.99722200	3.93715100	-1.51619600
H	1.97911100	4.86568900	-2.06931000
C	2.93056600	3.52757900	-0.58123100
H	3.81962400	4.01425300	-0.19971300
C	-1.56667800	-0.18360700	0.43298500
H	-1.66355800	-0.35827200	1.51009400
C	-1.23800100	1.15943500	-0.00773800
H	-1.02306700	1.89983900	0.77209800
C	-2.27842500	1.69464300	-1.03510400
C	-2.36960100	0.70027800	-2.16093900
H	-2.11850300	0.97013300	-3.18453500
C	-2.65656700	-0.54083100	-1.77068500
H	-2.65473400	-1.40602100	-2.42991100
C	-2.84073600	-0.69902200	-0.28172600
C	-3.89437500	0.31987800	0.15271100
C	-3.58644100	1.62403600	-0.27058400
C	-0.59642400	-2.88573400	2.01887400
H	-0.20901400	-2.35237200	2.89417200
H	-0.52318500	-3.96384800	2.20818200
H	-1.64397800	-2.61903700	1.85407600
C	2.00383600	-3.21568200	1.02892700
H	1.84166200	-4.19183800	1.50181100
H	2.50233000	-2.54844300	1.74495900
H	2.66220900	-3.34581600	0.16511000
C	-0.25516400	-3.64385700	-0.70595300
H	-1.30990500	-3.39809200	-0.87785800
H	-0.17332200	-4.67986700	-0.35503500
H	0.28866600	-3.52977600	-1.65000800

N	1.17615400	0.47338900	1.81002700
N	2.39840900	1.02443700	2.01916100
N	2.69701000	-0.56685800	-0.58905400
N	3.68411400	0.09063100	0.06246600
N	1.44326100	1.90209400	-0.74858700
N	2.58224100	2.30555000	-0.13358900
N	0.25187900	-0.62988400	-1.97842100
O	0.20811200	-1.05683400	-3.11267900
P	0.39560300	-2.47389900	0.53667600
W	0.51764400	-0.09701100	-0.31328700
C	-4.40200500	2.68294200	0.09222000
C	-5.53602700	2.44418600	0.87786200
C	-5.82837400	1.15679800	1.31020000
C	-4.99834300	0.08606100	0.95501900
H	-4.15567900	3.69543400	-0.22784300
H	-6.18362100	3.27263900	1.15874400
H	-6.70357100	0.97758700	1.93203200
H	-5.21256400	-0.91963900	1.31386900
O	-3.13206100	-2.01329500	0.14831000
C	-4.13730400	-2.68373100	-0.58044900
H	-3.74582500	-3.15083100	-1.49716700
H	-4.53498100	-3.47718400	0.06161500
H	-4.96518000	-2.01740700	-0.86219100
H	-2.05331400	2.71393600	-1.37063200

Structure 10

Electronic energy: -988.51387414 a.u.

Free energy: -988.347254 a.u., -620197.2656 kcal/mol

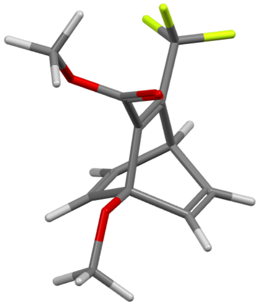

C	1.48583900	-0.61076300	-0.02102200
C	1.38778100	-1.68407500	-1.11225200
H	2.13362900	-1.72706700	-1.90023500
C	0.31899300	-2.46114000	-0.99127400
H	0.04146600	-3.27193400	-1.65661000
C	-0.56151300	-2.08930200	0.20189200
H	-1.45451600	-2.71069100	0.29632900
C	-0.90070500	-0.61061800	0.00079100
C	0.16603200	0.17191900	-0.11906400
C	-2.31997900	-0.19884300	-0.03054300
C	3.83402800	-0.28913700	-0.05158200
H	4.55417600	0.49969000	-0.28380900
H	3.98356800	-1.11545600	-0.76107000
H	4.04222200	-0.66030600	0.96187300
F	-2.47613900	1.11974700	-0.19414400
F	-2.98477200	-0.80868700	-1.02512900
F	-2.94903800	-0.53856200	1.10671400
O	2.54980000	0.28480000	-0.16012400
O	0.26703300	2.12327900	-1.48293900
C	0.22500100	1.63675600	-0.37924600
O	0.25624100	2.32161600	0.76274900
C	0.35194300	3.74291700	0.61816600
H	1.26515300	4.01509100	0.08087800
H	-0.51482100	4.13407900	0.07715400
H	0.37608200	4.14762400	1.63047500
C	1.40925000	-1.34647600	1.32197500
H	2.17085700	-1.18429000	2.07841200
C	0.34098400	-2.12577300	1.43454800
H	0.08121900	-2.72956600	2.29781600

Structure 15

Electronic energy: -911.23926936 a.u.

Free energy: -911.101101 a.u., -571724.5727 kcal/mol

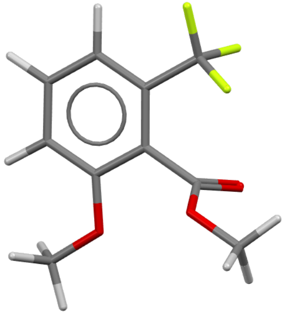

C	1.60875000	0.68733400	0.02651800
O	2.63361500	-0.17424200	0.18694200
C	3.94979500	0.34949500	0.18176100
H	4.61781700	-0.50113900	0.32862400
H	4.18702800	0.83075600	-0.77584400
H	4.09910500	1.06800600	0.99818100
C	-0.79015200	0.93555000	-0.07152200
C	0.32878700	0.11613900	0.06800500
C	0.20796300	-1.35887900	0.30333200
O	-0.10337500	-1.85377300	1.36043500
O	0.47868800	-2.04576700	-0.80575800
C	-2.16800700	0.35053400	0.02745500
F	-3.08297800	1.12203700	-0.56897200
F	-2.24616900	-0.86252300	-0.54310900
F	-2.55278700	0.20436500	1.30210500
C	0.32791800	-3.46347500	-0.69214600
H	-0.70382300	-3.71639300	-0.42716900
H	0.57447900	-3.87322000	-1.67232700
H	1.00391300	-3.86863300	0.06652700
C	0.62223400	2.86030700	-0.29522200
C	-0.65019800	2.30913700	-0.25310300
H	-1.52923200	2.93567300	-0.36600800
H	0.74205700	3.93080900	-0.43975500
C	1.75224300	2.06293800	-0.15590400
H	2.73863300	2.51501000	-0.19043700

Structure 20

Electronic energy: -1437.98460489 a.u.

Free energy: -1437.685493 a.u., -902161.2676 kcal/mol

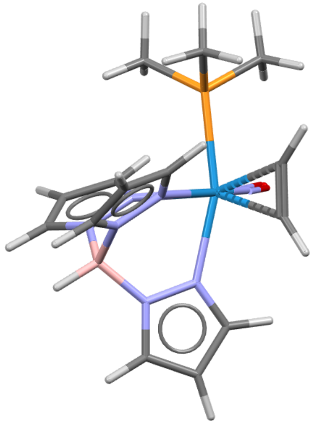

B	2.38865500	0.75943500	1.06434100
H	3.37516900	1.12948800	1.64241500
C	-0.02942500	-1.56133900	2.39765200
H	-0.88631300	-2.19026200	2.18268900
C	0.79948800	-1.52970200	3.52157600
H	0.72309000	-2.12568600	4.42007800
C	1.75358000	-0.57401100	3.21845400
H	2.61476200	-0.22497600	3.77484100
C	0.08119800	2.98544200	-0.62483900
H	-0.80117100	3.08605000	-1.24587900
C	0.95607100	3.97060000	-0.15951000
H	0.90562200	5.03731200	-0.32710500
C	1.90961400	3.27060100	0.55834100
H	2.78922500	3.60630900	1.09376100
C	2.45583900	-1.52170200	-1.77370800
H	1.88608800	-2.08491800	-2.50379300
C	3.83755000	-1.45283400	-1.57489500
H	4.61387800	-1.96528900	-2.12551200
C	3.98903700	-0.58069100	-0.51191400
H	4.87567200	-0.22191000	-0.00391000
N	0.37987600	-0.66810000	1.49214300
N	1.48173900	-0.06655800	2.00156900
N	0.47119900	1.77604700	-0.21092100
N	1.60002200	1.96178300	0.51517800
N	1.81971600	-0.74702200	-0.89237600
N	2.76924800	-0.16827500	-0.11685100
P	-2.55691500	0.34022400	0.42906200
W	-0.37251800	-0.33783700	-0.60007500
C	-3.92836900	-0.69495700	-0.18981500
H	-3.68510800	-1.74807000	-0.00682400
H	-4.03932200	-0.55372100	-1.27031200
H	-4.87136800	-0.44226000	0.30995700
C	-3.16864400	2.04494600	0.15952200
H	-3.21107700	2.28349100	-0.90773400
H	-2.50382900	2.75987800	0.65718000
H	-4.17458200	2.14554500	0.58518400
C	-2.76988300	0.20787200	2.24603600
H	-3.76263700	0.57847100	2.52953600
H	-2.00562500	0.80322900	2.75858500
H	-2.67970000	-0.83326900	2.57132200
C	-0.29406600	0.08170200	-2.67271000
H	0.34469600	-0.00479200	-3.54698500

C	-1.44855100	0.49121400	-2.26624900
H	-2.37311300	0.94158000	-2.61840900
N	-0.95778500	-2.01103600	-0.63418800
O	-1.37078400	-3.15468200	-0.54633400

Structure 21

Electronic energy: -77.26647701 a.u.

Free energy: -77.259162 a.u., -48480.8561 kcal/mol

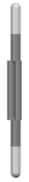

C	0.60307200	-0.00240200	0.00001900
C	-0.60307200	-0.00236800	-0.00001900
H	1.67307400	0.01434600	-0.00010500
H	-1.67307600	0.01427400	0.00010800

Structure 22

Electronic energy: -2271.90731699 a.u.

Free energy: -2271.469563 a.u., -1425368.6709 kcal/mol

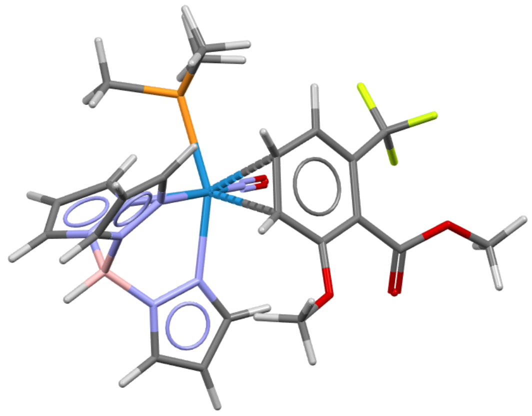

O	-1.27102100	-0.81112200	-2.19958200
N	0.98645300	1.90502600	-0.60817200
N	2.20961600	2.48702600	-0.51333900
N	2.27132400	0.22464100	1.53449800
N	3.32235200	1.02183400	1.22422100
N	2.42374800	-0.28581400	-1.41973900
N	3.51145000	0.49972300	-1.22896100
N	-0.47706600	-0.57026400	-1.32354300
C	2.15102300	3.73691300	-1.01818200
C	0.85980400	3.98018800	-1.44792200
C	0.16922100	2.79388000	-1.17809400
C	4.13959900	1.13838100	2.28604300
C	3.61227100	0.39905000	3.33052600
C	2.44077600	-0.15193100	2.80772600
C	4.39246600	0.30133100	-2.22806700
C	3.87322200	-0.64451900	-3.09464900
C	2.62954300	-0.97138900	-2.54866300
B	3.46232200	1.64007600	-0.17997700
H	4.45585400	2.31182300	-0.24155200
H	3.03892000	4.35670200	-1.03970700
H	0.47099200	4.88361500	-1.89637100
H	-0.87513300	2.54758600	-1.34033300
H	5.03669100	1.74143600	2.22124300
H	4.01578300	0.27960800	4.32605700
H	1.71193700	-0.79560000	3.28771400
H	5.31959400	0.86022800	-2.25521500
H	4.32383000	-1.03248600	-3.99721300
H	1.87737100	-1.66681000	-2.90477400
C	3.05957700	-3.04750800	0.08397100
H	3.24871800	-4.10607800	0.29870100
H	3.39997800	-2.82335800	-0.93184900
H	3.63814000	-2.43073500	0.78375600
C	0.45144200	-3.76880600	-0.92168200
H	0.72263100	-3.47827200	-1.94284400
H	0.73652200	-4.81496900	-0.75716600
H	-0.63573200	-3.66600500	-0.81852300
W	0.68887600	-0.22749900	-0.03519900
P	1.27509300	-2.66572100	0.28140800
C	0.94142800	-3.52088000	1.86824600
H	1.31062600	-4.55160400	1.80636100
H	1.45874400	-3.01286000	2.69012000
H	-0.13111100	-3.54693200	2.08532800

C	-0.75023700	-0.79282300	1.60241200
C	-0.70880400	0.66051400	1.46233200
C	-1.87779900	1.35085000	0.97520200
H	-0.27497800	-1.23439800	2.48127400
H	-0.10215600	1.19927000	2.18925100
C	-1.93249300	-1.46069100	1.10106600
H	-1.97105100	-2.54783900	1.15288400
O	-1.95363800	2.69582900	1.04782000
C	-0.99319500	3.41924800	1.79636600
H	-1.24066600	4.47455300	1.65678800
H	-1.05506200	3.18001000	2.86637600
H	0.03293200	3.24943900	1.44426700
C	-2.94323300	-0.78391100	0.50060700
C	-2.95832500	0.67296100	0.45254200
C	-4.09642500	1.42749400	-0.09697700
O	-4.03954700	2.37522500	-0.85335100
O	-5.26917400	0.92383400	0.34330000
C	-3.99468300	-1.59183600	-0.19509200
F	-5.09288700	-1.81674800	0.54972900
F	-4.41428700	-1.02095700	-1.33726900
F	-3.54151700	-2.81512300	-0.53503000
C	-6.42604600	1.38607600	-0.33942900
H	-6.34755700	1.18281100	-1.41396400
H	-7.27037700	0.83468100	0.07972900
H	-6.57490000	2.46111900	-0.19366100

Structure 23

Electronic energy: -500.07291834 a.u.

Free energy: -499.927861 a.u., -313709.4691 kcal/mol

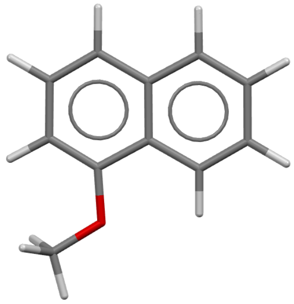

C	-1.07817100	0.84899100	-0.00006500
C	-0.23249600	-0.29697200	-0.00004800
C	3.32420600	-1.11342300	0.00039100
H	3.72599300	-2.12884300	0.00067000
H	3.68216900	-0.58624900	0.89511200
H	3.68279300	-0.58651100	-0.89423600
O	1.91726900	-1.23864700	-0.00006800
C	-0.81294900	-1.58658600	-0.00000700
C	-2.17848000	-1.73828300	0.00001300
C	-3.02061900	-0.60683500	-0.00000700
C	-2.48088600	0.65541100	-0.00004500
H	-0.16090600	-2.45571000	0.00001100
H	-2.61396900	-2.73492100	0.00004700
H	-4.10037800	-0.73855600	0.00001100
H	-3.12426800	1.53431100	-0.00005800
C	-0.50387300	2.14586600	-0.00009200
C	1.18388600	-0.10290000	-0.00005800
C	0.85761500	2.29087100	-0.00010300
C	1.71538900	1.16817000	-0.00008900
H	2.79012400	1.32258300	-0.00011500
H	1.30071600	3.28442800	-0.00012800
H	-1.16215200	3.01278500	-0.00010200

Structure TS1A-*d*

Electronic energy: -2349.15761251 a.u.

Free energy: -2348.695163 a.u., -1473828.4666 kcal/mol

Imaginary vibration: 358.31i cm^{-1}

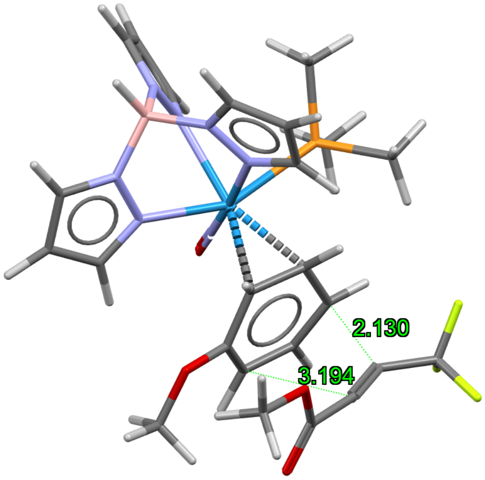

B	3.53612200	1.25371800	1.15440900
H	4.47436900	1.77431900	1.69387500
C	0.77504300	-0.24965200	2.97010700
H	-0.20033500	-0.72171300	2.93591300
C	1.54467300	0.09841200	4.08163900
H	1.30637800	-0.04767500	5.12567700
C	2.67744500	0.68430500	3.54468200
H	3.55022900	1.11599200	4.01857300
C	3.81467900	-1.18467600	-1.50110900
H	3.29627000	-1.74982200	-2.26785800
C	5.18461500	-1.05292400	-1.25991600
H	6.00075400	-1.50218900	-1.80795700
C	5.26301500	-0.20345500	-0.16973800
H	6.11747300	0.20538700	0.35519000
C	1.40052600	2.94210400	-1.25630600
H	0.57168800	2.86856000	-1.95046100
C	2.26554500	4.01141300	-1.00796800
H	2.26424900	4.98713700	-1.47307200
C	3.12472900	3.54052300	-0.03231200
H	3.96168700	4.01100600	0.46862100
C	-1.18701000	-0.60509200	0.18652400
H	-1.30182800	-1.00413900	1.19828800
C	-0.99323700	0.84157900	0.07288100
H	-0.87297700	1.43224500	0.98450800
C	-1.70634900	1.53555500	-0.97065000
C	-2.32352600	0.87606900	-2.02538300
H	-2.77129200	1.42317600	-2.84850300
C	-2.45756100	-0.51418900	-1.94839500
H	-2.98488100	-1.04302100	-2.74079000
C	-2.10226600	-1.20804700	-0.79281400
H	-2.17943500	-2.29531900	-0.81006100
C	-3.97340200	-0.78116800	0.13033400
C	-4.23565700	0.38479800	0.48488600
C	-4.53015700	-2.13831800	0.23371600
C	-0.17529200	-3.44722900	1.27610600
H	-0.12915500	-2.97701200	2.26483600
H	0.07298900	-4.50997100	1.38398800
H	-1.19676600	-3.36320800	0.89382900

C	2.59831700	-3.17568100	0.97248800
H	2.56046200	-4.24379300	1.21833200
H	2.72405600	-2.60117700	1.89925300
H	3.46368800	-2.98823800	0.32887600
C	0.95308400	-3.73767600	-1.32388400
H	-0.00147600	-3.57215300	-1.83672000
H	1.03774800	-4.79491600	-1.04493200
H	1.76017600	-3.48297400	-2.01942100
C	-2.35966300	3.66157600	-1.80470700
H	-2.23932500	4.69880100	-1.48379400
H	-3.42629500	3.41276000	-1.84028600
H	-1.90664900	3.53837800	-2.79767000
F	-3.76315600	-2.94305100	0.99456900
F	-5.74842700	-2.13613900	0.78646900
F	-4.64419900	-2.73914700	-0.96121900
N	1.40007800	0.09404300	1.83731400
N	2.57066400	0.67197200	2.20398400
N	3.11691300	-0.47991600	-0.60458800
N	4.01343400	0.12375300	0.21025500
N	1.71104900	1.90169500	-0.48039800
N	2.77825100	2.27456500	0.27245600
N	0.67071600	-0.45489600	-2.10770700
O	0.53770700	-0.67165200	-3.29008400
O	-1.68671200	2.86821200	-0.83430900
P	1.03896700	-2.66171400	0.15119200
W	0.92112000	-0.18998900	-0.37592500
O	-4.83005000	2.60285600	-0.01931600
C	-4.24389300	1.78973400	0.67877100
O	-3.56109500	2.12641700	1.79684900
C	-3.49429400	3.52399000	2.05914500
H	-4.49199400	3.94622400	2.22099200
H	-3.01231500	4.05555800	1.23179700
H	-2.89613700	3.63224700	2.96619600

Structure Int-*d*

Electronic energy: -2349.17762717 a.u.

Free energy: -2348.710126 a.u., -1473837.8560 kcal/mol

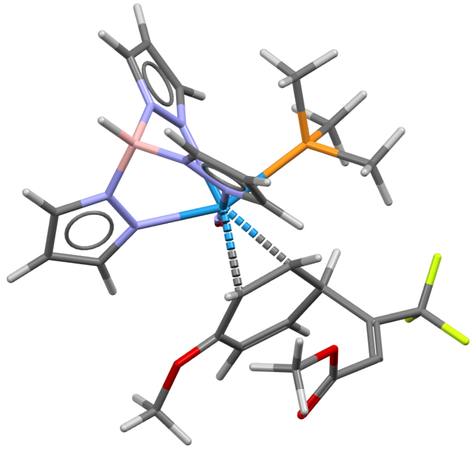

B	3.75135900	0.58709200	0.93270100
H	4.82405200	0.86934600	1.39055500
C	0.76198900	0.15358100	2.94219700
H	-0.31093000	-0.00493200	2.97260500
C	1.64531500	0.39116600	3.99625000
H	1.41791300	0.44683500	5.05133800
C	2.87681300	0.55013400	3.38509900
H	3.85565600	0.76143300	3.79723200
C	3.21753100	-2.06122200	-1.47819900
H	2.53044500	-2.52884600	-2.17447000
C	4.58209100	-2.28455700	-1.28553600
H	5.21981600	-2.98287200	-1.80872700
C	4.93965700	-1.39750400	-0.28445900
H	5.89801100	-1.19144000	0.17586900
C	2.06248400	2.57058500	-1.61051400
H	1.22592100	2.65834000	-2.29409900
C	3.19346400	3.37847600	-1.47889300
H	3.44192000	4.26799700	-2.04025000
C	3.92781800	2.78189000	-0.46979300
H	4.88114700	3.04747200	-0.03006300
C	-1.29466400	-0.02250800	0.26593400
H	-1.50143000	-0.27564600	1.30668600
C	-0.78585600	1.34106100	0.11226700
H	-0.43310100	1.89576600	0.98264300
C	-1.23686500	2.14292700	-0.95387200
C	-2.05660700	1.62282900	-2.00369400
H	-2.28909600	2.22806000	-2.87374800
C	-2.58411500	0.38633700	-1.86070700
H	-3.23294900	-0.00541000	-2.64275400
C	-2.42807900	-0.45694100	-0.64812900
H	-2.25518400	-1.48693800	-0.99886500
C	-3.81006300	-0.42759900	0.06109200
C	-4.27542700	0.49682500	0.90272400
C	-4.64357300	-1.60252800	-0.32243600
C	-1.01982400	-2.84279900	1.67131300
H	-0.82172500	-2.26613700	2.58158000
H	-1.02972300	-3.90896700	1.92812300
H	-2.00475700	-2.56877500	1.28223800
C	1.72337700	-3.32950200	1.29591100
H	1.42286900	-4.30373800	1.69938200

H	2.04505300	-2.68503300	2.12365200
H	2.56775400	-3.47227900	0.61451200
C	-0.13024100	-3.74616600	-0.87439500
H	-1.08969500	-3.46212900	-1.32307000
H	-0.21898700	-4.75678600	-0.45791300
H	0.63263200	-3.74538600	-1.66006600
C	-1.35919000	4.33375600	-1.87619600
H	-0.96239600	5.30585400	-1.58143900
H	-2.45323400	4.36256400	-1.86181600
H	-0.99718600	4.08173400	-2.87936800
F	-4.06579200	-2.78358100	0.03625500
F	-5.86619500	-1.61282100	0.20658400
F	-4.80426500	-1.70541400	-1.66768500
N	1.41469500	0.15865200	1.77254800
N	2.71669000	0.40778400	2.05741800
N	2.77972600	-1.11957700	-0.63522000
N	3.84540600	-0.71281700	0.09591700
N	2.10632600	1.56219300	-0.73499500
N	3.26238100	1.69564800	-0.03420600
N	0.32725800	-0.53658300	-2.05829900
O	-0.00625400	-0.82089100	-3.18232500
O	-0.87205500	3.40583000	-0.90253800
P	0.30063900	-2.55268900	0.43996200
W	0.78002900	-0.18843700	-0.38202600
O	-3.71984600	2.79894100	0.74060000
C	-3.63017400	1.70248300	1.29089600
O	-2.97317700	1.57779200	2.49639700
C	-2.42538900	2.78141000	2.99887400
H	-3.20537300	3.51808400	3.22860700
H	-1.72544600	3.24279900	2.28963600
H	-1.89380500	2.52128000	3.91900400

Structure TS1B-*d*

Electronic energy: -2349.16599419 a.u.

Free energy: -2348.699822 a.u., -1473831.3901 kcal/mol

Imaginary vibration: 76.92i cm^{-1}

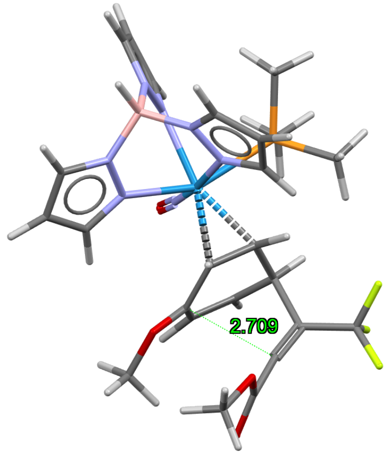

B	3.49094100	1.35812100	1.26014700
H	4.39875300	1.89272000	1.83571300
C	0.79452500	-0.37624900	2.96422900
H	-0.15948600	-0.88647600	2.89871600
C	1.54516300	-0.06321700	4.09836900
H	1.30974100	-0.28146600	5.13033400
C	2.65281500	0.60233100	3.60394200
H	3.50413200	1.04275200	4.10785600
C	3.89903900	-0.89220600	-1.53967100
H	3.41206700	-1.43782600	-2.34040200
C	5.25933600	-0.69467600	-1.29169500
H	6.09833600	-1.05868900	-1.86773200
C	5.29227000	0.08792000	-0.15017900
H	6.12336600	0.51425100	0.39778200
C	1.23763400	3.09605500	-1.01001400
H	0.40699800	3.02777500	-1.70312900
C	2.02727300	4.19704600	-0.67048000
H	1.95071900	5.20839100	-1.04400500
C	2.93062000	3.70200500	0.25260000
H	3.73779900	4.18443800	0.78962100
C	-1.09503900	-0.68236700	0.14649000
H	-1.21061500	-1.09605600	1.15269400
C	-1.02923400	0.78013700	0.11133300
H	-0.96578100	1.34482700	1.04415300
C	-1.79522800	1.42806100	-0.90991300
C	-2.14497900	0.75337900	-2.12467700
H	-2.36140700	1.31837500	-3.02671900
C	-2.20767400	-0.59797600	-2.09427700
H	-2.47478300	-1.16330200	-2.98442900
C	-2.13361300	-1.27227200	-0.79177600
H	-2.01670200	-2.35675800	-0.90559800
C	-3.55416200	-0.99580600	-0.09573700
C	-3.97905500	0.20730900	0.12888200
C	-4.21136900	-2.25825400	0.33860800
C	0.04348900	-3.49401100	1.11383100
H	0.09526800	-3.07998500	2.12703900
H	0.33388900	-4.55067400	1.15469800
H	-0.98849400	-3.42630000	0.75889400
C	2.79839700	-3.08873200	0.78602800

H	2.80399200	-4.16606400	0.99024200
H	2.91845300	-2.54532900	1.73214300
H	3.64466000	-2.84408900	0.13670500
C	1.13723700	-3.59505400	-1.51805900
H	0.16736800	-3.44126800	-2.00558700
H	1.27013600	-4.66169000	-1.30082500
H	1.92037300	-3.26813400	-2.21054100
C	-2.75762300	3.45203100	-1.69457800
H	-2.89337900	4.44987100	-1.27347900
H	-3.73309900	2.97352100	-1.83066900
H	-2.22519200	3.53722500	-2.64956400
F	-3.42728800	-2.96683200	1.18816600
F	-5.37509900	-2.06908000	0.96226100
F	-4.45242500	-3.08719800	-0.69932000
N	1.40704500	0.06175000	1.85680500
N	2.55106000	0.66626200	2.26455400
N	3.16505500	-0.28866000	-0.59898000
N	4.02783900	0.31479300	0.25179100
N	1.63353600	2.01369000	-0.33546700
N	2.68031800	2.39242900	0.44138600
N	0.78009800	-0.28990800	-2.12524300
O	0.74916200	-0.44482000	-3.32296800
O	-1.97606600	2.72741200	-0.73908500
P	1.20587300	-2.60411200	0.01512500
W	0.95399600	-0.11293000	-0.37295900
O	-5.38415000	2.04874900	-0.24766500
C	-4.57764700	1.41600600	0.43926400
O	-4.10957500	1.90671500	1.63560200
C	-4.48776000	3.24233500	1.90988100
H	-5.57637800	3.35782100	1.96495800
H	-4.10578700	3.93543200	1.14883100
H	-4.04627200	3.49288800	2.87826200

Structure TS1A-*p*

Electronic energy: -2349.15732050 a.u.

Free energy: -2348.691269 a.u., -1473826.0230 kcal/mol

Imaginary vibration: 378.25i cm^{-1}

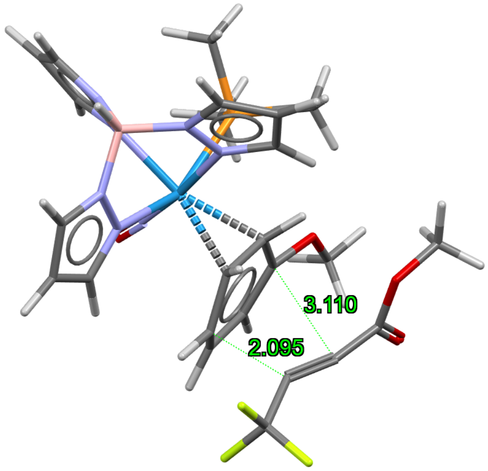

B	3.35156200	1.57981800	1.25411700
H	4.21719200	2.17341400	1.83761900
C	0.76789200	-0.33148700	2.94252400
H	-0.15347600	-0.89914200	2.87118900
C	1.48125800	0.04279600	4.08257200
H	1.24630000	-0.17690200	5.11437100
C	2.55110700	0.77122700	3.59361300
H	3.36520400	1.27187100	4.10276300
C	3.90967100	-0.56616900	-1.59516400
H	3.45635000	-1.12213000	-2.40864600
C	5.25475700	-0.28174400	-1.34501100
H	6.11451000	-0.57005500	-1.93329000
C	5.23868300	0.46749900	-0.18117000
H	6.04085600	0.93407600	0.37703600
C	1.00503300	3.15906800	-1.03161400
H	0.19515400	3.03068200	-1.74029500
C	1.71707200	4.31182600	-0.68752500
H	1.58074100	5.31394700	-1.06906900
C	2.63661000	3.88144500	0.25073800
H	3.40389800	4.41689100	0.79605400
C	-1.14981600	-0.71968100	0.13142000
H	-1.23382500	-1.21164500	1.10241300
C	-1.06153000	0.74050200	0.06909500
H	-0.99315800	1.25853100	1.03122700
C	-1.91622300	1.41459200	-0.91043000
C	-2.39719500	0.70910900	-2.01513200
H	-2.86612100	1.25106000	-2.83463100
C	-2.45274800	-0.68651300	-1.99687900
H	-2.96766700	-1.22014600	-2.78902500
C	-1.95050700	-1.35855900	-0.89130400
C	-4.18539000	0.26507700	0.53774300
C	-3.77079000	1.34311100	0.06140400
C	0.35054600	-3.47569600	1.22828300
H	0.49021200	-3.03647800	2.22252200
H	0.70313200	-4.51396100	1.25430300
H	-0.71173600	-3.46248700	0.97043100
C	3.02023800	-2.93931200	0.59771400
H	3.10243900	-4.01248000	0.80808600

H	3.20569900	-2.37959200	1.52391100
H	3.78429900	-2.65957100	-0.13374900
C	1.16235100	-3.57018700	-1.51204700
H	0.13958500	-3.47605200	-1.89634100
H	1.37530800	-4.62466300	-1.29854000
H	1.85199500	-3.21189500	-2.28436900
N	1.36744800	0.12958300	1.83763900
N	2.46456500	0.81082200	2.25203000
N	3.14034800	-0.04228200	-0.63588300
N	3.96267600	0.59492100	0.22972800
N	1.45834300	2.10850900	-0.34421500
N	2.46780100	2.55843300	0.44377900
N	0.73296000	-0.24500500	-2.14656500
O	0.66711200	-0.37832800	-3.34708500
P	1.33280500	-2.54529900	-0.00812900
W	0.92824400	-0.07217400	-0.39593700
O	-2.14460100	-2.66879300	-0.68010600
C	-2.96838200	-3.39878000	-1.58304500
H	-2.52298300	-3.43011100	-2.58566400
H	-3.02528700	-4.41431200	-1.18445600
H	-3.97422700	-2.96721100	-1.62396100
C	-4.35495300	-1.10144600	0.87141200
O	-5.05991200	-1.90448100	0.27895900
O	-3.66487000	-1.41208400	1.99640200
C	-3.72786800	-2.78022900	2.38141300
H	-3.46845500	-3.43867700	1.54559200
H	-3.00364200	-2.90023400	3.19119100
H	-4.72840800	-3.04574100	2.74156600
C	-4.15821200	2.76399300	0.03679800
F	-3.27642500	3.54009000	0.69116000
F	-4.23763800	3.24732400	-1.21378600
F	-5.35056000	2.96689600	0.60882400
H	-1.83267700	2.49746300	-0.98673300

Structure Int1-*p*

Electronic energy: -2349.17117745 a.u.

Free energy: -2348.702912 a.u., -1473833.3291 kcal/mol

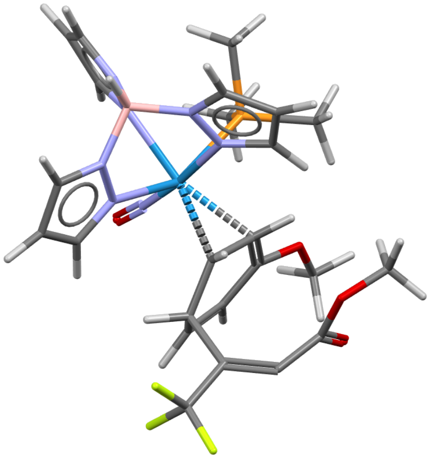

B	2.96301800	1.93442900	1.29365800
H	3.72224700	2.64744600	1.88987800
C	0.85215200	-0.53092800	2.91514100
H	0.05165700	-1.25811000	2.83206100
C	1.52171800	-0.09516000	4.05883900
H	1.36761700	-0.41471000	5.07962700
C	2.41682300	0.85143400	3.59276800
H	3.13830300	1.46844500	4.11369600
C	3.80093100	0.10129100	-1.70652300
H	3.42611400	-0.48215100	-2.54069400
C	5.07897800	0.61332600	-1.47255300
H	5.95463600	0.52330900	-2.09965300
C	4.96901400	1.27970400	-0.26333600
H	5.69328600	1.85286600	0.30207700
C	0.36638200	3.15690400	-0.93057300
H	-0.38599000	2.92007600	-1.67323900
C	0.86396900	4.40219900	-0.53825700
H	0.56440500	5.37693000	-0.89628100
C	1.82740100	4.10573000	0.40704600
H	2.48621900	4.74700400	0.97920300
C	-1.07220700	-1.11023700	0.18392700
H	-0.97817100	-1.68255900	1.10548700
C	-1.22044800	0.34412500	0.20328900
H	-1.22292100	0.76224000	1.21428100
C	-2.29748600	0.94958000	-0.65918000
C	-2.59372000	0.15716800	-1.86956900
H	-3.14306800	0.65046000	-2.67017900
C	-2.32730300	-1.16732500	-1.97163000
H	-2.66117800	-1.73533100	-2.83352500
C	-1.68378000	-1.82356400	-0.88116400
C	-4.27243600	0.17918500	0.85875300
C	-3.67804300	1.07030400	0.07207300
C	1.00599300	-3.60787200	0.98465000
H	1.01324200	-3.20927400	2.00574100
H	1.58824000	-4.53713800	0.97001200
H	-0.02248100	-3.82545600	0.68632700
C	3.50311800	-2.44118800	0.48348600
H	3.82048400	-3.48040100	0.63050700
H	3.53724600	-1.91890500	1.44793100
H	4.19762600	-1.95031500	-0.20454800

C	1.88048000	-3.33006900	-1.73099800
H	0.87053200	-3.45814400	-2.13867800
H	2.33912600	-4.31509800	-1.58324300
H	2.47197200	-2.76111900	-2.45704300
N	1.31505700	0.09964000	1.82760100
N	2.27768800	0.95217900	2.25928400
N	2.98562000	0.41971000	-0.69526700
N	3.70848000	1.14620800	0.18808400
N	0.97628300	2.17823500	-0.25734000
N	1.88247000	2.76843500	0.56387900
N	0.60888700	-0.14407200	-2.14790500
O	0.53963000	-0.20528800	-3.35100500
P	1.78666200	-2.40863900	-0.15697900
W	0.83303500	-0.04842500	-0.39327000
O	-1.65431600	-3.13484100	-0.78149700
C	-2.41089900	-3.94430500	-1.68887000
H	-2.01107300	-3.87398100	-2.70605700
H	-2.30525900	-4.96768800	-1.32713900
H	-3.46375300	-3.64918200	-1.66323300
C	-3.89440300	-1.13422500	1.22768200
O	-4.20909000	-2.18538600	0.66656700
O	-3.23543600	-1.16083700	2.43824600
C	-2.97618200	-2.45421400	2.94553100
H	-2.31496900	-3.03391200	2.28475600
H	-2.49039600	-2.31535100	3.91598500
H	-3.89802600	-3.03232200	3.08273700
C	-4.32907500	2.37777600	-0.23340500
F	-3.60523500	3.43003700	0.22498000
F	-4.43796700	2.59383200	-1.57102700
F	-5.55527900	2.51799600	0.27166400
H	-2.01248300	1.96710300	-0.95855400

Structure TS1B-*p*

Electronic energy: -2349.16397556 a.u.

Free energy: -2348.697860 a.u., -1473830.1589 kcal/mol

Imaginary vibration: 74.14i cm^{-1}

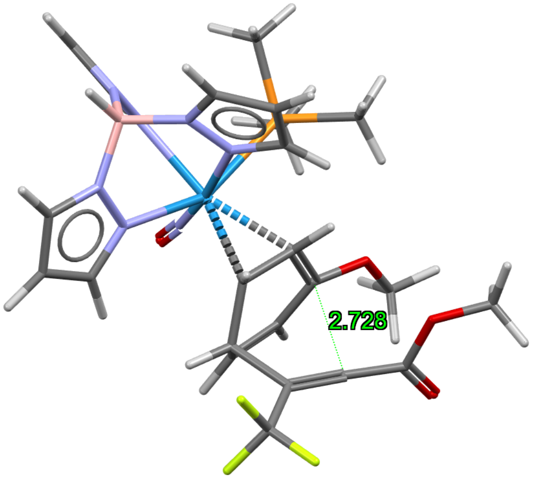

B	3.45097800	1.50814000	1.17972600
H	4.34211700	2.09954000	1.72498000
C	0.79308600	-0.18830900	2.97950900
H	-0.14600700	-0.72812800	2.94295300
C	1.52277800	0.22587800	4.09463300
H	1.28293900	0.07512300	5.13759900
C	2.61670800	0.88527000	3.56282100
H	3.45038800	1.38425900	4.04094000
C	3.90342000	-0.85812200	-1.50908300
H	3.42390400	-1.44306100	-2.28642900
C	5.26072000	-0.62804800	-1.27188400
H	6.10518900	-1.00213500	-1.83333800
C	5.28204100	0.20272200	-0.16441800
H	6.10671300	0.66550300	0.36332700
C	1.17814500	3.02724200	-1.21908200
H	0.36635700	2.88473200	-1.92305700
C	1.93397900	4.17091000	-0.94723100
H	1.83708900	5.15077800	-1.39298100
C	2.83495600	3.76664200	0.02053300
H	3.62162600	4.30630100	0.53300000
C	-1.17212200	-0.63725700	0.21350400
H	-1.28324100	-1.06137800	1.21240500
C	-0.97504400	0.79993900	0.03043500
H	-0.91398500	1.36693300	0.96618400
C	-1.93200400	1.44328000	-0.94758000
C	-2.12944900	0.65573500	-2.16622300
H	-2.33583900	1.15203900	-3.11220300
C	-2.25002000	-0.68901900	-2.05054700
H	-2.54907500	-1.30987200	-2.88965400
C	-2.00646900	-1.27469900	-0.76947100
C	-3.97351200	0.37720400	0.14996100
C	-3.37058700	1.46104300	-0.21667300
C	0.16964900	-3.41592700	1.41721800
H	0.31013400	-2.93858800	2.39330700
H	0.46056800	-4.47020100	1.49984300
H	-0.88476600	-3.35643000	1.13390800
C	2.88553900	-3.04530800	0.82311400
H	2.90651700	-4.10513700	1.10375000

H	3.09074600	-2.43574500	1.71281200
H	3.67056900	-2.85573000	0.08502400
C	1.03051500	-3.68650000	-1.29242800
H	0.01097000	-3.57863300	-1.68241300
H	1.20942800	-4.73690300	-1.03322800
H	1.72771600	-3.38273400	-2.08064800
N	1.40465200	0.18616000	1.84860500
N	2.52736300	0.85006800	2.22151100
N	3.16123100	-0.22788200	-0.59282600
N	4.01449200	0.42534600	0.22957100
N	1.59061500	2.00687700	-0.46366200
N	2.61509000	2.46667400	0.29779600
N	0.77924300	-0.42432100	-2.10197300
O	0.76893300	-0.65209100	-3.28880100
P	1.23123100	-2.60485500	0.16613700
W	0.94581600	-0.13477500	-0.36379400
O	-2.38287300	-2.51189900	-0.47917100
C	-3.24237000	-3.21329800	-1.38641100
H	-2.71076700	-3.46396900	-2.31179000
H	-3.52333100	-4.13587500	-0.87550600
H	-4.13825600	-2.62025500	-1.59420300
C	-4.66579600	-0.75418700	0.54559800
O	-5.54252800	-1.35328900	-0.08285500
O	-4.23323700	-1.19585800	1.77433700
C	-4.80617500	-2.41899400	2.19559800
H	-4.58874000	-3.23600200	1.49604700
H	-4.35929700	-2.64713100	3.16718000
H	-5.89451800	-2.34429800	2.30547500
C	-3.81559800	2.85259900	0.07272900
F	-2.91601900	3.51420200	0.83472100
F	-3.94476500	3.58594800	-1.05349900
F	-4.98499200	2.91787200	0.71238100
H	-1.66236900	2.48215800	-1.16460700

Structure TS2-*d*

Electronic energy: -2349.16210229 a.u.

Free energy: -2348.699434 a.u., -1473831.1466 kcal/mol

Imaginary vibration: 470.81i cm^{-1}

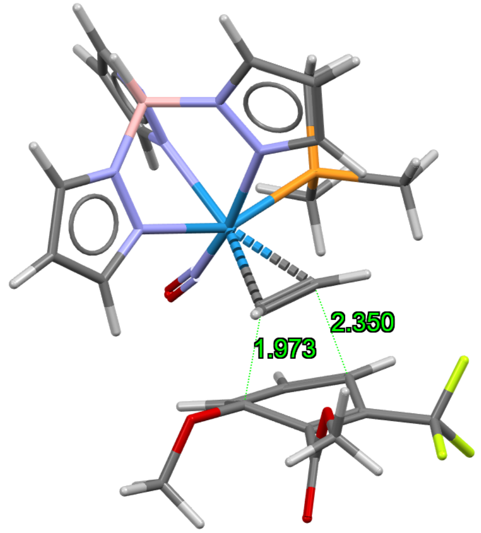

B	3.49576900	1.57372500	1.17615300
H	4.38241400	2.19195500	1.70021000
C	1.09268800	-0.44042100	3.00472200
H	0.18277100	-1.02839400	2.97419800
C	1.88497900	-0.08757500	4.09904900
H	1.73608400	-0.34681300	5.13777600
C	2.89636900	0.68589700	3.55585800
H	3.73464000	1.19144300	4.01908800
C	3.89803900	-0.58728700	-1.68436700
H	3.39347600	-1.14042100	-2.46916200
C	5.25245100	-0.27957000	-1.53021500
H	6.07494000	-0.56420300	-2.17125800
C	5.30361700	0.49087200	-0.38081300
H	6.13410700	0.97892800	0.11416200
C	0.79063700	3.11835300	-0.71935900
H	-0.13674100	2.97089400	-1.26398400
C	1.47470800	4.29339700	-0.39491700
H	1.21226600	5.30791100	-0.66038900
C	2.55997600	3.86919300	0.35045700
H	3.36262700	4.42312100	0.82148400
C	-0.91295600	-0.78900200	0.40510800
H	-1.28593500	-1.44496900	1.18701600
C	-1.02400700	0.53189100	0.15206700
H	-1.32078500	1.34836500	0.81244500
C	-2.38834500	0.94019800	-1.21271700
C	-1.92459600	0.18996600	-2.34048400
H	-1.40120600	0.69289100	-3.15014300
C	-1.94736600	-1.16708400	-2.22272000
H	-1.43199200	-1.79653900	-2.94455500
C	-2.50527300	-1.72392200	-1.04813400
H	-2.47346100	-2.80357400	-0.90111200
C	-3.52127600	-0.98523100	-0.36967000
C	-3.50577700	0.37435600	-0.47933800
C	-4.41456200	-1.73753800	0.55064900
C	0.37975400	-3.59849200	1.09284300
H	0.36263100	-3.20439800	2.11480300

H	0.75648900	-4.62796400	1.11969300
H	-0.64195400	-3.60192600	0.69863800
C	3.10572500	-2.91786100	0.79358600
H	3.23575800	-3.99463500	0.95635200
H	3.17161600	-2.40029600	1.75902200
H	3.91357600	-2.55451900	0.14950000
C	1.50374000	-3.55612500	-1.51052600
H	0.51050800	-3.52070700	-1.97361900
H	1.77222100	-4.59986900	-1.30749300
H	2.22404900	-3.12792900	-2.21509000
C	-3.11718100	2.88008400	-2.33084600
H	-2.69407200	2.62903900	-3.31316400
H	-3.09406200	3.96640300	-2.20573700
H	-4.16008000	2.53692800	-2.29137100
F	-5.35283800	-0.97266800	1.12386700
F	-5.05580900	-2.73698500	-0.07667500
F	-3.71611300	-2.31771800	1.54726300
N	1.59514200	0.07440600	1.87627300
N	2.70434000	0.76900500	2.22728800
N	3.18796600	-0.05797800	-0.68302300
N	4.05689000	0.60593000	0.11273700
N	1.42493200	2.05955300	-0.21017700
N	2.51538500	2.52707800	0.45123700
N	0.82446600	-0.35971300	-2.06577600
O	0.96582100	-0.55943600	-3.25734100
O	-2.35247600	2.32642600	-1.27029000
P	1.46989900	-2.58159600	0.03417100
W	0.97220700	-0.12929600	-0.30523800
O	-5.38709400	1.83276400	-0.18150000
C	-4.39392300	1.30969800	0.26985900
O	-3.93457400	1.52116100	1.50713400
C	-4.74475800	2.37541200	2.31772400
H	-5.73991600	1.93914200	2.45432200
H	-4.23486200	2.45383100	3.27892900
H	-4.84618100	3.36530500	1.86274900

Structure TS2-*p*

Electronic energy: -2349.16582210 a.u.

Free energy: -2348.701397 a.u., -1473832.3784 kcal/mol

Imaginary vibration: 303.64i cm^{-1}

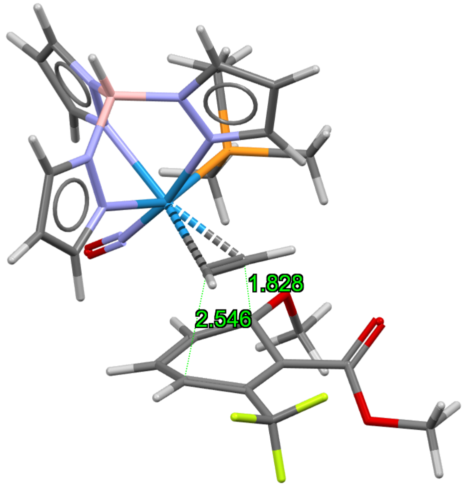

B	3.79250000	1.41949700	0.99225000
H	4.76818700	1.95381600	1.44444000
C	1.09101900	0.09282200	3.02000800
H	0.09787100	-0.34094300	3.04663700
C	1.91884500	0.48150500	4.07517900
H	1.71997900	0.41057800	5.13520400
C	3.04642600	0.99417900	3.45741400
H	3.95007800	1.42719400	3.86810100
C	3.82785300	-1.15005000	-1.54232900
H	3.23824900	-1.72953300	-2.24469000
C	5.21446000	-1.04076800	-1.41095000
H	5.97764000	-1.53883500	-1.99222800
C	5.39403500	-0.13636800	-0.37782700
H	6.29439000	0.27761200	0.05902600
C	1.38624200	3.08598500	-1.17881800
H	0.47193300	3.00389400	-1.75562800
C	2.24178600	4.17585900	-0.99889500
H	2.14936800	5.16813900	-1.41745000
C	3.23468200	3.70045500	-0.16090300
H	4.10992200	4.18477600	0.25408100
C	-1.06675400	-0.33055200	0.34119000
H	-1.46696900	-0.74619100	1.27000100
C	-0.73193800	0.96825100	0.13492800
H	-0.98754600	1.91474900	0.60879900
C	-2.47224100	1.52701100	-1.63762800
C	-1.91789500	0.57316700	-2.52277700
H	-1.43409900	0.89689900	-3.44254500
C	-1.90161800	-0.73076100	-2.14111400
H	-1.41945800	-1.50615800	-2.73115100
C	-2.32283600	-1.02983800	-0.78857800
C	-3.46174100	-0.23237800	-0.32511800
C	-3.43343700	1.09671000	-0.69210100
C	-0.02573900	-3.12020100	1.69491600
H	0.17949600	-2.61120800	2.64323300
H	0.16068100	-4.19296500	1.82935900
H	-1.07094700	-2.96469600	1.41196200
C	2.71042100	-3.02649500	1.04703100
H	2.64755900	-4.06173600	1.40359400

H	2.99262400	-2.37694800	1.88586600
H	3.48976700	-2.95972400	0.28133000
C	0.77085100	-3.63842000	-0.99700700
H	-0.27277500	-3.51731100	-1.31126500
H	0.94258700	-4.67671800	-0.68823700
H	1.42141600	-3.39521500	-1.84420700
N	1.68221300	0.34228400	1.84589800
N	2.88447900	0.90067200	2.12543000
N	3.21664600	-0.38122300	-0.63557300
N	4.18469700	0.24164000	0.07514400
N	1.83433100	2.02745600	-0.50010600
N	2.97466800	2.41097600	0.12725800
N	0.84222300	-0.48672300	-2.01143700
O	0.98552000	-0.86136600	-3.15747500
P	1.08387500	-2.48843600	0.38675500
W	1.02318800	-0.04619500	-0.28917300
O	-2.32902700	-2.38274600	-0.45328900
C	-3.26880700	-3.15491900	-1.16871300
H	-3.06423900	-3.15659800	-2.24989600
H	-3.19481200	-4.18006200	-0.79337900
H	-4.29918000	-2.79685600	-1.01683100
C	-4.32521800	-0.75251800	0.75328300
O	-3.94174800	-1.35807400	1.73207500
O	-5.61852500	-0.47282200	0.51769700
C	-6.50298900	-0.73577200	1.60400000
H	-6.21195900	-0.15144700	2.48428200
H	-7.49635400	-0.43322800	1.26843700
H	-6.50346500	-1.79843600	1.86586900
C	-4.26836000	2.14039300	-0.01284600
F	-3.67282300	3.34378700	-0.05255700
F	-5.47316400	2.29478200	-0.58026500
F	-4.48263200	1.87576500	1.28817600
H	-2.39575700	2.58633000	-1.86709800

Structure TS3-*d*

Electronic energy: -2349.10270760 a.u.

Free energy: -2348.639630 a.u., -1473793.6191 kcal/mol

Imaginary vibration: 376.76i cm^{-1}

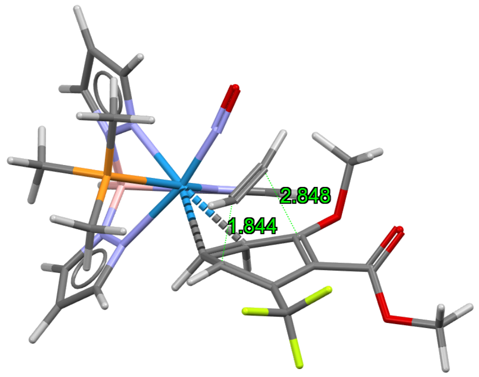

B	3.87760200	1.15734300	1.12044000
H	4.90155000	1.57562000	1.58900300
C	1.80430600	-1.30544200	2.80632500
H	0.89588700	-1.89444500	2.83075300
C	2.86069200	-1.30665800	3.71688400
H	2.95570300	-1.88984900	4.62180900
C	3.75275700	-0.38157600	3.20460600
H	4.71325700	-0.03857800	3.56866000
C	3.53614900	-0.18482900	-2.20913900
H	2.85118000	-0.50623200	-2.98533700
C	4.91619900	0.02343300	-2.26598700
H	5.57345500	-0.11556100	-3.11273800
C	5.25189900	0.46626000	-0.99799900
H	6.20068300	0.77674300	-0.57817200
C	1.02472600	3.26819100	0.31503200
H	-0.00221800	3.32699700	-0.02767500
C	1.87222100	4.27762600	0.77688000
H	1.66416200	5.33605800	0.84694000
C	3.03332200	3.61760700	1.13418800
H	3.96369100	3.98325000	1.55080300
C	-0.88370500	-0.71069100	0.84141300
H	-0.53321300	-1.08072500	1.80766500
C	-0.90567700	0.72674200	0.69068500
H	-0.61296100	1.36848700	1.53186400
C	-2.10011700	1.25083800	0.01496600
C	-2.09134400	-0.69098100	-2.06840700
H	-1.87744200	0.02134000	-2.83927800
C	-2.24129100	-1.69857900	-1.34422300
H	-2.50661100	-2.74823000	-1.41040100
C	-2.14887400	-1.43702800	0.47883800
H	-2.17293000	-2.46951000	0.83099500
C	-3.35566700	-0.71256300	0.64723400
C	-3.32858100	0.63534600	0.22005000
C	-4.63534100	-1.46602000	0.74751600
C	0.33820800	-3.73671500	0.29957000
H	0.34848900	-3.54881400	1.37853900
H	0.76583400	-4.72886600	0.10979300
H	-0.69752800	-3.73037600	-0.05349000
C	3.02117700	-3.02580900	-0.21854100
H	3.12023000	-4.09952600	-0.41911100
H	3.24491900	-2.83967500	0.83906800
H	3.74878500	-2.48146400	-0.82961800

C	1.09164800	-3.00702000	-2.33314600
H	0.07802600	-2.75563600	-2.66451600
H	1.25020300	-4.08867100	-2.42262500
H	1.80506800	-2.48879700	-2.98230000
C	-1.89755400	2.78405600	-1.78386000
H	-0.98771700	2.29771700	-2.16065700
H	-1.80549900	3.86701800	-1.91217100
H	-2.77229300	2.42784000	-2.34008900
F	-5.51711800	-1.13957100	-0.22489700
F	-4.44564900	-2.79352300	0.64097400
F	-5.28179100	-1.27533900	1.91108400
N	2.03376400	-0.44047000	1.80757600
N	3.24171800	0.12135200	2.06801500
N	3.07608800	0.09230300	-0.98361700
N	4.13598000	0.49509800	-0.24684100
N	1.63402600	2.07925100	0.37921000
N	2.87344600	2.30409200	0.88918400
N	0.51150400	0.27000500	-1.88891600
O	0.37813300	0.45783900	-3.08406600
O	-2.07351600	2.55653000	-0.38834000
P	1.31163300	-2.47079600	-0.59850200
W	0.97747500	0.00122700	-0.21009600
O	-4.81628600	1.81034600	-1.25882100
C	-4.57120000	1.35662900	-0.16298600
O	-5.41722300	1.43201000	0.87362200
C	-6.72041300	1.91874700	0.55993800
H	-7.19533600	1.27831500	-0.19160100
H	-7.28929400	1.88691700	1.49057400
H	-6.67847300	2.94441400	0.18088000

Structure TS4

Electronic energy: -988.46822477 a.u.

Free energy: -988.302214 a.u., -620169.0026 kcal/mol

Imaginary vibration: 159.93i cm^{-1}

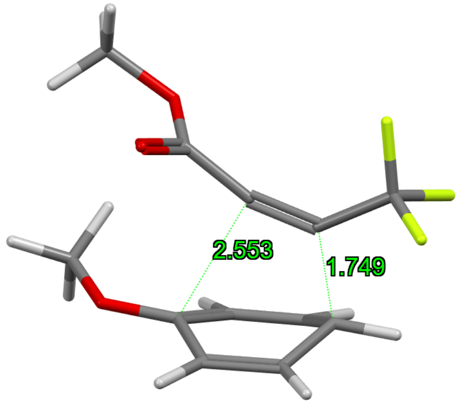

C	0.88135000	-2.11710700	1.08217300
H	1.57754400	-2.34929900	1.88329400
C	-0.45310500	-2.06162100	1.28517500
H	-0.92831100	-2.24586400	2.24462100
C	-1.27395900	-1.64864000	0.18534900
C	-0.81044100	-1.86869900	-1.15252200
H	-1.51609600	-1.93458200	-1.97521300
C	0.52915600	-1.93536300	-1.34096900
H	0.96180400	-2.04536000	-2.33141300
C	1.38697600	-1.64014500	-0.20271300
H	2.45008400	-1.81372400	-0.37215300
C	1.18757800	0.08842700	-0.03020300
C	0.01812300	0.55302800	0.15070300
C	2.52675000	0.71705600	-0.08811400
C	-1.02315200	1.46420600	0.45192700
C	-2.70636000	2.81760100	-0.45312900
H	-3.05216500	3.12548800	-1.44164400
H	-2.43677500	3.70000900	0.13610500
C	-3.40868100	-0.94477900	-0.55159600
H	-4.30515100	-0.56827300	-0.05602800
H	-3.68647600	-1.77029100	-1.21696100
H	-2.93688400	-0.14144700	-1.13050400
F	3.33769000	0.24190500	0.87480100
F	2.47401800	2.04189800	0.05843200
F	3.14509500	0.47132200	-1.25746000
O	-2.54037800	-1.39241600	0.48762700
O	-1.42865100	1.71685400	1.57302500
O	-1.57598500	1.97881600	-0.67413100
H	-3.50410500	2.27738500	0.06927700

Structure TS5

Electronic energy: -988.45070675 a.u.

Free energy: -988.286829 a.u., -620159.3483 kcal/mol

Imaginary vibration: 592.81i cm^{-1}

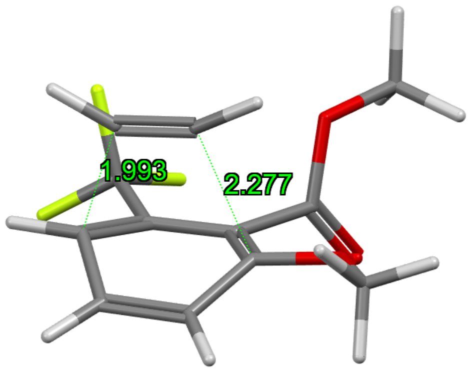

C	1.61557300	-0.21699100	-0.32096000
C	1.99317700	-1.50981000	-0.80298500
H	3.00789500	-1.69702300	-1.14114800
C	1.06728300	-2.49822000	-0.70176300
H	1.30222100	-3.53071900	-0.94298500
C	-0.18528400	-2.16283800	-0.08998500
H	-0.92884500	-2.95343900	0.00746600
C	-0.68015200	-0.84263100	-0.37284500
C	0.23782100	0.16008100	-0.44178600
C	-2.13541400	-0.59391200	-0.20062900
C	3.85337200	0.51315000	-0.12307200
H	4.35395700	1.43423500	0.18250800
H	4.27697900	0.18084800	-1.07911900
H	4.02187000	-0.26167200	0.63745300
F	-2.51298600	0.61927600	-0.62601000
F	-2.88073200	-1.49294800	-0.86033600
F	-2.49194300	-0.68746600	1.09326600
O	2.47520700	0.82263900	-0.24107000
O	-0.09857900	2.31575400	-1.44422000
C	-0.09280700	1.61819500	-0.46038300
O	-0.36540600	2.03927200	0.77770300
C	-0.75519300	3.41218800	0.87835600
H	0.03609800	4.07063300	0.50841500
H	-1.67007300	3.58990100	0.30379400
H	-0.93568500	3.59866100	1.93758400
C	1.26423600	-0.89917400	1.82296700
H	1.92853600	-0.19299000	2.28781900
C	0.42543200	-1.82304600	1.77673900
H	-0.16002700	-2.55145300	2.31468700

Structure TS6-*d*

Electronic energy: -1937.94318772 a.u.

Free energy: -1937.476710 a.u., -1215784.9914 kcal/mol

Imaginary vibration: 39.76i cm^{-1}

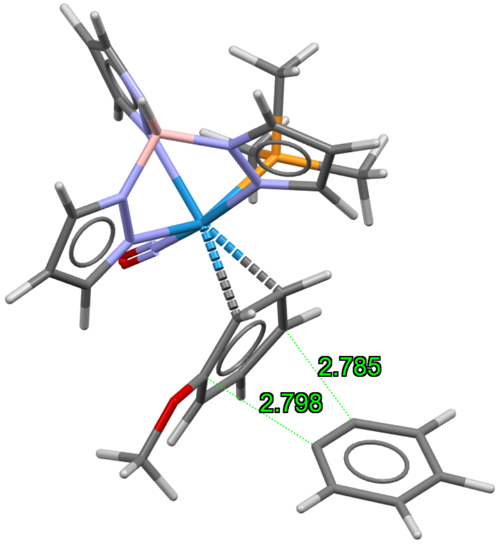

B	2.87604100	1.36380400	1.59778800
H	3.68606700	1.90263400	2.30283500
C	0.30536500	-0.86562500	2.86205700
H	-0.57333500	-1.46626800	2.65661900
C	0.94507900	-0.63093100	4.08075400
H	0.67837600	-1.01494500	5.05520700
C	1.99162200	0.21593300	3.76068800
H	2.75376000	0.67369000	4.37903600
C	3.69456800	-0.44332800	-1.42411100
H	3.31635900	-0.91515900	-2.32438600
C	5.01013400	-0.16251700	-1.04438900
H	5.91793600	-0.37165200	-1.59268300
C	4.88891300	0.46016800	0.18579300
H	5.63510300	0.87845200	0.84995000
C	0.65484700	3.15270600	-0.65478500
H	-0.11025100	3.09415500	-1.41983700
C	1.32808900	4.26803300	-0.14679100
H	1.20612700	5.30328400	-0.43299400
C	2.18431600	3.74931200	0.80642800
H	2.90524800	4.23052900	1.45574000
C	-1.45969800	-0.93471800	-0.09131600
H	-1.53226100	-1.54327700	0.81372500
C	-1.45244400	0.51935200	0.07282000
H	-1.44808600	0.92733200	1.08773900
C	-2.23323300	1.30472200	-0.85951800
C	-2.72771200	0.77130800	-2.03052100
H	-3.24237300	1.39052000	-2.75912100
C	-2.67410100	-0.63398800	-2.21575300
H	-3.14207300	-1.06170300	-3.10086100
C	-2.18175600	-1.45848400	-1.24178500
H	-2.25973500	-2.53976500	-1.36337500
C	-4.58172600	-0.96822900	0.08243500
C	-4.62273300	0.29654400	0.18989600
C	-0.05840900	-3.77756100	0.50958500
H	-0.19049400	-3.50690000	1.56316200
H	0.36148700	-4.78946300	0.45906500

H	-1.03696700	-3.77873800	0.01988600
C	2.64519300	-3.02564600	0.57851700
H	2.77644800	-4.11362500	0.62246900
H	2.59583500	-2.63075500	1.60167700
H	3.51241600	-2.58173800	0.07876600
C	1.31323200	-3.37830600	-1.94958500
H	0.38843800	-3.26440000	-2.52715900
H	1.55581600	-4.44381900	-1.85592100
H	2.11810500	-2.87208700	-2.49398100
C	-3.15571900	3.43344000	-1.30516400
H	-3.21076300	4.40249700	-0.80353800
H	-4.17015900	3.02863400	-1.43063800
H	-2.70183200	3.56949900	-2.29758800
N	0.92404500	-0.21286300	1.87082300
N	1.96017000	0.45287800	2.43702200
N	2.84256300	-0.03666000	-0.47755700
N	3.58273100	0.52098900	0.50879500
N	1.06991500	2.04042000	-0.04633500
N	2.01694700	2.41268800	0.85400200
N	0.52729300	-0.09150900	-2.15960800
O	0.52933100	-0.09146300	-3.37028200
O	-2.36955800	2.59569600	-0.48518300
P	1.09130400	-2.60589200	-0.30715100
W	0.61917000	-0.13043300	-0.39274600
C	-5.50387700	1.00621100	0.98758900
C	-6.39123600	0.16890900	1.68894900
C	-6.34464500	-1.22473500	1.56516400
C	-5.40669100	-1.86681100	0.73482600
H	-5.54124100	2.09001200	1.09515500
H	-7.13441100	0.61706200	2.34820300
H	-7.05353100	-1.83064300	2.12915800
H	-5.37646900	-2.95201400	0.64825200

Structure TS6-*p*

Electronic energy: -1937.94467812 a.u.

Free energy: -1937.479557 a.u., -1215786.7779 kcal/mol

Imaginary vibration: 40.35i cm^{-1}

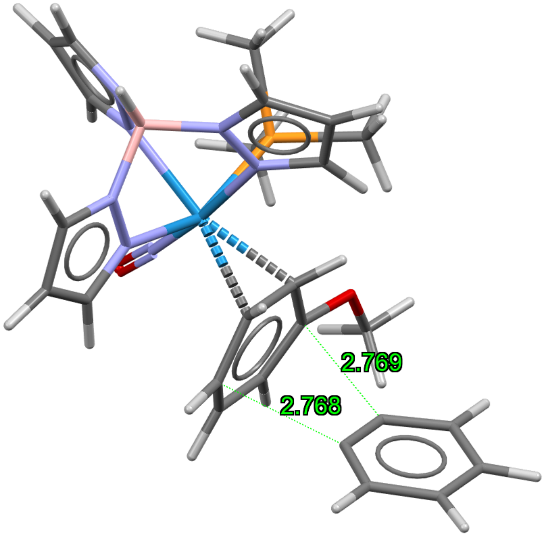

B	-3.16096200	-1.67971100	0.91082300
H	-4.06890800	-2.33023400	1.35357800
C	-0.29795800	-0.65424400	2.89294600
H	0.67498800	-0.17718700	2.92568200
C	-0.99969900	-1.29133100	3.91831100
H	-0.69792800	-1.42110000	4.94799400
C	-2.16576500	-1.72821700	3.31522300
H	-3.01032800	-2.27881800	3.71061000
C	-3.56999500	1.29895200	-1.08728000
H	-3.08627000	2.03122600	-1.72490500
C	-4.92829800	1.09219000	-0.83007500
H	-5.77034000	1.63867700	-1.23100300
C	-4.95479400	0.01864100	0.04292400
H	-5.78334800	-0.51230500	0.49504700
C	-1.14116600	-2.70971700	-1.93176900
H	-0.34905500	-2.45735500	-2.62705300
C	-1.98497300	-3.82438500	-1.90210100
H	-1.99899500	-4.66379100	-2.58314000
C	-2.79870600	-3.61142600	-0.80512900
H	-3.60577000	-4.20190500	-0.38937400
C	1.55441200	0.34064500	0.17506600
H	1.72851000	0.46736600	1.24668000
C	1.27957400	-1.00237600	-0.33796300
H	1.15055700	-1.79536800	0.40484700
C	1.92478300	-1.40471100	-1.57350800
C	2.54624900	-0.49050100	-2.38068500
H	2.95615000	-0.79724400	-3.34127000
C	2.79551800	0.82590800	-1.92023400
H	3.37501500	1.50783800	-2.53549200
C	2.41895000	1.17459800	-0.64181100
C	4.58638200	-0.37354000	0.11389700
C	4.35427300	-1.56579900	-0.25741600
C	0.44977700	2.78201000	2.18530400
H	0.23094500	2.11420900	3.02631700
H	0.27497800	3.81556300	2.50855600
H	1.49546700	2.66892800	1.88955900

C	-2.27227500	2.81402900	1.57337000
H	-2.18540600	3.75661200	2.12734600
H	-2.54500800	2.01293300	2.27311700
H	-3.07061800	2.90994300	0.83150800
C	-0.38263300	3.79636100	-0.35909100
H	0.62305900	3.70917500	-0.78696100
H	-0.47671600	4.75810700	0.15979800
H	-1.10743100	3.75323600	-1.17960700
N	-0.99192300	-0.69573000	1.74913400
N	-2.14116700	-1.36233700	2.02104500
N	-2.83031100	0.42428200	-0.39915800
N	-3.68716700	-0.36343000	0.29180000
N	-1.42142500	-1.88505800	-0.92037000
N	-2.44749500	-2.44448200	-0.22926700
N	-0.48835300	0.84911800	-1.96368400
O	-0.46531900	1.37296100	-3.05531100
P	-0.66592600	2.39752200	0.78322200
W	-0.62418300	0.14053700	-0.34834500
C	5.04669200	-2.69386600	0.14312200
C	6.08714000	-2.38927100	1.04118100
C	6.34567100	-1.07780300	1.45694400
C	5.57958900	0.01020900	0.99868100
H	4.85009800	-3.71795600	-0.17036200
H	6.70785900	-3.19877900	1.42469700
H	7.16160100	-0.89551300	2.15609600
H	5.78228900	1.02771400	1.33265000
O	2.75491000	2.34364500	-0.04484100
C	3.67348600	3.18395800	-0.71320500
H	3.25428000	3.57921300	-1.64898600
H	3.88455600	4.01698700	-0.03844600
H	4.60679500	2.64856900	-0.93786500
H	1.84292500	-2.44401400	-1.89004900

Structure TS7-*d*

Electronic energy: -1938.00050517 a.u.

Free energy: -1937.532552 a.u., -1215820.0328 kcal/mol

Imaginary vibration: 425.79i cm^{-1}

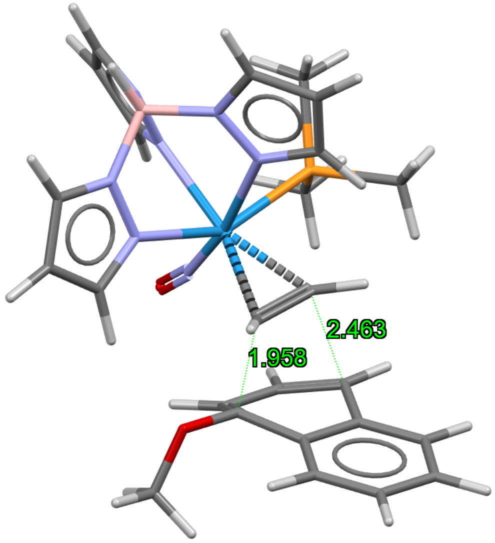

B	3.15430600	1.48987800	1.08198700
H	4.09249500	2.07370800	1.55355400
C	0.98284400	-0.70655800	2.98742800
H	0.07658400	-1.30108700	3.00541600
C	1.89238200	-0.43831700	4.01267700
H	1.86236600	-0.78710700	5.03528700
C	2.83348000	0.39197000	3.42857800
H	3.71675400	0.86734100	3.83692200
C	3.23441700	-0.41471900	-1.98044100
H	2.64547100	-0.90817300	-2.74626300
C	4.59674000	-0.10290600	-1.95622900
H	5.34122500	-0.32171800	-2.70874300
C	4.77670300	0.56929500	-0.75912100
H	5.65675800	1.02480500	-0.32209100
C	0.24390000	3.15529500	-0.35106000
H	-0.73950100	3.04222800	-0.79722400
C	0.95840800	4.30670400	-0.00702400
H	0.66527400	5.33698700	-0.15290800
C	2.12257600	3.83381000	0.57090600
H	2.97258400	4.35512100	0.99369900
C	-1.30147600	-0.84851500	0.61339400
H	-1.61856700	-1.59144800	1.34038100
C	-1.46156500	0.48196900	0.45903800
H	-1.68988600	1.25613600	1.19540100
C	-2.94468400	0.97950300	-0.71903700
C	-2.61791300	0.32077300	-1.93571400
H	-2.14793100	0.87776100	-2.74296300
C	-2.65465700	-1.05343700	-1.93678100
H	-2.20398600	-1.60789500	-2.75831100
C	-3.12177000	-1.72210800	-0.79709000
H	-3.09233400	-2.81224900	-0.75907200
C	-4.03127100	-1.04676000	0.08777400
C	-3.98951300	0.36608100	0.10230800
C	0.08642000	-3.69758600	0.92806700
H	0.19366200	-3.38623100	1.97297400
H	0.47259300	-4.71859800	0.82378200

H	-0.97515300	-3.68815900	0.66114500
C	2.74617100	-2.95846900	0.33647400
H	2.89588600	-4.04346400	0.39362600
H	2.93777400	-2.51883300	1.32363300
H	3.46310900	-2.53695300	-0.37596100
C	0.85758600	-3.42226400	-1.78485900
H	-0.18778400	-3.35681500	-2.10921300
H	1.14950900	-4.47661300	-1.70786400
H	1.48033500	-2.92824900	-2.53788900
C	-3.78230900	2.99662500	-1.59488400
H	-3.74412200	4.07165100	-1.39343800
H	-4.81556500	2.64563200	-1.45677500
H	-3.49499700	2.82487500	-2.64197800
N	1.35379800	-0.09090500	1.85867000
N	2.49287100	0.58563400	2.14187900
N	2.64214100	0.01989400	-0.86383200
N	3.59414600	0.62619000	-0.11907100
N	0.93418500	2.06460800	-0.00983000
N	2.09250100	2.48749500	0.56068300
N	0.14413700	-0.19951900	-1.99562100
O	0.17457700	-0.29492300	-3.20888900
O	-2.89121700	2.37057400	-0.69136000
P	1.02448500	-2.57917700	-0.17299600
W	0.48108300	-0.11673400	-0.24516700
C	-4.80845200	1.07986500	0.98392700
C	-5.68806100	0.40597500	1.81806700
C	-5.73493900	-0.99462700	1.80826800
C	-4.90648700	-1.71250600	0.96235100
H	-4.73965700	2.16599000	1.01232100
H	-6.33610100	0.96579000	2.48956900
H	-6.42066100	-1.51886800	2.47118200
H	-4.92960300	-2.80248900	0.95889200

Structure TS7-*p*

Electronic energy: -1938.00229051 a.u.

Free energy: -1937.532731 a.u., -1215820.1451 kcal/mol

Imaginary vibration: 408.27i cm^{-1}

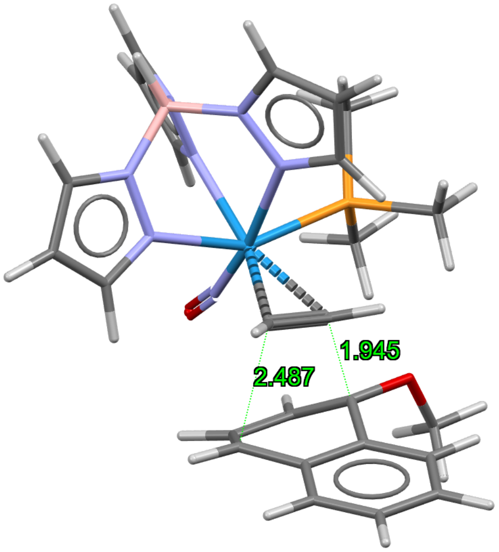

B	-3.49964700	-1.22215400	0.71724200
H	-4.55794200	-1.67809900	1.05636100
C	-0.88014400	-0.25845300	3.03582300
H	0.14149200	0.07838200	3.16941900
C	-1.83281000	-0.60935500	3.99482600
H	-1.72603000	-0.60187500	5.07035700
C	-2.94176000	-0.98450000	3.25639300
H	-3.91566000	-1.34421000	3.56460400
C	-3.06507400	1.45631200	-1.66037100
H	-2.36109900	2.00363100	-2.27826500
C	-4.46257400	1.47889900	-1.65411500
H	-5.11958600	2.07685800	-2.26996900
C	-4.81944600	0.54892500	-0.69246600
H	-5.79066500	0.20562900	-0.35804600
C	-1.08620300	-3.01970900	-1.33765100
H	-0.12203700	-3.00346700	-1.83303100
C	-2.05609700	-4.02479900	-1.28840400
H	-2.02505400	-5.00046200	-1.75265900
C	-3.06694400	-3.49039700	-0.50970700
H	-4.01812400	-3.90156400	-0.19496500
C	1.52354400	0.04200100	0.57129800
H	1.90768100	0.40936300	1.52564700
C	1.12044600	-1.20164100	0.23756200
H	1.23655200	-2.20450400	0.64081300
C	2.85847600	-1.82692000	-1.42712300
C	2.49363600	-0.76416800	-2.26320500
H	1.97998200	-0.95578500	-3.20417100
C	2.64422500	0.52632900	-1.81196800
H	2.26370700	1.37244700	-2.37893100
C	3.03970600	0.69776500	-0.45601400
C	3.99216100	-0.28102900	0.07471700
C	3.83766200	-1.60400900	-0.40002600
C	0.67719000	2.87880600	1.96815300
H	0.37564300	2.32860500	2.86621200
H	0.56153100	3.95233000	2.16225300
H	1.72422700	2.66305200	1.73654100

C	-1.99025100	3.09513700	1.10042600
H	-1.85501600	4.10255000	1.51233800
H	-2.40738800	2.44358300	1.87942600
H	-2.70401000	3.14160500	0.27192800
C	0.18054800	3.59047200	-0.73544900
H	1.23948500	3.38908200	-0.93727800
H	0.06521100	4.62672300	-0.39516000
H	-0.38453200	3.44187900	-1.66183800
N	-1.38143100	-0.40079800	1.80381700
N	-2.65043700	-0.85154500	1.94984000
N	-2.61390400	0.58949900	-0.74888100
N	-3.69645400	0.03236300	-0.15956500
N	-1.48562300	-1.95398500	-0.64092600
N	-2.70823400	-2.24843600	-0.13125600
N	-0.13383300	0.51454400	-1.92478800
O	-0.14875200	0.95308800	-3.06056800
P	-0.37605400	2.41488600	0.54714300
W	-0.49629200	0.01779200	-0.24922200
C	4.60791300	-2.62846100	0.17609900
C	5.52321400	-2.34310000	1.17488000
C	5.67238500	-1.02924200	1.63946700
C	4.89801400	-0.00964300	1.10570600
H	4.47987800	-3.65028600	-0.18135100
H	6.12564200	-3.14142100	1.60410300
H	6.38932300	-0.81082000	2.42852500
H	4.98098700	1.00683900	1.48714300
O	3.17343300	1.99959800	0.02532800
C	4.13517200	2.77571000	-0.66283400
H	3.83046200	2.98624100	-1.69828700
H	4.22796200	3.72512100	-0.12582600
H	5.11779700	2.28176600	-0.68545800
H	2.67827900	-2.85488500	-1.74323500

Structure anisole

Electronic energy: -346.53865662 a.u.

Free energy: -346.437245 a.u., -217392.6534 kcal/mol

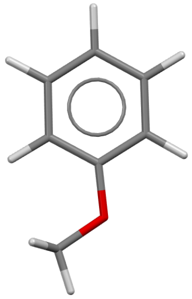

C	-1.84723100	-0.99349000	0.00002300
C	-0.49609800	-1.30065400	-0.00002800
C	0.45611000	-0.27533900	-0.00013400
H	-2.57782700	-1.79974100	0.00012300
H	-0.14756100	-2.33104800	0.00002700
C	-2.27195800	0.33568500	-0.00002600
H	-3.33249000	0.57481200	0.00003000
O	1.75031000	-0.67523800	-0.00021500
C	2.74748100	0.32362500	0.00052300
H	3.70796300	-0.19671400	0.00102800
H	2.68857500	0.95885600	-0.89409000
H	2.68747100	0.95861200	0.89523100
C	-1.32235900	1.34894300	-0.00015100
C	0.04147200	1.05762000	-0.00021800
H	0.76449400	1.86835300	-0.00035400
H	-1.63761200	2.39043300	-0.00021000

REFERENCES:

1. Salomon, R. J.; Lis, Edward C.; Kasbekar, M. U.; Bassett, K. C.; Myers, W. H.; Trindle, C. O.; Sabat, M.; Harman, W. D., Stereoelectronic Effects in Dihapto-Coordinated Complexes of TpW(NO)(PMe_3) and Their Manifestation in Diels−Alder Cycloaddition of Arenes. *Organometallics* **2009**, *28*, 4724-4734.
2. Weatherford-Pratt, J. T.; Bloch, J. M.; Smith, J. A.; Ericson, M. N.; Siela, D. J.; Ortiz, M. R.; Shingler, M. H.; Fong, S.; Laredo, J. A.; Patel, I. U.; McGraw, M.; Dickie, D. A.; Harman, W. D., Tungsten-anisole complex provides 3,6-substituted cyclohexenes for highly diversified chemical libraries. *Science Advances* **2024**, *10*, eadl0885.
3. Welch, K. D.; Harrison, D. P.; Lis, E. C.; Liu, W.; Salomon, R. J.; Harman, W. D.; Myers, W. H., Large-Scale Syntheses of Several Synthons to the Dearomatization Agent {TpW(NO)(PMe3)} and Convenient Spectroscopic Tools for Product Analysis. *Organometallics* **2007**, *26*, 2791-2794.
4. Peschel, L. M.; Belaj, F.; Mösch-Zanetti, N. C., Towards Structural-Functional Mimics of Acetylene Hydratase: Reversible Activation of Acetylene using a Biomimetic Tungsten Complex. *Angewandte Chemie International Edition* **2015**, *54*, 13018-13021.
5. Xu, F.; Qian, X.-Y.; Li, Y.-J.; Xu, H.-C. Synthesis of 4*H*-1,3-Benzoxazines via Metal- and Oxidizing Reagent-Free Aromatic C–H Oxygenation. *Org. Lett.* **2017**, *19* (23), 6332–6335.
